# Tree Nut and Peanut Consumption and Risk of Cardiovascular Disease: A Systematic Review and Meta-Analysis of Randomized Controlled Trials

**DOI:** 10.1016/j.advnut.2023.05.004

**Published:** 2023-05-05

**Authors:** Lauren Houston, Yasmine C. Probst, Mamatha Chandra Singh, Elizabeth P. Neale

**Affiliations:** 1 The George Institute for Global Health, University of New South Wales, Sydney, New South Wales, Australia; 2 School of Medical Sciences, Faculty of Medicine, University of New South Wales, Sydney, New South Wales, Australia; 3 School of Medical, Indigenous and Health Sciences, Faculty of Science, Medicine and Health, University of Wollongong, Wollongong, New South Wales, Australia; 4 Illawarra Health and Medical Research Institute, University of Wollongong, Wollongong, New South Wales, Australia

**Keywords:** nuts, peanuts, CVD, cholesterol, lipids, apo, blood pressure, meta-analysis

## Abstract

Cardiovascular disease (CVD) is the leading cause of death globally. Habitual consumption of tree nuts and peanuts is associated with cardioprotective benefits. Food-based dietary guidelines globally recommend nuts as a key component of a healthy diet. This systematic review and meta-analysis were conducted to examine the relationship between tree nut and peanut consumption and risk factors for CVD in randomized controlled trials (RCTs) (PROSPERO: CRD42022309156). MEDLINE, PubMed, CINAHL, and Cochrane Central databases were searched up to 26 September, 2021. All RCT studies that assessed the effects of tree nut or peanut consumption of any dose on CVD risk factors were included. Review Manager software was used to conduct a random effect meta-analysis for CVD outcomes from RCTs. Forest plots were generated for each outcome, between-study heterogeneity was estimated using the I^2^ test statistic and funnel plots and Egger’s test for outcomes with ≥10 strata. The quality assessment used the Health Canada Quality Appraisal Tool, and the certainty of the evidence was assessed using grading of recommendations assessment, development, and evaluation (GRADE). A total of 153 articles describing 139 studies (81 parallel design and 58 cross-over design) were included in the systematic review, with 129 studies in the meta-analysis. The meta-analysis showed a significant decrease for low-density lipoprotein (LDL) cholesterol, total cholesterol (TC), triglycerides (TG), TC:high-density lipoprotein (HDL) cholesterol, LDL cholesterol:HDL cholesterol, and apolipoprotein B (apoB) following nut consumption. However, the quality of evidence was “low” for only 18 intervention studies. The certainty of the body of evidence for TC:HDL cholesterol, LDL cholesterol:HDL cholesterol, and apoB were “moderate” because of inconsistency, for TG were “low,” and for LDL cholesterol and TC were “very low” because of inconsistency and the likelihood of publication bias. The findings of this review provide evidence of a combined effect of tree nuts and peanuts on a range of biomarkers to create an overall CVD risk reduction.

**Statements of Significance**

This is the first systematic review and meta-analysis of randomized controlled trials to provide a comprehensive picture of whether all or some types of tree nuts and peanuts are preferential for improving CVD risk and if there is a dose response to the effect.

## Introduction

Cardiovascular disease (CVD) is the leading cause of death globally, accounting for 17.9 million lives lost each year (32% of all global deaths) [1]. The high prevalence and mortality rate of CVD is not unique to high-income countries but also to low- and middle-income countries. Low- and middle-income countries account for ∼80% of all CVD deaths, with ∼40% of these deaths defined as premature [2]. Contributing to an increased risk of CVD is the consumption of poor diets. As part of a healthy diet, food-based dietary guidelines globally recommend nuts as a key component, with a typical serving of 15–30 g/d [3,4]. Nuts are rich in fats, making them energy-dense, and concern regarding the impact of nuts on body weight has been reported in the literature [[5], [6], [7], [8]]. A recent review has demonstrated that energy compensation may occur following a meal with nuts, which mitigates this concern [9]. However, nuts are considered to be good sources of unsaturated fatty acids, vitamin E, minerals (for example, magnesium and potassium), plant sterols, polyphenols, and dietary fibers [10]. Tree nuts are defined as dry fruits that contain a seed within the ovary wall that becomes hard at maturity. Tree nuts include almonds, Brazil nuts, cashews, chestnuts, hazelnuts, macadamias, pecans, pine nuts, pistachios, and walnuts. Although peanuts, also known as ground nuts, are botanically classified as a legume rather than a nut, they appear in cuisines in a similar way to that of tree nuts and have a similar nutrient composition. This review offers the advantage of including whole tree nuts, peanuts, and mixed nuts in dietary patterns.

Habitually consuming tree nuts and peanuts has been associated with cardioprotective benefits [11]. The effects of nuts have been shown via improvements to lipid profiles, glucose regulation, and antioxidant effects [[12], [13], [14]] and their ability to mediate inflammation, hyperglycemia, and oxidative stress [15]. In addition, a considerable amount of evidence has been reported from meta-analyses of prospective cohort studies that higher nut consumption is associated with lower CVD incidence and/or CVD mortality [[16], [17], [18], [19], [20], [21]]. However, to date, only 1 meta-analysis of 61 randomized and nonrandomized controlled intervention trials has reported the effects of all tree nuts and dose response on CVD risk factors [22]. Since 2015, the consumption of all nuts has remained largely under investigation within randomized controlled trials (RCTs). Most meta-analyses of RCTs have focused on only 1 type of nut – almonds [23,24], cashews [25,26], peanuts [27], pistachios [28], and walnuts [29] on key CVD risk factors (for example, blood lipids, apolipoproteins, blood pressure, and inflammation). Therefore, the effect of combined tree nut and peanut consumption remains unclear. This review provides an opportunity to advance the understanding of whether all or some types of tree nuts and peanuts are preferential for improving CVD risk and if there is a dose response to the effect.

Despite the known health benefits associated with tree nut and peanut consumption and the promotion of nut intakes through dietary guidance messages, no health claim has been authorized globally for a cause-and-effect relationship between nuts and CVD. However, in 2012 the European Food Safety Authority panel substantiated a health claim related to 30 g/d of walnuts having an improvement in endothelium-dependent vasodilation [30]. Further, in 2014, Food Standards Australia New Zealand considered the relationship between walnuts and endothelium-dependent vasodilation [31] not to be assessable because of the small number of studies and high risk of bias. More recently, the United States Food and Drug Administration (FDA) 2017 approved a qualified health claim for 1.5 oz (42.5 g/d) of macadamia nuts and reduced risk of coronary heart disease (CHD) [32]. The FDA has also approved qualified health claims for nuts (2003) [33] and walnuts (2004) [34]. However, these 2 health claims were approved based on supportive but not conclusive evidence that consuming nuts and walnuts may reduce risk of CHD. The above-mentioned regulatory applications were constructed from the available scientific literature at the time. Since these applications were assessed, new studies have become available. An update of the literature is warranted to determine if the findings from the observational evidence are also demonstrated in experimental studies of nut consumption on CVD outcomes. Therefore, we performed a systematic review and meta-analysis of randomized controlled trials (RCT) studies to quantify the relation between nut consumption and risk of CVD.

## Methods

This systematic review and meta-analysis are reported according to the Preferred Reporting Items for Systematic Reviews and Meta-Analyses (PRISMA) guidelines [35] and checklist (Supplemental Table 1). The protocol was registered in PROSPERO (the international prospective register of systematic reviews, registration no. CRD42022309156).

### Search strategy and study selection

A systematic search was undertaken in MEDLINE (EBSCO), PubMed, CINAHL (EBSCO), and Cochrane Central databases from inception to 26 September, 2021. The search strategy used a combination of keywords and Medical Subject Headings in addition to free-text search terms related to nuts and the cardiovascular outcomes of interest (that is, blood lipids, apolipoproteins, and blood pressure). Both MEDLINE and PubMed databases were searched to ensure that recent studies were identified [36,37]. An example of the detailed search strategy for the MEDLINE database is presented in Supplemental Table 2. Studies were restricted to those published in the English language only.

The records identified from the search strategy were imported to Covidence software (Covidence Systematic Review software; Veritas Health Innovation), and duplicates were removed. Records were screened by title and abstract by 1 reviewer (LH), and subsequent full-text candidate articles were assessed by 2 independent reviewers (LH and EPN). Where a study protocol was retrieved by the search (for example, a clinicaltrials.gov listing), a search was conducted to determine if a relevant article had been published from the study. Any discrepancies with full-text screening were resolved by the research team (LH, EPN, YCP) by discussion to consensus. Reference lists of the included articles were manually searched to identify additional articles.

In the case that the results from 1 study were reported in multiple articles, all articles were checked (LH, EPN, YCP) to avoid duplication of study populations in the analysis or oversight of new information on included outcomes. Where different information for included outcomes was reported across the articles, all relevant articles were included. Where the same outcomes from a single study were reported across multiple articles, decisions relating to article inclusion were based firstly on the length of the follow-up for the outcome and then on the total sample size, with articles including larger sample sizes prioritized.

### Study eligibility criteria

Studies were included if they: *1*) were RCTs (parallel or cross-over); *2*) assessed the effects of tree nut or peanut consumption [whole tree nuts (almonds, Brazil nuts, cashew, chestnut, hazelnut, macadamia, pecan, pine nut, pistachio, or walnut), mixed nuts, peanuts, or their related products (for example, nut oils, nut powders, nut flours, and new genetic varieties of nuts)] of any dose on CVD risk factors [total cholesterol (TC), low-density lipoprotein (LDL) cholesterol, high-density lipoprotein (HDL) cholesterol, triglycerides (TG), apolipoprotein A-I (apoA-I), apolipoprotein B (apoB), systolic blood pressure (SBP), and diastolic blood pressure (DBP)]; *3*) with a control or comparator group (an intervention with a lower amount of nuts or a usual diet without the addition of nuts); *4*) in adults or children (>2 y of age) and healthy individuals or with chronic noncommunicable diseases such as diabetes, hyperlipidemia, or hypertension; *5*) a study duration of ≥3 wk; and *6*) outcomes available as a change from baseline or at the end of the intervention as final values.

Studies were excluded if they were: *1*) animal studies, observational studies, nonrandomized study design, reviews, commentaries, or clinical trials without a control group; *2*) in children <2 y or acutely ill individuals; *3*) targeting coconut and coconut products, as their nutrient profiles differ from the aforementioned “nuts” [38]; or *4*) unable to isolate the effect of nut consumption on the health outcome.

### Data extraction

Outcome data were extracted by the first researcher (LH) and confirmed by a second researcher (MCS). A third researcher (ES) also performed quality checking on a 10% random sample of the data extracted for inclusion in the meta-analysis using source data verification [39]. The following data were extracted from each study: study details, country, study design, type of nut, nut dose, study duration, sample size and loss to follow-up, participant details [age, sex, body mass index (BMI, in kg/m^2^), and health status], intervention, background diet, a method to measure health effect and method to measure food consumption, results, and quality appraisal.

Mean changes in relevant outcomes were extracted where possible, and in the case that these data were not available, mean final values were retrieved as recommended by the Cochrane Handbook [40]. Study authors were contacted for additional information if the published article did not provide sufficient information. Where a study involved >1 intervention group meeting the inclusion criteria, data for the 2 intervention groups were combined as recommended by the Cochrane Handbook [40], with the sample size divided across the 2 groups in the case of cross-over studies to avoid a unit-of-analysis error. In the case of the Prevention with Mediterranean Diet (PREDIMED) study [41], which included 2 intervention arms featuring a Mediterranean diet supplemented with either nuts or olive oil and a low-fat control arm, data from the arm receiving the Mediterranean diet with olive oil was treated as the comparator group, and included studies were required to compare the nut group with the olive oil group. This decision was made to ensure that the outcomes were not confounded by differences in the background diet of the 2 groups. Data were converted to common units, for example, mg/dL was converted to mmol/L by multiplying by 0.0259 for LDL cholesterol, HDL cholesterol, and TC and by multiplying by 0.0113 for TG. Standard deviations (SDs) were imputed from standard errors (SEs) or 95% confidence intervals (CIs) using formulas in the Cochrane Handbook [42]. Where studies reported medians and 25th and 75th percentiles, means and SDs were imputed using the formulas developed by Wan et al. [43]. Where imputation was not possible (for example, where geometric means were reported or where a median was reported with 95% CIs), the values were used in place of the means. Where studies did not report sufficient data to be included in the meta-analysis (for example, if they did not report either SDs, SEs, 95% CIs, or interquartile range), the results were reported descriptively.

### Data synthesis and analysis

Review Manager software version 5.3 (RevMan; Copenhagen: The Nordic Cochrane Centre, the Cochrane Collaboration, 2014) was used to conduct the random-effects meta-analyses to determine the weighted mean differences (with 95% CIs) in change or final mean values for each measure. Forest plots were generated for each outcome. Cross-over studies were treated in the same way as parallel studies by comparing measurements from the intervention periods with the control periods. Although this approach results in a unit-of-analysis error, it is considered a conservative approach [40] and was therefore deemed appropriate.

The proportion of total variation attributable to between-study heterogeneity was estimated using the I^2^ test statistic [44] to provide an indication of the consistency of the results. An I^2^ value of ≥75% was deemed to indicate a high level of inconsistency, 50%–75% substantial inconsistency, 36%–60% moderate inconsistency, and 0%–35% low inconsistency; based on the recommendations by Higgins et al. [44] I^2^ values were generated for each analysis. The presence of small study effects (which may be the result of publication bias) was explored via funnel plots, which were conducted for all outcomes with ≥10 strata. Egger’s test was then conducted in Stata IC software version 15.1 (StataCorp LLC) to test for funnel plot asymmetry.

Prespecified subgroup analyses were conducted based on study design, nut group, and nut dose (≤30 g/d, 31–60 g/d, >60 g/d, based on ∼30 g typically recommended as 1 serving of nuts in dietary guidelines globally [3]), study duration (<12 wk compared with ≥12 wk, aligning with the approaches used in previous meta-analyses of nut consumption [[45], [46], [47]]) and participant health status to explore differences in the magnitude of effects between subgroups. For the studies that reported nut dose as a percentage of energy intake, we multiplied the reported percentage by the reported mean energy intake of the trial participants to recalculate the dosage in grams per day. Subgroup analyses were conducted where there were ≥10 effect sizes per outcome in total [40], although the number of effect sizes per individual subgroup was not restricted. As part of the sub-analyses based on nut dose, sensitivity analyses were also conducted to explore the effect of whole nuts by removing individual studies that assessed the effect of nut oils, nut powders, and/or nut flours from the meta-analysis.

### Quality assessment

All included studies were assessed using the Health Canada Quality Appraisal Tool [48]. Quality assessment was completed by 1 researcher (EPN), and the presence or absence of a clearly stated hypothesis, statistical power, study duration, and the background diet was taken into account when evaluating the quality of each study.

The certainty of the body of evidence for interventions included in the meta-analyses was determined using the grading of recommendations assessment, development, and evaluation (GRADE) [49]. GRADE evaluates the study design, risk of bias, inconsistency, indirectness, imprecision, and other considerations such as publication bias. GRADEpro GDT software [GRADEpro Guideline Development Tool (software). McMaster University and Evidence Prime, 2022. Available from gradepro.org] was used to conduct the certainty of evidence appraisal.

## Results

**FIGURE 1:**
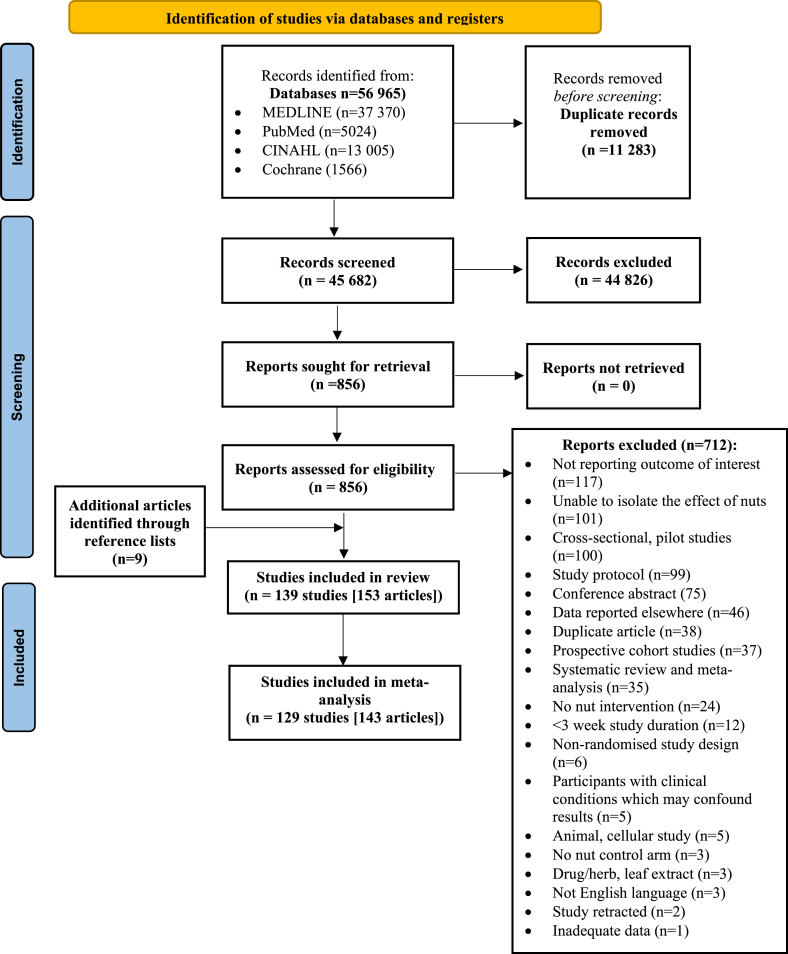
PRISMA [35] flow diagram of study selection. Abbreviations: CINAHL, Cumulated Index to Nursing and Allied Health Literature; MEDLINE, Medical Literature Analysis and Retrieval System Online.

A total of 56,965 articles were identified from the systematic search, including the review of relevant reference lists. After applying the exclusion criteria, 153 articles describing 139 studies were included in the systematic review and 129 studies in the meta-analysis. Ten studies (11 strata) [[50], [51], [52], [53], [54], [55], [56], [57], [58], [59], [60]] were excluded from the meta-analysis because of insufficient data reported; therefore, the results of the 11 strata are reported descriptively below for each study outcome. The process of study inclusion and exclusion is shown in Figure 1.

Characteristics of 139 included studies (81 parallel designs and 58 cross-over designs), including 9099 participants in the meta-analysis, are shown in Supplemental Table 3. Study duration ranged from 3–260 wk (or 5 y); only 8 [[61], [62], [63], [64], [65], [66], [67], [68], [69], [70], [71], [72]] out of 139 studies (6%) had a duration of >1 y.) Studies were conducted in 25 countries across 6 continents. The mean participant age was 48.3 y, and 116 studies (83%) included both males and females. Studies included participants who were healthy [51,53,56,58,[73], [74], [75], [76], [77], [78], [79], [80], [81], [82], [83], [84], [85], [86], [87], [88], [89], [90], [91], [92], [93], [94], [95], [96], [97], [98], [99], [100], [101], [102], [103], [104], [105]], had risk factors for chronic disease such as overweight or obesity [55,57,63,68,69,72,[106], [107], [108], [109], [110], [111], [112], [113], [114], [115], [116], [117], [118], [119], [120], [121], [122], [123], [124], [125], [126], [127], [128], [129], [130], [131], [132]], hypercholesterolemia/hyperlipidemia [59,60,71,[133], [134], [135], [136], [137], [138], [139], [140], [141], [142], [143], [144], [145], [146], [147], [148], [149], [150], [151], [152], [153], [154], [155], [156], [157], [158], [159], [160]], prediabetes [61,[161], [162], [163], [164]], at risk of CVD or had diagnosed CVD [52,[165], [166], [167]], at risk of metabolic syndrome or met the criteria for metabolic syndrome [64,70,[168], [169], [170], [171], [172], [173], [174], [175], [176]], had type 2 diabetes mellitus [54,62,[177], [178], [179], [180], [181], [182], [183], [184], [185], [186], [187], [188], [189], [190], [191], [192], [193]], had diagnosed coronary artery disease [[194], [195], [196], [197], [198]], or included a mixture of health conditions [50,[65], [66], [67],[199], [200], [201], [202]]. Included studies examined the effects of consumption of a range of tree nuts, including walnuts [52,56,59,60,62,63,[68], [69], [70],^75^,^76^,^81^,^83^,^84^,^86^,[96], [97], [98],^102^,^104^,^111^,^116^,^119^,^125^,^129^,^134^,^139^,^146^,^151^,^152^,[157], [158], [159],^162^,^167^,^170^,^176^,^184^,^186^,^187^,^192^,^202^], almonds [50,51,53,57,58,61,72,80,85,89,90,94,95,103,106,110,[112], [113], [114], 117,118,120,[131], [132], [133],^143^,^153^,^155^,^156^,^160^,^164^,^165^,^177^,[181], [182], [183],^191^,^195^,^198^], pistachios [54,87,121,126,138,141,142,144,145,149,154,161,169,174,[188], [189], [190]], hazelnuts [55,105,128,136,150,178,201], mixed nuts [[64], [65], [66], [67],^73^,^77^,^99^,^107^,^115^,^130^,^171^,^180^,^196^,^197^], cashews [74,91,179,185], macadamias [78,79,82,127,140,147], pecans [92,93,123,137,166,194], Brazil nuts [122,168,199,200], and peanuts [71,88,100,101,108,109,124,148,163,175,193] as well as comparing 2 different types of nuts [135,172,173] (Table 1). Nuts were consumed in either prescribed doses, ranging from ∼3 [59] to 88 [124] g/d for nut oils, flour, and butter and for whole nuts from 8 [116] to 168 [85] g/d or were included to provide a proportion of dietary energy, for example, 10% [178,179] to 30% [100,101] of total energy (equating to ∼21–76 g/d).

**TABLE 1:** Included studies and the type of nut analyzed (*n* = 139)

Type of nut	Number of studies *n*	Participants *n*	Female %	Mean age (y)	Mean duration (wk)	Mean nut dose (g/d)	Participant health status
Almond	36	2276	58	47	12	58	1 CVD or at CVD risk, 1 multiple health conditions, 2 CAD, 2 prediabetic, 4 T2DM, 6 hypercholesterolemia/hyperlipidemia, 8 healthy, and 12 overweight/obese
Brazil nut	4	216	69	49	11	14	1 MetS or at risk of MetS, 1 overweight/obese, and 2 multiple health conditions
Cashew nut	4	394	59	54	7	37	2 healthy and 2 T2DM
hazelnut	7	393	56	41	8	37	1 healthy, 1 multiple health conditions, 1 T2DM, 2 hypercholesterolemia/hyperlipidemia, and 2 overweight/obese
Macadamia	6	237	57	40	5	49	1 overweight/obese, 2 hypercholesterolemia/hyperlipidemia, and 3 healthy
Peanut	10	895	46	45	14	58	1 prediabetic, 1 MetS or at risk of MetS, 1 T2DM, 2 healthy, 2 hypercholesterolemia/hyperlipidemia, and 3 overweight/obesity
Pecan	6	272	51	50	8	58	1 CAD, 1 CVD or at CVD risk, 1 hypercholesterolemia/hyperlipidemia, 1 overweight/obese, and 2 healthy
Pistachio	14	652	56	49	12	62	1 healthy, 1 prediabetic, 2 MetS or at risk of MetS, 2 overweight/obese, 3 T2DM, and 5 hypercholesterolemia/hyperlipidemia
Walnut	39	2824	52	52	16	38	1 prediabetic, 1 multiple health conditions, 2 CVD or CVD risk, 3 MetS or at risk of MetS, 5 T2DM, 7 overweight/obese, 9 hypercholesterolemia/hyperlipidemia, and 11 healthy
Mixed nuts	11	858	49	47	35	45	1 CAD, 1 multiple health conditions, 1 T2DM, 2 MetS, 3 healthy, and 3 overweight/obese
Different study arms compared different nuts (for example, group 1 consumed almonds, and group 2 consumed walnuts)	2	82	52.5	51	6	69	1 hypercholesterolemia/hyperlipidemia and 1 MetS or at risk of MetS
Total (all nuts)	139	9099	54	48	14	48	

Abbreviations: CAD, coronary artery disease; CVD, cardiovascular disease; MetS, metabolic syndrome; T2DM, type 2 diabetes mellitus.

### Effect of nut consumption on study outcomes

#### LDL cholesterol

**FIGURE 2:**
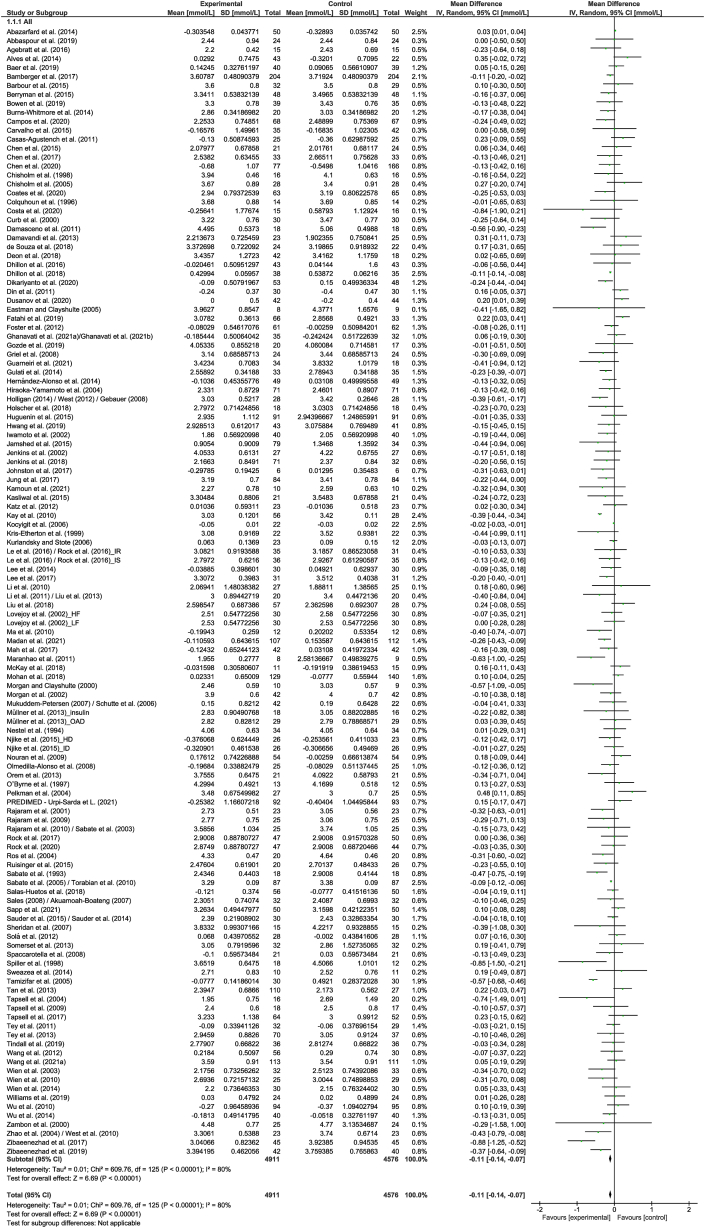
Difference in LDL cholesterol (mmol/L) between nut consumption and control. The diamond indicates a weighted mean difference with 95% CIs. Abbreviation: IV, inverse variance.

A total of 126 strata from 122 studies explored the effect of nut consumption on LDL cholesterol. The meta-analysis showed that nut consumption was associated with a significant decrease in LDL cholesterol (Table 2 and Figure 2). Of the 126 strata, 35 explored the effect of walnut consumption, and 101 had a duration of ≤3 mo. Eleven strata from 10 RCTs [50,[52], [53], [54], [55], [56], [57], [58], [59], [60]] were removed from the meta-analysis, of which 2 [56,60] reported a significant reduction in LDL cholesterol with the consumption of nuts. Subgroup analysis indicated significant differences, including those related to study design, nut type, and health status (Supplemental Table 4). Larger reductions in LDL cholesterol were found in cross-over studies, compared to studies with a parallel design, although the magnitude of effects in the different subgroups is not likely to be clinically significant. Regarding nut type and health status, it should be noted that the significance of the effects should be interpreted with caution because of the uneven distribution of studies in the subgroups. When studies assessing the effect of nut oils, nut powders, and/or nut flours were removed from the dose subgroup analysis, similar results were found in the overall analysis (Supplemental Table 5).

**TABLE 2:** Change in outcomes following nut intervention compared to a control

Outcome	Number of strata	Number of studies	Number of participants	Effect estimate	Inconsistency (I^2^)%
LDL cholesterol, mmol/L	126	122	9487	−0.11 (−0.14, −0.07), *P* < 0.00001	80
HDL cholesterol, mmol/L	126	122	10,102	0.00 (−0.01, 0.02), *P* = 0.59	81
TC, mmol/L	126	122	9423	−0.13 (−0.18, −0.09), *P* < 0.00001	87
TG, mmol/L	122	118	9744	−0.06 (−0.08, −0.03), *P* < 0.00001	61
TG:HDL cholesterol	5	5	331	−0.12 (−0.40, 0.15), *P* = 0.37	0
TC:HDL cholesterol	51	49	3837	−0.15 (−0.24, −0.07), *P* = 0.0003	68
HDL cholesterol:LDL cholesterol	3	3	209	−0.06 (−0.23, 0.11), *P* = 0.46	53
LDL cholesterol:HDL cholesterol	39	38	2539	−0.14 (−0.20, 0.08), *P* < 0.00001	75
ApoB, mg/dL	39	39	3069	−3.01 (−4.44, −1.58), *P* < 0.00001	44
ApoA-I, mg/dL	33	33	2259	−1.02 (−2.53, 0.49), *P* = 0.19	0
SBP, mmHg	71	70	6521	−0.05 (−0.83, 0.72), *P* = 0.89	47
DBP, mmHg	67	66	6198	−0.10 (−0.53, 0.33), *P* = 0.65	24

Abbreviations: ApoA-I, apolipprotein A-1; ApoB, apolipoprotein B; DBP, diastolic blood pressure; HDL, high density lipoprotein; LDL, low density lipoprotein; SBP, systolic blood pressure; TC, total cholesterol; TG, triglycerides.

#### TC

**FIGURE 3:**
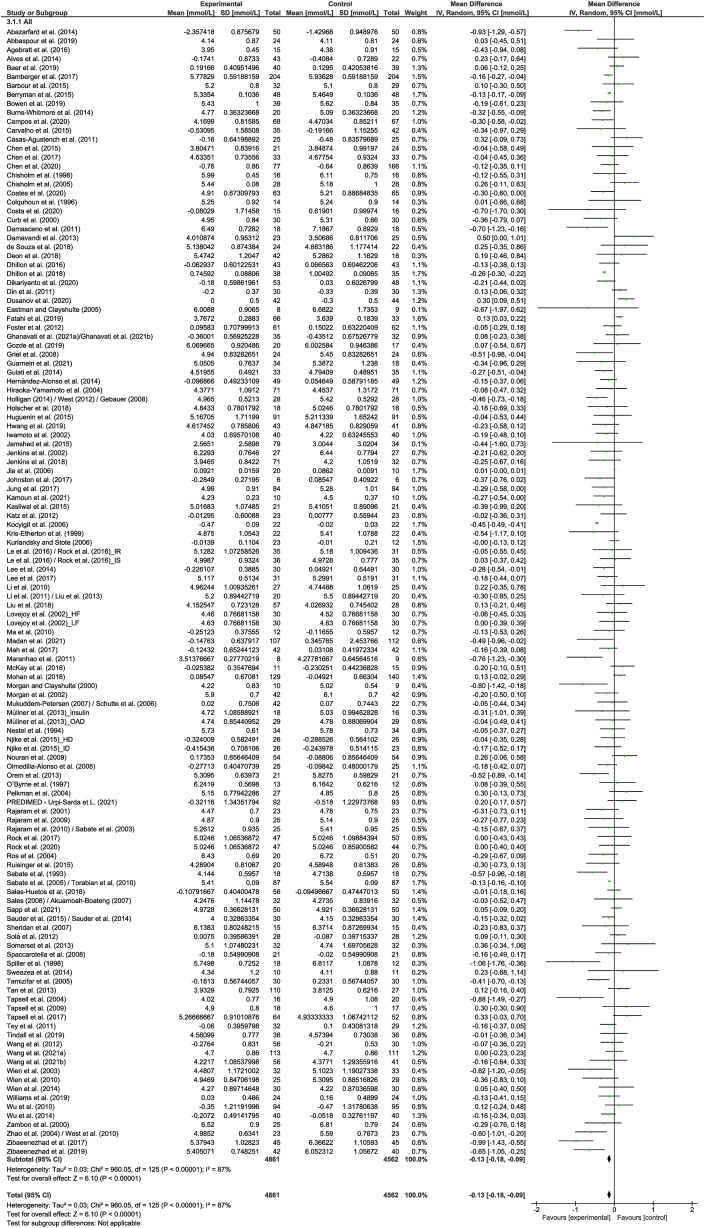
Difference in total cholesterol (mmol/L) between nut consumption and control. The diamond indicates a weighted mean difference with 95% CIs. Abbreviation: IV, inverse variance.

A total of 126 strata from 122 studies explored the effect of nut consumption on TC. The meta-analysis showed that nut consumption was associated with a significant decrease in TC (Table 2 and Figure 3). Walnuts (*n* = 35) and almonds (*n* = 33) were the most common nut types in this analysis. Eleven strata from 10 RCTs [50,[52], [53], [54], [55], [56], [57], [58], [59], [60]] were removed from the meta-analysis, of which 3 [54,56,60] reported a significant reduction in TC with the consumption of nuts. Variation in the magnitude of the effect was observed for nut type, although these results should be interpreted with caution because of the uneven distribution of studies in the subgroups (Supplemental Table 6). For the nut dose, a significant dose response was found. Stronger effects were observed for >60 g/d; however, there was substantial heterogeneity that should be considered when interpreting results. Findings were similar when studies assessing the effect of nut oils, nut powders, and/or nut flours were removed (Supplemental Table 5).

#### TG

**FIGURE 4:**
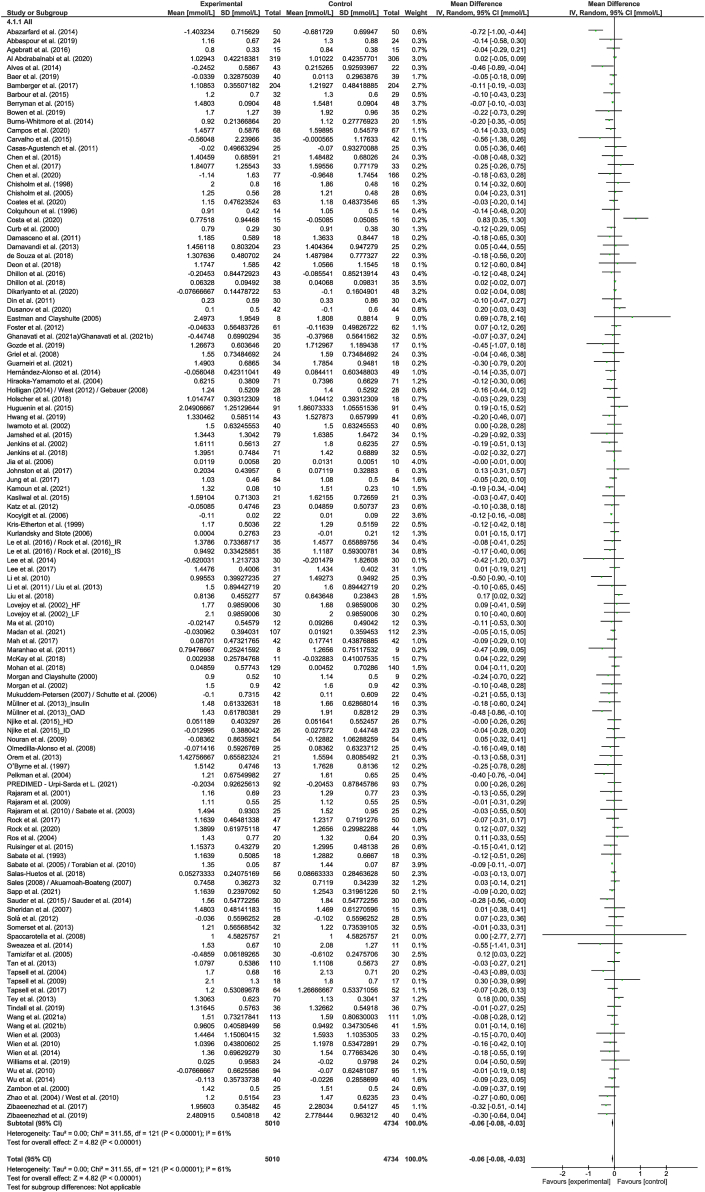
Difference in TGs (mmol/L) between nut consumption and control. The diamond indicates a weighted mean difference with 95% CIs. Abbreviation: IV, inverse variance.

A total of 122 strata from 118 studies explored the effect of nut consumption on TG. The meta-analysis showed that nut consumption was associated with a significant decrease in TG (Table 2 and Figure 4). Eleven strata from 10 RCTs [50,[52], [53], [54], [55], [56], [57], [58], [59], [60]] were removed from the meta-analysis, of which 3 [56,59,60] reported a significant reduction in TG with the consumption of nuts. Subgroup analysis indicated differences related to nut type (Supplemental Table 7). However, this significant effect should be interpreted with caution because of the uneven distribution of studies included in the subgroups. When studies assessing the effect of nut oils, nut powders, and/or nut flours were removed from the dose subgroup analysis, results were similar to the overall analysis (Supplemental Table 5).

#### TC to HDL cholesterol ratio

**FIGURE 5:**
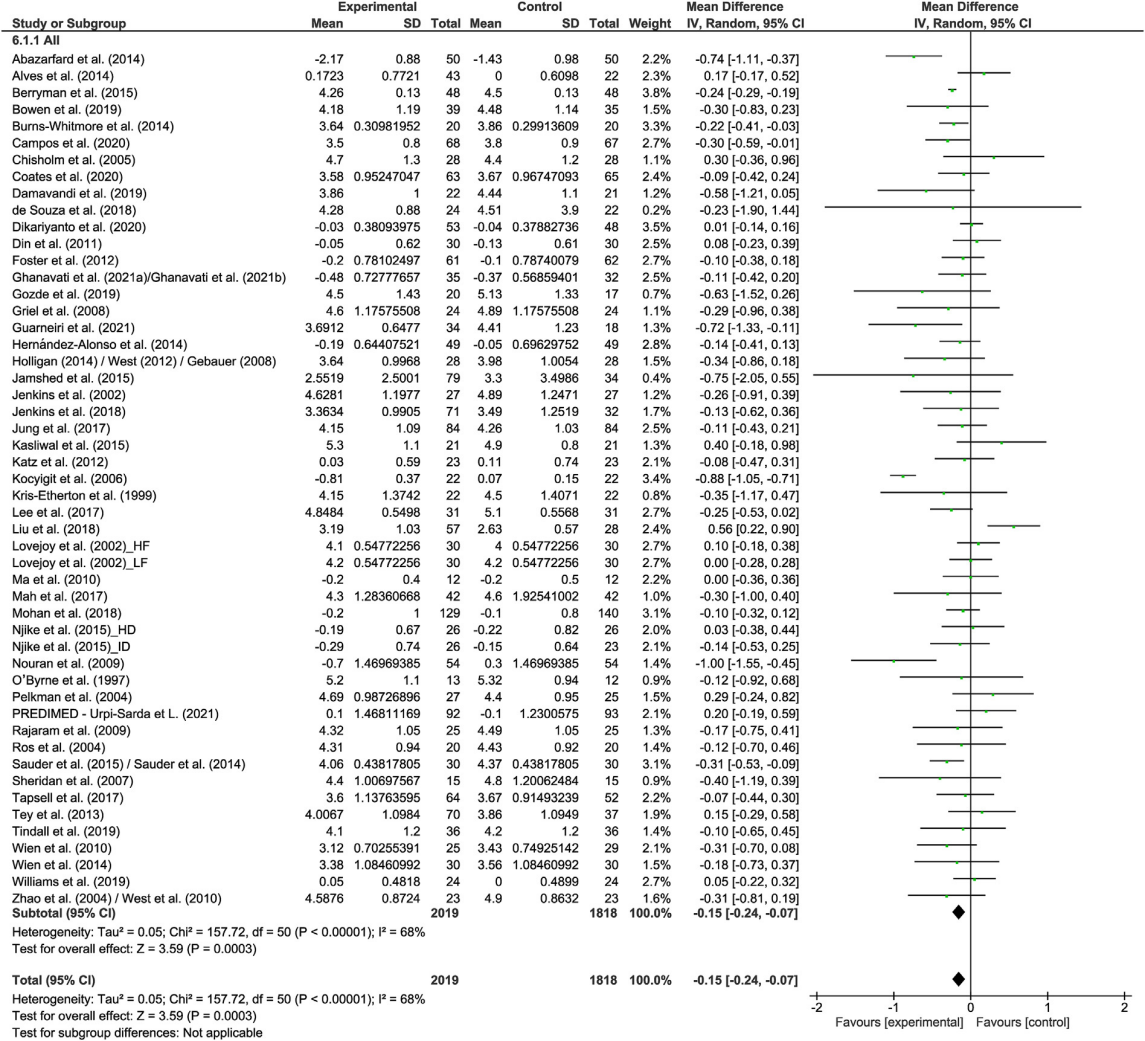
Difference in total cholesterol to HDL cholesterol ratio between nut consumption and control. The diamond indicates a weighted mean difference with 95% CIs. Abbreviation: IV, inverse variance.

A total of 51 strata from 49 studies explored the effect of nut consumption on TC:HDL cholesterol. The meta-analysis showed that nut consumption was associated with a significant decrease in TC:HDL cholesterol (Table 2 and Figure 5). Six strata from 6 RCTs [53,54,56,57,60,105] were removed from the meta-analysis, of which 3 [53,54,60] reported a significant reduction in TC:HDL cholesterol with the consumption of nuts. The subgroup analyses indicated that a similar magnitude of the effect was found across the different subgroups (Supplemental Table 8). After removing studies assessing the effect of nut oils, nut powders, and/or nut flours from the dose subgroup analysis, results were similar to the overall analysis (Supplemental Table 5).

#### LDL cholesterol to HDL cholesterol ratio

**FIGURE 6:**
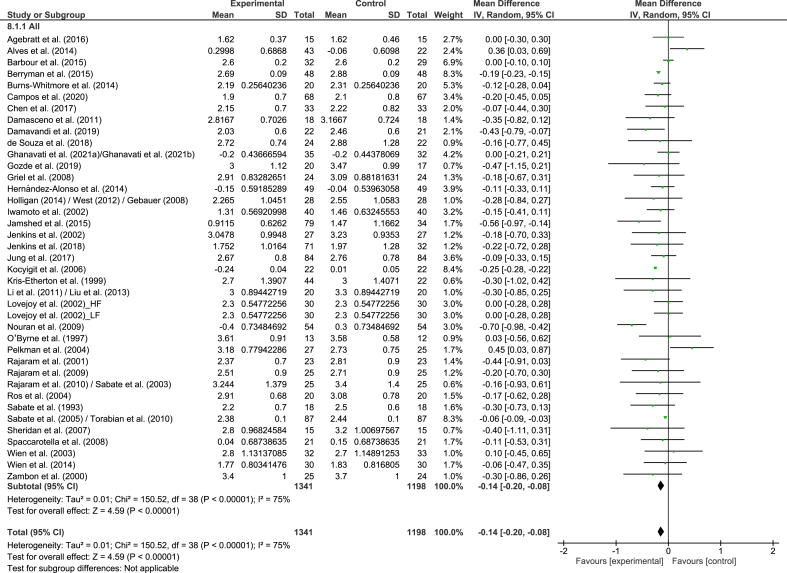
Difference in LDL cholesterol to HDL cholesterol ratio between nut consumption and control. The diamond indicates a weighted mean difference with 95% CIs. Abbreviation: IV, inverse variance.

A total of 39 strata from 38 studies explored the effect of nut consumption on LDL cholesterol:HDL cholesterol. The meta-analysis showed that nut consumption was associated with a significant decrease in LDL cholesterol:HDL cholesterol (Table 2 and Figure 6). Two strata from 2 RCTs were removed from the meta-analysis, of which both [53,54] reported a significant reduction in LDL cholesterol:HDL cholesterol with the consumption of nuts. Variation in the magnitude of the effect was observed for nut type, study duration, and health status (Supplemental Table 9). Although studies with a shorter duration (<12 wk) had a larger decrease in LDL cholesterol:HDL cholesterol than found for studies of ≥12 wk, the magnitude of the effects in the subgroups is not likely to be clinically significant. For nut type and health status, these results should be interpreted with caution because of the small and/or uneven distribution of studies included in the subgroups. When studies assessing the effect of nut oils, nut powders, and/or nut flours were removed from the dose subgroup analysis, similar results were found in the overall analysis (Supplemental Table 5).

#### ApoB

**FIGURE 7:**
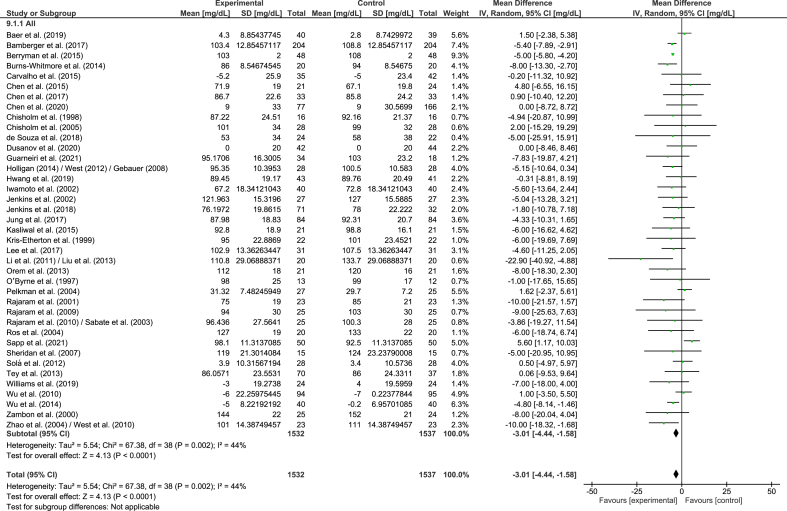
Difference in apoB (mg/dL) between nut consumption and control. The diamond indicates a weighted mean difference with 95% CIs. Abbreviation: IV, inverse variance.

A total of 39 strata from 39 studies explored the effect of nut consumption on apoB. The meta-analysis showed that nut consumption was associated with a significant decrease in apoB (Table 2 and Figure 7). One stratum from 1 RCT [57] was removed from the meta-analysis, though no significant finding in relation to apoB and the consumption of nuts was reported. Subgroup analysis indicated significant subgroup differences related to study design, nut type, and health status (Supplemental Table 10). Larger decreases in apoB were observed in cross-over studies, compared to parallel studies, although the magnitude of the effects in the different subgroups is not likely to be clinically significant. Other significant effects for nut type and health status should be interpreted with caution because of the small and/or uneven distribution of studies included in the subgroups. When studies assessing the effect of nut oils, nut powders, and/or nut flours were removed from the dose subgroup analysis, similar results were found in the overall analysis (Supplemental Table 5).

#### HDL cholesterol, TG to HDL cholesterol ratio, HDL cholesterol to LDL cholesterol ratio, apoA-I, SBP, and DBP

The meta-analysis showed that consumption of nuts had no significant effect on HDL cholesterol and did not result in significant differences in TG:HDL cholesterol, HDL cholesterol:LDL cholesterol, apoA-I, SBP, and DBP (Table 2 and Supplemental Figures 1–6). In the case of the subgroup analyses, a similar magnitude of the effect was found across the different subgroups for apoA-I (Supplemental Table 11) and SBP (Supplemental Table 12). Although there were significant subgroup differences found for HDL cholesterol (Supplemental Table 13) and DBP (Supplemental Table 14) according to health status, these differences should be interpreted with caution because of the uneven distribution in the number of participants and studies included in the subgroups. Variation in the magnitude of the effect was also observed for DBP and study duration (Supplemental Table 14). Although a reduction in DBP was only observed in studies with a duration of 12 or more weeks, it should be noted that the magnitude of this reduction was small and not likely to be clinically significant. Similar results to the original analysis were found when studies assessing the effect of nut oils, nut powders, and/or nut flours were removed from the dose subgroup analysis for HDL cholesterol, TG:HDL cholesterol, HDL cholesterol:LDL cholesterol, apoA-I, SBP, and DBP (Supplemental Table 5).

#### Heterogeneity

Considerable heterogeneity (I^2^ >75%) was observed for LDL cholesterol, HDL cholesterol, and TC (see Table 2). In addition, substantial heterogeneity (I^2^: 50%–75%) was observed for 4 measures (TG, TC:HDL cholesterol, LDL cholesterol:HDL cholesterol, and HDL cholesterol:LDL cholesterol), moderate heterogeneity (I^2^: 36%–60%) for 2 measures (apoB and SBP), whereas low heterogeneity (I^2^: 0%–35%) was observed for 3 measures (TG:HDL cholesterol, apoA-I, and DBP).

#### Small study effects

Funnel plots were generated for outcomes with 10 or more strata (LDL cholesterol, HDL cholesterol, TC, TG, TC:HDL cholesterol, LDL cholesterol:HDL cholesterol, apoA-I, apoB, SBP, and DBP) (see Supplemental Figures 7–16). Egger’s test outcomes indicated funnel plot asymmetry for LDL cholesterol (bias: −0.69; 95% CI: −1.10, −0.28; *P* = 0.001), HDL cholesterol (bias: 0.58; 95% CI: 0.07, 1.09; *P* = 0.027), TC (bias: −0.82; 95% CI: −1.33, −0.31; *P* = 0.002), TG (bias: −0.58; 95% CI: −0.86, −0.30; *P* = 0.000), SBP (bias: −0.61; 95% CI: −1.11, −0.12; *P* = 0.016), and DBP (bias: −0.57; 95% CI: −0.99, −0.14; *P* = 0.010). These results indicate the presence of small study effects, which may be caused by publication bias. By comparison, funnel plot asymmetry was not detected for TC:HDL cholesterol (bias: 0.42; 95% CI: −0.33, 1.16; *P* = 0.266), LDL cholesterol:HDL cholesterol (bias: 0.14; 95% CI: −0.63, 0.91; *P* = 0.713), apoB (bias: 0.35; 95% CI: −0.20, 0.90; *P* = 0.205), and apoA-I (bias: 0.42; 95% CI: −0.34, 1.19; *P* = 0.268).

#### Quality assessment

Quality assessment is presented for each study in Supplemental Table 3, with full quality assessment available in Supplemental Table 15. The quality of evidence was “low” for *n* = 18 intervention studies [50,51,53,55,[58], [59], [60],^77^,^78^,^82^,^85^,^90^,^100^,^101^,^122^,^124^,^127^,^146^,^149^,^150^].

The certainty of the body of evidence was assessed for studies included in meta-analyses using GRADE, with results presented in Supplemental Table 16. The certainty of the body of evidence for apoA-I was “high.” The body of evidence for TC:HDL cholesterol, LDL cholesterol:HDL cholesterol, and apoB was downgraded to “moderate” because of inconsistency and for DBP because of the likelihood of publication bias. The certainty for TG and SBP was downgraded to “low” because of inconsistency and the likelihood of publication bias, and the certainty for TG:HDL cholesterol was downgraded to “low” because of imprecision and the likelihood of publication bias. Certainty for LDL cholesterol, HDL cholesterol, TC, was downgraded to “very low” because of the inconsistency and likelihood of publication bias, and HDL cholesterol:LDL cholesterol because of inconsistency, imprecision, and the likelihood of publication bias.

## Discussion

This systematic review and meta-analysis showed favorable effects of tree nut and peanut consumption on lowering LDL cholesterol, TC, TG, TC:HDL cholesterol, LDL cholesterol:HDL cholesterol, and apoB from RCTs including 9099 participants. These findings align with a review conducted in 2015, which explored the effects of all tree nuts on CVD risk factors [22]. The present meta-analysis builds on these findings with the addition of studies published since the 2015 review [22] and focuses solely on RCTs. It also includes studies investigating other nut forms, including nut oils [55,59,71,88,100,101,124,147,[158], [159], [160],^168^,^186^,^187^,^202^,^203^], nut butter [88,124], and nut flour [199] and adolescents (aged 13 y or older) [136]. Subgroup analyses found differences across the types of nuts for outcomes, LDL cholesterol, TC, TG, LDL cholesterol:HDL cholesterol, and apoB. Having fewer studies within the Brazil nut, cashew nut, hazelnut, macadamia, and pecan subgroups may have played a role in the significant effects observed. A dose response and stronger effects were also observed for >60 g/d for TC. Despite the nut dose response, a reduction in LDL cholesterol, TC, TG, TC:HDL cholesterol, LDL cholesterol:HDL cholesterol, and apoB, and an increase in HDL were found in ≤30 g/d of nuts. Taken together, this meta-analysis provides a timely effect of tree nut and peanut intake on overall CVD risk reduction, including dose-response relations.

The scientific evidence indicates that the consumption of nuts is beneficial to positive changes in many biomarkers of CVD. Although biomarkers were assessed in this review, the population effect for LDL cholesterol, HDL cholesterol, TC, and TG was based on >4500 interventions and >4500 control participants, and SBP and DBP were based on >3000 interventions and >3000 control participants. This substantial population size for high-quality study designs indicates significant reductions in LDL cholesterol, TC, and TG and no change in HDL cholesterol. Despite the heterogeneity, this combined effect suggests that nuts do not simply display positive changes to 1 biomarker of CVD risk but a range of biomarkers to create an overall risk reduction. Although the findings of the SBP and DBP studies indicated a nonsignificant reduction, there was less heterogeneity which is of clinical relevance. The effects on HDL cholesterol were also not indicative of risk reduction, though the meta-analysis suggested no change, which is also a favorable outcome. Both blood lipids and blood pressure are major risk factors for CVD and are included in the Framingham risk equation [204], used globally by clinicians. Therefore, this review provides evidence of a causal link between nut intake and lower CVD risk.

In this review, we observed that there were significant differences in outcomes (LDL cholesterol, TC, TG, LDL cholesterol:HDL cholesterol, and apoB) across different types of nuts. Consideration is needed when interpreting the findings of this review as it is unclear whether all or some types of nuts are preferential for improving CVD risk. This review suggests stronger evidence for almonds and walnuts because of the greater number of studies (*n* > 30), and therefore more studies are needed for other nut types.

Our findings suggest a dose response was observed for tree nut and peanut consumption of >60 g/d for TC. Similar findings were reported in a recent meta-analysis of RCTs and non-RCTs in this field [22], which showed the relationship between tree nut intake and TC and LDL cholesterol was nonlinear, with stronger effects at >60 g/d. However, the scientific evidence suggests that consumers typically do not meet recommended serving sizes of 15–30 g. In Australia, for example, nut intake was estimated at an average of 4.61 g/d, with only 5.6% of the population consuming the recommended daily amount of nuts [205]. Public health messaging needs to support reasons for consumers to increase their intake of nuts. However, nuts, as a food group because of their high-fat content, have been related to consumer concerns about weight gain, a risk factor for CVD. While this review did not look at body weight, a recent meta-analysis of 106 RCTs and 6 prospective cohorts showed that higher nut intake was associated with reductions in body weight and body fat [206]. Establishing health messages about the regular consumption of nuts to reduce CVD risk may shift consumer opinion and add to the acknowledgment of core foods for positive lifestyle change related to CVD risk.

Consideration should be given when interpreting findings from this review, which included only RCTs when compared to real-life consumer settings, as they are substantially different. RCTs aiming to explore the influence of specific foods must consider many methodological challenges, including the design of the dietary intervention and control arms to avoid increases in total energy intake, which could skew results [207]. Although a dose response was reported for TC, several studies incorporated nuts as a proportion of a participant’s total energy, resulting in substantial variation between individual nut doses. Whether the energy value of nuts was adjusted for in the total diet may also influence the results. We did not consider in our subgroup analysis whether the energy provided by nuts was accounted for by dietary modeling or advice to substitute other foods or nuts, though there is evidence suggesting that energy-restricted diets are effective in improving the blood lipid profile [208] and blood pressure [209]. This highlights the importance of total energy intake and should be considered in future meta-analyses. Further, energy-restricted diet studies that were designed for weight loss were included in this analysis. As weight loss is a significant contributor to improving CVD risk factors, it is challenging to determine whether the effects observed from this review were from nuts alone or if further benefits were obtained from the additional weight loss. The design of the control arm may have also impacted our results; for example, Agebratt et al. [73] compared nut intake with a control intervention (that is, fruit), which may have potentially influenced the control effects. A previous meta-analysis has highlighted the potential impact of control groups on underestimating intervention effects in weight-loss RCTs [210]. Thus, RCTs that aim to explore the influence of specific foods should carefully design the dietary intervention and control arms to avoid a total energy increase to skew results. Despite these challenges, the most recent meta-analyses of prospective cohort studies in this field [21] have shown an inverse association between nut consumption and CVD incidence and mortality. Taken together, this review provides the most recent evidence that the findings from RCTs conducted in highly controlled conditions support the findings from observational studies that reflect free-living populations.

This review has several strengths and limitations. A notable strength was that the processes followed current guidelines in review conduct, reporting, and analysis. The review is grounded in the evidence with findings aligned to previous reviews of observational studies and 1 review of randomized and nonrandomized trials. We considered a range of outcomes associated with CVD, including blood lipids, apolpoproteins,and blood pressure. In a recent review by the authors, the effects of nuts and inflammation (c-reactive protein, adiponectin, tumor necrosis factor alpha, interleukin-6, intercellular adhesion molecule 1, and vascular cell adhesion molecule 1) reported nonsignificant changes because of a lack of consistent available evidence, and as a result, such variables were not included in this review [211]. However, we recognize that the outcomes explored in this review are not interchangeable with disease endpoints such as CVD mortality and morbidity. The range of population groups addressed in the included studies of this review may be considered both a strength and a limitation. The populations included some groups who had existing risk factors for CVD, such as overweight and type 2 diabetes mellitus, whereas others were considered “healthy” populations. Because of the existing comorbidities, the effect size of some studies may appear greater than if all studies targeted a healthy population. For cross-over trials, there were no additional exclusion criteria applied, which meant studies using this design were not required to meet a “sufficient” wash-out period for inclusion in this review. This may have resulted in potential carryover effects, though the potential impact on the results was considered in the quality appraisal. These above-mentioned points need to be considered when interpreting the findings.

The heterogeneity of the evidence included can also be considered a limitation. Variation existed because of study design and duration, participant health status, nut type, and dose, although these factors were explored in subgroup analyses. In particular, relatively few studies in some nut types (Brazil nut, cashew nut, hazelnut, macadamia, and pecan) limit the statistical power to detect a potential interaction that needs to be considered in relation to the whole body of evidence. Of the different tree nut types, all tree nuts except chestnuts were addressed in the body of evidence that was reviewed. The lower fat profile of chestnuts suggests that they could mechanistically work differently from other nut types [212]. In addition, pine nuts were only included in 1 study as part of mixed nuts consumed by participants [171]. Thought should be given to the variability of nuts as a food group, as there are a number of methodological challenges relating to the study of dietary patterns and CVD. One major challenge is related to the way food groups are formed and the limitations of the different food composition databases used to analyze the outcomes, especially if energy is adjusted [213]. Background diets also varied between studies, with some prescribing dietary guidelines, whereas others advised participants to follow their habitual diet, which may have varied considerably between individuals. For 1 study, the PREDIMED study [[65], [66], [67]], we considered the olive oil arm to be the control arm when compared with the other Mediterranean diet arms. The authors acknowledge that olive oil provides unsaturated fats and has been associated with lipid-lowering properties. This comparison, however, eliminates the confounding nature of the overall Mediterranean diet, allowing for the comparison between nut and olive oil consumption. Similarly, 1 study [176] also included seeds in their control diet. Although linseeds have a similar nutrient profile to nuts, a meta-analysis reported particular doses and participant characteristics to influence the lipid-lowering effect [214]. Analysis of funnel plots suggested the results for LDL cholesterol, HDL cholesterol, TC, TG, SBP, and DBP may have been influenced by small study effects, which may be caused by publication bias. This resulted in downgrading the quality of evidence for these outcomes. Finally, although we were unable to explore the distribution of the published data included in this meta-analysis, several studies reported median values rather than means, which may suggest that some of the data was skewed and may have impacted our analyses.

In conclusion, this systematic review and meta-analysis of the effects of tree nut and peanut consumption on improving risk of CVD finds evidence of significantly favorable effects on LDL cholesterol, TC, TG, TC:HDL cholesterol, LDL cholesterol:HDL cholesterol, and apoB. The nonsignificant differences in SBP and DBP suggest a lack of consistent evidence for the effects of tree nuts and peanuts on blood pressure. The results should also be interpreted with caution because of the large variation in the included studies. The findings of this review provide further insight into the combined effect that tree nuts and peanuts do not simply display positive changes to 1 biomarker of CVD risk but a range of biomarkers to create an overall risk reduction. The findings of this review also build on previous observational and intervention meta-analyses, which have shown an inverse association between nut consumption and risk of CVD. Although stronger effects are observed for tree nut and peanut consumption of >60 g/d for LDL cholesterol and TC, most dietary guidelines recommend the consumption of 15–30 g as a serving size. Importantly, this review shows a reduction in LDL cholesterol, TC, TG, TC:HDL cholesterol, LDL cholesterol:HDL cholesterol, and apoB, and an increase in HDL with the consumption of ≤30 g/d of nuts, supporting dietary guidelines. As consumers are not currently meeting these recommendations, public health messaging is needed to support reasons for consumers to increase their intake of nuts, which also have favorable effects on risk of CVD.

## Acknowledgments

We thank Dr. Vivienne Guan for her contribution to the scoping review, which set the foundations for this systematic review. We also thank Ms. Elizabeth Sherry for her help with quality checking (source data verification).

The authors’ responsibilities were as follows—LH: designed and conducted the research, performed the literature search, wrote the initial draft, and had primary responsibility for final content; LH, EPN, and YCP: performed data screening; LH and MCS: performed data extraction; EPN: performed quality assessment; and all authors: read and approved the final manuscript.

## Data availability

The data used in this review are available from the corresponding author upon reasonable request.

## Funding

This research was funded by the Australian Nut Industry Council Ltd, which did not provide any input into the design, collection, analysis, interpretation of the data, or manuscript writing.

## Author disclosures

EPN has previously received funding from the International Nut and Dried Fruit Council. In addition, EPN and YCP have previously received funding from the California Walnut Commission. LH and MCS, no conflicts of interest.
