# Supplementary material for: Tree Nut and Peanut Consumption and Risk of Cardiovascular Disease: A Systematic Review and Meta-Analysis of Randomized Controlled Trials
Source: Adv Nutr. 2023 May 5;14(5):1029–49. doi: 10.1016/j.advnut.2023.05.004 (PMC10509427; doi:10.1016/j.advnut.2023.05.004)
Supplement: Multimedia component 1 [file mmc1.docx]

**Tree nut and peanut consumption and risk of cardiovascular disease: A systematic review and meta-analysis of randomised controlled trials**

Lauren Houston^*^, Yasmine C Probst, Mamatha Chandra Singh and Elizabeth P Neale

Corresponding author: Lauren Houston (Email: [lhouston@georgeinstitute.org.au](mailto:lhouston@georgeinstitute.org.au),Telephone: +61 2 8052 4876, Mailing address: Level 5, 1 King Street Newtown 2042. NSW, Australia)

**Online Supplementary Material**

Contents

[**Supplementary Table 1:** PRISMA Checklist 4](#_Toc137677527)

[**Supplementary Table 2:** Combined search strategy employed to address the research question. 7](#_Toc137677528)

[**Supplementary Table 3:** Characteristics of included randomised controlled trials 9](#_Toc137677529)

[**Supplementary Table 4:** Results of sub-group analyses for LDL cholesterol 104](#_Toc137677530)

[**Supplementary Table 5:** Results of sensitivity analyses for studies exploring the effects of whole nuts 105](#_Toc137677531)

[**Supplementary Table 6:** Results of sub-group analyses for total cholesterol 106](#_Toc137677532)

[**Supplementary Table 7:** Results of sub-group analyses for triglycerides 107](#_Toc137677533)

[**Supplementary Table 8:** Results of sub-group analyses for total cholesterol to HDL cholesterol ratio 108](#_Toc137677534)

[**Supplementary Table 9:** Results of sub-group analyses for LDL cholesterol to HDL cholesterol ratio 109](#_Toc137677535)

[**Supplementary Table 10:** Results of sub-group analyses for apolipoprotein B 110](#_Toc137677536)

[**Supplementary Table 11:** Results of sub-group analyses for apolipoprotein A-I 111](#_Toc137677537)

[**Supplementary Table 12:** Results of sub-group analyses for systolic blood pressure 112](#_Toc137677538)

[**Supplementary Table 13:** Results of sub-group analyses for HDL cholesterol 113](#_Toc137677539)

[**Supplementary Table 14:** Results of sub-group analyses for diastolic blood pressure 114](#_Toc137677540)

[**Supplementary Table 15:** Health Canada quality appraisal of included studies 115](#_Toc137677541)

[**Supplementary Table 16:** GRADE evidence profile 254](#_Toc137677542)

[**Supplementary Figure 1:** Difference in HDL cholesterol (mmol/L) between nut consumption and control. Diamond indicates weighted mean difference with 95% CIs. 257](#_Toc137677543)

[**Supplementary Figure 2:** Difference in triglycerides to HDL cholesterol ratio between nut consumption and control. Diamond indicates weighted mean difference with 95% CIs. 258](#_Toc137677544)

[**Supplementary Figure 3:** Difference in HDL cholesterol to LDL cholesterol ratio between nut consumption and control. Diamond indicates weighted mean difference with 95% CIs. 258](#_Toc137677545)

[**Supplementary Figure 4:** Difference in apoA-I (mg/dL) between nut consumption and control. Diamond indicates weighted mean difference with 95% CIs. 259](#_Toc137677546)

[**Supplementary Figure 5:** Difference in SBP (mmHg) between nut consumption and control. Diamond indicates weighted mean difference with 95% CIs. 260](#_Toc137677547)

[**Supplementary Figure 6:** Difference in DBP (mmHg) between nut consumption and control. Diamond indicates weighted mean difference with 95% CIs. 261](#_Toc137677548)

[**Supplementary Figure 7:** LDL cholesterol funnel plot 262](#_Toc137677549)

[**Supplementary Figure 8:** HDL cholesterol funnel plot 263](#_Toc137677550)

[**Supplementary Figure 9:** TC funnel plot 264](#_Toc137677551)

[**Supplementary Figure 10:** TG funnel plot 265](#_Toc137677552)

[**Supplementary Figure 11:** TC:HDL cholesterol funnel plot 266](#_Toc137677553)

[**Supplementary Figure 12:** LDL cholesterol:HDL cholesterol funnel plot 267](#_Toc137677554)

[**Supplementary Figure 13:** ApoB funnel plot 268](#_Toc137677555)

[**Supplementary Figure 14:** ApoA-I funnel plot 269](#_Toc137677556)

[**Supplementary Figure 15:** SBP funnel plot 270](#_Toc137677557)

[**Supplementary Figure 16:** DBP funnel plot 271](#_Toc137677558)

# **Supplementary Table 1:** PRISMA Checklist

| **Section and Topic** | **Item #** | **Checklist item** | **Location where item is reported (page number)** |
| --- | --- | --- | --- |
| **TITLE** | | |  |
| Title | 1 | Identify the report as a systematic review. | 1 |
| **ABSTRACT** | | |  |
| Abstract | 2 | See the PRISMA 2020 for Abstracts checklist. | 3-4 |
| **INTRODUCTION** | | |  |
| Rationale | 3 | Describe the rationale for the review in the context of existing knowledge. | 5-6 |
| Objectives | 4 | Provide an explicit statement of the objective(s) or question(s) the review addresses. | 7 |
| **METHODS** | | |  |
| Eligibility criteria | 5 | Specify the inclusion and exclusion criteria for the review and how studies were grouped for the syntheses. | 8-9 |
| Information sources | 6 | Specify all databases, registers, websites, organisations, reference lists and other sources searched or consulted to identify studies. Specify the date when each source was last searched or consulted. | 7 |
| Search strategy | 7 | Present the full search strategies for all databases, registers and websites, including any filters and limits used. | Supplementary file 2 |
| Selection process | 8 | Specify the methods used to decide whether a study met the inclusion criteria of the review, including how many reviewers screened each record and each report retrieved, whether they worked independently, and if applicable, details of automation tools used in the process. | 7-8 |
| Data collection process | 9 | Specify the methods used to collect data from reports, including how many reviewers collected data from each report, whether they worked independently, any processes for obtaining or confirming data from study investigators, and if applicable, details of automation tools used in the process. | 9 |
| Data items | 10a | List and define all outcomes for which data were sought. Specify whether all results that were compatible with each outcome domain in each study were sought (e.g. for all measures, time points, analyses), and if not, the methods used to decide which results to collect. | 9-10 |
|  | 10b | List and define all other variables for which data were sought (e.g. participant and intervention characteristics, funding sources). Describe any assumptions made about any missing or unclear information. | 9-10 |
| Study risk of bias assessment | 11 | Specify the methods used to assess risk of bias in the included studies, including details of the tool(s) used, how many reviewers assessed each study and whether they worked independently, and if applicable, details of automation tools used in the process. | 11-12 |
| Effect measures | 12 | Specify for each outcome the effect measure(s) (e.g. risk ratio, mean difference) used in the synthesis or presentation of results. | 10-11 |
| Synthesis methods | 13a | Describe the processes used to decide which studies were eligible for each synthesis (e.g. tabulating the study intervention characteristics and comparing against the planned groups for each synthesis (item #5)). | 9-10 |
|  | 13b | Describe any methods required to prepare the data for presentation or synthesis, such as handling of missing summary statistics, or data conversions. | 10 |
|  | 13c | Describe any methods used to tabulate or visually display results of individual studies and syntheses. | 10-11 |
|  | 13d | Describe any methods used to synthesize results and provide a rationale for the choice(s). If meta-analysis was performed, describe the model(s), method(s) to identify the presence and extent of statistical heterogeneity, and software package(s) used. | 10-11 |
|  | 13e | Describe any methods used to explore possible causes of heterogeneity among study results (e.g. subgroup analysis, meta-regression). | 10-11 |
|  | 13f | Describe any sensitivity analyses conducted to assess robustness of the synthesized results. | N/A |
| Reporting bias assessment | 14 | Describe any methods used to assess risk of bias due to missing results in a synthesis (arising from reporting biases). | 10-11 |
| Certainty assessment | 15 | Describe any methods used to assess certainty (or confidence) in the body of evidence for an outcome. | 11-12 |
| **RESULTS** | | |  |
| Study selection | 16a | Describe the results of the search and selection process, from the number of records identified in the search to the number of studies included in the review, ideally using a flow diagram. | 12 and Figure 1 |
|  | 16b | Cite studies that might appear to meet the inclusion criteria, but which were excluded, and explain why they were excluded. | 12 |
| Study characteristics | 17 | Cite each included study and present its characteristics. | 12-13 and Supplementary file 3 |
| Risk of bias in studies | 18 | Present assessments of risk of bias for each included study. | 17 and Supplementary file 7 |
| Results of individual studies | 19 | For all outcomes, present, for each study: (a) summary statistics for each group (where appropriate) and (b) an effect estimate and its precision (e.g. confidence/credible interval), ideally using structured tables or plots. | Table 2, Figures 2-7, Supplementary file 4 and 5 |
| Results of syntheses | 20a | For each synthesis, briefly summarise the characteristics and risk of bias among contributing studies. | 13-16 |
|  | 20b | Present results of all statistical syntheses conducted. If meta-analysis was done, present for each the summary estimate and its precision (e.g. confidence/credible interval) and measures of statistical heterogeneity. If comparing groups, describe the direction of the effect. | 13-16 |
|  | 20c | Present results of all investigations of possible causes of heterogeneity among study results. | 16 |
|  | 20d | Present results of all sensitivity analyses conducted to assess the robustness of the synthesized results. | N/A |
| Reporting biases | 21 | Present assessments of risk of bias due to missing results (arising from reporting biases) for each synthesis assessed. | 17 |
| Certainty of evidence | 22 | Present assessments of certainty (or confidence) in the body of evidence for each outcome assessed. | 17 |
| **DISCUSSION** | | |  |
| Discussion | 23a | Provide a general interpretation of the results in the context of other evidence. | 18-21 |
|  | 23b | Discuss any limitations of the evidence included in the review. | 20-21 |
|  | 23c | Discuss any limitations of the review processes used. | 21-23 |
|  | 23d | Discuss implications of the results for practice, policy, and future research. | 20-21 |
| **OTHER INFORMATION** | | |  |
| Registration and protocol | 24a | Provide registration information for the review, including register name and registration number, or state that the review was not registered. | 7 |
|  | 24b | Indicate where the review protocol can be accessed, or state that a protocol was not prepared. | 7 |
|  | 24c | Describe and explain any amendments to information provided at registration or in the protocol. | N/A |
| Support | 25 | Describe sources of financial or non-financial support for the review, and the role of the funders or sponsors in the review. | 2 |
| Competing interests | 26 | Declare any competing interests of review authors. | 2 |
| Availability of data, code and other materials | 27 | Report which of the following are publicly available and where they can be found: template data collection forms; data extracted from included studies; data used for all analyses; analytic code; any other materials used in the review. | 24 |

*From:*  Page MJ, McKenzie JE, Bossuyt PM, Boutron I, Hoffmann TC, Mulrow CD, et al. The PRISMA 2020 statement: an updated guideline for reporting systematic reviews. BMJ 2021;372:n71. doi: 10.1136/bmj.n71

For more information, visit: <http://www.prisma-statement.org/>

# **Supplementary Table 2:** Combined search strategy employed to address the research question.

|  | Search strategy (MEDLINE) |
| --- | --- |
| **Nuts with MeSH terms** | (TX Almond*) OR *(MH "Prunus dulcis")* OR *(TX "Prunus dulcis")* |
|  | (TX “Brazil nut”) OR *(TX "Brazil nuts")* OR *(MH "Bertholletia")* OR *(TX Bertholletia)* |
|  | (TX “Cashew nut”) OR *(TX "Cashew nuts")* OR *(TX Cashew*)*OR *(MH "Anacardium")* OR *(TX Anacardium)* |
|  | (TX Chestnut) |
|  | (TX Hazelnut*) OR *(MH "Corylus")*OR *(TX corylus)* |
|  | (TX Macadamia*) OR *(MH "Macadamia")* |
|  | (TX Nut) OR *(MH "Nuts")* OR *(TX Nuts)* |
|  | (TX Peanut*) OR *(MH "Arachis")* OR *(TX Arachis)* |
|  | (TX Pecan*) OR *(MH "Carya")* OR *(TX carya)* |
|  | (TX “Pine nut”) OR *(TX "Pine nuts")* |
|  | (TX Pistachio*) OR *(MH "Pistacia")* OR *(TX Pistacia)* |
|  | (TX Walnut*) OR *(MH "Juglans")*OR*(TX juglans)* |
| **Health with MeSH terms** | *(MH "Heart+")* OR (TX heart) |
|  | *(MH "Cardiovascular Diseases+")* OR *(TX* "*cardiovascular disease***")* OR ( TX cardiovascular) OR *(TX* *CVD*) |
|  | (TX coronary) OR *(MH*"*Coronary Disease*+") OR *(TX "coronary disease**") OR *(TX*"*coronary heart disease*") OR*(TX* *CHD*) |
|  | *(MH "Coronary Artery Disease")*OR*(TX "Coronary Artery Disease")*OR*(TX "Ischemic heart disease")*OR*(TX "Ischaemic heart disease")*OR*(MH "Myocardial Ischemia+")*OR*(TX "Myocardial Ischemia") (TX "Myocardial Ischaemia")*OR *(MH "Heart Diseases+")*OR*(TX "heart disease*")*OR*(MH "Myocardial Infarction+")*OR*(TX "Myocardial Infarction")*OR*(TX "heart attack")*OR*(MH "Stroke+")*OR*(TX Stroke)*OR*(MH "Heart Failure+")*OR *(TX "Heart failure")* |
|  | *(MH* "*Cholesterol*+") OR (TX cholesterol) OR *(MH "Cholesterol, LDL")* OR *(TX "cholesterol, LDL")* OR (TX LDL) *OR (TX "low-*d*ensity lipoprotein*")*OR *(MH "Lipoproteins, LDL+")* OR *(TX "Lipoproteins, LDL")* |
|  | *(MH "Atherosclerosis+")* OR (TX atherosclerosis) |
|  | *(MH "Lipids+")* OR (TX lipid*) |
|  | *(MH "Blood Pressure+")* OR (TX "Blood Pressure") |
|  | *(MH "Hypertension+")* OR (TX hypertension) |
| **Population with MeSH terms** | *(MH "Humans")*OR*(TX Human*)*OR*(MH "Men+")*OR*(TX men)*OR*(TX man) O*R *(MH "Male")*OR*(TX male)*OR*(TX males)*OR*(MH "Women")*OR*(TX women)*OR *(TX woman)*OR*(MH "Female")*OR*(TX female*)*OR*(MH "Infant")*OR*(TX infan*)*OR*(MH "Child+") OR (TX child*)*OR *(MH "Adolescent")*OR *(TX adolescen*)*OR*(MH "Adult+")*OR*(TX adult*)* |
| **Combined search strategy** | (TX Almond*) OR (MH "Prunus dulcis") OR (TX "Prunus dulcis") OR (TX “Brazil nut”) OR (TX “Brazil nuts”) OR (MH "Bertholletia") OR (TX Bertholletia) OR (TX “Cashew nut”) OR (TX "Cashew nuts") OR (TX Cashew*) OR (MH "Anacardium") OR (TX Anacardium) OR (TX Chestnut*) OR (TX Hazelnut*) OR (MH "Corylus") OR (TX corylus) OR (TX Macadamia*) OR (MH "Macadamia") OR (TX Nut) OR (MH "Nuts") OR (TX Nuts) OR (TX Peanut*) OR (MH "Arachis") OR (TX Arachis) OR (TX Pecan*) OR (MH "Carya") OR (TX carya) OR (TX “Pine nut”) OR  (TX “Pine nuts”) OR (TX Pistachio*) OR (MH "Pistacia") OR (TX Pistacia) OR (TX Walnut*) OR (MH "Juglans") OR (TX juglans)  AND  (MH "Heart+") OR (TX heart) OR (MH "Cardiovascular Diseases+") OR (TX "cardiovascular disease*") OR ( TX cardiovascular) OR (TX CVD) OR (TX coronary) OR (MH "Coronary Disease+") OR (TX "coronary disease*") OR (TX "coronary heart disease") OR (TX CHD) OR (MH "Coronary Artery Disease") OR (TX "Coronary Artery Disease") OR (TX "Ischemic heart disease") OR (TX "Ischaemic heart disease") OR (MH "Myocardial Ischemia+") OR (TX "Myocardial Ischemia") (TX "Myocardial Ischaemia") OR (MH "Heart Diseases+") OR (TX "heart disease*") OR (MH "Myocardial Infarction+") OR (TX "Myocardial Infarction") OR (TX "heart attack") OR (MH "Stroke+") OR (TX Stroke) OR (MH "Heart Failure+") OR (TX "Heart failure") OR (MH "Cholesterol+") OR (TX cholesterol) OR (MH "Cholesterol, LDL") OR (TX "cholesterol, LDL") OR (TX LDL) OR (TX "low-density lipoprotein*") OR (MH "Lipoproteins, LDL+") OR (TX "Lipoproteins, LDL") OR (MH "Atherosclerosis+") OR (TX atherosclerosis) OR (MH "Lipids+") OR (TX lipid*) OR (MH "Blood Pressure+") OR (TX "Blood Pressure") OR (MH "Hypertension+") OR (TX hypertension)  AND  (MH "Humans") OR (TX Human*) OR (MH "Men+") OR (TX men) OR (TX man) OR (MH "Male") OR (TX male) OR (TX males) OR (MH "Women") OR (TX women) OR (TX woman) OR (MH "Female") OR (TX female*) OR (MH "Infant") OR (TX infan*) OR (MH "Child+") OR (TX child*) OR (MH "Adolescent") OR (TX adolescen*) OR (MH "Adult+") OR (TX adult*) |

# **Supplementary Table 3:** Characteristics of included randomised controlled trials

| **Reference, country** | **Study design** | **Nuts** | **Nut dose (g/d)** | **Study duration (week)** | **Participants and sample size** | **Age (years)** | **BMI  (kg/m2)** | **Other subject characteristics** | **Interventions** | **Background diet** | **Methods** | **Results** | **Quality appraisal** |
| --- | --- | --- | --- | --- | --- | --- | --- | --- | --- | --- | --- | --- | --- |
| Abazarfard et al. (2014), Iran | RCT parallel | Almonds | 50 | 12 | n= 108 overweight and obese premenopausal women.  n=100 completed the study, 50 per group. | 20 - 55 | ≥25 | Minimal exercise, without diabetes, chronic illness, or taking lipid lowering medications. | Intervention group consumed 50g raw almond (2 snacks of 25g) per day. Control group consumed compensatory serving from the meat and fat exchange list (i.e. sunflower or corn oil). | Balanced hypocaloric diet. | Compliance by telephone call every 15 days. Dietary intake by 24-hr dietary recall at baseline, end of study and the end of each month. Lipid profiles (plasma TC, HDL, LDL, and TG) by a photometric method. BP by a calibrated automatic digital monitor. | Intervention group had significant decreases in TG, TC and TC: HDL than the control group. LDL decreased in both groups, control group had significant decrease. Intervention group had non-significant increase in HDL, while control group had significant increase. SBP and DBP decreased in both groups. DBP decreased in the intervention group was statistically significant. | HIGH |
| Abbaspour et al. (2019), US | RCT parallel | Mixed nuts | 42.5 (split between two servings) | 8 | n=54 overweight and obese adults (F: n=22, M: n= 32). n= 6 patients dropped out. n=48 patients completed the study, 24 per group. | C: 29.1 ± 9.3 I: 30.4 ± 10.2 | ≥27 | Without being allergic to nuts or wheat or had a history of significant chronic or inflammatory diseases or non-smokers, pregnant or lactating women, and those taking medications or supplements known to affect markers of cardiovascular disease risk factors. | Intervention group consumed 42.5 g/d (split between two servings) of mixed nuts. Control group consumed two daily isocaloric servings of unsalted pretzels totalling 69 g (173 mg Na). | Continue usual dietary habits. | Compliance: mailed or texted once a week to be reminded of their snack consumption and they were also asked at every visit if they consumed their snacks and whether they had any issues. Dietary intake by two 24 h dietary recalls. BP was measured after participants were seated for 10 min and averages were made of duplicate readings. Serum TG, TC and HDL were measured using assay kits from Stanbio Laboratory. LDL was calculated using the following formula: LDL = TC − (HDL) − (TG/5). | No significant changes were detected between the two groups for TC, LDL, and HDL. TG levels increased significantly from week 4 to week 8 in the pretzel group, although no difference was detected between the beginning and end of the intervention. TG levels were not changed within the nut group. TC remained unaffected, a significant reduction in HDL and a significant increase in LDL were observed from baseline to week 4 in the pretzel group with no significant changes in the nut group. There were no significant differences BP over time and between groups. | HIGH |
| Agebratt et al. (2016), Sweden | RCT parallel | Mixed nuts | 1.24 per kg body weight (7 kCal/kg/ body weight) | 8 | n=30 healthy non-obese subjects (M: 18 and F: 12). | 23.5 ± 3.7 | 22.3 ±1.9 | Without pre-existing medical conditions or use of thyroid hormone replacement. | Intervention group consumed 7 kCal/kg/body weight of nuts daily as snacks between meals for 8 weeks. Control group consumed 7 kCal/kg/body weight of fruit. | Habitual diet. | Compliance by food diaries. Dietary intake by 3 consecutive day weighed food record including a Saturday or Sunday at baseline and end of trail period. Lipid profiles (plasma TC, HDL, LDL, TG, LDL:HDL) by fasting blood samples and standard laboratory tests analysed according to routines at Department of Clinical Chemistry at the Link√∂ping University Hospital. BP using mercury sphygmanometer. | Fruit and nut groups had non-significant decrease in TC, HDL, LDL, LDL:HDL and DBP from baseline and for changes between the two groups.   Fruit group had a significant decrease and nut group had a non-significant decrease in SBP from baseline. Changes between the two groups were non-significant.   Compliance expressed as reported intake of nuts or fruit in kcal/day/kg bodyweight during the study period compared with the goal of 7 kcal/day/kg body weight was 107.6 ± 8.8% in the nut group and 98.8 ± 9.4% in the fruit group (p = 0.014 between groups). | HIGH |
| Al Abdrabalnabi et al. (2020), US and Spain | RCT parallel | Walnuts | 30-60  (15% energy) | 104 | n=708 having or being free of MetS. n=10 excluded from analysis due to incomplete MetS data. n=698 Included subjects. n=73 WD. n=625 completed the study. | 62 - 79 (Mean: 69.1) | < 40 (Mean 73.5) | Without being extremely obese BMI ≥ 40 kg/m2, and uncontrolled diabetes or hypertension, or allergic to walnuts. | Intervention group consumed 15% of energy (≈30–60 g/d). Control group abstained from walnuts. | Habitual diet | Compliance by using nutrient analysis from dietary recalls and by assessing the red blood cell content of α-linolenic acid, as proportion of total identifiable fatty acids. Dietary intake by five unannounced 24-hour diet recalls or three-day food records that were obtained every six months. BP was measured at baseline and at scheduled bimonthly clinic visits using an Omron BP762 Series blood pressure cuff. All of the blood samples were shipped to the appropriate laboratory for biochemical determinations. All samples were concurrently run in the same laboratory to reduce assay variability. | A significant decrease in plasma TG and HDL was observed in both groups. The walnut group showed a greater decrease in SBP and DBP as compared with the control group. | HIGH |
| Alves et al. (2014), Brazil | RCT parallel | Peanuts | 56 | 4 | n=76 overweight and obese males subjects (BMI ranging from 26 to 35 kg/m2 and stable weight (±3kg) during the previous 3 months).  n= 7 WD. n=69 completed the study. n=65 included in final analysis (C: n=22, conventional peanut: n=22, and high-oleic peanuts: n=21). | 18 and 50 | 26 to 35 | Without acute diseases and/or eating disorders or any chronic disease other than obesity. Without the use of medications that might affect study outcomes over the 3 months prior to study initiation and high alcohol intake (>168 g/week). | Intervention group 1 consumed conventional peanuts group and the intervention group 2 consumed high-oleic peanuts group. Control group consumed a biscuit with no peanuts. | Hypocaloric diet. | Compliance NR. Dietary data by 3-day food records (before baseline assessments and during the fourth week of the study). Serum lipids (TC, HDL, LDL, TG) were quantified in fasting serum by automated analyser systems using commercial assay kits. | Plasma TC decreased significantly only in the control group. All the groups showed a significant decrease in HDL. TGs levels were significantly reduced in the conventional peanuts and high-oleic peanuts groups. The conventional peanuts group was the only group that showed a significant increase in LDL:HDL ratio. | HIGH |
| Baer et al. (2019), US | RCT crossover | Cashews | 42 | 4 | n=42 healthy adults, 20 M and 20 F. n=3 discontinued. n=39 completed the study.  n=40 included in analysis. | 25 -75 (Mean: 56.8 ± 1.7) | 20 - 38 (Mean 29.0 ± 0.7) | Without kidney disease, liver disease, gout, hyperthyroidism (overactive thyroid), untreated or unstable hypothyroidism (underactive thyroid), certain cancers, gastrointestinal disease, pancreatic disease, other metabolic diseases, or malabsorption syndromes. Without use of cholesterol lowering medication, treat hypertension, cardiovascular disease, type 2 diabetes, given birth in the previous 12 months, pregnant or planning to be pregnant, use of prescription or over the counter antiobesity medications or supplements, smokers, history of eating disorders, allergy or adverse reaction to cashew nuts or other nuts. | Intervention group consumed the base diet with 42 g of cashew nuts/day. Control group consumed the base diet with no cashew nuts. | A typical western diet. | Compliance by providing all meals and supervised consumption. Dietary intake by providing subjects breakfast and dinner, Monday through to Friday in a dining facility supervised by a dietitian. Lunch and weekend meals were provided to subjects and were weighted. Serum TC, LDL, HDL, TG, apo A1, apo A2, apo B and (a) were measured with a clinical chemistry analyser. Arterial stiffness was measured from the PWV. The distance between the carotid and femoral arteries was measured, and the PWV was determined by dividing the measured distance by the pulse transit time. | There were no significant differences in the concentration of lipids and lipoproteins. There were no differences in pulse-wave velocity after consumption of the 2 diets. | HIGH |
| Bamberger et al. (2017), Germany | RCT crossover | Walnuts | 43 | 24 | n=204 healthy non-smoking subjects (men and post-menopausal women).  n= 194 completed the study (60 M and 134 F), 96 in group 1 and 98 in group 2.  n=2014 ITT analysis. | 50-86 (Mean: 63 ± 7) | 25.4 to ± 0.29 | Without history of cardiovascular and atherosclerotic disease, a known allergy to tree nuts, a vegan or ovo-lacto vegetarian lifestyle, and patients on regular medication. | Intervention group consumed walnut-enriched diet (43 g of shelled walnuts/day) replacing 70 g carbohydrates or 30 g of (saturated) fat or both 35 g carbohydrate and 15 g fat with nuts. Control diet consumed nut-free western-type diet. | A nut-free western-type diet . | Compliance and dietary intake by four day food record completed on 7 occasions throughout study. EDTA-containing blood samples were used to analyse fasting lipid parameters. TC, TG, apo B and apo A1 were directly measured on an autoanalyser by using ready-to-use reagent kits. HDL was measured after precipitation with heparin and manganese (II) chloride (polyanion precipitation). LDL was calculated by subtracting HDL from the TC in the infranatant of the ultracentrifugation. Endothelial activation markers sVCAM-1 and endothelin-1 were determined using commercially available ELISA kits. | Walnut consumption significantly reduced TC, LDL, TG and ApoB when compared with the control period, but did not significantly increase HDL and apo A1.  Walnut consumed to replace CHO, fat or both did not significantly change TC, LDL, HDL, TG, apo A1 and apo B when comparing the differences between groups and the control period.   Walnuts given as a meal versus a snack resulted in no significantly change in TC, LDL, HDL, TG and apo A1 when comparing the differences between groups and the control period. Walnuts given as a meal versus a snack resulted in a significant change in apo B when comparing the differences between groups, but not significant when comparing walnuts and the control.   Walnut consumption did not significantly change endothelial markers VCAM-1 and endothelin-1 which remained unaffected during the walnut diet. | HIGH |
| Barbour et al. (2015), Australia | RCT cross-over | Peanuts | M: 84 and F: 56 (15%-20% energy). | 12 | n=69 healthy overweight males or post-menopausal female. n= 61 completed the study (29 M and 32 F). | 65 ± 7 | 31 ± 4 | Smokers, regular nut consumers and people with cardiovascular disease, hypertension (>160/100 mmHg), a thyroid condition or nut allergy or those consuming‚ ≥ 40 g of alcohol/day were excluded. | Intervention group consumed high oleic peanuts roasted unsalted with skins. Subjects were asked to consume any skins which had fallen off in the packaging. Control group consumed a nut free diet (habitual diet). | Habitual diet. | Compliance by diet diaries. Dietary intake by four day weighed food diaries. Fasting plasma TGs, TC and HDL are determined using a commercial assay kit with a Konelab 20XT clinical chemistry analyser. LDL as calculated using the Friedewald equation. | No significant differences observed in lipids between intervention and control groups. | HIGH |
| Berryman et al. (2015), US | RCT cross-over | Almonds | 42.5 | 6 | n=61 health men and women with elevated LDL levels.  n=48 complete the study. | 49.9 ± 9.4 | 26.2 ± 2.8 | Without chronic illness, did not use tobacco or consume, >3 servings/week of alcohol, agreed to stop taking vitamin/mineral, lipid-lowering, or other supplements, taking cholesterol-lowering medications, vegetarian diet, weight gain/loss of ≥10% within the previous 6 months, and pregnant, lactating, or wanting to become pregnant before or during the study. | Intervention group consumed 2.5 g (1.5 oz.) unsalted, whole, natural almonds with skins (253 kcal/day). Control group consumed 106 g banana muffin + 2.7 g butter (273 kcal/day). | All meals and snacks were prepared. Diets were identical with the exception of the snack that was provided. | Compliance and dietary intake by daily weigh-ins (Monday to Friday) and daily food logs (Monday to Sunday) to assure that participants were eating all and only study foods. TC and TG were determined by standard enzymatic and spectrophotometry procedures. HDL was measured according to the modified heparin-manganese procedure. LDL was calculated using the Friedewald equation. Apo B and apo A1 were calculated using results from the VAP test and patented equations. | The intervention diet significantly decreased LDL compared with control. The intervention diet reduced HDL significantly less than the control diet. The intervention diet significantly improved apoB compared to the control diet. The intervention diet significantly decreased the TC/HDL, LDL/HDL, and apo B/apo A1 ratios. | HIGH |
| Bowen et al. (2019), Australia | RCT parallel | Almonds | 56 | 8 | n=94 adults who were overweight or obese, had elevated WC, fasting plasma glucose or type 2 diabetes, and were weight stable (n=76 commended trial, n=39 almond snack and n=37 biscuit snack).  n= 10 WD prior to commencement and n=2 WD during trial. n=74 completed the study.  . | 20 - 70  (I: 60.8 ± 6.6, C: 60.6 ± 8.8) | >27  (I: 34.4 ± 6.2 C: 33.2 ± 4.9) | Without taking any medication/supplements that may affect primary outcome, chronic health conditions and lifestyle factors that may affect the study outcomes or participant's health at the discretion of the overseeing physician, allergies to nuts, dairy, gluten or not willing to consume test foods, no history of heavy alcohol consumption, not a current smoker or history of smoking during 6 month prior to the study, and women attempting to become pregnant, pregnant or lactating. | Intervention group consumed 68 g/d (28 g morning and afternoon snack) raw almonds. Control group consumed 72 g/d (energy matched) commercial sweet, nut and seed free biscuit per day (36g morning and afternoon snack). Both groups were advised to maintain their usual ad litum diet. | Usual ad litum diet. | Intervention compliance was assessed using a daily, self- reported checklist and plasma a-tocopherol and fatty acids were measured as objective biomarkers of intervention and control compliance. Dietary intake by three day weighed food records. Serum TC, HDL, TG were measured using commercial enzymatic kits on a Beckman AU480 clinical analyser. Friedewald equation was used to calculate LDL levels. | There was no statistically significant differential response between the groups for serum TC, LDL, HDL and TG concentrations. Serum TC/HDL ratio was significantly reduced in the intervention group, but this reduction was not significantly different from the control group. Gender modified this response such that in women TC/HDL ratio significantly reduced after the intervention compared to the control, but not significant in men. | HIGH |
| Burns-Whitmore et al. (2014), US | RCT cross-over | Walnuts | 28.4 (6/week) | 8 | n=26 recruited.  n=20 completed the study (n=16 females, n=4 males). | 21 - 64 (Mean: 38 ± 3) | 23 ± 1 | With lacto ovo vegetarians for <3 months with normal TC and TG levels and without pre-existing medical conditions. Participants did not smoke, drink alcohol, take supplements, take cholesterol lowering medications or on daily prescription anti-inflammatory medication. | Intervention group consumed walnut diet (28.4 g, 6/week). Control group consumed standard egg, (6/week).  4 weeks washout between treatments. | Habitual diet | Compliance by: 1) counselling with the dietitian; 2) maintaining a daily diary to record deviations in food and medicine intake; 3) obtaining three 24-hour recalls; 4) assessing fatty acid composition of erythrocyte membranes. Dietary intake by 24-hour recalls. Lipid profiles and lipoprotein analyses by enzymatic colorimetric assays with the use of the Bayer 550 Express Chemistry analyser. | WD had a significant decrease in TC, TG and apo B compared to control diet but not n-3 egg diet. WD had a significant decrease in total:HDL compared to n-3 egg diet and control diet. | HIGH |
| Campos et al. (2020), Brazil | RCT parallel | Pecans | 30 | 12 | n=204 participants with stable coronary artery disease for >60 days, n=67 control, n=68 pecan and n=69 olive oil.  n=31 withdrawals and n=1 death. n=204 included in final analysis by intention-to-treat. | 40 - 80 (C1: 60.40 ± 8.54,  C2: 57.51 ± 11.72, I1: 59.59 ± 8.40) | C: 29.06 ± 3.98 I1: 29.78 ± 5.69 I2: 29.12 ± 4.36 | Without psychiatric diseases, extreme obesity (≥ 40 kg/m2), life expectancy <6 months, pregnancy or lactation, renal insufficiency on dialysis, wheelchair-bound, uncontrolled hypo- or hyperthyroidism, congestive heart failure, use of ditary supplements, long-term use of anti-inflammatory and immunosuppressive drugs, and participation in other clinical trials. | Intervention group consumed the same dietary pattern as control group however were supplemented with pecans (30 g/d) as a substitute for other foods. Control group consumed a diet meeting the Brazilian nutritional guidelines. | The Brazilian nutritional guidelines. | Compliance: adherence assessed by the presence in consultations). Dietary intake was assessed by means of the 24-h dietary recall. Interviews were conducted by trained nutritionists and/or academic nutritionists. Serum TC, LDL, TG levels were assessed by an enzymatic colorimetrtic method, and HDL was assessed by immunoprecipitation. | No difference regarding final means of SBP and DBP between groups at the end of the study. No difference regarding LDL, TC, HDL, TG, LDL/HDL ratio, TG/HDL ratio according to groups. However, compared to the control group and the olive oil group, pecan group had significantly reduced TC/HDL ratio values. | HIGH |
| Canales et al. (2007), Spain | RCT cross-over | Walnuts | 21.4 (150g/week in steaks and sausages) | 5 | n=25 men and postmenopausal women. n=22 completed the study. | 54.8 ± 8.3 | 29.6 ± 3.4 | Without familiar hypercholesterolemia and/or type 1 diabetes, those taking any hypolipemiant, antihypertensive or anti-inflammatory drugs and those receiving hormonal substitutive therapy. With at least one of the following criteria: serum total cholesterol ≥5.69mmol/L, smoking habit (≥10 cigarettes per day), and/or hypertension (SBP≥140 mmHg and/or DBP≥90 mm Hg). With a high meat consumption (5 times/week). | Intervention group consumed 4x150 g restructured walnut paste enriched meat steaks and 150 g ratio of walnut paste enriched meat sausages/week (contains 20% walnut paste, additional 473.2 kJ/100g meat, 12.9 g fat/100g meat, less SFA and MUFA, 55.1 g PUFA/100 g meat). Control group consumed low fat meat: 4x160 g restructured low fat steaks and 150 g ration of low fat sausages/week. | Habitual diet | Compliance by intake with regard to frequency, dates and number of steaks consumed and by measuring plasma [γ tocopherol] at 5 weeks. Dietary intake by participants daily recording the type of food consumed. HDL by by enzymatic colorimetric method. | HDL (mmol/L) not different. HDL +0.10 mg/dL, +8.3% (p=0.316) | HIGH |
| Carvalho et al. (2015), Brazil | RCT parallel | Brazil nuts | 13 | 12 (+6 days) | n=89 male and females diagnosed with dyslipidaemia and hypertensive patients already receiving lipid-lowering drugs or both conditions, n=44 intervention and n=45 control group.  n=12 loss to follow-up or WD. n= 77 completed the study. | 40 - 80 | 29.54 ± 5.60 (I: 29.9 ± 6.5 and C: 29.3 ± 4.8) | Without a history of thyroid disease, thyroid medication use, chronic renal failure with glomerular filtration rate, currently or previously having ingested supplements containing > 20 μg selenium/day or presenting excessive consumption of Brazil nuts in the past year, having plasma selenium levels above 125 μg/L, being current smokers, and having been in a rigorous exercise/weight-reduction program within the 3 months before entering the study. | Intervention group consumed 13 g/d Brazil nut flour (partially defatted granulated Brazil nut). Control group consumed 11 g/d artificially flavoured dyed cassava flour. | The Brazilian nutrition recommendations. | Compliance by plasma selenium levels were used as markers of Brazil nut consumption. Dietary intake was verified monthly at the check-up via 24 h diet reminder. Serum concentrations of TG, TC, and HDL were assayed by enzymatic-colorimetric methods. LDL values were obtained according to the Friedewald formula. Serum levels of apo A1 and apo B were measured with immunoturbidimetry. | No significant differences within or between groups in LDL, HDL, TG, apo B and the apo B / apo A1 ratio. After 90 days, TC and apo A1 decreased significantly only in the Brazil nut group, however there was no difference between groups. | HIGH |
| Casas-Agustench et al. (2011), Spain | RCT parallel | Mixed nuts | 30 (walnuts:15 g, almonds:7.5 g, hazelnuts:7.5 g) | 12 | n=52 men and women. n=2 WD. n=50 completed the study, 25 per group. | 26 - 63 (Mean: 52.9 ± 8.4) | 31.6 ± 2.8 (C: 31.6 and I: 30.0) | With ≥3 MetS criteria. Without nut allergy, history of alcohol abuse or drug dependence, type 2 diabetes endocrine disorders, acute or chronic infection; chronic inflammatory dx, history of cancer; leucocytosis (>10 cells/nL), treatment with anti-inflammatory, corticosteroid, hormonal or antibiotic agents, and restrictive diet or weight change≥5 kg past 3 months prior to study. | Intervention group consumed a prudent diet supplemented with 30 g/d of raw unpeeled nuts.  Control group consumed a prudent diet excluding intake of nuts and peanuts. | Prudent diet. | Compliance by collecting empty nut packages and by measuring plasma alpha-linolenic acid levels. Dietary intake by 3- day food record every 4 weeks. Fasting serum TC and TG concentrations by standard enzymatic methods in an automatic analyser. HDL was measured by a precipitation technique, LDL calculated. | TC and LDL had a non-significant decreased in the control group, without differences in treatment effects with the intervention group. No changes of HDL or TGs occurred in either diet group. | HIGH |
| Chen et al. (2015), US | RCT cross-over | Almonds | 85 | 6 | n=51 patients with angiographically proven coronary artery disease.  n=6 dropped out/lost to follow up. n=45 completed study. | 21 - 80 (Mean: 61.8 ± 8.6) | 20 - 41 (Mean: 30.2 ± 5.1) | Without planning on or being pregnant, chronic illness, treatment with an investigational new drug within the last 30 day, history of a psychological illness or condition, taking dietary supplements (including multivitamins and herbal supplements), and eating any nuts within 1 month of enrolment. | Intervention group consumed 85 g/d almonds which were pre-packaged in ~28.4 g servings. Control group consumed a diet absent of nuts. | A diet absent of nuts. | Compliance by frequent phone calls and package bags were collected. Dietary intake by a self- administered semi-quantitative FFQ before the run in and at the end of each treatment period. Lipid profiles (plasma TC, HDL and TG) were determined with a clinical chemistry analyser and LDL measured using employing an Olympus AU400 automated analyser using kit. Vascular reactivity by FMD, PAT and PWV. Brachial artery ultrasound was used to determine FMD and hyperemic flow measured in the brachial artery. BP using an automated physiologic recorder. | The lipid profiles of the subjects, including TC, HDL, LDL, TG, and small density LDL, was unchanged during the study. No significant effect on BP. Endothelial function was evaluated plasma E-selectin, VCAM-1, and NO. No significant effect on vascular reactivity by FMD, PAT and PWV. | HIGH |
| Chen et al. (2017), China | RCT cross-over | Almonds | 60 | 12 | n=40 males and female with type 2 diabetes and regular use of prescribed oral hypoglycemic agents.  n=7 WD during trial.  n=33 completed the study. | 54.9 ± 10.5 | I: 25.6 ± 4.3,  C: 25 ± 4.1 | Without regular use of insulin, oral steroids or anti-inflammatory agents, ≥5% body weight change in the last 6 months, diagnosed cardiovascular disease, stroke, gastrointestinal diseases, inflammatory bowel disease, chronic kidney disease, hepatobiliary disease, renovascular disease, endocrine diseases, hyperuricemia, autoimmune diseases, active treatment for cancer of any type ≤1 y, poor hyper-tension control (SBP‚ ≥150 mmHg and/or DBP‚ ≥95 mmHg), known allergies to nuts of any kind, frequent nut consumption(≥3 oz./week), regular use of any dietary supplements or homeopathic remedies, daily ethanol intake of ≥2 drinks and smoking. | Intervention group consumed 60 g roasted, unsalted whole almonds with skins. Control group consumed a diet that provided daily calories from CHO, protein, and fat at 55, 17, and 28%, respectively. | Study meals were tailored to meet daily energy need of each subject. | Compliance and dietary intake by weighing and recording unconsumed foods. Also a calendar was used to monitor the consumption of almond foods. Lipid profiles (TC, LDL, HDL and TG) in serum were determined using a SYNCHRON LX20 Pro clinical chemistry analyser. Apo A1 and apo B in serum were measured using PEG enhanced immunoturbidimetric assays with the SiemensAdvia 1800 analyser. Plasma ICAM-1, E-selectin, and endothelin-1 were assessed using human sICAM-1/CD54 Quantikine ELISA kit, human E-selectin/CD62E ELISA kit and endothelin-1Quantikine ELISA kit, respectively. Serum NO was determined by a colorimetrictotal nitric oxide assay kit. | Lipid profile (TC, LDL, HDL and TG) did not differ between diets in all subjects. Intervention group significantly decreased serum NO values as compared to control group. Intervention group non-significantly decreased E-selectin as compared to control group. Both ICAM-1 and endothelin-1 contents remained similar during the course of the trial. | HIGH |
| Chen et al. (2020), China | RCT parallel | Peanuts | 25 (~28 mL/d) | 52 | n=251 subjects, 155 F and 96 M. n=24 loss to follow-up. n= 227 completed the study. n=243 subjects, 151 F and 92 M included in the ITT analysis. | 40 - 65 (F: 54.2 ± 5.2, M:57.2 ± 7.2) | I1: 23.20 ± 2.60 C1: 23.23 ± 2.86 C2: 23.63 ± 3.19 | Without cardiovascular diseases, diabetes mellitus, liver or other metabolic dysfunction, and mental disability. | Intervention group consumed peanut oil (28 ml/day, 43% oleic acid and 34% linoleic acid). Control group 1 consumed corn oil consumed 28 ml/day, 55% linoleic acid and 28% oleic acid and control group 2 consumed blend oil (28 ml/day, extra 7% alpha linoleic acid).  Has a similar fatty acid profile to peanut oil except for alpha linoleic acid. | Dietary guidelines for Chinese residents. | Compliance by analysing fatty acid composition of erythrocyte membranes. Dietary inatke by FFQs. TC, HDL, LDL, TG, apo A1 and apo B were measured by colorimetric methods using commercial kits. | SBP, DBP, TC, LDL, TG, apo A1, and apo B were improved in all participants, but with no statistically significant difference among the three groups. | HIGH |
| Chisholm et al. (1998), New Zealand | RCT cross-over | Walnuts | 78 | 4 | n=21 men with polygenic hyperlipidaemia. n=5 did not provide full set of dietary records. n=16 completed the study. | <65 | NR | With TC levels between 5.5 ± 7.5 mmol/L. Without symptoms of disease or family history of premature coronary heart disease, familial hypercholesterolaemia, familial combined hyperlipidaemia or secondary hyperlipidaemia. | Intervention diet contained walnuts contributing 20% of total energy and 55% of energy from fat in the walnut diet with the remaining fat coming from foods chosen by the participants. Control (low fat) diet contained fat from a variety of foods other than nuts. | Subjects ate all meals at home and followed their usual activities. | Compliance by increases of linoleic acid in plasma TG and alpha linoleum acid. Dietary intake by an initial 4-day diet record during the run-in period, 8 day diet records were collected during each experimental period by recording food eaten on two different days each week. Lipid profiles (TC, HDL and LDL) in plasma and lipoprotein fractions (apo A and apo B) were measured by enzymatic methods using kits and calibrators from Boehringer Mannheim and TG were measured enzymatically with Roche Diagnostic kits on a Cobas Fara analyser. | TC, LDL and apo B were lower on both experimental diets than at baseline, but the differences were significantly lower at the end of the walnut diet in comparison to the low fat diet.TC and LDL were slightly lower at the end of the walnut diet in comparison to the low fat diet but these differences were not significant. HDL was significantly higher than at baseline for both groups. | HIGH |
| Chisholm et al. (2005), New Zealand | RCT cross-over | Mixed nuts | 30 | 6 | N=28 (no reported loss to follow up). | Mean: 48.3 ± 10.3 | Mean: 26.9 ± 3.2 | Without familial or secondary hyperlipidaemia or were consuming lipid lowering medication. Healthy participants with moderately raised TC and LDL- C , n=23 females, n=5 males, free from major medical illnesses and not on medications known to influence lipids and lipoproteins. | Intervention group consumed 30 g of nuts (may have included almonds, Brazil nuts, cashews, hazelnuts, macadamia nuts, peanuts, pecans, pistachios, or walnuts – consumed raw or roasted at low heat without any additional oil). Control group consumed a serving of cereal (82 g) (rolled oats, wheat germ, barley flakes) containing canola oil (16 g fat). | Participants on nut diet instructed not to eat cereal containing added fats or oils and avoid canola oil/spreads. On cereal diet, to avoid nuts of any kind, including peanut butter, nuts spreads, sauces etc. | Compliance by weighing 4-day (3 weekday, 1 weekend day) food records were conducted by participants at beginning and end of each study arm to determine adherence and provide intake data. Blood lipids were assessed following an overnight fast at baseline and week 6. BP was measured using a sphygmomanometer after a 10 min rest. | There were no significant differences in TG between diet groups at the end of the intervention periods. LDL significantly lowered within both groups after study. | LOW |
| Choudhury et al. (2014), UK | RCT parallel | Almonds | 50 | 4 | n=75 healthy males. Group 1: n=20 healthy middle-aged men Group 2: n=20 healthy young men Group 3: n=20 'young, at risk' for cardiovascular disease men Group 4 (control): n=15 males, five subjects were randomly assigned to this group from groups 1-3. n=6 WD from group 4.  n=69 completed the study. | Group 1: 56.07 ± 5.8  Group 2:  22.14 ± 2.9 Group 3: 27.27 ± 5.04  Group 4: 23.00 ± 5.87 | Group 1: 25.37 ± 4.04 Group 2: 19.99 ± 1.595 Group 3: 26.34 ± 4.32 Group 4: 2.20 ± 2.260 | Group 1: Fasting (12 h) plasma TG <220 mg/dL, or TC<250 mg/dL. Group 2: Fasting (12 h) plasma TG <220 mg/dL, or TC<250 mg/dL. Group 3: At least one risk factor (lipids, BP and BMI) above the normal range. | Intervention group 1-3: substitute any daily snack for two bags of almonds (50 g) and otherwise to eat to satiety. Control group (Group 4) - Habitual diet (One in four of the subjects recruited to group 1, 2 and 3 were assigned to group 4. | Habitual diet. | Compliance and dietary intake with the intervention diet was assessed with 3-day food diaries, including a weekend day and bag return. TG and cholesterol concentrations were measured enzymatically with CHOD-PAP kits. HDL was separated from the plasma by precipitation with dextran sulphate and magnesium chloride and cholesterol was analysed using CHOD-PAP. BP measurements were taken twice from volunteers after sitting for 5 minutes using a manual sphygmomanometer. Blood flow measurements were obtained using venous occlusion plethysmography. | HDL and LDL and TG remained unchanged and were not affected by almond intake.  In groups 1-3, SBP reduced significantly after 50 g almonds/day for 4 weeks, but DBP reduced only in healthy men. Individual data of pre/post intervention/change between groups not provided as text (only figures).   A significant increase in FMD was observed after 50 g almonds per day for 4 weeks in the young groups and a trend for increase was observed in the older adults showing an improvement in blood flow. There was a trend for reduction in blood flow in the subjects who maintained a habitual diet (only figures). | LOW |
| Coates et al. (2020), Australia | RCT parallel | Almonds | 15% energy | 12 | n=151 adult men and women postmenopausal. n= 23 (n=7 did not receive allocated intervention. n=128 completed the study (78 F and 70 M). n=63 nut group and n=65 nut free group. ITT analysis. | 50 - 80  (I: 64 ± 8, C: 65 ± 8) | 25 - 39.9 (I: 30.3 ± 3.6, C: 30.5 ± 3.8) | Without diagnosis of diabetes, liver, gastrointestinal or cardiovascular disease, non-smokers, uncontrolled hypertension (SBP/DBP > 160/100 mmHg), neurological disorders or history of inflammatory brain disease, history of depression or anxiety disorders in past 2 years, cognitive impairment or memory loss (score <18 on the Montreal Cognitive Assessment (MOCA)), regular nut consumption (>30g per day), known hypersensitivity or allergy to nuts, were a restrained eater (score ≥12 on the three factor eating questionnaire), pregnant or breastfeeding, unstable medications in the past 3 months, taking vitamin supplements, herbal extracts or illicit drugs. | Intervention group consumed raw almonds. Control group consumed carbohydrate-rich snack foods (providing 15% energy) which were chosen nut and seed free. | Habitual diet | Compliance by returning any uneaten test foods so they could be weighed. Dietary intake was assessed based on a 4-day food record (1 weekend day). SBP and DBP and small (SAC) and large (LAC) arterial compliance were recorded using the Cardiovascular Profiler™. Fasting plasma lipids (TG, TC, HDL) were determined using a commercial assay kit. LDL was calculated using the Friedewald equation. | There were significant reductions in TG and SBP following the almond diet. There were no significant changes in TC, HDL, LDL, TC:HDL, DBP and small and large arterial compliance following the almond diet. | HIGH |
| Colquhoun et al. (1996), Australia | RCT cross-over | Macadamias | 50-100 (depending on energy requirement) | 4 | n=14 completed the study (n=7 males, n=7 females). n=20 were initially enrolled, however n=6 were excluded due to significant non-compliance with the dietary protocol. Data presented are for the n=14 completers (no intention to treat analysis performed). | 25 - 59 (Mean: 46.36 ± 10.44) | NR | Without taking lipid lowering medication. With healthy men and women of TC from 4-8 mmol/L. | Intervention group consumed a macadamia diet. Control group consumed a carbohydrate diet. | iso-energetic diet either high fat or high CHO. | Compliance by 3-day weighed food diaries weekly. Fasted blood samples were taken 3 times before the start of the study, during the pre-entry phase and on 2 occasions during the final week of each dietary period. Lipids were measured using standard analytical procedures. LDL was determined using the Friedewald equation. | For TC a decrease was observed between the pre-entry and macadamia nut or low fat diet . Consistent with this reduction, LDL decreased on both the macadamia and low total fat diets. The high CHO diet reduced HDL compared to baseline, however HDL was not significantly different to control with the macadamia diet. On the macadamia diet there was a reduction in fasting TG level while there was no change on the high carbohydrate diet. | LOW |
| Costa E Silva et al. (2020), Brazil | RCT parallel | Brazil nuts | 10 mL/day | 4 (+2days) | n= 41 male and female adults diagnosed with MetS, 20 brazil nut group and 21 soybean group. n=31 completed the study. | 36 - 65 (I: 58.5 ± 8.4, C: 58.3 ± 5.7) | I: 32.2 ± 4.4, C: 30.8 ± 4.4 | Without thyroid function alterations, neurodegenerative, or chronic liver diseases and not a smoker. | Intervention group consumed 10 mL/d Brazil nut oil. Control group consumed 10 mL/d soybean oil. | Habitual diet | Compliance and dietary intake NR. BP measured with the arm positioned and supported at the level of the heart and the palm of the hand facing upwards. TC, HDL and TG were analysed in a biochemical self-analyser (Dade AR®) using Dade Behring kits. LDL was determined according to the Friedwald equation. | There was no difference in SBP and DBP between the groups before and after the use of oils. Intervention group showed a significant decrease in HDL and a significant increase in TG. Intervention group showed a non-significant decrease in TC and LDL. | HIGH |
| Curb et al. (2000), US | RCT cross-over | Macadamias | 46 | 4 (+2days) | n=30 included in final analysis. n=42 participated in the run-in period, of these, n=16 men and n=18 women began the experimental diet period, of whom n=15 men and n=15 women completed the study (reasons for withdrawal not given). | 18 and 55  (Mean M: 36.7, F: 33.8) | M: 19.5 - 27.9 (Mean: 24 ± 2.4) F: 19.1 - 28.3 (Mean: 22 ± 2.6) | Without current pharmacological treatment for hyperlipidaemia, weighing between 80% and 130% of ideal weight, with no history of diabetes mellitus or pancreatic insufficiency, or an unstable medical condition of any kind, having no history of food allergies, especially to tree-grown nuts, and not pregnant, breastfeeding, or taking certain birth control pills. Men and women with a fasting cholesterol level above 3.9 mmol/L (150 mg/dL) and a TG level below 4.5 mmol/L (400 mg/dL). | Intervention group consumed a macadamia nut-based MUFA diet (37% energy from fat) in random order. Control group were fed a typical American diet (37% energy from fat), An American Heart Association Step 1 diet (30% energy from fat).  Finely ground macadamia nuts were used for the macadamia diet. | Subjects ate breakfast and dinner at metabolic kitchen each day and ate a prepared lunch off site. Subjects were allowed a ‘free’ meal on Saturday nights. | Compliance assessed by studying diary to record deviations from diet plan, physical activity, medication use etc. Body weight was measured by researchers on a digital scale Cholesterol levels were measured enzymatically (from fasted blood samples) using an autoanalyser. HDL cholesterol level were determined directly after manganese chloride–heparin precipitation. LDL was calculated using the Friedewald algorithm. Participants told to avoid alcohol in the 5 days preceding a blood sample. | Compared with the typical American diet, the mean TC level was significantly lower for the macadamia nut and AHA Step 1 diets. The mean low-density lipoprotein cholesterol level was also lower for these 2 experimental diets. Mean TG values were significantly higher than with the typical American diet for the Step 1 diet and significantly lower for the macadamia nut diet. The mean HDL level was lower after the Step 1 and the macadamia nut diets. When men and women were compared, lipid profile trends were not statistically different. | HIGH |
| Damasceno et al. (2011), Spain | RCT cross-over | Almonds and walnuts | 40 to 65 (walnuts) or 50–75 (almonds) (22% energy) | 4 | n=18 completed the study.  n=20 participants randomised. n=2 WD due to the demanding nature of the intervention. | 25-75 (Mean: 56 ± 13) | 25.7 ± 2.3 | Without chronic illnesses or secondary hypercholesterolemia, no known nut allergy, those who did not take vitamin supplements, HRT, or medications known to affect lipid metabolism. Asymptomatic men and women with moderate hypercholesterolemia (post menopause in F), LDL ≥3.36 mmol/L, TG ≤2.82 mmol/L. | Intervention group 1 consumed Spanish grown walnuts (40-65 g/d, varied according to participant’s total energy requirements- ~40% of total fat, 22% of total energy) partially replaced olive oil and other MUFA-rich foods (eg olives and avocados).  Intervention group 2 consumed raw, shelled Spanish almonds (50-75 g/d, varied according to participant’s total energy requirements, ~40% of total fat, 22% of total energy). No nuts other than those prescribed in nut diets were allowed during the study. Control group consumed virgin olive oil (35-50 g/d,~40% of total fat, 22% of total energy) instead of other refined olive oil. | A Mediterranean type diet. | Compliance was assessed by 3-day diet recalls. SBP and DBP were measured using standard methods by researchers and serum lipids (TC, LDL- C, HDL, TG) were measured at baseline and with completion of each arm of the study fasted. Cholesterol and TGs were measured using enzymatic procedures. HDL was quantified after precipitation with phosphotungstic acid and MgCl. Apo A1 and B and lipoprotein(a) were determined by using turbidimetry. LDL was isolated from plasma by preparative sequential ultracentrifugation using a Sorvall ultracentrifuge . | LDL was significantly lower within groups over time, but didn’t differ across groups. The intervention had no significant effect on HDL. No significant effect on BP. Supplementation of almonds or walnuts on a Mediterranean weight maintenance diet had a similar effect on body weight and blood lipids to a Mediterranean diet supplemented with virgin olive oil. | HIGH |
| Damavandi et al. (2013), Iran | RCT parallel | Hazelnuts | 10% energy | 8 | n=50 recruited (n=16 men, n=34 women) n= 48 (n=33 women and n= 15 men) completed the study (n=2 WD due to personal reasons. Control group (n=23) and intervention group (n=25). | Mean: 55.68 ± 7.74 | ≤35 | With type 2 diabetes, adult male and females all taking oral hypoglycaemic agents, serum TGs <400 mg/dl, and HbA1C <9%. | Intervention group with 10% of total daily energy intake was replaced with hazelnuts. Control group followed own self-selected diet). | Usual dietary pattern. | Compliance assessed by 24-h dietary recall (2 weekdays and 1 weekend day) at baseline and at weeks 4 and 8 of the study.  Fasted venous blood samples were obtained at the start and at the end of the study period to measure TC, TG, LDL, and HDL concentrations. | No effect was seen for TC, TG or HDL. But after 8 weeks, significant differences were observed between hazelnut group and control group in HDL levels, using ANCOVA, with dietary fat and carbohydrate intake as confounding factors (specific data not provided). | HIGH |
| Damavandi et al. (2019), Iran | RCT parallel | Cashews | 10% calorie | 8 | n=50 male and female patients with type 2 diabetes, 25 per group. n=7 WD. n=43 completed the study. | 30 - 75 (Mean: 53.86 ± 7.22) | < 35  (C: 28.6 ± 3.1, I: 28.7 ± 5.8) | With fasting blood glucose ≥ 126 mg/dL, two-hour plasma glucose (2h-PG) ≥ 200 mg/dL, HbA1C 7% - 9%, serum LDL < 200 mg/dL, TG < 400 mg/dL, and BP ≤ 160/90 mmHg. Without allergy to nuts, insulin therapy, smoking, history of stroke, heart diseases or thyroid dysfunction, nephropathy or retinopathy, medications that could affect body weight and/or energy expenditure, consumption of nuts more than twice/week, vegetarians, and weight-loss diets up to two months before the enrolment in the study. | Intervention group was asked to substitute 10% of their total calorie intake with unsalted cashews (~28 g/d, based on baseline calculated energy intake). Control group were advised to not change current dietary pattern. | Usual dietary pattern. | Compliance was monitored once a week by telephone call and double checked by the three day food record. Dietary intake by 24-hour dietary recall forms were completed in three day. Lipid profile (TC, TG, LDL and HDL) were measured by an enzymatic method. | TC/HDL and LDL/HDL were significantly lower in the cashews than the control group at the end but were not significant between the two studied groups. TG/HDL were not significant at end of trial or between groups. | HIGH |
| de Souza et al. (2018), China | RCT parallel | Almonds | 20 (15 units) | 8 | n=60 overweight and obese adult women, 30 per group.  n=14 loss to follow-up or WD. n=46 completed the study, n=24 nut group and n=22 nut free group. | 20 - 59 | 24.9 - 40 | Without food restriction or weight loss diet, acute disease that requires treatment, chronic immune disease, food allergy, thyroid disease, pregnancy or plans to become pregnant, gastrointestinal surgery, hormone placement therapy, antibiotic treatment, or treatment with any drug known to affect immune response or lipid metabolism, chronic alcoholism and those who used insulin or nutritional supplements. | Intervention group consumed 20 g (15 units) of roasted baru almonds. Control group consumed a daily supplement consisting of 800 mg maltodextrin in a sachet. | Individualised normocaloric and isoenergetic diet. | Compliance was assessed weekly via telephone calls and during routine monthly consultations when the supplements were provided. Adherence to the intervention was assessed with food records and counting the empty packages that remained when the participants returned to the laboratory. Dietary intake by a 24-h recall instrument and three dietary intake registers (2 weekdays and 1 weekend day). TC, HDL, TG were determined by automated enzymatic methods on a System VitrosChemistry 950 Xrl automatic analyser. LDL concentrations were calculated using the Friedewald equation. Apo A1 and apo B were measured in duplicate using multiplex immunoassay kits. | Intervention group showed significant reductions in TC, LDL and TG between baseline and endpoint but not between groups. The intervention group showed a significant increase in HDL and a trend reduction in apo B compared with the changes of these parameters in the control (nut free) group. There were no changes in levels of apo A1 and apo B did not change during the intervention in either group. | HIGH |
| Deon et al. (2018), Australia | RCT parallel | Hazelnuts | 15-30 (0.43 g/kg of body weight) | 8 | n=66 children and adolescents with primary hyperlipidaemia (31 F and 35 M).  n=6 WD due to personal reasons.  n=60 completed the study (26 F and 34 M), n=22 hazelnut with skin, n=20 hazelnut without skin and n=18 control. | 6.7 - 17.5 (Mean: 11.6 ± 2.6) | C: 0.25 ± 1.11, I1: 0.46 ± 1.36 I2: 0.12 ± 1.14 | Without food allergies or specific aversion for nut consumption, secondary forms of hyperlipidaemia, obesity, defined as a BMI 97th percentile, for age and sex (to exclude a confounding variable), chronic diseases requiring medical treatment, smoking habit, treatment with lipid-lowering treatment or functional foods in the previous 3 months. | Two intervention arms consumed roasted hazelnuts with skin and hazelnuts without skin in pre-weighed vacuum packed portions. The amount of hazelnuts per portion was calculated based on the doses advised to adults, adjusted on children body weight (0.43 g/kg of body weight on average, corresponding to 15-30 g portions) Control group consumed a nut-free diet. | Nutritional recommendations based on the cardiovascular health integrated lifestyle diet (CHILD1) guidelines. Diet | Compliance by collecting any uneaten hazelnut package at the last visit. Compliance was assessed by weighing the eventual packages returned, and by analysing weekly food diaries. Dietary intake weekly food diaries, before and after enrolment in the study, and were periodically interviewed for the duration of the study. Serum levels of TC, HDL and TG were directly determined by an automatic biochemical analyser. LDL concentration was estimated using the Friedewald formula. | Both hazelnut with skin and hazelnut without skin groups had significant decreases in LDL and HDL/LDL ratio when compared to baseline. All plasma lipids had a non significant difference between groups. | HIGH |
| Dhillon et al. (2016), US | RCT parallel | Almonds | 15% energy | 12 | n=86 healthy adults overweight and obese (21 men and 65 women), 43 per group. n=7 lost to follow up. n=50 compliers analysis. | 18 - 60 | 25 - 40 | Without nut allergies, willingness to consume almonds, not taking medications known to influence metabolism and appetite, nonsmoker for >1 y, consistent diet and activity patterns, and weight-stable (<3 kg change over the last 3 months). | Intervention group consumed almonds providing 15% energy. The almonds were dry-roasted and lightly salted. Control group were asked to avoid all nuts, seeds, and nut products. | Individualized energy-restricted diet. | Compliance with energy restriction (regardless of group) was assessed via self-reported intake (24-h food recalls) and weight loss. Lipids (TC, LDL, HDL and TGs) were analysed with the use of a COBAS INTEGRA 400 Plus analyser. Resting BP was assessed with the use of an automated digital BP monitor. | There were no changes in serum TGs, TC, HDL, and LDL with almond consumption. | HIGH |
| Dhillon et al. (2018), US | RCT parallel | Almonds | 56.7 | 8 | n=80 healthy college first years (41 women and 32 men), 40 per group.  n=7 WD prior to intervention.  n=73 completed the study. | 18 - 19 | 18 - 41  (I: 25.6 ± 5.0 and C:  25.3 ± 4.5 | Without nut allergies, willingness to consume almonds or graham crackers, willingness to maintain consistent diet and activity patterns, not taking medications known to influence metabolism and appetite, and non-smoker over the previous year. Without diabetes or pre-diabetes, uncontrolled hypertension, cardiovascular disease or dyslipidemia requiring drug therapy. | Intervention group consumed 56.7 g/d of dry-roasted almonds. Control group consumed 5 sheets (77.5 g/d) of graham crackers. Both groups were asked to avoid consumption of other nuts and seeds. | NR. | Compliance by participants meeting with researchers every day before 12 pm over the study duration (except weekends and 5-day spring break) to collect and consume their study snacks. Peer-researchers witnessed and recorded participants’ consumption of assigned snack. Dietary intake by a validated, automated, and self-administered 24-h Dietary Assessment Tool (ASA24). Serum lipid samples (TC, HDL, LDL and TG) were analysed using the Olympus AU400. Resting BP was assessed using an automated digital BP monitor. Reactive Hyperemia Index (RHI), a measure of endothelial function, and augmentation index (AI), a measure of arterial stiffness, were assessed using an Endopat device. | Resting SBP, DBP and mean arterial pressure were not significantly different between baseline, week 4 and week 8. There was no difference in any of the BP outcomes or RHI and AI between the almond and cracker groups at the end of the 8-week intervention. Fasting serum TC and HDL significantly decreased from baseline to week 8 and week 4 to week 8 of the intervention. LDL progressively significantly decreased from baseline to week 4 to week 8. TG marginally significantly decreased from week 4 to week 8, though no overall difference over the 8-week intervention. | HIGH |
| Dikariyanto et al. (2020), UK | RCT parallel | Almonds | 20% energy | 6 | n =107 adult men and women with above-average risk of developing cardiovascular disease. n=2 WD. n=105 completed the study (n=51 CG and n=54 IG).  n=101 in analysis. | 30 - 70 (I:56.3 ± 10.3, C: 56.0 ± 10.7) | 18 - 40 (I:27.3 ± 4.4, C: 26.7 ± 4.5) | Without diabetic condition or pre-diabetic, history of a heart attack, stroke or cancer, epilepsy or regular fainting, cholestatic liver diseases, pancreatitis, alcohol or drug abuse; diagnosis of cardiovascular problems, angina, thrombosis, pacemaker, gastrointestinal disorders, renal or bowel diseases; currently pregnant, planning pregnancy, breastfeeding or given birth in the preceding 12-months; and use of a drug likely to alter gastrointestinal motility or nutrient absorption. | Intervention group consumed almond snacks were dry-roasted whole nonsalted almonds of the nonpareil variety. Control group consumed sweet and savory mini-muffins were baked at the study center. | Habitual diet. | Compliance and dietary intake by telephone 24-h recalls and 4-d estimated portion size food records. Endothelial function was assessed as EDV of the brachial artery using the FMD technique by ultrasound. Ambulatory blood pressure (ABP) was measured using TM- 2430 ABP monitors worn for 24 h. Analysis software was used to analyse mean 24-h, daytime, and night-time SBP, DBP, and pulse. A small, lightweight, chest-worn, wireless 2-lead ambulatory heart rate/ECG monitor was fitted to measure 24-h ambulatory HRV. Plasma lipids [TC, HDL, LDL, TG, and calculated TC:HDL ratio] were analysed using venous blood samples taken at baseline and end point. | Almond consumption significantly increased FMD by 4.1% units relative to the change following control snacks. There were no significant group differences observed in changes in BP or in 24-h HRV. LDL concentrations were significantly reduced by almond snacks relative to control. There were no significant treatment effects on SBP, DBP, TC, HDL, TG, and TC:HDL. | HIGH |
| Din et al. (2011), Scotland | RCT cross-over | walnuts | 15 | 4 | N=30 (no mention of. | Mean: 23 ± 3 | 24.5 ± 2.3 | Without taking regular medications, those with clinical evidence of atherosclerotic vascular disease, hypertension, diabetes mellitus, hypercholesterolaemia, an intercurrent illness likely associated with acute phase inflammatory response, and renal or hepatic insufficiency. Young healthy males with normal BMI, BP and lipid profile, alcohol intake was within recommended limits (15 ± 10 units/week). | Intervention group consumed walnut supplementation (15 g/d) for 4 weeks. Control with no walnuts continue with habitual diet for 4 weeks. | Habitual diet. | Compliance monitored by a 3-day weighed food diary during each phase of the study. BP was measured using an automated upper arm BP monitor, blood lipids were assessed using fasted blood samples. Arterial stiffness was measured non-invasively with the SphygmoCor system. Peripheral pressure waveforms were obtained using applanation tonometry of the radial artery with a pressure-sensitive micromanometer (Millar Instruments, Houston, TX, USA) | Dietary intervention with walnuts did not affect heart rate, peripheral BP or central aortic pressures compared with control. There were no differences observed in augmentation index or augmented pressure | HIGH |
| Dusanov et al. (2020), Norway | RCT parallel | Mixed nuts | 28.6 (~200 g/week) | 26 | n=131 overweight and obese participants (56 men and 75 women). n=11 loss to follow-up. n= 120 completed the study. n= 131 ITT analysis. | 37- 69 | 25.5 - 38.2 | Without cigarette smoking, diabetes, allergy to or dislike offish or nuts, chronic disease including cancer, gastrointestinal disease or cardiovascular disease, morbid obesity and self-reported weight fluctuations, eating disorder, history of bariatric surgery, use of anti-obesity drugs or other drugs affecting body weight, such as anty-psychotics or glucocorticoids. | Intervention group consumed ~100 g walnuts, ~50 g hazelnuts and ~50 g almonds weekly, also providing ~1400 kcal/week. Control group consumed their usual diet, but were asked to avoid fatty fish and nuts. | Habitual diet. | Compliance were done at each visit by dietitian/responsible physician by direct query as to whether all of the assigned food had been consumed. Dietary intake by FFQ. Lipids were measured using enzymatic colorimetric methods, while apo B was determined using an immunoturbidimetric method. | SBP and DBP and heart rate remained unchanged and did not differ between the 3 groups. From baseline to 6 months there was no change in TC, HDL, LDL and apo B for the nut group. From baseline to 6 months there was non-significant increase in TG. | HIGH |
| Eastman and Clayshulte (2005), US | RCT parallel | Pecans | 68 | 8 | n=17 final analysis (completed the study). n=8 WD/data not included as they were not able to comply with the study protocols. | Mean I: 46±6,  C: 53 ± 10 | I: 27 ± 4  C: 27 ± 3 | Without an active disease, and not being pregnant or lactating, had current or history of drug or alcohol abuse, food allergy to nuts and were not taking lipid lowering medications. With hyperlipidaemia (adults). | Intervention group consumed 68 g of pecans/day. Control group avoided nut consumption. | Self selected dietary intake. | Compliance with dietary protocol was monitored by interviewing subjects at each study visit, reviewing food records and for pecan treatment group, inspecting pecan ration boxes (each subject recorded 3-day food diaries). Blood analytical procedures were certified by the Northwest Lipid Research Laboratories (Seattle, Washington) with traceability to the National Reference System for Cholesterol | No differences between the groups post intervention in blood lipids | HIGH |
| Fatahi et al. (2019), Iran | RCT parallel | Walnuts | I1: 10.2 (18 walnuts/week) I2: 5.14 (9 walnuts/week) | 12 | n=99 overweight and obese women, 33 per group.  n=0 loss to follow-up. | 20 - 50 (Mean: 53.5 ± 1.6) | 33.29 ± 5.63 | Without being pregnant, lactating, or with a history of renal disorders, type 1 or 2 diabetes, elevated BP, cardiovascular diseases, allergic reactions to fish or walnut, receiving agents lowering blood glucose or lipids. | Intervention group 1 consumed 6 walnuts 3 times a week (18 walnuts/week) and avoid the consumption of fish.  Intervention group 2 consumed 150 g of fatty fish and nine walnuts per week. Control group consumed 150 g of fatty fish (salmon or trout) twice per week (300 g/week). | Low-calorie diet. | Compliance and dietary intake by dietary records. Serum concentrations of HDL, LDL and TG were quantified using commercially available enzymatic reagents adapted to an autoanalyser system. SBP and DBP were measured twice after 15 minutes of resting in a seated position using a standard mercury sphygmomanometer. The mean of the 2 measured SBP and DBP was reported. | The reduction in SBP and LDL, and the increase in HDL were statistically higher in the group randomised to the fish + walnut diet (group 2) compared with the walnut group (group 1) or the fish group (group 3). A significant decrease was seen in TG and DBP levels in the fish group (group 3) and the walnut group (group 1) compared with the fish + walnut group (group 2). | HIGH |
| Foster et al. (2012), US | RCT parallel | Almonds | 56 | 78 | n=123 (n=112 women, n=11 men). n=61 were randomised to almond enriched diet.  n=14 lost to follow up (n=2 cited time constraints, n=1 cited dissatisfaction with program, n=1 cited life stressors, n=10 no reason provided).  n=62 were randomised to the nut free diet. n=17 lost to follow up (n=1 cited time constraints, n=1 dissatisfied with diet,  n=5 cited life stressors, n=1 pregnancy, n=1 relocation, n=8 no reason provided). | 18-75 (Mean: 46.8 ± 12.4) | 27-40 (Mean: 34.0 ± 3.6) | With overweight and obesity without uncontrolled Hypertension, cardiovascular disease or an inflammatory condition, diabetes or use of antihyperglycaemic medications, dyslipidaemia requiring prescription drug therapy or any known allergy or sensitivity to nuts anduse of medications, dyslipidaemia requiring prescription drug therapy or any known allergy or sensitivity to nuts. | Intervention group consumed a hypocaloric, almond enriched diet and the control group consumed a hypocaloric, nut-free diet.   Hypocaloric diet =1200-1500 kcal/day for female participants, 1500-1800 kcal/day for male participants subject to individual requirements. | Habitual diet | No data on compliance or overall dietary intake. BP assessed seated using automated BP monitor. Plasma lipids and lipoproteins were assessed via blood samples taken from participants following an overnight fast. | The intervention compared with the control was associated with greater reductions in TC and TGs but no differences were observed between the groups. | HIGH |
| Ghanavati et al. (2021 a) Ghanavati et al. (2021 b), Iran | RCT parallel | Mixed nuts | 39-60 (20% energy) | 8 | n= 70 stable coronary artery disease patients who underwent percutaneous coronary intervention more than 6 months ago, 35 per group. n=3 WD. n=67 completed the study. | 58.8 ± 7.4 | >25 (30.9 ± 3.9) | Without an allergy to nuts, did not have chronic kidney disease stage 4 and 5 and had the readiness to follow a low calorie diet for weight loss. | Intervention group consumed nut-enriched low calorie diet. Mixed nuts were non-salted roasted pistachios, almonds, and peanuts, the amount of nuts in each patient’s diet was determined based on 20% of the calculated energy needs. Control group consumed a low calorie diet. | Individualised low-calorie diet. | Compliance by subjects returning empty or unconsumed packages. Dietary intake by food recalls. Plasma concentrations of ICAM-1 were measured by enzyme-linked immunosorbent assays kits. | Participants in the nuts enriched group showed a significant decrease in ICAM-1 concentrations, and the treatment effects were significantly different from those in the control group. | HIGH |
| Gozde et al. (2019), Turkey | RCT parallel | Walnuts | 40 | 6 | n=39 moderate hyperlipidemic subjects, 22 allocated to intervention and 17 allocated to control group. n= 2 loss to follow up or dropped out.  n=37 completed intervention. | 37 - 52 | NR | Without TGs >300 mg/dL and TC >500 mg/dL; were not obese, smokers or frequent alcohol users, free of dietary restriction/food allergies and not taking medications known to alter plasma lipids. Patients who have other health problem rather than coronary heart disease did not include in the study. | Intervention group consumed 40 g of walnuts as snacks once a day. Control group consumed 4 placebo capsules containing 1.25 CC distilled water, three times daily with food for 90 days. | American heart association (AHA) low-fat and low-cholesterol diet AHA diet. | Compliance by visiting participants once in every 15 days and in order to follow their nutritional status. Dietary intake by 3-day consecutive food records. TC was measured by cholesterol esterase, cholesterol oxidase and peroxidase reactions. Method for direct determination of HDL uses polyethylene glycol (‘PEG’) based system in which sulfated a-cyclodextrin, dextran sulfate and MgCl2 form water soluble complexes with the non-HDL lipoproteins present in a sample, after which pegylated cholesterol esterase and cholesterol oxidase are introduced. LDL concentration were calculated using the Friedewald formula. | Compared with baseline, the intervention diet favourably decreased the concentrations of TC and LDL. while there were no significant differences found on HDL concentrations and TG levels in both groups. There was no statistical significant difference found in TC/ HDL in intervention group, it was appeared a decreasing trend for the parameter. In addition, the intervention diet favourably altered the ratio of LDL/HDL cholesterol compared with the control diet. | HIGH |
| Griel et al (2008), US | RCT cross-over | Macadamias | 42.5 | 5 | n=25, (10M, 15F) enrolled.  n=1 participant WD following completion of the first diet sequence due to time constraints. | 50.2 ± 8.4 | 22-35 (Mean: 26.3 ± 3.3) | With moderately hypercholesterolaemic subjects who were reasonably healthy with no other major co-morbidities. Non- smoker, LDL 25 -90th percentile NHANES, not on lipid-lowering medications or other medications known to affect lipids. Subjects were representative of the population in the United States at high risk for cardiovascular disease. | Intervention group consumed macadamia nut diet (roasted, half salted) and the control group consumed average American diet (matched for macronutrients, designed for weight maintenance). | Subjects consumed either breakfast or dinner at the study centre Mon-Fri. Lunches & weekend meals were prepared or packed for off-site consumption. | Compliance of diet, physical activity levels and medication changes were monitored by staff and by review of daily and weekly monitoring forms. 12 hour blood samples taken from fasting subjects on 2 consecutive days at baseline and the end of each diet period to evaluate blood lipids. TC, TG determined using enzymatic assays. HDL was estimated according to the modified heparin-manganese precipitate procedure. | The macadamia diet was significantly different to American diet for TC to HDL ratio.  The macadamia diet showed significant difference from the baseline for TC, LDL, and HDL. | HIGH |
| Guarneiri et al. (2021), US | RCT parallel | Pecans | 68 | 8 | n=56 were assigned to an intervention.  C: n = 20.  I1 (n = 17). I2 (n = 19).  n=4 WD. | 30 and 75 | ≥28 | Without familial hypercholesterolemia, habitual nut consumption (56 gram/week), nut allergies, special diets (i.e., ketogenic diet, intermittent fasting), excessive alcohol use, tobacco or nicotine use, exercise >3 hour/week, weight loss or gain >5% of body weight in the past 3 months, plans to begin a weight loss or exercise regimen, history of medical events or medication use affecting digestion, absorption, or metabolism, gastrointestinal surgery, and chronic or metabolic diseases. Without medications that could affect blood lipid or glycemic, fasting glucose. | Control consumed no-nuts. Intervention 1 diet provided with 68 g (∼0.5-cup or 2.25-oz) portions of pecans to consume as part of their free-living diet with no additional diet instructions. Intervention 2 group were instructed to substitute the 475 kcal provided by the 68 g of pecans for foods habitually consumed in their free-living diet. | Habitual diet | Compliance was categorized as consumption of <75% of pecans. All participants completed a food diary once per week alternating between weekdays and weekend days. A fasting blood drawn for a lipid panel and glucose measurement was obtained, and anthropometrics and resting metabolic rate. A portion of the fasting blood sample was drawn into a serum separator clot activator vacutainer. The rest of the fasting blood sample and all postprandial blood samples were drawn into an EDTA vacutainer. | With or without dietary isocaloric substitution instructions, resulted in significant improvements in fasting TC, LDL, TGs, HDL, TC/HDL ratio, non–HDL, and apo B. In addition, there were improvements in fasting NEFA and postprandial glucose or TGs in at least 1 of the 2 pecan groups. | HIGH |
| Gulati et al. (2014), India | RCT cross-over | Pistachios | 20% energy | 24 | n = 68 commenced study after run-in period. n=60 finished study, ITT used (pistachio group: n = 33, control group: n = 35). | 25 – 65 (Mean: 42.5) | 30.9 | Without type 2 diabetes, accelerated or uncontrolled hypertension, uncontrolled hypothyroidism or uncontrolled lipids. Only Asian Indian adults with MetS. | Intervention group consumed pistachios (20% of total energy) as part of diet in line with dietary guidelines for Asian Indians (51% carbohydrates, 20% protein, and 29% fat). Control diet was 60% carbohydrates, 15% protein, and 25% fat. | A standard diet in accordance with dietary guidelines. | Compliance assessed by food frequency questionnaire, 24 hour dietary recall, phone interviews. Participants also asked to bring empty pistachio packets to investigators as measures of compliance. Compliance checks conducted approximately 1/month. Lipid levels were assessed after an overnight fast and estimated using commercial kits. LDL was calculated according to Friedewald's equation. | Significant differences were found between pistachio and control group after 6 months for TC, LDL.  No significant effect was found for HDL and TG. | HIGH |
| Hernández-Alonso et al. (2014), Spain | RCT cross-over | Pistachios | 57 | 16 | n=54 community living men and women (29 M, 25 F) with a fasting plasma glucose levels between 100 and 125 mg/dL. n=5 WD. n=49 completed the study. | 25 - 65 | <35 | Without one of the following criteria: 1) diabetes or using oral antidiabetic drugs, 2) alcohol, tobacco, or drug abuse, 3) frequent consumption of nuts or known history of allergy to them, 4) use of plant sterols, psyllium, and fish oil supplements. | Intervention diet consumed a pistachio supplemented diet. Pistachio nuts were half roasted and salted. Control diet consumed energy from other fatty foods, mostly olive oil. | Provided dietary instructions, including bi-weekly menus and seasonal recipes according to the type of diet. | Compliance by counting the empty sachets of pistachio administered and measuring plasma lutein- zeaxanthin and g-tocopherol levels with a liquid chromatograph coupled to a 6490 QqQ/MS. Dietary intake was estimated using the mean of 3-day dietary records including two workdays and a weekend day. Serum lipid profiles (TC, HDL and TG) were determined using standard enzymatic automated methods. LDL was estimated using the Friedewald formula. BP was measured in the non- dominant arm, using a validated semi- automatic oscillometer. | Lipid profile did not change significantly between groups, LDL showed a nonsignificant reduction after pistachio intervention compared with the increase observed in the control diet period. SBP and DBP nonsignificant changes. | HIGH |
| Hiraoka-Yamamoto et al (2004), Japan | RCT cross-over | Macadamias | 20 | 3 | n=71 randomised and completed study. Macadamia nut bread (n=24), butter bread (n=23), and coconut bread (n=24). | 19 - 23 | C1: 21.0 - 0.4  C2: 19.9 - 0.5  I: 19.9 - 0.4 | Young healthy Japanese women. | Intervention group consumed bread contained 10g of macadamia nuts, control 1 consumed bread containing 10 g butter. Control group 2 consumed bread containing 10 g coconut. | NR. | Compliance to the bread was considered good based on percentages of prescribed bread consumed. Serum total, HDL and LDL, TGs and free fatty acids were measured by enzymatic methods. | Significant reductions in macadamia group over time found for Tc and LDL.  No significant effect on HDL, TG, and SBP. | LOW |
| Holligan et al. (2014)  West et al. (2012)  Gebauer et al. (2008), US | RCT cross-over | Pistachios | 1 serve: 32g-63g (10% energy) 2 serves: 63g-126g (20% energy) | 12 | n=28 adults with elevated LDL, M: 10 males, F: 18. n=1 withdraw due to an inability to comply with study protocols. n=27 completed the study. | 25 - 65 | 21- 35 | Without taking BP or cholesterol-lowering medication, use of nutritional supplements, pregnancy, weight loss ≥10% of body weight in the previous 6 months, vegetarian or weight-loss diets, and history of liver, kidney, autoimmune, or vascular disease. | Intervention group 1 consumed 10% energy from pistachios. Intervention group 2 consumed 20% of energy from pistachios. Control group consumed a low fat diet. | Typical American diet. | Compliance by questionnaires and plasma b-sitosterol levels increased dose dependently with the inclusion of pistachios, consistent with dietary approximations. Dietary intake by controlled-feeding design and all meals were provided to the participants. SBP and DBP were measured using an automated oscillometric device at 1 to 4 minute intervals as participants were seated with their arm at heart level. Serum HDL samples (apo B-depleted serum) were prepared from individual serum samples by precipitation of apo B containing lipoproteins using polyethylene glycol. FMD was measured as the maximum percentage of change in brachial artery diameter after hyperemia. | TC, LDL, and non-HDL were significantly lower with the 1 PD and 2 PD than with the control diet. TG, TC/HDL, and LDL/HDL were significantly lower with the 2 PD than with the control diet. No significant differences in HDL when comparing the pistachio diets with the control diet. No significant change in DBP for diet effect while SBP showed significant difference. TG:HDL ratio revealed a significant effect of diet, and a significant reduction was observed in this ratio following the pistachio diet v. the control diet; the pistachio diets minimised the increase in the TG:HDL ratio from baseline compared with the lower-fat control diet. Heart rate decreased to a greater extent after 2 servings per day versus the control diet; the response to 1 serving per day was intermediate and did not differ from the other diets. | HIGH |
| Holscher et al. (2018), US | RCT cross-over | Walnuts | 42 | 3 | n=18 participants, n=9 per group (10 men and 8 women).  n=0 WD. | 35.0 - 67.8 (53.1 ± 2.2) | 20.2 - 34.9 (Mean: 28.8 ± 0.9) | Without fasting glucose ≥126 mg/dL, BP ≥160/100 mm Hg, fasting total serum blood cholesterol ≥280 mg/dL, and fasting serum TGs ≥300 mg/dL. Were not smokers or allergic to walnuts; abused alcohol; /did not have kidney disease, liver disease, gout, hyperthyroidism, untreated or unstable hypothyroidism, certain cancers, gastrointestinal disease, pancreatic disease, diabetes requiring medication, unstable body weight over the past 12 months, malabsorption syndrome, or were women who were pregnant, lactating, or had given birth in the past 12 months. | Intervention group consumed base diet + walnuts. Control group consumed base diet. | Typical American diet. | Compliance by participants completing questionnaires daily to report any dietary deviations. Dietary intake NR, though participants consumed meals at a center under supervision of study staff or provided meals packed for carryout. Serum samples were assayed to assess TC, LDL, HDL and TGs using enzymatic procedures. | Serum TC concentrations were significantly lower and LDL concentrations were significantly lower after walnut consumption compared with the control. HDL and TG were unaffected. | HIGH |
| Holt et al. (2015), US | RCT parallel | Walnuts | I:40 C:5 | 4 | n=38 postmenopausal women. n=36 completed the study and n=30 included in final analysis (n=12 (5 g/d group). n=18 (40 g/d group)). | 50 - 70  (I: 60 ± 3.8,  C: 60 ± 5.6) | <35  (I: 25.1 ± 4.1,  C: 24.1 ± 2.7) | Without allergies to walnuts or other nuts, use of prescription medications except for treating hypothyroidism, use of dietary supplements except standard multi-vitamin/mineral formulas, cigarette smoking, and a history of cardiovascular disease or other chronic diseases. | Intervention group consumed 40 g of walnuts daily. Control group consumed 5 g of walnuts daily. | Habitual diet. | Compliance was monitored by a self-administered log. Food intake was assessed from the compliance log as well as two 3-day food records that included two weekdays and one weekend day, consecutively, that were completed before each study day visit. | No significant differences were observed after 4 weeks of walnut intake for fasting plasma concentrations of TC, HDL- C, LDL or TGs (data not shown). | HIGH |
| Huguenin et al. (2015), Brazil | RCT cross-over | Brazil nuts | 13 | 12 | n=125 males and females diagnosed. dyslipidemia and hypertension who had been taking medication for their condition for at least three months. n= 34 loss to follow-up. n=91 completed the study.. | >20 (Mean: 62.1 ± 9.3) | 28.8 ± 5.1 | Without food allergy to the Brazil nut; pregnancy or breastfeeding; undergoing in a low calorie diet, using dietary supplements containing antioxidant vitamins or minerals; using corticoid substances, and thyroid diseases, chronic renal failure, liver disease, cancer, rheumatic disease or systemic connective tissue disease. | Intervention groups consumed 13 g/d of partially defatted granulated Brazil nut. Control group consumed 10 g/d flavored cassava flour. | The guidelines for hypertension and dyslipidemia. | Compliance adherence to the consumption of the supplement was monitored using the valuation of plasma serum concentrations. Dietary intake: NR. SBP and DBP were measured in the supine position using a sphygmomanomete. Serum TG, TC, and HDL were performed by an automated method using commercial kits. LDL were calculated using the Friedewald formula. | No difference was present between diets, at the beginning or the ending periods for MAP, TC, TG and HDL. However, a significant increase of HDL can be observed in both intervention and control groups. | HIGH |
| Hwang et al. (2019), South Korea | RCT cross-over | Walnuts | 45 | 16 | n=119 adults with MetS. n= 35 loss to follow up, dropped out or excluded from analyses.  n= 84 completed the study. | 30-55 | ≤35 | Without use of any medications to control BP, lipid metabolism, or glucose; consumption of a hypocaloric diet within the past year and regular consumption of omega-3 supplements; and post-menopausal women and smokers. | Intervention group consumed 45g of walnut/day, as convenient during the daytime. Control group consumed white bread/day, as convenient during the daytime. | NR. | Compliance by a self-reported consumption log sheet. Dietary intake by three-day dietary records that included two consecutive weekdays and one weekend day. Serum TC and TG levels were measured by using an enzymatic-colorimetric method, HDL and LDL levels were determined via a homogeneous enzymatic-colorimetry method. BP was measured on the right arm using an up-load BP monitor. Serum apo B was measured by turbidimetric immunoassay using a HITACHI 7600 chemistry analyser. | A significant intervention effects on HDL compared to the control group after applying linear mixed model adjustments for age, gender, BMI, and sequence. The HDL level was increased after walnut intervention but was stable after control intervention. SBP, TG, TC and apo B were significantly reduced after walnut intake, although there was no significant intervention effects compared to those after the control. | HIGH |
| Iwamoto et al. (2002), Japan | RCT cross-over | Walnuts | 44-58 | 4 | n = 40 randomised and completed the study. | Women: 23.6 (1.1)  Men: 23.8 (0.7) | Women: 20.7 (0.5)  Men: 22.2 (0.5) | Without a history of hypertension or atherosclerotic or metabolic disease, or taking medications regularly or considered unable to comply with the study protocol. Excluded if they ate nuts, had nut allergies, and smoked cigarettes. | The 2 experimental diets were identical except that the intervention diet substituted two servings of walnuts per day (25 or 27 g per serving, or 52 g of walnuts per 10.0 MJ) (stated to range from 44 – 58 g/d), for portions of some foods in the reference diet. | Average Japanese diet (reference diet). | Dietary compliance was assessed by tray checks at the meals eaten on site and by self-reporting on standardized forms for the packed meals. Subjects were also requested to record in diaries any signs of illness, medications used and any deviations from their experimental diet. Fasted blood samples were taken at the end of the run-in period and at the end of each dietary period. Serum lipoprotein subfractions were analysed to measure levels of cholesterol and TGs using enzymatic kits. HDL was measured using a commercial HDL kit and LDL was determined with the friedewald calculation. Concentrations of apo A1 and apo B were determined with modified commercial turbidimetric assays. BP was measured at the end of the run-in period and twice during each diet period after 5min of resting by trained physicians. | Significant effects for walnut diet compared to control (stronger in females than males) for: TC, LDL, LDL:HDL.  No significant effect on: HDL and TG. | HIGH |
| Jaceldo-Siegl et al. (2011)   Fraser et al. (2002), US | RCT cross-over | Almonds | ~52 (15% energy) | 26 | n=100 participants were enrolled and n = 81 completed the study (n=19 WD). | 25 - 70 | M: 26.7 ± 3.56, F: 25.9 ± 5.54 | Non smokers, did not drink more than two alcoholic drinks per day, had not experienced more than 9 kg weight change in the 6 months leading up to the study, were not heavy exercisers and were not eating nuts more than twice weekly. | For the first six months of the study, participants were not given any dietary advice or provided with any food.  For the second six months, participants were provided with an amount of raw or dry-roasted (participants could choose) almonds to account for 15% of daily energy. This corresponded to 54.3 g on average. | NA. | Compliance assessed by 24 hour dietary recalls (seven collected during first six month period), two one day diet diaries. Blood was collected after a 12 hour fast, and serum concentrations of TGs, TC, LDL and HDL. | A significant difference in the LDL:HDL ratio between the almond and control diets was found. No significant differences were found for TC, LDL and TG. | LOW |
| Jamshed et al. (2015),  Pakistan | RCT parallel | Almonds | 10 (~7 almonds/day) | 12 | n=150 CAD patients with optimal LDL and low HDL, n=50 per group.  n=37 loss to follow up. n=113 completed the study. | 32 - 86 | NR | Without presenting with acute myocardial infarction in the previous 5 week, regular nut consumers (>15 g/d; 3 days/week), and/or patients with a nut allergy. | Intervention group consumed 10 g/d of either American almonds or Pakistani almonds soaked overnight and eat them after removing the skin, before breakfast (presumably on an empty stomach). Control group were not given almonds and were instructed not to consume other nuts during their enrollment in the trial. | NR. | Compliance was monitored through regular phone calls (2 times/week). Patient diaries were also provided to record daily almond consumption. Concentrations of TC, TGs, and LDL and HDL were measured with the use of a c-111 automated analyser. TC:HDL and LDL-to-HDL ratios were calculated. | The almond interventions significantly increased serum HDL concentrations compared with their respective baseline concentrations. The almond interventions caused a significant decrease in serum lipid concentrations (TGs, TC and LDL), at both week 6 and week 12 when compared with control. Similarly, the almond interventions significantly decreased TC:HDL and LDL:HDL cholesterol ratios at weeks 6 and 12, compared with their respective baseline values. SBP and DBP and body weight remained fairly constant in the 3 groups over time. | HIGH |
| Jenkins et al (2002), Canada | RCT cross-over | Almonds | I1: 73, I2:37 | 4 | n=43 subjects started study. n=16 WD during or after end of 1-2 study phases.  n=27 completed all phases. | 64 ± 9 | 20.5 to 31.5 (Mean: 5.7 ± 3.0) | Healthy hyperlipidemic men and post-menopausal women, LDL of 4.32 ± 0.63 mmol/L (range 2.77 to 5.32 mmol/L). All subjects had elevated LDL levels on initial assessment at recruitment (>4.1 mmol/L) and TG concentrations <4.0 mmol/L. None had clinical or biochemical evidence of diabetes, liver, or renal disease. | Intervention group 1 consumed almond (full-dose), Intervention group 2 consumed almond (half-dose) + muffin and the control group consumed muffin. | Self-selected low-fat therapeutic diets or NCEP Step II diet. | Compliance to supplements and diet assessed by 7-day food record, a supplement checklist on which subjects recorded supplements consumed and return of uneaten supplements which were weighed and recorded. Fasting blood samples, weight, and BP measured at start and week 2 and 4 of each 4 week diet phase. Expired air was collected through a modified Haldane-Priestly tube for NO measurement at week 0 and 4 of each phase. | Small changes in body weight with each treatment. Significant differences found for: TC, LDL, HDL, LDL:HDL No significant effect found for: TG, SBP, DBP, Lp(a) decreased with full-dose almonds No treatment differences in C-reactive protein, BP or pulmonary NO. Cardiovascular disease risk for full dose of almonds was significantly reduced compared with baseline. | HIGH |
| Jenkins et al. (2018), Canada | RCT parallel | Mixed nuts | I1:75, I2:37.5 | 12 | n= 117 men or postmenopausal women with type 2 diabetes, n=40 full-dose, n=38 half-dose and n=39 muffin-dose. n=14 WD/dropped out. n=103 completed the study. | I (full-nut): 63 ± 8.9, I (half-nut): 61 ± 7.9, C: 61 ± 9.9 | I (full-nut): 28.8 ± 4.5, I (half-nut): 30.3 ± 5.0, C: 29.4 ± 4.2 | Without clinically significant cardiovascular, renal or liver disease (alanine aminotransferase >3 times the upper limit of normal), a history of cancer or were on warfarin. | Intervention group 1 consumed (Full-nut): 75 g/d mixed nuts, Intervention group 2 consumed (half-nut): half portions of both nuts and muffins, and Control group consumed three whole-wheat muffins/day (188 g/d). | General dietary advice conformed to the National Cholesterol Education Program Adult Treatment Panel III. | Compliance assessed by last 7 day diet record. Dietary intake by 7 day record at each visit. TC, TG and HDL were measured on a Roche Cobas 6000 c501 Analyser. LDL calculated by the Friedewald equation. Apo A1 and apo B were measured by nephelometry with the Siemens BN ProSpec analyser. | Compared with the control diet, the change in apo B with the full-dose nut diet was significantly different. In the unadjusted model, there was a significant reduction in TC and LDL with the full-dose nut diet vs the control diet. In the dose–response regression analysis, a single unit increase in nut dose reduced TC, LDL, and apo B. | HIGH |
| Jia et al. 2006, China | RCT parallel | Almonds | 84 or 168 | 4 | n=30 healthy male smokers from an army unit completed the study, 10 per group based on their duration of smoking and number of cigarettes consumed daily and age. | 18 - 25 | NR | Without a known history of gastrointestinal, liver or kidney disease. | Intervention group 1 and group 2 consumed 28 g and 56 g of almond powder at each of the 3 meal/day, respectively. Control group did not consume almonds. | All subjects consumed the same diet from the same canteen. | Compliance by daily diary. General information and dietary information collected using self-administrated questionnaires (unclear if validated, no inclusion of nutrition data). Fasted blood samples assessed TGs and TC using various analyses. | No significant difference between almond intervention groups and control group for TC or TGs (P value not provided). | LOW |
| Johnston et al. (2017), US | RCT parallel | Almonds | 70 | 3 | n=15 middle aged sedentary adults with at least one cardiovascular disease mortality risk factor.  n=3 WD. n=12 completed the study, 6 per group. | I: 55.5 ± 8.6, C: 52.7 ± 10.9 (Males: 45 - 60, post- menopausal women up to 60 years) | I: 28.0 ± 4.0, C: 25.6 ± 1.8 | Without active chronic disease. Individuals who reported nut, gluten, or other food allergies; cigarette use within the past year; or specific medication use (nitrate vasoactive hypertensive medications, nitroglycerin, beta-blockers, and calcium channel blockers). | Intervention group consumed 70 g/d raw almonds. Control group consumed 4 tablespoon/day cookie butter. | Habitual diet. | Compliance by plasma concentrations of alpha-tocopherol. Dietary intake by 2 x 3day food record. FMD and BP measures were conducted at baseline and trial weeks 5 and 8 by a certified sonographer. Blood sample were collected. | The additive effect of the walking intervention with almond ingestion revealed a significant difference between groups for TC. There was also a significant difference between groups for LDL. The combined walking intervention and almond ingestion was not related to SBP, DBP, TG, HDL or FMD. | HIGH |
| Jung et al. (2017), South Korea | RCT cross-over | Almonds | 56 | 4 | n=90 middle aged overweight or obese healthy adults. n=6 lost to follow-up and excluded from analysis. n=84 analysed. | 45 – 69 (52.4 ± 0.6) | 23 - 29.9  (Mean: 25.4 ± 0.22) | Without any diagnosed chronic disorders or acute inflammatory diseases 2 year prior to the enrolment, no known allergies to nuts, non-smoking (or ceased smoking for ≥1 year), not taking any vitamin supplements, functional foods or hormone replacement therapy for the last 1 month prior to the enrolment, not taking any medications known to affect lipid metabolism, such as statins and consuming typical Korean diet. | Intervention group consumed 56 g/d of roasted almonds. Control group consumed 70 g/d of isocaloric home-made cookies as a snack. | Habitual diet. | Compliance was assessed with a diary calendar and counting returned packages. Dietary intake by four 3-day dietary records (2 weekdays and 1 weekend day). BP using an automatic BP monitor. Serum TG, TC, LDL, HDL, apo A, and apo B were analysed using an automated biochemical analyser. | At the end of the intervention, neither almonds nor control food altered BP as compared to the respective baseline value. Mean TG, HDL, apo A and apo B values were not significantly changed during the intervention. Almonds significantly decreased TC and LDL. | HIGH |
| Kamoun et al. (2021), Tunisia | RCT parallel | Walnuts | 15 | 6 | n=28 active elderly men. n=8 loss to follow up/dropped out n=20 completed the study. | ≥ 65 (C: 66.9 ± 2.13, I: 66.5 ± 2.68) | C: 25.51 ± 2.48, I: 24.5 ± 2.45 | Without smoking and alcoholic participants or those with a background in systematic physical training during the 2 months before the study were excluded. No participant was taking drugs that may influence the sleep/wake cycle. | Intervention group consumed 15 g of walnut at 10:00 am daily additionally to their habitual diets. Control group were asked to maintain their usual dietary habits during the period of intervention. | Habitual diet. | Compliance NR. Dietary intake by a detailed verbal explanation and written instructions on data collection procedure. TC, HDL, LDL and TG were measured enzymatically. | HDL significant increase from pre-intervention only for the intervention group. TC, TG and LDL significant decrease from pre-intervention only for the intervention group. In addition, the intervention group had a significant decrease in TC from the control group. | HIGH |
| Kasliwal et al. (2015), India | RCT parallel | Pistachios | 80 (~ 1.5 oz shelled pistachios) | 12 | n=56 adults with dyslipidemia, (n=27 control and n=29 intervention).  n=14 lost to follow up. n=42 completed the study. | 25 - 60  (I: 37.7 ± 7.6, C: 40.4 ± 8.2) | I: 26.1 ± 2.9,  C: 27.8 ± 4.7 | Without on lipid-lowering drug therapy or had lipid abnormalities that mandated initiation of pharmacotherapy during the first visit itself. Without diabetes mellitus, uncontrolled hypertension requiring modification of antihypertensive treatment, known cardiovascular disease, and pregnant women. | Intervention group had life style modifications with daily pistachios. Control group had lifestyle modification alone. | Therapeutic lifestyle change' diet recommended by the American Heart Association. | Compliance by biweekly telephone calls. Additionally, in the pistachio group, monthly visits to collect the next month’s quota of pistachios where patients were questioned if they were actually consuming the pistachios provided. Dietary intake:NR. BP was measured in the right arm, with the participant in supine position, using a standard sphygmomanometer. Biochemical investigations included fasting lipid profile including estimation of apo A1 and apo B. Brachial artery FMD was performed using a 7.5 MHz linear array transducerattached to a commercially available ultrasound system. FMD was calculated as percentage increase in the diameter of the brachial artery from the baseline. PWV measurement was performed using a noninvasive deviced the PeriScopeÒ. | Patient in the pistachio group had a significant increase in HDL and a significant reduction in LDL, and TC-to-HDL ratio from baseline. At 3 months, patients in control group had increases in PWV in all arterial segments and a reduction in BAFMD but these changes were not statistically significant except for cfPWV. In contrast, those in the pistachio group had significant reduction inleft baPWV and statistically nonsignificant improvement in most other parameters, including BAFMD. As a result, patients in the pistachio group had lower cfPWV, lower left baPWV, and lower average baPWV compared with the LSM group. | HIGH |
| Katz et al. (2012), US | RCT cross-over | Walnuts | 56 | 8 | n = 46 recruited. n =40 completed the study. Intention-to-treat used (n=46) | Mean: 57 | 33.2 | Without diabetes or other diseases or were allergic to walnuts. | Intervention group contained walnut enriched ad libitum diet with 56g shelled unroasted English walnuts/day. Control group consumed ad libitum diet without walnuts. (8 week wash-out period included between dietary periods). | Ab libitium diet | Compliance by a 3-day diet record (completed once during run-in period, once during each dietary period and once during the wash-out period). Walnut consumption log sheets also completed. Participants counselled by dietitians on substituting walnuts for other foods to ensure energy intake remained stable. Serum lipids, BP and weight were measured after a minimum 8 hour fast. BP was measured supine after 5 minutes of rest. | Compared to the control period, walnut consumption resulted in a significantly greater increase in: TC, TG, HDL, LDL, SBP, DBP. Significantly greater increase in FMD found for walnut group. | HIGH |
| Kay et al. (2010), US | RCT cross-over | Pistachios | I1: 32 to 63  I2: 63 to 126 | 4 | n= 28 adults with moderately elevated LDLholesterol. n=1 WD. n=28 completed the study. | 35 – 61 | 26.8 ± 0.7 | Without use of BP or cholesterol-lowering medication, supplemental use of psyllium, fish oil, soy lecithin, or phytoestrogens, being pregnant or wishing to become pregnant 6 mo before or during the study, lactating 6 week before or during the study, having weight loss ≥ 10% body weight 6 mo prior to the study, following vegetarian or weight-loss diets, having diabetes, liver, kidney, or autoimmune diseases, or previous stroke, and inability to comply with the study protocol. | Intervention diet consumed 10% of energy from pistachios (amounts ranged from 32 to 63 g/d). Intervention 2 consumed 20% of energy from pistachios (amounts ranged from 63 to 126 g/d. Control diet did not consume pistachios. | Isoenergetic and matched for saturated fat and cholesterol. | Compliance by daily questionnaires. All food was prepared and consumed by the participants at the Metabolic Diet Study Center at The Pennsylvania State University. They ate 1 meal per day in the center and had their other meals and snacks packed for offsite consumption. TC and TG were determined by enzymatic procedures with commercially available kits. HDL was estimated according to the modified heparin-manganese precipitation procedure, whereas LDLl was calculated by Friedewald's equation. | After the interventions participants had lower LDL relative to baseline (P<0.05) and both the intervention 1 and 2 resulted in lower post-treatment concentrations compared with the control diet (P<0.05). | HIGH |
| Khorramirad et al. (2021), Iran | RCT cross-over  (After an 8-week washout period, the participants were crossed over to the alternate arm in both groups) | Pistachios | 50 | 12 | n= 48 randomised. n=4 WD. n=44 completed study (Group A, n=23 and Group B, n=21). | I: 53.1 ± 10.1 C: 50.1 ± 10.8 | I: 30.3±4.1  C: 31.8±7.3 | Patients with diabetes of at least 1 year’s duration, not taking multivitamin supplements within 1 month before the study, treatment with oral hypoglycemic agents, having normal levels of serum creatinine, and liver function tests within the normal range. | Intervention group followed their routine diet and consumed 50 g of raw pistachios per day at morning and evening snack times for 12 weeks, while the control group followed their routine diet for 12 weeks. (Each subject followed the 2 diets and had an 8-week washout period). | Usual diet. | Compliance adherence to the normal diets and possible changes were carefully monitored by using food record questionnaires, which were completed in 12 days in 4 separated phases The nutrient composition of the diets was calculated using the Nutritionist 4 software. The lipid profile for each patient was measured before and after each phase. Blood samples were collected after overnight fasting. TC, TGs, and HDL were measured by enzymatic procedures (Pars Azmun, Iran). LDL was determined indirectly according to the Friedewald formula. | Pistachios reduced resting SBP, but it did not improve the lipid profile of the participants. | HIGH |
| Kocyigit et al. (2006), Turkey | RCT parallel | Pistachios | 65-75 (~20% energy) | 3 | n = 44 recruited and completed (I: n = 22, C: n = 22). | Mean I: 32.8 ± 6.7, C: 33.4 ± 7.2 | Mean I: 24.2 ± 6.1  C: 24.6 ± 5.6 | Doctors and nurses working at the Research Hospital, University of Harran, Turkey who did not eat nuts ≥ once per week, or had any food allergies, smoked cigarettes, history of hypertension or atherosclerotic metabolic diseases, took any medications on a regular basis, had serum cholesterol <20th percentile or >80th percentile. | Control group followed regular diet group with no nuts and the intervention group consumed pistachio involving substitution of pistachios for 20% of daily caloric intake for 3 weeks. A 1 week run-in diet of usual intake (excluding nuts) preceded the intervention. Actual nut intakes in the intervention group ranged from 65-75 g/d depending on caloric requirements of the participants. | Regular diet | Compliance assessed by conducting weekly meetings with dietitian. One week dietary records kept at baseline (not clear how regularly kept during study). Overnight fasting blood samples tested for TC, TG, HDL, LDL. TC, TG, HDL, LDL measurements were made using a colorimetric method with a commercial kit with an automatic analyser. | No significant change in LDL, and TG. Significant effect of pistachio diet compared to control on: TC, HDL, and LDL:HDL ratio. | HIGH |
| Kris-Etherton et al. (1999), US | RCT cross-over | Peanuts | Dose not reported | 3 (+2days) | n = 26 recruited. n = 22 completed all diet periods. | 21 – 54 | 20 - 27 | With normocholesterolemic healthy adults. | Each group followed diet for 24 days. 1. Average American diet (AAD)  2. Step II diet 3. Higher fat diet with olive oil (OO)  4. Higher fat diet with peanut oil (PO)  5. Higher fat diet with peanuts and peanut butter (PPB)   All meals provided 4 – 11 day break between each study period | Average American diet, step II diet, or three high MUFA diets | Compliance was monitored by body weight measurements and a dietary assessment questionnaire administered daily. Serum concentrations of TC, HDL, and triacylglycerol were determined by enzymatic assays, LDL calculated. | Lipid levels found to be significantly different after consuming high MUFA diets, compared to the average American diet.  LDL:Step II and all high MUFA diets lower than AAD. HDL:Step II lower than AAD, p<0.05, no significant difference for high MUFA diets, although there was a trend for higher HDL.  TG: All high MUFA diets lower than AAD.  LDL:HDL: Step II and all high MUFA diets lower than AAD. | HIGH |
| Kurlandsky and Stote (2006), US | RCT parallel | Almonds | 60 | 6 | Total: n = 47 (control: n =12, chocolate: n = 12, almond: n = 12, chocolate and almond: n =11). Two participants were excluded due to illness and not meeting inclusion criteria.. | 22 – 65  C: 51.3 ± 6.3 I: 41.8 ± 11.7 | Mean C: 26.1 ± 4.1  I: 25.3 ± 3.5 | With TC 4.1-7.8mmol/L, no hx of HT / atherosclerosis or metabolic diseases, no lipid-lowering meds/ diet supplements, willingness to maintain current weight and PA patterns. Oral contraceptives and HRT meds were stabilised for study. Without allergy to nuts or chocolate, smoker, >2oz alcohol/day. | Subjects randomly assigned to National Cholesterol Education program therapeutic lifestyle changes diets containing: Intervention group 1 with almonds (60g/d) (n=12), intervention group 2 with chocolate & almonds. Control (no chocolate or almonds). | Self-selected diet. | Compliance by a 3-day diet records (2 weekdays, 1 weekend) reviewed fortnightly and recorded at week 0, 2, 4 & 6. Blood samples collected after 12 hour fast, at 0 & 6 weeks. Serum lipids analysed included LDL, TC, HDL and TGs. Serum lipids analysed at commercial clinical laboratory, LDL calculated. | No difference between groups in change in: LDL, HDL and TG. | HIGH |
| Le et al. (2016) Rock et al. (2016), US | RCT parallel | Walnuts | 42 (18% of energy) | 52 | n=245 overweight and obese women. n=28 loss to follow-up. n=213 at 6 month analysis (TC only) (Le et al., 2016). n=214 at 12 month analysis (87%). low fat diet, n=76 low carb diet, n=69 walnut rich diet, n=69 (Rock et al., 2016). | Mean: 50 (≥21) | 27 to 40 (Mean 33.5) | Without an inability to participate in physical activity due to severe disability, history or presence of a comorbid disease for which diet modification and increased physical activity may be contraindicated, self‐reported pregnancy or breastfeeding or planning a pregnancy within the next year, current involvement in another diet intervention study or weight loss program, and history or presence of a significant psychiatric disorder or any condition that would interfere with participation in the trial. | Intervention group consumed walnut-rich (18% energy) diet i.e., 42 g (1.5 oz) of walnuts per day. Control groups either a lower fat (20% energy)/higher carbohydrate (65% energy) diet or higher fat (35% energy)/lower carbohydrate (45% enegry). | Energy-reduced meal plan. | Compliance by recording walnut consumption on a simple form. Dietary intake by web‐based tracking programs that guide dietary intake toward the prescribed macronutrient distribution. The Kodak Ektachem Analyser system was used to measure TC, TG, and HDL with enzymatic methods, and LDL values were calculated. | 6 months (TC only) There was a significant reduction in TC in all 3 of the study groups; however, only the walnut-rich diet group had a notable reduction in the percentage of participants with high cholesterol.  12 months  TGs significantly decreased from baseline at study end in all three groups . Although HDL decreased at 6 months in lower fat diet group subjects (data not shown), HDL had increased from baseline in each of the diet arms at study end. TG and HDL differed by insulin resistance status. TG was higher in insulin resistant than in insulin sensitive women, whereas HDL was lower in insulin resistant women than in insulin sensitive women. No significant 3-way interactions were observed for lipids. | HIGH |
| Lee et al. (2014), South Korea | RCT parallel | Mixed nuts | 30 (walnuts: 15 g, 7.5 g, pine nuts: 7.5 g, peanuts: 7.5 g) | 6 | N= 61 participants randomised, n = 60 completed the study (nut group: n = 30, control group: n = 30).. | 35 – 65 | 23 | Adults with the MetS, aged 35 – 65 years, with BMI at least 23kg/m2. Participants taking medication for diabetes were not included, as were those with elevated HbA1c levels, nut allergies or those consuming 15g nuts 3 times a week or more. | Intervention group followed dietary recommendations for prudent diet plus 30 g/d of mixed nuts (15 g raw walnuts, 7.5 g raw pine nuts, 7.5 g roasted peanuts). Control diet contained dietary recommendations for prudent diet. | All participants were provided dietetic support to help them maintain their usual diet. | Compliance to monitor adherence to the interventions daily self-record of dietary intake, three day food record (prior to baseline and at 6 weeks) maintained. BP was measured manually with a mercury sphygmomanometer. Serum lipid levels were measured in the fasting state. | No significant effect for nut consumption compared to baseline on: SBP, HDL, LDL, TG, and TC. Non-significant effects found for endothelial function markers. | HIGH |
| Lee et al. (2017), US | RCT cross-over | Almonds | 42.5 | 4 | n=48 overweight and obese individuals with LDL between the 25th and 95th percentile from the National Health and Nutrition. Examination Survey 1999–2000. n=17 WD. n=31 completed the study. | 30 - 70 (Mean: 46.3 ± 1.8) | 25 - 40 (29.6 ± 0.5 ) | Without smoking, elevated BP, a history of MI, stroke, DM, liver disease, kidney disease, thyroid disease (unless controlled by medication), and inflammatory gastrointestinal tract disease. Without taking nutritional supplements, herbs, vitamins or minerals, nonsteroidal anti‐inflammatory drugs, cholesterol‐lowering supplements/ medications, and stanol/sterol supplemented foods. Women lactating, pregnant, or planned to become pregnant were excluded. Individuals who followed a vegetarian diet or had nut allergies were excluded. | The 4 test diets:  AAD (Control): no natural cocoa powder and dark chocolate, almonds. ALD: 42.5 g of raw almonds. CHOC: 18 g of natural cocoa powder and 43 g of dark chocolate. CHOC+ALD: 42.5 g of raw almonds, 18 g of natural cocoa powder and 43 g of dark chocolate. | Isocaloric weight maintenance diets. | Compliance was assessed by the Diet Study Center coordinators who checked daily weight logs and daily food logs to ensure that participants consumed all meals and snacks provided. TC and TG were measured by enzymatic analysis. HDL‐C was measured according to the modified heparin‐manganese procedure. The Friedewald equation was used to calculate LDL‐C. Apolipoproteins were calculated using equations according to Kulkarni et al. FMD of the brachial artery was scanned by ultrasonographic probe in a longitudinal section. BP was measured in triplicate by a standard mercury sphygmomanometer. | The ALD had significantly lower levels of TC and LDL compared with the AAD. However, there were no differences between the diets for HDL and TG. The CHOC+ALD had a significant lower concentration of apo B compared with the AAD; however, there was no treatment effect on apolipoprotein A1. The ratios of apo B/apo A1 and TC/HDL‐C were significantly lower after the CHOC+ALD compared with the AAD. | HIGH |
| Li et al. (2010), US | RCT parallel | Pistachios | 53 | 12 | n = 70 randomised. n = 59 entered the study, n = 52 completed (pistachio: n = 27, pretzel: n = 25).. | I: 45.4 ± 2,  C: 47.3 ± 2.3 | 27 - 35 I: 30.1 ± 0.4  C: 30.9 ± 0.4 | With good health without type 2 diabetes, glucose intolerance, high TC, currently taking cholesterol- lowering agents, drank >1 alcoholic drink/day. | Intervention group had afternoon snack of 53 g (240 Cal) of salted pistachios (n=31, n=27 completed), 15% protein, 55% carbohydrate, 30% fat while control group had afternoon snack of 56g of salted pretzels (220 Cal) (n=28, n=25 completed), 15% protein, 65% carbohydrate, and 20% fat. | Meals were isocaloric. | Compliance monitored by instructing to record their daily food intake on weekly food record. Met with dietitian fortnightly to ensure compliance with diet. Fasting bloods measured at weeks 0, 6 & 12. Serum cholesterol, HDL, and TG concentrations were determined using standard enzymatic methods, LDL was calculated. | No significant differences in TC, LDL or HDL between groups. TG had significant differences between groups at weeks 6 and 12. | HIGH |
| Lima et al. (2017), Brazil | RCT parallel | Hazelnuts | 25 (1 capsule) | 8 | n=40 overweight or obese women. n=10 per group. | 20 - 59 | 25.0 - 35.0 (30.5 ± 5.3) | Without alcoholism, smoking, neuro- psychiatric disorders, use of drugs known to interfere with folic acid metabolism (in the last 3 months), use of multivitamin or mineral supplements, use of anorexi- genic substances or of anabolic substances, chronic dis- eases affecting the endocrine and metabolic system, pregnancy, plans to become pregnant, and loss of weight during the study period. | Group 1 subjects received 300 g of vegetables and legumes (~191 μg/day folate) and 1 hazelnut oil capsule; Group 2 subjects received 300 g of vegetables and legumes (~191 μg/day folate) and 1 placebo capsule, Group 3 subjects received 300 g of vegetables and legumes (~90 μg/day folate) and 1 hazelnut oil capsule, and Group 4 subjects maintained their regular dietary habits. | Dietary Reference Intake (DRI) formulas to meet the recommendations of the American Heart Association | Compliance and dietary intake by 24-h dietary recalls. Lipid profile concentrations were determined using the turbidimetry method using a Labmax 240 premium- Labtest automated biochemical analyser. | The values of TC and TG did not show significant differences between the intervention groups. However, for LDL, a decrease was observed in groups 1, 2, and 3. Significant differences observed between the means of HDL values, in groups 1 and 2, and in groups 1 and 3, that is, group 1 presented a higher level of HDL, post- intervention. | LOW |
| Liu et al. (2013)  Li et al. (2011), Taiwan | RCT cross-over | Almonds | 56 (20% of energy) | 4 | n = 22 subjects with type 2 diabetes and mild hyperlipidaemia, taking OHAs but not insulin. n=2 WD. n=20 subjects completed the study (9 male, 11 female). | 58 ± 2 | 26.0 ± 0.7 | Without dietary restrictions/food allergies, not receiving insulin therapy, not using medications or supplementations known to alter lipid metabolism, stable blood lipid and sugar levels within 3 months before study (routinely determined every 4 months in patients), no clinical history of cardiovascular, hepatic, gastrointestinal, or renal disease, no alcoholism, no recent-history of smoking, and normal liver and kidney function. | Intervention group consumed roasted, unsalted whole almonds with skins into meals to replace 20% calories of control diet. On average, 56 g almonds/day. Control group was based on NCEP step II dietary guideline. | Calculated energy need of each subject based on their daily activity level. | Compliance by daily food diary in which the patient recorded the study foods not eaten, non-study foods eaten, and beverages consumed. Dietary intake by packaging all meals for take-out. Lipid profiles in serum were determined with enzymatic assays using a clinical chemistry analyser. Concentrations of apo A1 and apo B in serum were measured using immunoturbidimetric immunoassays. VCAM-1 and ICAM-1 were determined using commercial ELISA assays obtained from R&D Systems. BP was determined using the automatic BP monitor FT-500 R. | Significantly different effect of almond arm on: TC, LDL, LDL:HDL ratio. No significant effect on BP, TG, and HDL. Compared to the control diet, greater reductions were seen with the almond intervention for apo B and apo B/ apo A-1 ratio. ICAM-1 and VCAM-1, were not altered by the almond diet. SBP and DBP were not altered by the diets. | HIGH |
| Liu et al. (2018), South Korea | RCT parallel | Almonds | 56 | 20 | n=169 healthy nonsmoking adults completed the original 16 week trial. n= 85 participated in the additional 4 weeks. 57 intervention group (M 31/F 26) and 28 control group (M 14/F 14). | 20 - 39 (I: 26.96 ± 5.22, C: 26.14 ± 5.40) | 17 - 30 (I: 23 ± 3.17, C: 21.66 ± 3.08) | With usual level of physical activity and avoid consuming any additional nuts or nut products. Nonsmokers who had no clinical symptoms and significant changes of body weight during the last 6 months. | Intervention group consumed 56 g of almonds/day. Control group consumed a high-carbohydrate control food items. | Habitual diet. | Compliance by self-report. A consumption log sheet was provided for participants to keep a record of their almond or cookies consumption. Subjects reported to care providers, trained registered dietitians using text message how many packages they did not consume during previous week. Dietary intake by 3-day diet records. BP measured on right arm using an up-load BP monitor. Serum TC and TG levels were measured by the enzymatic colorimetric method using a Cobas 8000 c702 chemistry analyser. HDL and LDL levels were determined through homogeneous enzymatic colorimetry. | Intervention group had significantly reduced level of TC, HDL, TC:HDL, LDL and TG compared with values at baseline. While HDL:LDL was significantly increased after consuming almond for 20 weeks. Control group had significantly reduced level of TC, TC:HDL, LDL and TG. Intervention group compared with control had a significant greater reduction in TC, HCL-C, LDL and TG.   Results from mixed procedure Time, intervention, and interaction effects throughout the trial were analysed using the mixed model. Significant interaction effects were observed for the changes in TC and non-HDL. There were significant group effects for changes in HDL, TC:HDL, HDL:LDL and TG. | LOW |
| Lovejoy et al. (2002), US | RCT cross-over | Almonds | 57-113 | 4 | n =34 enrolled. n=30 subjects completed the study. (17 females, 13 males). | Mean: 53.8 ± 1.9 | Mean: 33 ± 1.0 | Without medication to lower cholesterol. Subjects with type 2 diabetes (not taking insulin), aged 30–65 years, with a BMI of 20–40 kg/m2. | Subjects were randomly assigned to begin one of 4 diets - - high fat high almond (HFHA , 37% fat, 10% from almonds),   -low fat high almond (LFHA, 25% fat, 10% from almonds),   -high fat control (HFC, 37% fat, 10% from olive/canola oil) or   -low fat control (LFC, 25% fat, 10% from olive/canola oil).   At least 2 weeks wash-out period. Participants provided with all foods, almond dose was 57 – 113 g/d depending on energy levels | HFA: 37% fat, 10% almonds. HFC: 37% fat, 10% olive or canola oil. LFA: 25% fat, 10% almonds. LFC: 25% fat, 10% olive or canola oil. | Compliance monitored by advising subjects to keep a food diary to record study food, non-study foods and beverages consumed. Fasted blood samples were collected on two separate day in the first and last week of each diet period and a 2hr, 75 g-oral-glucose-tolerance test was conducted to determine glucose tolerance and insulin secretion. Lipids were measured on an autoanalyser using commercially available enzymatic kits. LDL was assessed via the Friedewald calculation. | There were significant main effects of fat level (low fat compared with high fat) on TC and TG, but no significant effect of fat source on these outcomes: TC, and TG. A significant main effect of fat source (almonds compared with oil) on HDL and LDL was found. No significant effect on LDL:HDL ratio  Lipid and lipoprotein concentrations during all experimental diets were lower than those at the time of enrolment. There was no significant main effect of diet on HDL subpopulation distribution.  The prediction equations based on diet composition would predict an increase in HDL whereas a decrease was actually observed. | HIGH |
| Ma et al, (2010), US | RCT-crossover | Walnuts | 56 | 8 | n = 24 (14 women and 10 men). n=21 completed the trial.  n=3 dropped out due to changes in medication or poor compliance with treatment protocol (intention-to-treat used). | Mean: 58 ± 9.2 | Mean BMI: 32.5 ± 5.0 | With type 2 diabetes diagnosed >one year no more than five years, non- smokers, stable serum glucose and medication 3 for months and NIDDM. Without eating disorder, atherosclerosis, vasoactive, lipid lowering and/or antihypertensive medication unless able to refrain from taking medication, sleep apnoea, restricted diet, nut allergy. | Intervention group consumed walnut-enriched ad libitum diet and control gorip with ad libitum diet without walnuts, separated by an 8-week washout period. | Usual dietary patterns. | Compliance with subjects by completing a 3-day diet records once during the original run-in period, once during each 8-week dietary period, and once during the 4-week washout period. The nuts were supplied to the subjects during the walnut treatment and they were asked to return empty packaging to assess compliance. Endothelial function was measured noninvasively in the right brachial artery by a high-frequency ultrasound scanning machine. Fasting serum lipids, fasting serum glucose, and plasma A1C were measured at the Griffin Hospital laboratory using standard procedures at each visit. | The control diet significantly lowered BP vs the walnut diet (study authors unsure of why this was found). Significant improvement in flow-mediated dilation found with walnut consumption. Significant decrease TC, LDL from baseline however, no significant decrease when compared to control. No improvement TG and HDL. | HIGH |
| Madan et al. (2021), India | RCT parallel | Almonds | 56 | 74 | n=421 (M 88, F 333) randomised. n=146 WD, n=107 in the almond group; n=112 control group analysed. | 16 - 25 | NR | With fasting glucose levels between 100 and 125 mg/dL and 2-h post-glucose value 140–199 mg/dL and/or fasting hyperinsulinemia or glucose challenge hyperinsulinemia. Without the presence of any known chronic disease, known history of food allergies with nuts, on prescribed medications like steroids, state of pregnancy and/or lactation. | Intervention group received raw almonds daily (providing ~20% of the daily energy intake i.e., 340 kcals/d) which was distributed in 2 packets that were to be consumed as snacks in a day. Control group received commonly consumed isocaloric (as provided by 56 g of raw almonds). | Usual diet. | Compliance achieved by contacting over the phone every 2 days to check for the consumption of either almonds or the control product. Participants were instructed to return the unused portions twice a week that were weighed to estimate the intake during a 7-day period. Dietary intakes were estimated by trained research assistants using 1 day 24-h diet recall. Each participant underwent a clinical examination that was conducted by a physician to assess the general health status at screening, baseline as well as at the end of the study. Whole blood was analysed for complete blood count. | The baseline characteristics of participants in the almond group did not differ significantly from those in the control group for any of the biochemical measurements. | HIGH |
| Mah et al. (2017), US | RCT cross-over | Cashews | 28– 64 (11% of total energy) | 4 | n=51 healthy adults with fasting LDL concentrations ≥130 and <200 mg/dL. n=8 WD. n=51 ITT analysis and n=42 per protocol. | 21 - 79 (55.7 ± 1.42) | ≥18.0 and <32.0 (26.9 ± 0.39) | Without cardiovascular disease, diabetes mellitus, and uncontrolled hypertension, medications or supplements that were intended to alter the lipid profile or of weight-loss drugs. | Intervention group consumed roasted, salted cashews. Control group consumed baked potato chips (Lays Baked Chips; PepsiCo). | Typical American diets. | Compliance with study-product consumption was documented by the percentage of scheduled study-product intakes over each 28 day intervention. Compliance with the overall diet by the study staff according to the returned food items. Meal plans were assigned to subjects on the basis of energy needs. If a subject consumed a nonstudy food or caloric beverage, he/she was instructed to record intake of the food/beverage item in a provided notebook and to return the uneaten portion of the nonstudy food or the label of the nonstudy item to the clinic. All blood samples (HDL, TC and TG) were collected after an overnight (12 ± 2 h) fast. LDL was calculated according to the Friedewald equation. | Compared with the control diet, the cashew diet significantly decreased concentrations of LDL, TC, and non-HDL without significant changes to HDL and TG concentrations. | HIGH |
| Maranhao et al. (2011), Brazil | RCT parallel | Brazil nuts | 15-25 (3 to 5 units/d, 124Cal) | 16 | n= 17 (Brazil nuts: n = 8, placebo: n = 9). | Mean: 15 | 35.6 ± 3.3 | Female adolescents, obese (15.4(2) yrs) from outpatient cardiovascular disease prevention clinics, >95th percentile for BMI by age. Without using any nutritional intervention or drugs, had chronic disease (DM, hypertension), lactose intolerance, or lost weight in prior 6/12, beyond stage IV for Tanner pubertal development, dietary habits showed excess consumption of any kind of nut. No differences between groups on anthro, lab or microvascular variables. | Intervention group consumed Brazil nuts (15-25 g/d, equivalent to 3 to 5 units/day, 124 calories – calculated to achieve 10% of energy from MUFAs in the diet), with snacks or meals. Control group received placebo (1 lactose capsule/day). | Diet duration was 16 weeks. Advised not to change their usual dietary habits. | Compliance at baseline and end of study, usual food intake assessed by dietary inquiry. Serum selenium levels and returned empty nut bags used as markers of Brazil nut consumption compliance. Bloods (measured in duplicate after 10 – 12 hour fast using automated method) analysed at baseline and after 16 weeks. | Brazil nut group had reduced TC, LDL, TG compared to lactose placebo. | LOW |
| McKay et al. (2010), US | RCT cross-over | Walnuts | 21 or 42 | 6 | n=21 non-smoking men and postmenopausal women. n=21 completed the study. | 21g: 64.8 ± 7.8 42g: 56.6 ± 13.9 | 21g: 27.3±3.6 42g: 27.4±3.8 | Without history or known allergy to nuts, regularly consumed 140 g nuts/week within 6 week of study admission, were taking estrogen, oral steroids or cholesterol-lowering medications, had renal, endocrine or gastrointestinal disease, rheumatoid arthritis, presented with SBP >150 mm Hg and/or DBP >95 mm Hg; had usual ethanol intake ≥2 drinks (28 g)/d or used illicit drugs, presented with EKG or standard clinical laboratory values outside acceptable parameters, and used dietary supplements containing either fish oils, high doses of vitamins C, E, β-carotene or selenium, and/or phenolic compounds within 6 week of study admission. | Intervention group received either 21 g or 42 g packaged raw walnuts/day during the first phase and the alternate dose during the second intervention phase. | Usual diet. | Compliance from each subject by keeping track of their walnut intake using a daily diary chart, and by having the study dietitian count opened bags at each visit. Dietary intake by validated FFQ. TC, LDL, HDL, and TG were determined with a clinical chemistry analyser. | Plasma TC, LDL, and TG levels decreased significantly compared to baseline levels after 6 week with the 21 g/d dose, but not with the higher dose. | HIGH |
| McKay et al. (2018), US | RCT cross-over | Pecans | 42.5 (15% energy) | 4 | n=26 healthy middle-aged and older adults who are overweight or obese with central adiposity. n=0 WD. n=26 completed the study. | ≥ 45 (M: 57.9 ± 1.0, F: 67.4 ± 2.9) | 25–35 (M: 29.4 ± 0.7, F: 28.4 ± 0.9) | Without the presence of cardiovascular disease, use of estrogen, with or without progesterone, use of medications known to affect lipid metabolism or suspected to influence BP, chronic disease, regular use of oral steroids, regular use of anti-inflammatory agents, inability to discontinue or refrain from aspirin, non-steroidal anti-inflammatory drugs, or acetaminophen use for 72 h prior to and during testing visits, SBP>150 mmHg and/or DBP>95 mmHg, regular use of any dietary supplements within ≤30 days, usual daily ethanol intake of ≥2 drinks, cigarette smoking and/or nicotine replacement use, allergy to nuts of any kind, frequent nut consumption (>142.8 g/week) or inability to refrain from consuming all nuts and nut products within ≤30 day, unwillingness or inability to consume animal-based foods, and laboratory blood or urine biochemistries outside of normal ranges. | Intervention group consumed pecans isocalorically substituted for 15% of the total energy (~42.5 g/2000 kcal). Control group consumed a typical American diet, i.e., low in fruit, vegetables, fiber, and n-3 fatty acids, and high in nutrient-poor, calorie-dense foods. | NA. | Compliance was assessed by the study dietitian. Dietary intake by subjects returning twice every week to pick up their supply of meals and drop off their empty containers and unused food. Plasma concentrations of TC, LDL, HDL, and TG were determined with a clinical chemistry analyser. SBP and DBP were measured at the brachial artery by use of an automated BP device. E-selectin and endothelin in serum were measured separately using quantitative solid phase sandwich enzyme-linked immunosorbent assay kit procedures. | Both TC and LDL were lower following the pecan diet. SBP and DBP, were also observed to be lower with the pecan diet; however, neither the within- nor between-group differences reached statistical significance. No statistically significant differences between groups were observed in endothelial function. | HIGH |
| Mohan et al. (2018), India | RCT parallel | Cashews | 30 | 12 | n=300 adults physician-diagnosed type 2 diabetes, duration of type 2 diabetes<10 y, and currently receiving oral hypoglycemic drugs, n=150 per group. n=31 WD. n=269 completed the study.. | 30 - 65 (51 ± 9.3) | 26.0 ± 3.4 | Without cashew nut allergy, not currently receiving insulin therapy, glycated hemoglobin (HbA1c) >10%, LDL >190 mg/dL, TC >240 mg/dL, TGs >300 mg/dL, and any known diabetes complications. | Intervention group consumed 30 g unsalted, raw, broken cashew nuts/d. Control group were advised to not consume any other types of nuts, follow their prescribed standard diabetic diet, exercise, and medications as usual. | Participants prescribed standard diabetic diet. | Compliance monitored by participants returning empty sachets of cashew nuts every week. Dietary intake by 24-h dietary recalls (1 weekday and 1 weekend). BP was assessed twice on each occasion at 5-min intervals and the average reading was taken. A Beckman Coulter AU 2700/480 Auto- analyser was used to measure serum cholesterol, serum TG, and HDL by direct method with polyethylene glycol-pretreated enzymes. LDL was calculated using the Friedewald formula. | The intervention group had a 1.9-fold greater reduction in SBP compared to participants in the control group. The intervention group also had a 16-fold greater increase in HDL compared to participants in the control group. There were no significant changes in other serum lipid variables. | HIGH |
| Morgan and Clayshulte (2000), Mexico | RCT parallel | Pecans | 68 | 8 | n = 23 randomised. n =19 completed study. I: n = 10 (7women, 3 men). C: n =9 (8 women, 1 man). | Mean C: 45 ± 10, I: 37 ± 12 | 24 ± 5 | With normo-lipidemic men and women. Without any active disease process, pregnancy and lactation in women, drug or alcohol abuse, food allergy to nut consumption and ingestion of lipid-lowering medications. | Intervention group consumed self-selected diets plus 68 g pecans per day for 8 weeks. Control group consumed self-selected diets. | Self-selected diets were nut-free. | Compliance achieved by instructing recorded 3-day food diaries at 2 week intervals (baseline, week 2, 4, 6 and 8). A food frequency checklist was completed by subjects at the commencement of the study to verify that food diaries were not dissimilar from usual dietary intakes. Compliance with the dietary protocol was monitored by interviewing participants at each study visit, reviewing food records and for the pecan group inspecting pecan ration boxes. Fasted blood samples were collected at baseline and after 4 and 8 weeks. LDL and VLDL was separated from plasma using phosphotungistic acid and cholesterol measured by enzymatic analysis. Analytical procedures were certified by the Northwest Lipid Research Laboratories with traceability according to the guidelines of the National Reference System for Cholesterol. | Significantly lower lipid levels at 8 weeks for the pecan group compared to control was found for: TC, LDL and HDL.  Non-significant reduction in TG found in both groups. | HIGH |
| Morgan et al. (2002), US | RCT cross-over | Walnuts | 64 | 18 | n = 67 enrolled. n = 49 randomised.  n = 42 completed the study.. | Mean: 55.7 ± 11.8 | Mean: 27.7 ± 5.8 | Outpatients with borderline high TC (greater than 5.2 mmol/L) and had no evidence of cardiovascular disease. Subjects were otherwise of good health and not allergic to walnuts. Subjects enrolling in the study were already adhering to a low-fat, low-cholesterol diet. | Intervention group consumed 64 g of walnuts daily as part of their diet. | Patients were stabilized on the low-fat, low cholesterol diet (AHA Step I diet) for the first 6 weeks. The AHA Step I diet provided 30% energy as fat, and less than 14 % energy from saturated fat. The aim was to have the walnut diet the same as the low-fat, low-cholesterol diet with respect to total energy, carbohydrates, protein and total fat. | Compliance by a 3-day diet record was obtained in the first and last week of the stabilization period, and at the end of each dietary period. Fasted blood samples were obtained from subjects at the initial visit, at the end of week 4,5 and 6 of the diet stabilization period, and one time at the end of each treatment period. Bloods were analysed for TC, HDL, coagulation parameters (factor VII, tPA, PAI-1 and fibrinogen) Lipids were measured using standard analytical procedures in a certified laboratory. Homocysteine was determined by fluorescence polarization immunoassay. | Statistically significant reductions in TG were found in the walnut group only. Non-significant effects found for: TC, LDL and HDL There were no statistically significant changes in coagulation parameters or homocysteine concentrations. | LOW |
| Mukuddem- Petersen et al. (2007)  Schutte et al. (2006), South Africa | RCT parallel | Walnuts or Cashews | 63 – 108 (~20% of energy) | 8 | n= 68 recruited. n = 64 completed (walnuts: n = 21, cashews: n = 21, control: n = 22). | I1: 45 (40.4 - 50.2), I2: 46 (40.7 - 50.7), C: 45 (40.8 - 49.3) | I1:  36 (95% CI 33.3 - 38.7),  I2:  34.4 (95% CI 32.2 - 36.6),   C: 35.1 (95% CI 32.8 - 37.4) | Without being pregnant, lactating, using thiazide diuretics or beta-blockers, nut allergies or diagnosed diabetes. With MetS defined using NCEP ATP III criteria. Subjects were included if they were able to comply with specified feeding conditions, able to eat walnuts and cashew nuts, aged 21-65. Walnut group with 107 (99.4-115) kg, 109 (103-115) cm waist. Cashew with 99(92.5- 106) kg, 105 (98.5-111) cm waist. Control with 106(99.2-113) kg, 108(102- 113) cm waist. | Intervention group consumed walnuts ~20% energy (63-108 g/d). Intervention group 2 consumed unsalted cashew nuts ~20% energy (63-108 g/d). Control diet without nuts of any kind. | Meals were provided to participants (~16% protein, 47- 51% carbohydrate, 33-37% fat). Participants assigned to energy intake level closest to their habitual energy intake to ensure body weight maintenance. | Compliance by assisting to keep food diaries to record all study foods, additional foods and any deviations from the study diet.: (1) foods were weighed to the nearest gram before being served to the participants; (2) the principal investigator, a registered dietitian, supervised mealtimes and ensured the complete intake of all study foods; (3) participants kept food diaries of the additional points used and possible left-overs were collected and weighed (by researchers). Fasting blood samples and BP measurements were taken at the end of the run-in (baseline) and at the end of the 8-week study. Serum TC, HDL, and TG were measured on a Vitros DT60 II Chemistry System. BP measurements were obtained by taking a 7 min continuous measurement of cardiovascular parameters using the Finometer. | Serum lipids not different between groups. No significant change from baseline within walnut or cashew nut group and control group had a small significant increase in HDL from baseline. No change in BP between groups or within groups from week 0 to week 8.  No significant effect of intervention on: TC,LDL, HDL, TG, SBP, and DBP. | HIGH |
| Müllner et al. (2013), Australia | RCT parallel | Walnuts | 9 | 10 | n=106 subjects with established type 2 diabetes treated with oral antidiabetic (OAD) medication and/or insulin. n=7 WD due to digestive discomfort or illness. n=7 excluded for failing to follow the study protocols. n=92 completed the study, n=18: Insulin treated type 2 diabetes mellitus allocated to nut oil, n=29: non insulin treated diabetes allocated to nut oil, n=16: Insulin treated diabetes allocated to mixed and n=29: non insulin treated diabetes allocated to mixed. | 40 - 70 | NR | With stable metabolic control, glycated haemoglobin concentration <9.5%, serum TC <300 mg/dL (<7.76 mM), serum TG <500 mg/dL (<5.7 mM) and serum creatinine <2.5 mg/dL (<221 lM). With stable body weight, constant dietary habits and physical activity levels for at least 4 weeks before entry to the study were included. Subjects who intended to change dietary habits, levels of physical activity or body weight within the study period were excluded. All medical therapies of subjects were continued unchanged throughout the study. | Intervention group consumed 9 g walnut oil. Control group consumed 9 g mixed oil (corn, sunflower and linseed oil). Participants consumed three reference spoons of oil (3 x 3 g) per day in addition to their usual diet. | Habitual diet. | Compliance by assisting oil intake by measuring plasma fatty acid composition and ɣ-tocopherol levels in plasma. Dietary intake by 24-hr recalls to assess energy and macronutrient intake. Serum TG, TC and HDL levels were measured enzymatically using an Abbott Aeroset Analyser. LDL concentrations were calculated according to the Friedewald formula. | No significant effects of walnut oil on lipid metabolism (TC, HDL, LDL and TG). None of these parameters were significantly different from baseline after the washout period (data not shown). | HIGH |
| Nestel et al. (1994), Australia | RCT parallel | Macadamias | 62 g of fat in products/day | 3 | n = 34.. | Mean: 49 ± 9.8 | Mean: 25.7 ± 2.96 | Males with mild to moderately elevated plasma cholesterol. Without being treated for hyperlipidaemia, or suffering from any metabolic disorder or taking medication to influence plasma lipids. | Group 1 – palmitoleic, oleic, palmitic Group 2 – palmitic, palmitoleic, oleic Group 3 – oleic, palmitic, palmitoleic Subjects were blinded to the type of fatty acid intervention. | A combination of self-selected foods of known fat content and low-fat frozen meals provided 3 times per week to facilitate meal planning. Specific advice was given to avoid all margarines and oils other than those provided as the test fats, to avoid specific foods/nutritional supplements. | Compliance by a 3-day weighed food record was kept during each test phase. Fasted blood samples were collected on 2 consecutive days at the end of baseline and after each dietary period. Plasma TC and TG were determined by enzymatic methods on an auto-analyser. HDL was determined after precipitating LDL and VLDL with PEG 6000. LDL was calculated with the friedewald calculation. Plasma fatty acids were analysed by gas chromatography during each of the 4 dietary periods. | For both total and LDL the oleic acid diet resulted in significantly lower values than either the palmitic or palmitoleic diets: TC and LDL.  HDL was significantly higher following palmitic oil consumption, compared to palmitoleic. No significant differences between diets for TGs. | HIGH |
| Nezhad et al. (2016), Iran | RCT parallel | Walnuts | 15 | 12 | n=100 diabetic adults who were diagnosed at least for two years, 50 per group. n=11 loss to follow up. n= 89 completed the study. n=100 included in analysis. | 30 - 60 (C: 54 ± 11.37; I: 55.5 ± 10.75) | C: 27.21 ± 2.27, I: 27.60 ± 2.47 | Without alcoholism, smoking, any internal organ dysfunction, being on any therapeutic diet or calorie restricted regimen, and oral consumption of glucose lowering herbs during the past three months before beginning of the trial. All the patients received oral hypoglycemic agents and did not have a history of receiving insulin. | Intervention group consumed 15 g/d cold pressed walnut oil per day. Control group did not undergo any intervention. | All patients received dietetic consultation about a balanced diet and were advised according to their calorie needed for maintaining their weight unchanged. | Compliance and dietary intake NR. BP were measured by a standard sphygmomanometer. | The two groups were not significantly different in distribution of, SBP and DBP. | HIGH |
| Njike et al. (2015), US | RCT parallel | Walnuts | 56 | 26 | n=112 subjects who were non-smokers and had a high risk for diabetes, n=28 per group (4 groups). n=6 loss to follow-up and n=7 WD. n=97 completed the study.  N=101 included in analysis.. | 25 – 75  (Ab libitum diet: 53.3 ± 11.1, Calorie adjusted diet 56.5 ± 11.7) | ≥25 (Ab libitum diet: 30.2 ± 4.1, calorie adjusted diet: 30.0 ± 4.0) | Not allergic to nuts, current eating disorder/restricted diet by choice, receiving pharmacotherapy for obesity, appetite suppressants, unstable use of lipid-lowering, antihypertensive medications or aspirin or unwilling to refrain from taking medication for 12 hour prior to ejection fraction scanning, regular use of high doses of vitamin E or vitamin C, intake of fish oil, flaxseed oil, ω-3 fatty acid, or fiber supplements unless willing to discontinue supplementation for the study duration, use of insulin, glucose-sensitizing medication, or vasoactive medications, diagnosed diabetes, sleep apnea, cardiovascular disease, coagulopathy, known bleeding diathesis, or history of clinically significant haemorrhage, current use of warfarin, regular exercise defined as participation in moderate-intensity exercise ≥150 min/week, substance abuse, any unstable medical condition, pregnant or lactating, women receiving Depo-Provera shots, and/or women receiving hormone replacement therapy. | Intervention group 1 consumed calorie adjusted diet with walnut. Intervention group 2 consumed ab libitum diet with walnut. Control group 1 consumed calorie adjusted diet without walnut. Control group 2 consumed ab libitum diet without walnut. | Calorie adjusted diet advised by registered dietitian and recipes for inclusion of walnuts in their meal plan. Participants received instruction to preserve an isocaloric condition after the addition of walnuts.  Ab libitium diet: Their caloric intake was not monitored or regulated, and thus was allowed to float ad libitum. | Compliance NR. Dietary intake by 24 hour food recalls using a web-based Automated Self- Administrated. The lipid profile was determined as follows: TC, TG, and HDL were obtained by direct measurements. LDL was obtained by calculation: LDL=TC−(VLDL+HDL). Endothelial function was measured as flow-mediated dilation (FMD), the per- centage change of brachial artery diameter from before cuff inflation to 60 s after cuff release. | EF, TC and LDL significantly improved from baseline after a walnut-included diet daily for 6 months with or without dietary counselling to adjust caloric intake. However, EF, TC and LDL did not significantly differ between the walnut-included diet for 6 months, with or without dietary counselling to adjust caloric intake compared with walnut-excluded diet. A walnut-included diet for 6 months, with or without dietary counselling to adjust caloric intake, did not significantly improve (p>0.05) BP in this sample of adults at risk for diabetes. | HIGH |
| Nouran et al (2009), Iran | RCT cross-over | Peanuts | 60g, 77g and 93g, allowing for three tertials of energy | 4 | n=60 commenced study, n=6 WD (n=2 lost interest in the study, n=4 WD due to unforeseen travel). | Mean: 43 ± 1.3 | 27.5 (SE 0.5) | Without acute or chronic disease (diabetes, liver, kidney, thyroid, cancer, inflammatory or infectious disease), consumption of vitamin supplements, hormone therapy or medication, recent history of weight gain or loss (≥9 kg) within the past 6 months, very atypical diet, rigorous exercise program, allergy or aversion to nuts, habitual consumption of >70 g nuts/week, cigarette smokers, first degree family history of coronary heat disease. Included males aged 25-65 years, TC levels 200-350 mg/dl, mean TG levels <400 mg/dl. | Intervention group consumed habitual diet supplemented with peanuts (consuming a daily allowance of peanuts equivalent to ~20% of subject’s mean energy intake in addition to habitual diet). Control group consumed habitual diet. | NR. | Compliance was assessed using 24 hour diet recalls conducted by a dietitian at 1 week intervals (3 separate time points) during each period. Serum MUFA was measured as a biological marker of adherence Energy & nutrient content of the diet was determined using Iranian modified food composition tables. A 3-day food & nutrient intakes were averaged & reported as the mean intake. TC and TG were measured using an enzymatic colorimetric method. HDL and LDL were quantified by the direct method. | Body weight was stable throughout the study. No changes in SBP, DBP, TC, LDL or TG. Increase in HDL and reductions in TC/HDL and LDL/HDL ratios. | HIGH |
| O’Byrne et al. (1997), US | RCT parallel | Pistachios | 35 – 68 (depending on energy requirements) | 26 | n = 36 enrolled. n = 25 completed the study (I: n = 12, C: 13). | 50 – 65 | I1: 26.4, C: 26.2 | With healthy postmenopausal females (50 – 65 years) with elevated serum cholesterol (5.68 – 7.76mmol/L), without indicators of genetic hyperlipidemia, without hypertension, non-smokers, and not taking medications to alter blood lipids. | Intervention group consuming a typical American diet (34% fat, 11% saturated fat) were assigned to consume a low fat, high MUFA diet (LFMR) – 50 – 60% fat as MUFA, main source was high oleic peanuts (35 – 68 g/d depending on energy requirements). Control group consumed low fat diet. | All participants were provided with dietary requirements for weight maintenance. For ethical reasons, participants who were already consuming a low fat diet were advised to continue their low fat diet. | Compliance was determined by monthly telephone conversations and evaluation of subject’s body weight and Seven-Day Diet Records. A detailed nutrient analysis of each subject's initial and final. Fasting serum lipid concentrations were measured in triplicate from each sample using microplate methods with reagents from enzymatic kits. | Significantly greater reduction in participants consuming LFMR diet than LF diet on TC and LDL. No significant group effect on TG and HDL:LFMR ratio. | LOW |
| Olmedilla-Alonso et al. (2008), Spain | RCT cross-over | Walnuts | 19.4 | 5 | n = 25 recruited (drop outs not reported). | M: 45 - 60,  F: 50 - 70 | ≥25 to <34.9 | Without use of vitamin/mineral supplements, regular use of aspirin, taking any lipid-lowering or metabolism meds, any chronic disease (eg diabetes), those receiving hormonal substitutive therapy. With cardiovascular disease risk factors, TC≥220, <290 mg/dL, or smoking and/or SBP≥140 mmHg and/or DBP≥90 mmHg, 60% M, HDL 53.5(14.5) mg/dL, LDL 152(35.5) mg/dL, TG: 150.4(89.6) mg/dL, 32% heavy smokers. Habitual consumption of a mixed diet. | Intervention group consumed 4*150 g walnut enriched restructured steaks and 80 g ratio of walnut enriched restructured sausages/week (contains 20% walnut paste, 14.5 g fat, less SFA and MUFA, 74.9 g PUFA) – 30 g walnut/steak, 16 g walnut/sausage (~19.4 g/d).  Control group received 4*150 g restructured steaks and 80 g ratio of restructured sausages/week (1.6 g fat, 19.8 g PUFA) | NR. | Compliance NR. A diet record maintained. Overnight fasting bloods collected at days 0, 12, 21, 28 and 35. Serum cholesterol and TG analysed using a modular Hitachi autoanalyser. | Significant difference between groups for TC. No differences found for: HDL, LDL, TG, and SBP. | HIGH |
| Orem et al. (2013), Turkey | RCT cross-over | Hazelnuts | 49-86 (18-20% energy, depending on individual requirement) | 4 | N= 21 (n=18 males, n=3 females) no loss/withdrawal reported. | Mean: 44.6 ± 10.4 | NR | Without any systemic illness (diabetes mellitus, liver or kidney disease, or hypertension) or history of allergy to hazelnuts. Included adults with hypercholesterolemia, if they had serum cholesterol level >200 mg/dL with or without TGs>150 mg/dL, not taking medication/supplements known to alter lipid metabolism. | Double control sandwich model intervention study with a single group and three isoenergetic diet periods: -control diet I (4 weeks) - hazelnut-enriched diet (4 weeks; hazelnut contributing 18%–20% of the total daily energy intake) -control diet period II. | The control diets were identical and based on the National Cholesterol Education Program adult treatment panel (ATP) III step 2 diet (<7 % energy from SFA and <200 mg/d dietary cholesterol). | Compliance monitored by a 3 day food record at the end of each diet period. Caloric and nutrient intake was estimated by using the BeBis computer program. Lipids & lipoproteins were assessed via a fasted blood test at baseline and days 30,60 & 90 and determined by enzymatic method using a ROCHE autoanalyzer and immunonephelometric method. | Consumption of a hazelnut-enriched diet significantly improved TC, TG, LDL, and HDL compared to control diet I. | LOW |
| Palacios et al. 2019, US | RCT cross-over | Almonds | 85.05 | 6 | n=54 prediabetic men and women. n=21 WD and loss to follow-up. n=33 (16 F and 17 M) completed the study. | 18 - 69 (48.3 ± 2.2) | 25.0 to 39.9 (30.5 ± 0.7) | Without atherosclerotic cardiovascular disease or pulmonary, endocrine (including type 1 diabetes and type 2 diabetes), chronic inflammatory, hepatic, renal, hematologic, immunologic, dermatologic, neurologic, psychiatric, or biliary disorders or if they had a recent history (prior 5 years) or presence of cancer other than nonmelanoma skin cancer. | Intervention group consumed 1.5 ounce of raw almonds twice per day (3.0 ounce/day). Control group consumed energy-matched carbohydrate based foods. | Habitual diet. | Compliance by daily log, subjects documented whether 0%, <50%, or ≥50% was consumed. Dietary intake by 3-day diet records. Lipid levels were analysed according to the Standardization Program of the Centers for Disease Control and Prevention and the National Heart, Lung and Blood Institute using enzymatic colorimetric methodology. HDL was analysed using the rate method. LDL were calculated according to the Friedewald equation. | No significant differences in responses between diet conditions observed for lipoprotein lipids and apo A1 or apo B. For almonds TC, LDL, HDL, TG, apo A1, and apo B had a non-significant increase from baseline. While TC:HDL, SBP and DBP non-significantly decreased. | HIGH |
| Parham et al. (2014), Iran | single-blind, randomised, case control, crossover clinical trial | Pistachios | 50 | 12 | n=48 patients with diabetes duration for at least one year, treatment with oral hypoglycemic agents. n=4 WD. n=44 patients completed the study (23 in intervention group and 21 in control group). | I: 53 ± 10,  C: 50 ± 11 | I: 32.16 ± 6.58, C: 30.24 ± 4.03 | Without multivitamin supplementation consumption within one month prior to study start, pregnancy or pregnancy planning, and heart failure based on clinical examination. | Intervention group received 2 snacks of 25 g pistachios per day consuming morning and evening servings. Control group received their previous diet without pistachios and routine physical activity. | Habitual diet. | Compliance was determined by a four weeks after initiation, patients visit. Dietary intake: NR. BP was measured in both arms using a mercury sphygmomanometer. | In both groups, SBP decreased during pistachio consumption. There was no significant effect on DBP. In the phase-2 but not phase-1 control group, the variation in SBP was statistically significant (p = 0.044). This points again to a sustained effect of pistachio consumption, indicating that the wash-out period may have been too short. | HIGH |
| Pelkman et al. (2004), US | RCT parallel | Peanuts | 88.3 | 10 | n=53 healthy overweight and obese (120-135% of ideal body weight). n= 1 WD and 1 removed. n = 52 included in data analysis, n=25 low fat. diet (7 M and 18 F) and n=27 moderate-fat diet (9 M and 18 F). | 20 and 67 I1: 45.4 ± 6.6 I2: 42.7 ± 10.7 | low fat diet: 29.9 ± 2.4 (26 - 34)  moderate-fat diet: 29.8 ± 2.5 (26 - 36) | With LDL concentrations between the 25th and 90th percentiles, based on age and gender and a TG concentration <4.52 mmol/L (<400 mg/dL). | Intervention group consumed moderate fat diet with 35% energy from fat, 7% energy from saturated fat and 200 mg cholesterol/day (half of fat provided by peanuts, peanut butter and peanut oil). Control group consumed low-fat diet with 20% energy from fat, 7% energy from saturated fat and 200 mg cholesterol/day.  Diets were hypocaloric (2.92 megajoule/day lower than requirements) for 6 weeks for weight loss, and eucaloric for 4 weeks | All foods were provided to subjects. | Compliance and dietary intake by daily and weekly self-reporting forms. Fasting serum TC, HDL, and TG concentrations were measured by enzymatic assays. LDL were calculated with the use of the Friedewald equation. Apo B and apo A1 were measured with the use of rate immunonephelometry. | Both diets were effective in significantly reducing TC and LDL concentrations during the weight-loss period. Subjects who consumed the low-fat diet during weight loss experienced a significant decrease in HDL. No significant change in HDL was observed in the subjects who consumed the moderate-fat diet. Total:HDL ratio significant decreased in the moderate-fat diet at end of the weight loss period. LDL:HDL significant reduction in the moderate-fat diet and non-significant reduction in low-fat diet at end of the weight loss period. Both groups showed significant decreases in TGs during the weight-loss period. Changes in apo B mirrored changes in LDL, with both groups with significant reductions during weight-loss period. Although only the low-fat diet group experienced significant decrease in HDL, both diet groups showed a reduction in apo A1. | LOW |
| Rajaram et al. (2001), US | RCT cross-over | Pecans | 72 | 5 | n = 24 randomised. n = 23 completed study. | 25 - 55 | >30 | Healthy adults without a history of coronary heart disease, not taking medication that interfered with lipid metabolism. | Intervention group consumed 72 g of pecans each day on the pecan diet. Control group consumed Step 1 Diet. | The diets were isoenergetic but the % of energy from fat was higher in the pecan (39.6%) than in the Step I diet (28.3%). Pecans did not replace a given food or fat in the step I diet, but a portion of the entire diet, accomplished by reducing the portion size of all items on the menu of the Step 1 diet by 1/5th (reducing total energy 20%) to accommodate the pecans which were served plain, salads, gravies, shakes and as toppings. | Dietary compliance was assessed by measuring plasma fatty acids at the end of each dietary period. Fasting blood samples taken on two alternate days at the end of the run-in phase and end of the 2 diet periods. TC, LDL, HDL and TGs were measured using enzymatic assays on an autoanalyser. Apo A1 and apo B were measured by rate immunoephelometry. Lp(a) was determined turbidimetrically. | Significantly differences found between Step I diet and pecan-enriched diet for: TC-C, LDL, HDL, LDL:HDL ratio, and TG.  These changes did not change when adjusted for a <1% reduction in body weight with the pecan diet. | HIGH |
| Rajaram et al. (2009), US | RCT cross-over | Walnuts | 42.5 | 4 | N = 27 randomised. n=25 completed study. | 23 - 65 | 24.8 | Normal to mildly hyperlipidaemic adults. Exclusion: consumed nuts or ate fish >2 times/week, drank caffeinated beverages >3 times/day, drank alcohol>2 drinks/week, had food allergies, smoked, history of chronic or metabolic diseases, regularly used medication or supplements known to affect blood lipids, serum cholesterol >7.76mmol/L, serum TG >3.33mmol/L. | Intervention diet consisted 42.5 g walnuts/10.1 megajoules) or a fish diet (113 g salmon twice/week), in a 3X3 Latin square design (6 diet sequences). A control diet with no nuts or fish).  3 isoenergetic diets for 4 weeks each. | All meals were prepared in the university metabolic kitchen. Participants ate breakfast and dinner at the facility Sunday-Friday. Daily lunch, snacks and Saturday meals were packaged for consumption at the participant’s discretion. | Compliance achieved by weighing foods to the nearest gram prior to serving, and at least one investigator was present at all meal times. Participants were also required to maintain daily diaries to record any deviations from study protocol. Erythrocyte membrane fatty acid composition was determined at the end of each diet period as a biochemical marker of compliance. A 12-hr fasting blood samples collected on two alternate days at the end of each diet period including baseline. TC, LDL, HDL, and TG (21, 22) were measured by using enzymatic colorimetric assays. | TC and LDL, reduced compared to control diet. HDL and TG had a significant difference in comparison of fish diet and other diets, but not for walnut and control. | HIGH |
| Rajaram et al. (2010) Sabate et al. (2003), US | RCT cross-over | Almonds | I1: 43 (10% energy)  I2: 85 (20% energy) | 4 | n = 27 health adults. n= 2 WD. n = 25 completed the study.  . | 41 ± 13 | <30 | Without history of hypertension, arteriosclerosis or metabolic diseases, had any weight change 6 months before the study, had BMI >30 kg/m2, fasting serum cholesterol < 15th or >90th percentile for age, sex and race, fasting TG >2·26 mmol/l (>200 mg/dl), were chronically ill or taking medications, consumed nuts frequently (>2 times/week), had erratic exercise habits or were athletes that had rigorous exercise routine, were smokers or drank alcohol >2 times a week. | Two intervention groups consumed either a low-almond diet (10%) or a high-almond diet (20 %) of the total energy replaced with almonds. Control group consumed a cholesterol-lowering control diet without nuts ( < 30 % energy from fat). | Isoenergetic diet. | Compliance by serving almonds either during breakfast or dinner and were eaten under the supervision of a senior investigator. In addition, subjects maintained a diary in which they recorded any deviations from the study diet protocols and this was reviewed on a weekly basis by a senior investigator. Dietary intake by highly controlled feeding protocol. On Sunday through Friday of each week, subjects ate breakfast and dinner at the Loma Linda University Metabolic Kitchen. Lunch meals and all Saturday meals were packaged for consumption away from the Metabolic Kitchen. TC, LDL and HDL and TGs were measured by enzymatic coloimetric assays on an autoanalyser. Serum concentrations of apo A and apo B were established with the use of a nephelometer. E-selectin was measured by ultra-sensitive ELISA. | Significantly lower levels with higher almond intake in: TC, LDL, apo B, LDL:HDL, and apo B:apo A. No significant effect found for: HDL, TG, and apo A. A high-almond diet had significantly lower E-selectin than the control and low-almond diets, but there were no differences between low-almond and control diets. For each 1 % increase in energy from almonds, there was an estimated decrease of 0·18 μg/l in E-selectin. Serum E-selectin significantly decreased with progressively higher amounts of almonds in the diet, but there was no clear dose response. | HIGH |
| Rock et al. (2017), US | RCT parallel | Walnuts | 25-42 (15% energy) | 26 | n=100 non-diabetic overweight and obese men and women, 51 control group and 49 walnut group. n=3 WD. n=97 completed the study. | ≥21 (C:53.3 ± 1.4, I: 52.2 ± 1.6) | 27- 40  (C: 91.1 ± 2.3), I: 90.9 ± 1.8) | Without history or presence of a comorbid diseases where diet modification and increased physical activity may be contraindicated, self-reported pregnancy or breastfeeding or planning a pregnancy within the next year, involved in another diet intervention study or weight loss program, and having a history or presence of a significant psychi-atric disorder or any condition that would interfere with participation in the trial. | Intervention group assigned to walnut enriched reduced energy diet with walnuts either 42 g/d (1.5 oz) diet prescriptions that were ≥1500 kcal/day, or 28 g/d (1 oz) for diet prescriptions <1500 kcal/day, all within their energy-reduced diet plan (~15% of total energy intake). Control group assigned to the standard reduced-energy- density diet. They were asked to refrain from eating any nuts (and products containing them) for the duration of the study. | Reduced-energy diet. | Compliance by quering participants about walnut consumption for the previous week when the walnuts were distributed. Dietary intake by a web-based planning and tracking program that enabled tracking kilocalories was encouraged. TC, TG, and HDL were measured by Arup Laboratories. LDL values were calculated by the Friedewald equation. BP was averaged from two sitting BP measurements. | Both groups decreased their SBP at 3 months, but only those in the walnut group maintained a lower SBP at 6 months compared to baseline. Participants in both study groups also decreased their DBP at 3 and 6 months. There was no significant group by time interaction observed in the BP. Participants assigned to the walnut group, but not the control group, had a reduction in TC. TGs decreased in the standard diet group at 3 months and in both groups at 6 months. HDL did not change significantly between baseline and 6 months in either of the diet groups. | HIGH |
| Rock et al. (2020), US | RCT parallel | Pistachios | 42 (18% energy) | 17 | n=100 non-diabetic overweight/obese adults, 50 per group. n=7 loss to follow up. n=93 completed the study. | Mean: 55.6 (≥ 21) | 27 - 40 (Mean: 32.8) | Without severe disability, history or presence of a comorbid disease, self-reported pregnancy or breastfeeding or planning a pregnancy within the next year, currently involved in another diet intervention study or weight loss program, having another member of the household participating in the study, and having a history or presence of a significant psychiatric disorder or any condition that would interfere with participation in the trial. | Intervention group consumed pistachios (roasted, shelled and unsalted kernals) packaged individually into 1.5-oz. portions. Both intervention and control groups were advised to reduce energy intake by 500-1000kcal/d below estimated maintenance requirements and aim for weight loss. | Achieve a deficit of 500-1000kcal/d. | Compliance was assessed via a self-monitoring form that participants completed and submitted each time they received a new supply of pistachios. Dietary intake by three 24-hour recalls. The lipid panel was measured by enzymatic colorimetric test. LDL was calculated from the total and HDL values using the Friedewald equation. | The pistachio group (but not the control group) exhibited a significant reduction in both SBP and DBP, although a significant difference between the groups was not observed at either time point. TC and TG levels decreased in both groups, the reduction did not reach statistical significance. HDL levels did not change significantly over the course of the study in either group, and LDL levels declined in both groups but did not reach statistical significance. | HIGH |
| Ros et al. (2004), Spain | RCT cross-over | Walnuts | 40 - 65 | 4 | n = 21 recruited. n = 20 completed the study. | 25 - 75 (Mean: 55) | NR | Non-symptomatic non-smoking men and women with hypercholesterolemia, absence of chronic disease or secondary hypercholesterolemia. | Intervention group consumed isoenergetic diet enriched with walnuts. Control group consumed Mediterranean-type diet. | Walnuts contributed 18% of total energy and replaced 32% of the energy obtained from MUFA in the control diet. Walnuts were consumed as snacks or with meals in desserts or salads. The diets were composed of natural foods, vegetable products and fish were emphasized, and red and processed meats, whole-fat dairy products, and eggs were limited. | Compliance with the diets was assessed through a 7-day food diet-recalls and serum levels of gamma-tocopherol which is a biological marker of adherence to the walnut diet. Fasted blood samples were obtained at baseline and at the end of each diet period. Cholesterol and TG were measured with enzymatic procedures as was HDL after precipitation from plasma. VLDL and LDL were separated by ultracentrifugation. Homocysteine by fluorescence polarization immunoassay. | Significant effects of walnut consumption found for: TC and LDL. Significant increases in endothelial dependent vasodilation and LDL gamma tocopherol with walnut consumption, whilst significant decreases in VCAM-1 and LDL alpha tocopherol following walnut consumption were also found.  No significant effect of diets on: LDL:HDL ratio, SBP, DBP, HDL, and TG. | HIGH |
| Ruisinger et al. (2015), US | RCT parallel | Almonds | 100 | 4 | n=50 (almond group, n=22 and non almond group, n=26). n=2 WD due to inability to consume almonds daily and concerns about the additional energy intake. n=46 completed the study. | 18 - 78 (C: 60.0 ± 10.4, I: 59.3 ± 11.7) | C: 29.8 ± 4.8 I:28.6 ± 3.9 | Without LDL levels <70 mg/dL, the use of lipid-lowering agents other than statins, adherence to specialized diets, nut consumption greater than twice weekly, nut allergies, liver disease, chronic renal or coronary heat disease, and alcohol or illicit drug dependence. Postmenopausal women if not taking hormone replacement therapy and females of child-bearing using an effective form of contraception were allowed to participate. | Intervention group received 100 g of almonds/day and ATP-III TLC diet counselling. Control group received or ATP-III TLC diet counselling only. | All subjects were instructed to maintain their current diet. | Compliance by counting unconsumed packages of almonds. Dietary intake by 3-day food records. Fasting plasma concentrations of LDL, HDL and TG were directly measured by Atherotech Diagnostics Lab using the inverted rate zonal, single verticle spin, ultra-centrifugation based Vertical Auto Profile method. | The almond group had notable improvements in LDL and TG, but these did not achieve statistical significance. There were no differences in TC and HDL between the 2 groups from baseline. | HIGH |
| Sabate et al. (1993), US | RCT cross-over | Walnuts | 84 | 4 | n = 24 participants selected, n = 19 entered the experimental phase, n = 18 completed the study | 21 - 43 | 18.7 - 30.6 | Healthy men, aged 21-43 years, weighing 60-103kg. | Intervention group consumed walnut diet while control group consumed reference diet. | Walnuts, in the context of Step I diet (31% fat diet). | Compliance monitored by supervising breakfast and dinner. Fasted blood samples were drawn on day 2 of the run-in period, days 30 and 32 and on days 58 and 60 at the end of each diet period. Serum and lipoprotein subfractions were analysed for cholesterol and TG content using an auto analyser and commercial enzymatic kits. HDL was isolated using dextran sulphate and LDL was measured with the Friedewald calculation. All measurements were standardized according to the Lipid Standardization Program of the Centre’s for Disease Control and Prevention and the National Heart, Lung and Blood Institute. BP was measured twice during the run-in period, weekly during the first 2 weeks of each diet period and twice during the last week of each diet period on seated subjects after 5min rest. | Significant differences between groups in: TC, LDL, HDL, and LDL:HDL ratio. Non-significant differences in TG and BP. | HIGH |
| Sabate et al. (2005)  Torabian et al. (2010), US | RCT cross-over | Walnuts | 35.2 (12% energy) | 26 | n = 94 enrolled, n = 90 completed study. n=87 included for analyses. | 30 - 72 | Mean: 26.1 ± 3.3 | Healthy adults with weight change <1kg prior 6/12, BMI<35kg/m2, habitual diet including nuts <1/7. Without metabolic disorder affecting weight (diabetes, hypothyroidism), aversion or known allergy to nuts. | Intervention group consumed 28-56 g walnuts (~12% energy intake). Control group consumed no walnuts or substantial amounts of any other nuts. | Habitual diet. | Compliance maintained by 24-hr dietary recalls collected seven times during 6 months (two weekends, five weekdays). RBC Alpha-linolenic acid membrane concentration measured at 6 months as a marker of adherence. Subjects requested to return unused walnuts. Dietary compliance was ~95%. Mean daily walnut consumption was 35 g during walnut diet. Walnut diet had greater daily EI (557 kJ). Plasma total cholesterol, HDL and TG were determined using methods previously reported, and LDL was calculated using the Friedewald formula. | LDL changes were nearly significant but no change seen in either HDL or LDL:HDL ratio. | HIGH |
| Salas-Huetos et al. (2018), Spain | RCT parallel | Mixed nuts | 60 (walnuts:30 g, almond:15 g and hazelnut:15 g) | 14 | n=119 healthy adult men. n=21 lost to follow-up. n=98 patients completed the study.  n=106 included in analysis. | 18 - 35 | I: 23.55 ± 2.84, C: 24.09 ± 3.43 | Without frequent consumption of nuts or a known history of allergy, use of plant sterol or fish oil supplements and multivitamins, vitamin E, or other antioxidant supplements, history of reproductive disorders or vasectomy; current smokers, use of medications for chronic illness, or use of illegal drugs. | Intervention group consumed Western-style diet enriched with 60 g of a mixture of nuts/day and the control group was fed the usual Western-style diet avoiding nuts. | Western-style diet. | Compliance was assessed by counting the empty sachets of nuts returned by the participants. Dietary intake by 3 day dietary records, including 2 workdays and a weekend day. TC, HDL, LDL and TG were determined by routine laboratory tests using standard enzymatic automated methods. | No significant differences for TC, LDL, HDL and TG with the control and intervention group on treatment effect. | HIGH |
| Sales et al. (2008) Akuamoah- Boateng et al. (2007), Ghana, Brazil, US | RCT parallel | Peanuts | 76 (30% energy) | 8 | n = 129 (control: n = 32, peanut: n = 32, safflower: n = 33, olive oil: n = 32) | 18 - 50 (Mean:  25.05 ± 5.58) | 18 - 25 (Mean: 22.09 ± 2.58) | Healthy adults, nonsmokers, weight stable without diabetes or regular medication use. Participants were from Brazil, Ghana and the US. | Intervention group consumed a daily test meal for 8 weeks, control group did not receive test meal. The test meal was a milkshake which provided 690 – 769kcal and contained either peanut, safflower or olive oil. | Milk shake made with skimmed milk, sugar, flour mix, and fruit added peanut, safflower oil or olive oil according to the test group. | Compliance by a 3 day diet records at baseline, 4 and 8 weeks. TC, LDL, HDL and TG levels were assayed from venous samples (does not state if fasting). Total cholesterol was checked by finger puncture during the screening process with an Accu-Check InstantPlus monitor. Plasma total cholesterol, HDL cholesterol, LDL cholesterol, and TG levels were analysed with a COBAS MIRA clinical chemistry analyser. BP was measured with an automatic monitor (does not state if seated/supine). | No significant effect of peanut oil on lipid parameters: TC, LDL, HDL, TG, SBP, and DBP. Significant reductions of SBP in participants from Ghana consuming peanut oil. | LOW |
| Sapp et al. (2021), US | RCT cross-over | Peanuts | 28 | 6 | n=51 adults with elevated fasting plasma glucose. n=1 WD. n=50, 25 per group completed study.. | 42 ± 15 | 28.3 ± 5.6 | Without diabetes, taking glucose, lipid, or blood-pressure lowering medications, or taking antibiotics ≤6-weeks prior to enrolment; history of cardiovascular disease, stroke, liver, kidney, autoimmune disease or inflammatory, taking supplements and botanicals were excluded unless willing to abstain during the course of the study. Finally, pregnant or lactating women, individuals consuming >14 alcoholic beverages/week or those who had lost >10% of their body weight in the previous 6-months were excluded. | Intervention group consumed 28 g/d of dry-roasted, unsalted, skinless peanuts. Control group consumed six low-sodium whole grain crackers (28 g) and one slice (19 g) of low-fat pre-packaged American cheese. | Participants were asked to consume the study food. | Compliance adherence was assessed bi-weekly by the Metabolic Kitchen Manger based on daily adherence checklists completed by the participants. Diet quality was assessed using the Healthy Eating Index (HEI)-2015, which consists of thirteen components including nine "adequacy‟ components (whole grains, total fruits, dairy, etc.) and four "moderation‟ components (fats, sodium, refined grains and sugars). Blood samples were drawn into serum separator and sodium fluoride/potassium oxalate tubes. A 5-minute seated rest, a SphygmoCor ECEL was used to assess peripheral and central BP in the seated position. | No differences were observed between the conditions for TC, LDL, HDL, TG, peripheral or central BP or measures of arterial stiffness. | HIGH |
| Sauder et al. (2014) Sauder et al. (2015), US | RCT cross-over | Pistachios | 59-128 (20% energy) | 4 | n=34 subjects with self-reported diagnosis of type 2 diabetes. n=4 WD. n=30 completed the study. | 30 - 75 (Mean: 56.1 ± 7.8) | 18.5 - 45.0 (31.2 ± 3.1) | Without insulin use, self-reported history of chronic disease other than type 2 diabetes, history of bariatric surgery, major surgery in the prior 6 months, nut or latex allergies, and use of tobacco, daily aspirin, anti-inflammatory medications, oral steroids, hormone replacement therapy, or anti-hypertensive medication. | Intervention group consumed a modification of the control diet wherein low-fat or fat- free snacks (i.e., pretzels, string cheese, etc.) were replaced with roasted pistachios that provided 20% of daily energy (range: 59–128 g). Control group consumed the American Heart Association’s Therapeutic Lifestyle Changes diet. | American Heart Association’s Therapeutic Lifestyle Changes diet. All meals and snacks were prepared in the Metabolic Kitchen at the Pennsylvania State University Clinical Research Center. | Compliance questionnaires regarding daily consumption of study and non-study foods, which were reviewed by the dietary staff, and returned food containers were checked for uneaten study foods. TC and TGs were measured by enzymatic procedures. HDL was estimated according to the modified heparin–manganese procedures. LDL was directly measured with a chromogenic reaction after removal of all non-LDL. Plasma concentrations of E-selectin, ICAM, and VCAM were measured via enzyme-linked immunosorbent assays (ELISA) kits. Brachial FMD and peripheral arterial tonometry (PAT) tests were performed to assess endothelial function. HRV was assessed in the resting state and during 2 acute stress tasks (mental arithmetic and hand cold pressor, described above) at the end of each diet period. BP automatically measured using an ambulatory BP monitor. | TC, the ratio of TC to HDL and TG were significantly lower following the pistachio diet compared to the control diet. There were no differences between treatments for HDL and LDL. There were no significant differences between treatments for any measures of endothelial function. Both RMSSD (square root of the mean squared differences of successive R‐R intervals) and high‐frequency power, which reflect parasympathetic activity, were significantly higher following the pistachio diet. Twenty-four-hour SBP was significantly lower following the pistachio diet compared to the control diet. DBP did not differ between treatments. | HIGH |
| Sheridan et al. (2007), US | RCT cross-over | Pistachios | 57 – 85 (12% energy) | 4 | N = 20 enrolled, n= 15 (11 M & 4 F) completed. | 36 - 75 (Mean: 60 ± 3) | 28 ± 0.9 | With TC>210mg/dL; TC 246 ± 6mg/dL, TGs 141 ± 11mg/dL, SBP 129 ± 4mmHg, DBP 84 ± 3mmHg. Excluded if treated for hyperlipidemia, hypertension, diabetes mellitus, kidney or liver disease, <18yrs, known food allergies, smokers, consuming >3alcoholic drinks/week, women on HRT. | Intervention group had 15% energy intake from pistachio nuts (~2-3 ounces/day: 57 – 85 g) – instructed to substitute nuts for normally consumed high fat snacks or fat calories + normal regular diet. Control group consumed normal, regular diet. | Habitual diet. | Compliance record by a 3-day food diary before baseline and before each weekly meeting with dietitian. Also recorded one- day food records for each week of pistachio diet – analysed for compliance. Subjects asked to return pistachio storage bags at each visit. Blood lipids measured at baseline, week 2 & week 4 of intervention. All blood samples were analysed within one day at American Medical Laboratories, Inc. (Chantilly, VA). BP measured after 5 minute seated rest. Lipids measured after overnight fast. | Significant effects of pistachio diet on LDL:HDL and HDL No significant effect for: TC, LDL, TG, SBP and DBP. | HIGH |
| Solá et al. (2012), Italy | RCT parallel | Hazelnuts | 30 | 4 | n = 113; n=0 lost to follow up reported, but n=11 excluded from per protocol analysis), (control group, n=28) (cocoa+hazelnut group, n=28). | 43 - 65 | <35 | Men and women with pre-hypertension (defined as SBP: 140–159 mm Hg or DBP: 90–99 mm Hg) and hypercholesterolemic, LDL between 3.35 mmol/L- 4.88 mmol/L and at least one cardiovascular disease risk factor such as age (men≥45 years; women≥55 years), smokers, HDL concentration of <1.0 mmol/L & <1.18 mmol/L in men and women, respectively; family history of premature cardiovascular disease. Without diabetes mellitus, any chronic disease, current hypolipemic treatment, TG>3.97 mmol/L and BMI>35 kg/m². | Control group consumed cocoa cream. Intervention group 1 consumed cocoa + hazelnut cream (+30 g/d hazelnuts). | Isocaloric diet | Compliance by a 3 day food records at beginning & end of intervention +24 hour recall at 2 weeks within the intervention period. Compliance was monitored by empty wrapper counting and any non-consumed doses were collected at follow-up clinical visits. BP measured seated using an automatic sphygmomanometer by trained personnel. Lipids assessed using fasted blood samples. TC, TG, HDL, apo A-1 and apo B-100 in serum were performed using standard methods in an autoanalyser (Beckman Coulter-Synchron, Galway, Ireland). LDL was calculated by means of the Friedewald formula. | Overall no differences between control and hazelnut only intervention group for SBP, TC, LDL, HDL, TG but with the exception of DBP. | HIGH |
| Somerset et al. (2013), Australia | RCT parallel | Macadamias | Not specified (MUFA 50% of total fat) | 10 | n = 64 randomised and completed study (breakdown by group not reported). | 26 - 55 | 27 - 40 | Healthy, not using medication to control blood sugar. | Intervention group consumed a macadamia enriched version of their usual isocaloric diet (replacing saturated fat with macadamia nuts, paste and oil, with the goal of 50% fat as monounsaturated). Control group consumed usual isocaloric diet. | For the intervention group, energy intakes of usual (baseline) diets were calculated from multiple 3 day diet diaries, and saturated fatty acid was replaced with MUFA and adding macadamia nuts to the diet. | Compliance assessed by a 3 day diet diary and 24 hr recall (at 0, 4, 6, 10 weeks). Serum lipids were collected after an 8 hour fast. Serum cholesterol, HDL, and TGs were analysed using a major local private pathology service (Queensland Medical Laboratories, Brisbane) using standard methods and LDL calculated using the Friedewald equation. BP measured seated after 10 min rest. | Significant differences between groups not reported. Significant change over time in nuts group found for TC.  Significant change over time in control group found for: HDL:LDL. No significant change over time found for: SBP, DBP, HDL, LDL, and TG. No significant change in VCAM-1. Significant reductions in nut group in: ICAM-1, leptin, and significant increases in flow mediated vasodilation. | LOW |
| Spaccarotella et al. (2008), US | RCT crossover | Walnuts | 75 | 8 | n = 22 enrolled, n = 21 completed. | 55 -75 (Mean: 65.9) | NR | Healthy, non-smoking men aged 45-75yrs total prostate specific antigen≥2ng/mL, no clinically diagnosed prostate cancer. Without allergies to nuts, use of prescription and non-prescription preparations known to alter prostate specific antigen/ hormone levels/ BP/lipids. Not permitted to take vitamin E supplements within prior 2/12 to baseline. Weight: 84.8(2.9) kg; HDL: 1.36(0.06); mmol/L, TGs: 1.31(1.01) mmol/L; LDL 3.11(0.15) mmol/L and LDL:HDL ratio: 2.37 (0.16). | Intervention group consumed 75 g shelled unroasted English walnuts/day (~24% energy intake based on 2000 calorie/day, 490 calories, 48.9 g fat) + usual diet.  Control group consumed usual diet (average American diet) – instructed to maintain their baseline weight and activity level throughout study. | Isocalorically replaced another fat source in their usual diet with the walnuts. | Compliance to reinforce food records reviewed by a registered dietitian and individualized strategies for weight maintenance were suggested. Food record data were analyzed for intakes of energy, fat, tocopherols and other nutrients using Nutrition Data System for Research software. BP & weight at weeks 1,2,3,4,6 and 8. Twelve hour fasting lipids (TC, HDL, LDL, LDL:HDL, TGs). Enzyme kits were used to analyse HDL. LDL was calculated using the Friedewald equation. BP was measured seated after a 5 minute rest (average taken of 2 measures). | No significant treatment effects on blood lipids or BP. | HIGH |
| Spiller et al. (1998), US | RCT parallel | Almonds | 100 | 4 | n = 48 recruited. n = 45 completed (almond: n = 18, olive oil: n = 15, control: n = 12). Men (n=12) and women (n=33). | Mean: 53 ± 10 | NR | With hyperlipidemia, mean weight = 66 ± 13 years. Without on medications known to alter lipids and for heart disease, diabetes, other major diseases, and any known food allergy. | Intervention group consumed 100 g/d raw unblanched almonds supplied whole and ground. Control group was provided 85 g/d of cheddar cheese and 28 g/d of butter and 21 g/d of rye crackers to match the protein and carbohydrate of the almond diet. | During the intervention period diet was matched for carbohydrate, protein, and total fat content. In each group 650 calories a day were added to the background diet and about 450 calories were supplied by either almonds and cheese (control diet) as the primary sources of fat. | Compliance with a 3-day food records collection on two days during the baseline week and in the last week of the four week intervention period. Random 24hr dietary recalls and phone interviews were conducted during the study. Subjects met with the investigators after 2 weeks to discuss experiences with the diet. Fasting blood samples were drawn on two days during the baseline week and in the last week of the four week intervention period. TC, HDL and TGs were measured from fresh plasma using analytical procedures that met the Lipoprotein Standardization Program of the Centers for Disease Control and National Reference System for Choelsterol. | TC and LDL were different between the 3 groups after 4 weeks. No differences between groups in any other lipid parameter. The almond based diet significantly reduced TC, TC:HDL ratio without changing HDL levels or TG levels. TC and HDL increased significantly in the control group. | HIGH |
| Sweazea et al. (2014), US | RCT parallel | Almonds | 43 | 12 | N=24 commenced the study. N=21 completed the study (n=3 WD, n=1 control participants, n=2 intervention. participants). Analysed control group (n=11) and intervention group (n=10). | 25 - 75 | C: 33.5 ± 8.8 I: 37.2 ± 7.8 | Adults (males and females aged 25-75 years) with type 2 diabetes (diagnosed at least 6 months previously), not receiving insulin therapy. Participants receiving medications (statins, anti-hypertensives, oral hypoglycaemic agents instructed not to alter dosage throughout trial). | Intervention consumed usual diet, usual physical activity, participants provided with 1.5oz (43 g) almonds/day. Control group with usual diet, usual physical activity. | Usual diet. | Compliance assessed by a 3 day diet record was maintained. BP measured using a digital monitor (Medline Automatic Digital BP monitor, Medline Industries Inc, Mundelein, IL). Blood lipids were measured following an overnight fast using enzymatic colorimetric assays with specific reagents from Roche Diagnostics for TC, TGs, HDL and LDL. | Following the 12 week intervention there was no significant difference between control and intervention groups for: SBP, DBP, TC, LDL, HDL and plasma TGs. | HIGH |
| Tamizifar et al. (2005), Iran | RCT crossover | Almonds | 25 | 8 | n = 35 hyperlipidemic subjects. n = 30 completed (M: 17). | Mean: 56 ± 6.1 | Mean: 24.1 ± 4.5 | With mild hypercholesterolaemia (TC>220 mg/dl). Without cardiovascular disease, diabetes mellitus, taking lipid- lowering agents, or had TC≥350 mg/dl and TG<400 mg/dl. | Intervention group consumed 25 g/d almond powder. Control diet consisted of a cholesterol-lowering diet without nuts, nut butter or margarines, and nut oils. Washout period of 5-7 days. | Diets were based on NCEP Step I diet. Diets were matched for calories and fat content. | Compliance monitored by diaries by participants to record side- effects or deviations from the experimental diets. A 3-day food records were used to assess and compare fat content of each subject’s diet before & during intervention. During each study visit, subjects met individually with a nutritionist to discuss compliance. Almond powder bags were prepared and pre-weighed in the pharmaceutical department of the university. Blood lipids were measured on day 1 and 3 of the baseline diet study, 28th day of the first treatment phase and second treatment phase, after a 12 hour fast. TC, HDL and TGs were analysed, LDL calculated. | Significant differences in changes between groups in TC and LDL.  No significant difference between groups in TG: HDL ratio. | HIGH |
| Tan et al. (2013), US | RCT parallel | Almonds | 25 | 4 | n = 150 commenced the study. n = 137 completed the study (n = 13 WD from the study). Final numbers by group: CL: n = 27, BF: n = 28, MS: n = 28, LN: n = 26, AS: n = 28. | CL: 28.7±9.6, BF: 32.9±11.5,  MS: 27.8±10.7,  LN: 29.3±13.5,  AS: 29.0±11.9 | 24.1 | With greater risk of type 2 diabetes due to being either overweight or obese (BMI > 27kg/m2), or normal weight with a strong family history of type 2 diabetes, both males and females, not allergic to nuts and test meals, non-diabetic, not taking any medication known to affect glycemia, metabolism or appetite, regular breakfast eaters and weight stable. Participants were aged between 18 – 60 years. | Intervention group 1 (breakfast meal) consumed 43 g of almonds with their daily breakfast. Intervention group 2 (lunch meal group) consumed 43 g of almonds with their daily lunch. Intervention group 3 (morning snack group)consumed 43 g of almonds as a morning snack (between breakfast and lunch). Intervention group 4 (afternoon snack group) consumed 43 g of almonds as an afternoon snack (between lunch and dinner). Control group were asked to avoid all nuts and seeds during study period. | Acute feeding session was conducted one week after baseline and at week 4, where participants were served standardised breakfast and lunch meals including almond allowance. | Compliance by completion of a 24-hour dietary recall using a multi-pass interview method with a dietitian (at weeks 1, 2 and 4). Compliance also assessed via fasting alpha-tocopherol concentrations. BP, fasting lipids (method of measurement not stated). | After 4 weeks, almond consumption had no significant effect on: SBP, DBP, TG, TC, LDL, and HDL. | HIGH |
| Tapsell et al (2004), Autralia | RCT parallel | Walnuts | 30 | 26 | n = 58 randomised. n = 55 completed the study (control: n = 20, low fat/modified: n = 19, walnut: n = 16). | Mean: 59.3 ± 8.1 | Mean: 29.2 ± 2.6 | Men and women with type 2 diabetes. Subjects had been diagnosed with type 2 diabetes for at least 1yr, not on insulin therapy, with major illness, known food allergies or inadequate English speaking skills. | All groups with dietary advice targeting <30% energy as fat: Intervention 1 with low fat/modified fat (using exchange lists inclusive of fatty acid considerations. Intervention 2 with walnut inclusive (low fat/modified fat approach including 30 g of walnuts supplied/day). Control with usual practice (low fat, control). | Each group was given advice on the number of servings of carbohydrate-rich foods and type of protein-rich foods (low-fat) and oils/spreads (MUFA or PUFA rich) and to include 2 fish meals/week. The modified low-fat and walnut groups were advised on the number of servings of protein-rich foods and oils/spreads, using an exchange list and provided 30 g of walnuts to consume each day. | Compliance of dietary data was assessed by a validated diet history method and a 3-day food record at each time point (0,3 and 6mths). As another measure of dietary compliance, changes in biomarkers of PUFA intake (erythrocyte fatty acids) were assessed. Blood lipids were measured at 0, 3 and 6 months. All blood samples were analysed at a quality assured pathology laboratory using standard analyses techniques. | TC levels of the walnut group were lower than the other two groups at each time point. LDL decreased significantly over time in the walnut group with no changes seen in the other two groups. HDL levels increased significantly in all groups. Changes in HDL: TC ratio for the modified-fat groups were significant for the first 3 months. There were no significant differences between groups for changes in TGs. | HIGH |
| Tapsell et al. (2009), Australia | RCT parallel | Walnuts | 30 | 52 | N = 50 randomised, n=35 completed the study. | Mean: 54 ± 8.7 | Mean C: 33 ± 4.0, I: 33.2 ± 4.4 | With type 2 diabetes, NIDDM, BMI >25 and <32kg/m2, WC >102 for men, WC >94cm for women. Without major illness, food allergy, inhibitory habits, illiteracy and/or poor English language. | Intervention group included 30 g walnut/day 10% energy from MUFA, and 10% energy from PUFA. Control group consumed 30% energy fat (10% energy from SFA, 12% energy from MUFA, 5% energy from PUFA), 20% energy from protein, 50% energy from carbohydrate. | Participants (n=50) received dietary advice on isocaloric diet, ~2000kcal/day, adjusted for individual requirement. | Compliance assessed by a core food serves advice given by qualified dietitian with dietary modelling to compare advice given matches targets. A 3 month visits for advice and measurement and regular newsletters to encourage adherence. Subjects completed a validated diet history interview and 3-day food records to assess dietary intake at baseline and every 3 months. Fasting blood samples collected. | No significant effect on: TC, HDL, LDL, and TG. | HIGH |
| Tapsell et al. (2017), Australia | RCT parallel | Walnuts | 30 | 52 | n=377 verweight and obese adults, control (n=126), intervention (n=125) and intervention+walnuts groups (n=126). n=178 completed the study. Analysed (intervention+walnut group: n=64, C: n=52). | 45 (37–51) | 25 - 40 | Without severe medical conditions impairing the ability to participate in the study or thought to limit survival to 1 year, illegal drug use or regular alcohol intake associated with alcoholism (>50 g/d), or other major impediments to participation. Were able to communicate in English. | Intervention group 1 were provided interdisciplinary advice, intervention group 2 intervention + food supplement (walnuts) and control group usual care with a general guideline- based diet and exercise advice. | General advice based on the Australian Guide to Healthy Eating. | The study was testing an approach applicable to primary care, so the analysis was conducted on an intention-to-treat basis rather than on compliance to treatment. Dietary intake was assessed using a diet history interview. SBP and DBP were measured at 0 months, 3 months and 12 months using the Omron BP-203RPEIII VP- 1000 device. Fasting blood lipids (TC, LDL, HDL, TG) were assessed through a registered pathology service (Southern IML Pathology) quarterly. | SBP decreased between baseline and 3 months but then remained unchanged. TC and LDL concentrations had a significant reduction at 3 months and at 6 months compared to baseline. The TC: HDL ratio decreased particularly after 6 months, while the HDL values first dropped at 3 months then significantly increased from baseline to 12 months. The group effect for TC showed a significant lower overall mean for the walnut group compared with controls and intervention group 1. | HIGH |
| Tey et al. (2011), New Zealand | RCT parallel | Hazelnuts | 42 | 12 | n = 124 randomised n = 118 included in intention-to-treat analysis (I: n =32, C: n = 29). | Mean: 37.4 ± 14.0 (18 - 65) | ≥ 30 | Healthy males or females aged 18- 65yrs. Without BMI: ≥30 kg/m, people with asthma, women pregnant or breastfeeding, people with chronic disease such as cancer, heart disease or diabetes, people with food allergies or food aversions. | Intervention group consumed hazelnuts (42 g). Control diet (no additional food) for 12 weeks. A 2 week run-in period was completed. | Habitual diet. | Compliance was measured by weighing returned bags of snacks. A 3 day weighed diet record collected at baseline and during study. Blood samples collected following 12 hour fast TC, HDL, and TGs were determined by enzymatic methods, LDL was calculated. | No significant effect on TC, LDL, HDL, and TG. In PP analysis of people with compliance ≥70% only there was tendency for lower plasma TC in hazelnut group compared to control group. | HIGH |
| Tey et al. (2013), New Zealand | RCT parallel | Hazelnuts | 30 or 60 | 12 | n= 107 recruited and included in final. analysis (I: n = 37, I1: n = 33, I2: n = 37). | Mean: 42.5 ± 12.4 | Mean: 30.6 ± 5.1 | Adults (aged between 18 – 65 years), with BMI > 25 kg/m2, without major chronic disease or inflammatory disease, or aversion to nuts. | Intervention group 1 consumed 30 g/d raw hazelnuts. Intervention group 2 consumed 60 g/d raw hazelnuts and the control consumed no nuts. | Habitual diet. | Compliance by a 3 day diet record (at baseline and week 7). Participants returned uneaten nuts, and recorded nut diary. Participants were asked to collect their nuts every 3 wk and to return any nuts not eaten at those visits. A researcher reviewed all dietary records upon return for completeness and accuracy. BP was measured by using an Omron BP monitor. ICAM-1, and VCAM-1 were measured by using Quantikine ELISA Kits following the instructions provided by the manufacturer. Plasma TC, HDL, and TG concentrations were measured in all blood samples by enzymatic methods using a Cobas Mira Plus Analyzer. LDL was calculated by using the Friedewald formula (40). Apo A1 and B100 were determined by immunoturbidity by using commercial kits from Roche Diagnostics. | No significant effect of nuts compared to control on SBP, DBP, TC, LDL, HDL, and TGs. No significant effect of nut consumption on other outcomes. | HIGH |
| Tindall et al. (2019), US | RCT cross-over | Walnuts | 57-99 (18% energy) | 6 | n=45 overweight and obese men (n=25) and wome (n=20) with elevated LDL and/or brachial BP .  n=9 loss to follow up/dropped out. n=36 completed the study. | 30 - 65 (Mean: 43 ± 10) | 25 - 40 (Mean: 43 ± 10) | Without smoking, BP ≥160/100 mm Hg, and a history of myocardial infarction, stroke, diabetes, liver disease, kidney disease, thyroid disease (unless controlled with medication), gastrointestinal diseases, and inflammatory diseases; taking certain supplements or medications unless they were willing to discontinue use before enrolling, women who were lactating, pregnant, or planning to become pregnant were excluded from the study; following a vegetarian or vegan diet or had nut allergies, consumed >14 alcoholic drinks per week before screening or were not willing to maintain their physical activity habits throughout the course of the study. | Intervention group consumed 18% of daily energy from walnuts delivered as a snack (57–99 g/d, containing 5-8.8 g/d alpha linoleic acid). Control group consumed walnut fatty acid-matched diet (same amount of alpha linoleic acid and PUFA as the walnut diet but was devoid of walnuts (and walnut bioactives). Oleic acid replaces alpha linoleic acid diet (ORAD) consumed 83% of alpha linoleic acid was replaced with oleic acid and devoid walnuts. | Isocaloric weight management diet. | Compliance by assessing daily food logs that participants completed and monitoring participant daily body weight logs. All participant diets used the same 6‐day‐cycle menu. On Fridays, participants were provided with a cooler containing Saturday and Sunday meals and snacks. Serum TC and TG were directly measured using spectrophotometry at a certified commercial laboratory. LDL‐C was calculated using the Friedewald equation. The SphygmoCor XCEL system was used to assess cSBP, cDBP, cMAP, bSBP, bDBP, bMAP, AI (adjusted to a heart rate of 75 beats/min), HR, and PWV. | The WD, the WFMD and the ORAD all significantly decreased TC, LDL and HDL from baseline, but the magnitudes of change from baseline were not significantly different between groups. TC:HDL‐C significantly decreased after the WD compared with baseline, but there was no main effect of diet. TGs did not significantly change from baseline after any study diets. The WD, WFMD, and ORAD did not significantly change cSBP from baseline, and there were no significant differences between diets for the magnitudes of change or mean values. There was a main treatment effect of diet for cDBP and a significant reduction from baseline following the WD. There were no statistically significant changes from baseline, differences in magnitudes of change, or diet effects for brachial SBP, bDBP, AI, HR or PWV. | HIGH |
| PREDIMED -  Urpi-Sarda et al. (2012) (ICAM-1); Casas et al. (2017) (VCAM-1, e-selectin); Urpi-Sarda et al. (2021) (LDL, HDL, TC, TG, SBP, DBP), Spain | RCT parallel | Mixed nuts | 30 | Urpi-Sarda et al (2012): 52 Casas et al (2017): 260 Urpi-Sarda et al (2021): 156 | Urpi-Sarda et al (2012): Med+Nuts: n=175; Med+EVOO: n=178. Casas et al (2017): Med+Nuts: n=22; Med+EVOO: n=22. Urpi-Sarda et al (2021): Med+Nuts: n=92; Med+EVOO: n=93. | Urpi-Sarda et al (2012): 67 ± 6 Casas et al (2017): MeDiet+EVOO: 67.8 ± 4.8 MeDiet+nuts: 66.0 ± 5.8 Low-fat diet: 66.0 ± 7.1 Urpi-Sarda et al. (2021): Med+nuts: 67 ± 5; Med+EVOO: 68 ± 6 | Urpi-Sarda et al. (2012): 29.4 ± 3.4 Casas et al. (2017):MeDiet+EVOO: 29.7 ± 3.7 MeDiet+nuts: 30.4 ± 3.2 Urpi-Sarda et al. (2021): Med+nuts: 29.8 ± 4; Med+EVOO: 30.5 ± 4.3 | Without history of cardiovascular disease, any severe chronic illness, drug or alcohol addiction, history of allergy or intolerance to olive oil or nuts, or low predicted likelihood of changing dietary habits according to the stages-of-change model. | Intervention group consumed either mediterranian diet supplemented with extra virgin olive oil or a mediterranian diet supplemented with nuts. Control group consumed a control low-fat diet (LFD). In order to isolate the effect of nuts, the Med+nuts diet was compared to Med+EVOO | Mediterranean or low fat diet. | Dietary intake by annual face-to-face interview with the dietitian. Group sessions took place every 3 months to provide the participants with descriptions of seasonal foods, shopping lists, weekly meal plans, and cooking recipes. Soluble VCAM-1 (sVCAM), soluble ICAM-1 (sICAM-1), and E-selectins were determined using the Versa- MAPTM human custom multi-analyte profiling development system (R&D Systems, Abingdon, UK) which is also based on multiplex assays designed to measure analytes in plasma matrices. ELISA assays were performed per participant in thawed plasma using commercial immunoassays kits for soluble ICAM-1. | Urpi-Sarda et al (2012): ICAM-1 tended to decrease in the MD-VOO group and did not change in the MD-Nuts group. In the LFD group, the plasma concentrations of ICAM-1, significantly increased. Casas et al. (2017): The MeDiet+nuts group showed significant lower serum E-selectin levels, and the LFD group showed a significant increase in sVCAM-1 serum levels in both evaluations. Urpi-Sarda et al (2021): no significant differences between nut and EVOO group in outcomes | HIGH |
| Wang et al. (2012), US | RCT parallel | Pistachios | 42 or 70 | 12 | n = 90 randomised, n 86 completed the study (I1: n= 27, I2: n=29, C: n = 30) | C: 50.66 ± 9.86, I1: 51.89 ± 8.82, I2: 51.83 ± 9.37 (25 – 65) | C: 28.03 ± 4.3; I1: 28.12 ± 3.22; and I2: 28.01 ± 4.51 | With MetS (for Chinese individuals), without type 2 diabetes, acute infectious diseases or chronic diseases. | Intervention groups consumed either 42 g pistachios/day or 70 g/d. Control group consumed no pistachios. | All participants were instructed to consume the American Heart Association Step 1 diet (without nuts). | Phone call to check compliance (once per fortnight) of taking pistachio, following study instruction on diet and activity. BP was measured seated after a 10 minute rest. Lipids were measured after a 10-12 hour fast. TC, TGs, and HDL were determined using standard enzymatic methods using a Roche Hitachi™ Analyzer and RANDOX reagents. LDL was estimated from these data using the Friedewald equation. | No significant effect of pistachio consumption compared to controls on BP, TC, TG and LDL. HDL stated to not have changed significantly (data not provided). | HIGH |
| Wang et al. (2021a), China | RCT parallel | Peanuts | 56 | 12 | n=224 participants at risk of MetS were defined as having central obesity and 1 additional MetS risk factor, n=113. intervention and n=111 control group.  n=15 loss to follow up/dropped out n=209 completed intervention. | 20 - 65 (I: 46.2 ± 9.9 and C: 46.2 ± 9.9) | I: 28.4 ± 3.4 C: 27.6 ± 2.7 | Without a history of peanut and/or rice allergy, severe cardiovascular, liver, or kidney disease, stroke, type 2 diabetes, cancer, psychological disorders, or heavy alcohol use (>14 drinks/week), as well as women who were pregnant or lactating. | Intervention group consumed 2 packs of roasted lightly salted peanuts (28 g, or 1 serving/packet) per day. Control group consumed 2 packets of energy- matched white rice snack bars per day. | Maintain their background diet (aside form their intervention foods). | Dietary intake by 3 day dietary records (2 non-consecutive weekdays and 1 weekend day). Dietary intake records and physical activity recall were reviewed by the dietitians in the in-person interview. Serum HDL, LDL, TC and TGs were determined by an automatic biochemical analyser. | No significant differences in increases in HDL between the peanut group and the white rice bar control group. There were no significant effects on LDL, TC, TG and BP. | HIGH |
| Wang et al. (2021b), US | RCT parallel | Mixed nuts | 42.5 | 24 | n=131 (I: n=67, C: n=64).  n=33 did not complete study. n=95 completed the study. | 30 - 68 (I: 48.3 ± 1.9; C: 46.9 ± 1.7) | 27.0 - 35.0 (I: 31.1 ± 0.4; C: 30.7 ± 0.4) | Without thyroid disease, history of chronic disease, following special diets, consuming access alcohol, smoking, or being pregnant. Individuals enrolled in commercial weight loss programs or taking weight loss medication were excluded. In addition, individuals who experienced more than a 5-pound weight gain or loss within 3 months prior to enrolment were excluded from the study. | Intervention group consumed a daily snack of 1.5 oz. of mixed nuts. Control group consumed pretzels with the same caloric content. | Meal were prepared to provide the following macronutrient composition: mixed nut group: 30% fat, 15% protein, and 55% carbohydrate, and the nut group was provided 20% fat diet, 15% protein, and 65% carbohydrate. | Compliance maintained by daily diet checklists used by individuals to monitor the appropriate consumption of all foods, beverages, and calories each day. BP determined at baseline and 4, 12, and 24 weeks. Fasting blood samples were collected at baseline and 4, 12, and 24 weeks to determine plasma TC, LDL. Fasting plasma total and HDL and TGs were analysed spectrophotometrically. | DBP decreased significantly in both groups while SBP was not changed. Heart rate decreased significantly at Weeks 4, 12, and 24 compared to baseline in participants consuming mixed nuts, while the heart rate did not change significantly in the control group at any time point. Comparing changes in plasma lipids and the fatty acid panel between the mixed nuts and control groups did not show any significant differences. | HIGH |
| Wien et al. (2003), US | RCT parallel | Almonds | 84 | 24 | n = 65 (I: n = 32, C: n =33).  n = 52 completed the study (I: n=24, C: n = 28). Intention-to-treat analysis used. | Mean I: 53 ± 2,  C: 57 ± 2 | Mean  I: 39 ± 1,  C: 37 ± 1 | With overweight or obese adults recruited from a diabetes and cardiovascular risk reduction program for medically supervised weight loss. | Intervention group consumed a formula-based low calorie diet supplemented with 84 g/d almonds and the control group with self-selected complex carbohydrates and safflower oil. | All subjects consumed a protein-sparing formulation designed to ameliorate the loss of lean body mass with 2 vitamin/mineral supplements daily. Intervention group were provided explicit instructions on self-selecting carbohydrate from a food list that were equivalent in calories to 84 g almonds, subjects also had to consume 2 teaspoons of safflower oil/day. | Compliance maintained by completing detailed daily food and exercise records which were reviewed weekly by the study dietitian and suggestions given to enhance compliance. Excessive deviations (failure to stay within 25% of prescribed weekly energy intake) prompted additional sessions with the program psychologist and/or dietitian. Clinical assessments were made each week using calibrated office instruments. BP and heart rate were measured weekly. Plasma lipids were measured at baseline and at weeks 8, 16, and 24. Plasma was analysed using the Centers for Disease Control certified Lipid Research Clinics Protocol for TC, TG and HDL. | The almond group had greater reductions in SBP compared to the control group. Almond group experienced significantly greater reductions in HDL. No significant difference between interventions found for TC, TG LDL, LDL:HDL ratio, DBP. | HIGH |
| Wien et al. (2010), US | RCT parallel | Almonds | 60 | 16 | n=65 enrolled (I: n = 32, C: n = 33). n = 54 completed the study (I: n = 25, C: n = 29) (intention-to-treat used). | 53 (44 - 62) | >25 | Adults with pre-diabetes (fasting BGL 100-125mg/dL), BMI 20-35kg/m2, willing to discontinue vitamin E supplements usage. Excluded if: self- reported allergy to almonds, hx of irritable bowel dx or diverticulitis, use of corticosteroids or immunosuppressant meds, presence of liver /renal disease and TGs <400mg/dL, TC<300mg/dL. | Intervention group with an American Diabetes Association diet (ADA) and ~60g daily of almonds (raw or dry-roasted) (equivalent to 20% of the total caloric intake). Control group with an ADA diet without nuts. | Personalised dietary advice with American diabetes association diet. | Dietary intake recorded by a 3-day food and activity diaries were completed at baseline and weeks 4, 8, 12 and 16. Fasting glucose, insulin, TC, LDP-C, HDL, TG, and TC:HDL measured at weeks 0, 8, and 16. BP and nutrient intake were measured at weeks 0, 4, 8, 12, and 16. Lipids measured after 12 – 14 hour fast and determined using standard enzymatic methods. | No significant change in TC, HDL, TG or BP between groups. Clinically significant in LDL observed in almond group compared to control group. | HIGH |
| Wien et al. (2014), US | RCT parallel | Peanuts | 46 (20% energy) | 24 | N=60 were recruited (n=3 WD due to unrelated health problems). | I: 59 ± 13, C: 64 ± 12  (34 - 84) | 17.2 - 48.7 | Adults with a medical diagnosis of type 2 diabetes for at least 6 months and HbA1c less than 9.0% Participants were excluded if they were <20 years of age, smoked, had nut allergies, liver disease, renal disease, severe dyslipidaemia (TG>4.52mmol/L or TC>7.77mmol/L) or a history of irritable bowel disease or diverticulitis that could be exacerbated by daily peanut intake. | Intervention group consumed an American Diabetes Association meal plan containing 20% of energy from peanuts. Control group: participants consumed an American Diabetes Association meal plan and did not consumed peanuts. | Individualized American Diabetes Association meal plan. | Compliance NR. Nutrient intake from 24-hour recalls were measured every 4 weeks. Venous blood samples were collected after a 12 hour overnight to determine TC, HDL, TG and LDL. Spectrophotometry was used to determine TC, HDL, TG and LDL. | No differences between the two groups in blood lipid measurements. Change from baseline data are presented for TC, LDL, HDL, TG, LDL:HDL ratio. | HIGH |
| Williams et al. (2019), US | RCT cross-over | Almonds | 20% energy | 3 | n=32 overweight or obese men and women.  n=8 loss to follow up or WD. n=24 completed the study. | ≥ 20 (M: 41 ± 15, F: 52 ± 9) | ≤ 38 (M: 31.7 ± 4.2, F: 31.0 ± 3.3) | Without a history of chronic disease in the last 5 years, not pregnant or breast feeding, no current use of hormones or drugs known to affect lipid metabolism or BP, no current use of nicotine products or recreational drugs, willingness to abstain from alcohol or dietary supplements during the study, SBP<160mmHg and DBP<95mmHg; TC and LDL <95th percentile for sex and age, fasting TG>0.56 mmol/L and <5.65 mmol/L, FBS<7.0 mmol/L, TSH within normal range, and weight stable for >3 months. | Intervention group consumed a higher carbohydrate diet with almonds. Control groups either consumed a higher carbohydrate reference diet or a lower carbohydrate reference diet. | Higher carbohydrate diet or a lower carbohydrate diet. | A compliance score was assigned to each study participant by the staff nutritionist, based on menu checklists, itemized grocery receipts and information gathered from weekly interactions. Dietary intake by providing participants ~ 65% daily energy in the form of two frozen entrees (lunch and dinner) and snacks. In addition, participants received dietary instructions, standardized menus, checklists, and shopping lists for home preparation of breakfast and sides for the remaining food items on the menu. Itemized grocery receipts were collected regularly from participants to verify their purchase of perishable foods on their shopping list. Participants were instructed to eat all food items provided/prescribed, and to report any deviations from the protocol. Plasma TG, TC and HDL were measured by enzymatic endpoint analysis on a clinical chemistry analyser. LDL was calculated from the Friedewald equation. Apo B and apo A1 were determined by immunoturbidimetric assay using the ITA reagent kit. | No significant differences in LDL between the carbohydrate high + almonds diet and the carbohydrate high diet. No significant differences among the diets for SBP, DBP, TC, LDL, HDL, TG, apo A1 and apo B. | HIGH |
| Wu et al. (2010), China | RCT parallel | Walnuts | 30 | 12 | n = 283 randomised, n=277 completed (intention-to-treat used). I: n=95, C: n=94. | Mean: 60 ± 1 | 24.9 ± 0.6 | Adults with the MetS. With at least three of 1)WC≥90 cm M /≥80 cm F. 2)TG≥1.7 mmol/L. 3)HDL<1.03 mmol/L M, <1.30 mmol/L F. 4)BP≥130/85 mmHg 5) fasting glucose ≥5.6 mmol/L 6) LD ≥3.4 mmol/L. Without the history of allergy or high intake (>120 gram/week) flaxseed, nuts, sesame seed, renal, heart, liver, pituitary thyroid or mental disease, alimentary tract ulceration of disease affecting absorption. History cancer, cardiovascular disease, mental disorder being pregnancy, lactating, use of antidepressants, oestrogen or steroid therapy. | Intervention group had lifestyle counselling with 30 g/d added walnut. Control group had lifestyle counselling with 30 g/d added flaxseed. | MetS was defined by the updated NCEP ATP III criteria for Asian Americans. | FFQ, 3-day food record and education and physical activity questionnaire at baseline and repeated at 12 weeks, no further information of method of dietary intake data analysis provided. Participants requested to return unused bread to measure compliance. BP measured seated after 5 minute rest. Fasting blood samples - LDL, HDL, TC, TG, apo A1, and apo B. | No significant effect on: SBP, DBP, TC, LDL, HDL, and TGs. | HIGH |
| Wu et al. (2014), Germany | RCT cross-over | Walnuts | 43 | 8 | n = 57 randomised. n = 40 included in analysis. n=46 completed the study. n=6 excluded from analysis due to protocol violations.. | 60 ± 1 | 24.9 ± 0.6 | Healthy Caucasian adults aged over 50 years, without obesity, diabetes or dyslipidaemia. | Intervention group consumed walnut enriched Western diet with addition of 43 g shelled walnuts/day (to replace 30 g of saturated fat in diet). Control group consumed isocaloric Western diet. | Mixed meal test of Cal shake powder, cream, milk and corn oil. | 4 day dietary report (4 reports per diet phase). Fasting blood samples and postprandial blood samples collected for lipid measures. Method of measuring BP measures not stated. Fasting concentration of adipokines, VCAM-1, ICAM-1 and endothelin-1 were determined using commercially available ELISA kits. Cholesterol and TG concentrations were measured on an autoanalyser (Alcyon 300) by using enzyme reagent kits. | No significant effect for walnut consumption compared to control was found for: fasting TC, LDL, HDL, and TG. No significant effects on measures of endothelial function. Participants maintained baseline BP during the study. | HIGH |
| Zambon et al. (2000), US | RCT cross-over | Walnuts | 41-56 | 6 | n = 55 randomised. n = 49 completed study. | Mean: 56 ± 11 | Mean: 27 ± 3.1 | Adults, with polygenic hypocholesterolemia, with no evidence of alcohol, tobacco, or drug abuse, absence of diabetes mellitus and liver, kidney, thyroid, or other endocrine diseases, as assessed by medical history, a complete physical examination, and laboratory tests, no intake in the previous 8 weeks of medications known to affect lipid metabolism, including hypolipidemic agents and estrogen compounds in women, infrequent consumption of nuts and no known history of allergy to them; no use of multivitamin or vitamin E supplements, with no evidence of familial hypercholesterolemia. | Intervention group consumed walnut diet similar to the control diet but walnuts partially replaced olive oil and other fatty foods. Control diet was Mediterranean diet of natural foods stuffs, red meat and eggs limited and vegetable products and fish were emphasized, olive oil was indicated for culinary use and no nuts were allowed. | The experimental diets were individually prescribed and were based on estimated energy requirements. | Compliance during the walnut period was assessed by a count of the empty walnut packages returned to clinic visits and fatty acid content of LDL. Twice during pretrial week and on weeks 5 and 6 of each dietary period patients visited the clinic for dietitian interview, anthropometric measurements and to have fasting blood samples taken. Adherence to the study diets was monitored by unannounced weekly 24hr diet recalls per patient. Dietary non-compliance was defined as at least 20% deviation from the dietary instructions regarding walnut or nutrient intake. Serum lipid and lipoprotein cholesterol and TG concentrations were determined using enzymatic analysis. All tests adhered to standard laboratory analysis for the outcome measures. | No significant difference found for HDL and TG. The 2 diets did not differ in levels of VLDL or apo A1. Apo B decreased after the 2 diets in parallel with LDL levels. Differences in lipid profile did not change when adjusted for baseline values or sex. | HIGH |
| Zaveri et al. (2009), UK | RCT parallel | Almonds | 56 | 12 | n = 45, n = 36 completed study (control: n = 12, cereal bar: n = 13, almonds: n = 11) | 25 - 50 | 25- 35 (C: 29.1 ±1.7; I: 30.4 ± 2.8) | With healthy overweight males without any major illness, or from weight loss diet or program. | Intervention group instructed to have 2x 28 g packets almonds/day at any time of the day they wish. Cereal group instructed to have 2x 30 g cereal bars/day at any time of the day they wish. Control group advised to maintain usual eating pattern. | The healthy eating advice did not differ from current public health messages (The Scottish Office, 1996) | Compliance was assessed in all groups by a 4 day unweighted food diary at baseline, week 6 and week12. Same four days of the week reported with each record. Portion sizes estimated using Standard Food Portion Sizes, diets analysed using CompEat Pro version 5.8 with additional nutritional information obtained from the cereal bar suppliers. Fasting blood samples collected for TC, TGs, LDL, and HDL. | There were no significant differences within groups or between groups in TC, TG, LDL, and HDL. After removing the under reporters, there was a significant decrease in mean TG in the C group from baseline to 12 weeks. | LOW |
| Zhao et al. (2004)  West et al. (2010), US | RCT cross-over | Walnuts | I1:37 I2:15 | 6 | Zhao et al. (2004): n=23 overweight/obese class I adults with moderate hypercholesterolemia, (M:20, postmenopausal F:3).   West et al. (2010): n=20 included in analysis. | Zhao et al. (2004): 49.8 ± 1.6  West et al. (2010): 49.3 ± 1.7 | Zhao et al. (2004): 28.1 ± 0.7  West et al. (2010): 28.8 ± 0.8 | Without current use of nutritional supplements or medications for hypercholesterolemia, hypertension, or inflammatory disease; and no history of cardiovascular disease, hypertension, diabetes, or other systemic disease. | Intervention group 1 consumed 100 g from walnuts and 100 g walnut oil. Intervention group 2 consumed walnuts, 100 g walnut oil and 100 g flaxseed oil). Control group consumed an average American diet. | All 3 diets had comparable amounts of fat [35% energy (energy)], carbohydrate (50% energy), protein (15% energy), and cholesterol (300 mg/d). | Compliance by changes in serum fatty acid profiles reflected the fatty acid composition of the 3 test diets. Dietary intake: NR. Assays for serum TC, HDL and TG were conducted at the Mary Imogene Bassett Research Institute, using an enzymatic method. LDL levels were calculated by Friedewald’s equation. Apo A1 and apo B were determined by rate immunonephelometry on a Beckman Array. Serum ICAM-1, VCAM-1, and E-selectin were measured using quantitative sandwich enzyme immunoassay kits. Measurements of brachial artery diameter and blood flow volume/velocity using an Acuson 128XP ultrasound imaging system with a 10-MHz linear-array transducer. Endothelin-1 was measured via enzyme immunoassay using commercially available kits. | Serum TC, LDL, TG, and apo B levels were lower, respectively, when subjects consumed the walnut only and walnut + flaxseed diet when compared with the control. Although the walnut +flaxseed diet significantly decreased HDL and apo A1 compared with the control, there were no differences in HDL and apo A1 levels when subjects consumed the walnut only and walnut + flaxseed diets, and the 2 diets reduced TC:HDL ratios similarly. The 2 intervention diets significantly decreased serum ICAM-1 and E-selectin compared with control diet. The walnut + flaxseed diet also significnalty reduced VCAM-1 levels compared with the control; however, VCAM-1 levels did not differ after subjects consumed the walnut only diet and the walnut + flaxseed diet. There was significant differences across the diets in DBP. There were no changes in endothelin-1. Participants with larger decreases in systemic inflammation had larger improvements in endothelial function. There was no relationship between diet-related change in lipids, vasoactive hormones, or cell adhesion molecules and change in FMD. | HIGH |
| Zibaeenezhad et al. (2003), Iran | RCT parallel | Walnuts | 3 | 6 (+3 days) | n = 60 (I: n=29, C: n = 31). | I: 49.2 ± 9.9, C: 43.8 ± 11.3 | NR | Men and women with either elevated plasma TGs or TC. Mean weight: walnut group: 71.4kg, control group: 70.8kg. | Intervention group were administered 6 capsules each filled with 500mg of the extracted Persian walnut oil per day for 45 days. Controls received placebo for 45 days. | Subjects in both groups were advised to not take any other forms of walnut or change their diet style and habit. | No assessment of background diet at any time in either group. No measures of subject compliance to the oil or placebo capsules. TG concentrations, TC, HDL and LDL were checked for each subject before; on days 15, 30, and 45 after the beginning and 15 days after termination of the study. Not clear if subjects fasted prior to lipid measurement or for how long they fasted. TGs were measured using the GPO-PAP method (standard method) and cholesterol was checked by GPA-PAP technique. HDL was measured by dextran sulphate magnesium sulfate and LDL was assessed using the Friedewald calculation. | In the walnut oil group, plasma TG decreased 19% after 15days compared to baseline. At 45 days TG levels were 19% lower than baseline 19% lower than baseline (statistical significance not reported). After 60 days TG concentrations were 23% lower than baseline values.  In the control group, TGs decreased by 20.5% of baseline after 30days, 1.4% after 45 days, and 2.8% after 60 days, but this change was not statistically significant. The changes in TC, LDL and HDL were not statistically significant in either group: TC-C, LDL, and HDL. | LOW |
| Zibaeenezhad et al. (2005), Iran | Randomised case control study | Walnuts | 20 | 8 | n = 52 recruited, n = 43 completed study (I: n=20, C: n = 23). | NR | NR | With plasma TG >350mg/dL or TC >250mg/dL; no clinical evidence of renal, liver or endocrine disease. | Intervention group (n=25) consumed Persian walnuts, 20 g/d. Controls were served with (n=27) usual diet without walnuts. | NR. | No assessment of background diet at any time in either group. No measures of subject compliance to walnuts.  TG, LDL and HDL were measured at baseline, 4 weeks and 8 weeks (does not state if fasted). | The mean plasma TG level declined from the baseline value in the intervention group while the HDL increased significantly. There was no statistically significant change in the lipid profile of the control group during the study period. | LOW |
| Zibaeenezhad et al. (2017), Iran | RCT parallel | Walnuts | 15 cc/day | 12 (+6 days) | n=90 clinical diagnosis of type 2 diabetes for at least 2 years, 45 per group.. | 35 - 75  (I:55.5 ± 10.7, C: 54 ± 11.4) | NR | Without a history of chronic or metabolic diseases, pregnancy, regular use of medication (fibrate and HMG-COA reductase inhibitor) or supplements known to affect the blood lipids, and currently receiving insulin therapy. | Intervention group consumed 4 walnut oil capsules containing 1.25cc Persian walnut oil, 3 times/day with food (15 cc daily). Control group consumed four placebo capsules containing 1.25 cc distilled water, 3 times/d with food (15 cc daily). | NR. | Compliance and dietary intake NR. TG was measured by glycerol phosphate oxidase- phenol and aminophenazone method. TC was also checked by cholesterol oxidase/phenol and aminophenazone technique. HDL was measured by dextran magnesium sulfate. LDL was derived according to the following formula: LDL = TC − (HDL+TG/5). BP was measured after a 5-min resting period using a standard mercury sphygmomanometer. | Compared to the control group, consumption of walnut oil resulted in a significant decrease in TC, TG, LDL and TC to HDL ratio. There was a trend toward increasing HDL level with consumption of walnut oil. | HIGH |
| Zibaeenezhad et al. (2019), Iran | RCT parallel | Almonds | 10 ml (two times per day) | 4 (+2days) | n=97 clinically diagnosed hyperlipidemic males and females who did not smoke (n=49 to intervention and n=48 to control group). n=9 loss to follow up, WD or excluded from analysis. n=82 completed the study (I: n=42 and C: n=40). | 20 - 75 (I: 49.4 ± 12.0, C: 50.19 ± 9.87) | I: 26.47 ± 2.89,  C: 28.5 ± 4.18 | With plasma TG level between 200–400 mg/dL, TC level of >200 mg/dL, LDL <160 or HDL <50 mg/dL in females and less than 40 mg/dL in males. Without history of renal, liver, kidney, heart and thyroid diseases, diabetes mellitus, allergy to nuts, asthma or atopic dermatitis. Were not breastfeeding or pregnacy, taking oral contraceptive pills, consuming alcohol or hospitalized during the study. | Intervention group received 10 ml of Persian almond oil, two times per day, for 30 days. The control group did not receive any intervention during the study period. | Habitual diet. | Compliance and dietary intake NR. Though consumption of <70% of the drugs during the trial was considered as drug intolerance, and the patient was excluded from the trial. The serum TG was measured by GPO-PAP method providing a normal upper limit of 200 mg/dL (2.3 mmol/L). The TC was also checked by GPA-PAP technique, which provided an upper limit normal value or 220 mg/dL (5.6 mmol/L). The HDL was measured by dextran magnesium sulfate. The LDL was derived according to the following formula: LDL = TC–(HDL + TG/5). | In TC, the intervention group had a significant decrease but a non-significant difference in the control group. In LDL, the intervention group a significant decrease and a significant increase in the control group. TGs decreased non-significantly in the intervention group and a non-significant trend toward increase in the control group. HDL non-significant increase for the intervention group and significantly decreased in the control group. | HIGH |

# **Supplementary Table 4:** Results of sub-group analyses for LDL cholesterol

| **Sub-group analysis category** | **Sub-group** | **Number of analyses** | **Number of participants** | **Effect estimate** | **Test for sub-group differences** |
| --- | --- | --- | --- | --- | --- |
| Study design | Cross-over | 54 | 3837 | -0.14 [-0.19, -0.09] | Chi² = 4.39, df = 1 (P = 0.04), I² = 77.2% |
|  | Parallel | 72 | 5650 | -0.07 [-0.11, -0.03] |  |
| Nut group | Almond | 32 | 2439 | -0.15 [-0.22, -0.08] | Chi² = 25.86, df = 10 (P = 0.004), I² = 61.3% |
|  | Brazil nut | 4 | 307 | -0.30 [-0.70, 0.11] |  |
|  | Cashew nut | 3 | 432 | 0.02 [-0.12, 0.16] |  |
|  | Hazelnut | 6 | 374 | -0.01 [-0.15, 0.12] |  |
|  | Macadamia | 6 | 410 | -0.11 [-0.27, 0.04] |  |
|  | Mixed nuts | 10 | 791 | 0.04 [-0.06, 0.14] |  |
|  | Peanut | 10 | 1021 | 0.08 [-0.04, 0.20] |  |
|  | Pecan | 6 | 295 | -0.23 [-0.46, 0.00] |  |
|  | Pistachio | 12 | 736 | -0.15 [-0.30, 0.00] |  |
|  | Walnut | 35 | 2582 | -0.12 [-0.18, -0.06] |  |
|  | Two different nuts | 2 | 100 | -0.31 [-0.82, 0.21] |  |
| Nut dose | ≤30 | 28 | 2524 | -0.02 [-0.14, 0.10] | Chi² = 5.43, df = 3 (P = 0.14), I² = 44.8% |
|  | 31-60 | 54 | 4343 | -0.11 [-0.15, -0.07] |  |
|  | >60 | 30 | 1472 | -0.19 [-0.29, -0.09] |  |
|  | Nut oil, butter and flour either alone or in combination with nuts | 11 | 812 | -0.22 [-0.44, -0.00] |  |
| Study duration | <12 weeks | 75 | 4213 | -0.13 [-0.18, -0.08] | Chi² = 1.95, df = 1 (P = 0.16), I² = 48.7% |
|  | ≥12 weeks | 51 | 5274 | -0.08 [-0.12, -0.03] |  |
| Health status | CAD | 4 | 360 | -0.11 [-0.32, 0.10] | Chi² = 22.11, df = 8 (P = 0.005), I² = 63.8% |
|  | CVD and/ or risk of CVD | 3 | 203 | -0.21 [-0.35, -0.06] |  |
|  | Healthy | 29 | 2219 | -0.08 [-0.12, -0.04] |  |
|  | Hypercholesterolemia/ hyperlipidemia | 26 | 1565 | -0.26 [-0.35, -0.17] |  |
|  | MetS and/or at risk of MetS | 9 | 856 | -0.06 [-0.17, 0.06] |  |
|  | Overweight/obesity | 29 | 2148 | -0.02 [-0.09, 0.05] |  |
|  | Prediabetes | 6 | 572 | -0.11 [-0.24, 0.02] |  |
|  | T2DM | 15 | 974 | -0.06 [-0.16, 0.04] |  |
|  | Multiple | 5 | 590 | -0.13 [-0.49, 0.23] |  |

Abbreviations: CAD, coronary artery disease; CVD, cardiovascular disease; MetS, metabolic syndrome; T2DM, type 2 diabetes mellitus.

# **Supplementary Table 5:** Results of sensitivity analyses for studies exploring the effects of whole nuts

| **Outcome** | **Nut dose sub-group** | **Number of analyses** | **Number of participants** | **Effect estimate** | **Test for sub-group differences** | **Test for overall effect** |
| --- | --- | --- | --- | --- | --- | --- |
| LDL | ≤30 | 28 | 2524 | -0.02 [-0.14, 0.10] | Chi² = 4.50, df = 2 (P = 0.11), I² = 55.5% | Chi² = 557.09, df = 111 (P < 0.00001), I² = 80.0% |
|  | 31-60 | 54 | 4343 | -0.11 [-0.15, -0.07] |  |  |
|  | >60 | 30 | 1472 | -0.19 [-0.29, -0.09] |  |  |
|  | Total | 112 | 8339 | -0.10 [-0.14, -0.07] |  |  |
| HDL | ≤30 | 29 | 2568 | 0.01 [-0.01, 0.04] | Chi² = 0.90, df = 2 (P = 0.64), I² = 0% | Chi² = 641.74, df = 110 (P = 0.75), I² = 83% |
|  | 31-60 | 55 | 5143 | -0.00 [-0.03, 0.02] |  |  |
|  | >60 | 27 | 1286 | 0.01 [-0.02, 0.04] |  |  |
|  | Total | 111 | 8997 | 0.00 [-0.01, 0.02] |  |  |
| TC | ≤30 | 28 | 2524 | -0.01 [-0.11, 0.09] | Chi² = 8.24, df = 2 (P = 0.02), I² = 75.7% | Chi² = 916.75, df = 111 (P < 0.00001), I² = 88.0% |
|  | 31-60 | 54 | 4471 | -0.14 [-0.18, -0.09] |  |  |
|  | >60 | 30 | 1418 | -0.23 [-0.36, -0.10] |  |  |
|  | Total | 112 | 8413 | -0.12 [-0.17, -0.08] |  |  |
| TG | ≤30 | 26 | 2369 | -0.05 [-0.12, 0.01] | Chi² = 0.62, df = 2 (P = 0.73), I² = 0% | Chi² = 269.95, df = 105 (P < 0.0001), I² = 61% |
|  | 31-60 | 53 | 4989 | -0.05 [-0.08, -0.02] |  |  |
|  | >60 | 27 | 1297 | -0.07 [-0.13, -0.01] |  |  |
|  | Total | 106 | 8655 | -0.05 [-0.07, -0.03] |  |  |
| TC:HDL | ≤30 | 10 | 1063 | -0.12 [-0.25, 0.01] | Chi² = 1.99 df = 2 (P = 0.37), I² = 0% | Chi² = 150.68 df = 46 (P = 0.0003), I² = 69% |
|  | 31-60 | 26 | 1934 | -0.11 [-0.20, -0.02] |  |  |
|  | >60 | 11 | 638 | -0.32 [-0.60, -0.04] |  |  |
|  | Total | 47 | 3647 | -0.16 [-0.25, -0.07] |  |  |
| LDL:HDL | ≤30 | 5 | 377 | -0.24 [-0.40, -0.09] | Chi² = 2.85, df = 2 (P = 0.24), I² = 29.9% | Chi² = 142.00, df = 36 (P < 0.00001), I² = 75.0% |
|  | 31-60 | 19 | 1355 | -0.11 [-0.18, -0.03] |  |  |
|  | >60 | 13 | 689 | -0.18 [-0.31, -0.06] |  |  |
|  | Total | 37 | 2421 | -0.15 [-0.21, -0.09] |  |  |
| ApoB | ≤30 | 8 | 650 | -0.11 [-3.87, 3.65] | Chi² = 4.52, df = 2 (P = 0.10), I² = 55.7% | Chi² = 55.99, df = 33 (P < 0.0001), I² = 41.0% |
|  | 31-60 | 16 | 1525 | -4.32 [-5.61, -3.03] |  |  |
|  | >60 | 10 | 461 | -4.70 [-7.97, -1.43] |  |  |
|  | Total | 34 | 2636 | -3.10 [-4.58, -1.62] |  |  |
| ApoA-I | ≤30 | 6 | 464 | 0.59 [-5.04, 6.22] | Chi² = 1.00, df = 2 (P = 0.61), I² = 0% | Chi² = 24.28, df = 27 (P = 0.45), I² = 0% |
|  | 31-60 | 13 | 941 | -1.43 [-3.61, 0.75] |  |  |
|  | >60 | 9 | 409 | 0.70 [-3.73, 5.14] |  |  |
|  | Total | 28 | 1814 | -0.64 [-2.30, 1.03] |  |  |
| SBP | ≤30 | 19 | 2035 | 0.82 [-0.68, 2.31] | Chi² = 2.36, df = 2 (P = 0.31), I² = 15.2% | Chi² = 121.68, df = 63 (P = 0.92), I² = 48.0% |
|  | 31-60 | 31 | 3129 | -0.76 [-2.11, 0.59] |  |  |
|  | >60 | 14 | 733 | 0.04 [-1.47, 1.56] |  |  |
|  | Total | 64 | 5897 | -0.05 [-0.92, 0.83] |  |  |
| DBP | ≤30 | 17 | 1890 | 0.21 [-0.62, 1.04] | Chi² = 1.68, df = 2 (P = 0.43), I² = 0% | Chi² = 78.70, df = 60 (P = 0.53), I² = 24% |
|  | 31-60 | 30 | 3003 | -0.24 [-0.91, 0.44] |  |  |
|  | >60 | 14 | 733 | -0.61 [-1.54, 0.32] |  |  |
|  | Total | 61 | 5626 | -0.15 [-0.61, 0.32] |  |  |

Abbreviations: ApoA-I, apolipprotein A-1; ApoB, apolipoprotein B; DBP, diastolic blood pressure; HDL, high density lipoprotein; LDL, low density lipoprotein; SBP, systolic blood pressure; TC, total cholesterol; TG, triglycerides.

# **Supplementary Table 6:** Results of sub-group analyses for total cholesterol

| **Sub-group analysis category** | **Sub-group** | **Number of analyses** | **Number of participants** | **Effect estimate** | **Test for sub-group differences** |
| --- | --- | --- | --- | --- | --- |
| Study design | Cross-over | 55 | 3881 | -0.14 [-0.19, -0.10] | Chi² = 0.47, df = 1 (P = 0.49), I² = 0% |
|  | Parallel | 71 | 5542 | -0.12 [-0.19, -0.05] |  |
| Nut group | Almond | 33 | 2469 | -0.19 [-0.27, -0.12] | Chi² = 28.72, df = 10 (P = 0.001), I² = 65.2% |
|  | Brazil nut | 4 | 307 | -0.43 [-0.80, -0.06] |  |
|  | Cashew nut | 3 | 432 | 0.03 [-0.12, 0.19] |  |
|  | Hazelnut | 5 | 267 | -0.02 [-0.30, 0.26] |  |
|  | Macadamia | 6 | 410 | -0.15 [-0.35, 0.06] |  |
|  | Mixed nuts | 11 | 888 | 0.03 [-0.12, 0.17] |  |
|  | Peanut | 10 | 1021 | 0.04 [-0.05, 0.14] |  |
|  | Pecan | 6 | 295 | -0.27 [-0.56, 0.03] |  |
|  | Pistachio | 11 | 652 | -0.21 [-0.35, -0.06] |  |
|  | Walnut | 35 | 2582 | -0.14 [-0.21, -0.07] |  |
|  | Two different nuts | 2 | 100 | -0.35 [-0.98, 0.29] |  |
| Nut dose | ≤30 | 28 | 2524 | -0.01 [-0.11, 0.09] | Chi² = 10.55, df = 3 (P = 0.01), I² = 71.6% |
|  | 31-60 | 54 | 4471 | -0.14 [-0.18, -0.09] |  |
|  | >60 | 30 | 1418 | -0.23 [-0.36, -0.10] |  |
|  | Nut oil, butter and flour either alone or in combination with nuts | 11 | 812 | -0.31 [-0.53, -0.08] |  |
| Study duration | <12 weeks | 74 | 3991 | -0.15 [-0.21, -0.08] | Chi² = 0.51, df = 1 (P = 0.48), I² = 0% |
|  | ≥12 weeks | 51 | 5264 | -0.11 [-0.18, -0.05] |  |
| Health status | CAD | 4 | 360 | -0.12 [-0.34, 0.10] | Chi² = 11.24, df = 8 (P = 0.19), I² = 28.8% |
|  | CVD and/or risk of CVD | 3 | 203 | -0.20 [-0.37, -0.04] |  |
|  | Healthy | 30 | 2249 | -0.15 [-0.23, -0.06] |  |
|  | Hypercholesterolemia/ hyperlipidemia | 25 | 1481 | -0.26 [-0.36, -0.16] |  |
|  | MetS and/or at risk of MetS | 9 | 856 | -0.10 [-0.23, 0.04] |  |
|  | Overweight/obesity | 29 | 2138 | -0.06 [-0.17, 0.05] |  |
|  | Prediabetes | 6 | 572 | -0.12 [-0.27, 0.03] |  |
|  | T2DM | 15 | 974 | -0.05 [-0.18, 0.08] |  |
|  | Multiple | 5 | 590 | -0.19 [-0.60, 0.22] |  |

Abbreviations: CAD, coronary artery disease; CVD, cardiovascular disease; MetS, metabolic syndrome; T2DM, type 2 diabetes mellitus.

# **Supplementary Table 7:** Results of sub-group analyses for triglycerides

| **Sub-group analysis category** | **Sub-group** | **Number of analyses** | **Number of participants** | **Effect estimate** | **Test for sub-group differences** |
| --- | --- | --- | --- | --- | --- |
| Study design | Cross-over | 54 | 3813 | -0.08 [-0.10, -0.07] | Chi² = 2.62, df = 1 (P = 0.11), I² = 61.8% |
|  | Parallel | 68 | 5931 | -0.05 [-0.08, -0.02] |  |
| Nut group | Almond | 32 | 2439 | -0.02 [-0.05, 0.02] | Chi² = 23.85, df = 10 (P = 0.008), I² = 58.1% |
|  | Brazil nut | 4 | 307 | 0.04 [-0.54, 0.63] |  |
|  | Cashew nut | 3 | 432 | -0.02 [-0.11, 0.07] |  |
|  | Hazelnut | 5 | 313 | 0.11 [-0.02, 0.25] |  |
|  | Macadamia | 5 | 342 | -0.10 [-0.21, 0.00] |  |
|  | Mixed nuts | 11 | 888 | -0.01 [-0.07, 0.06] |  |
|  | Peanut | 10 | 1021 | -0.09 [-0.16, -0.02] |  |
|  | Pecan | 6 | 295 | -0.11 [-0.24, 0.03] |  |
|  | Pistachio | 9 | 498 | -0.12 [-0.21, -0.03] |  |
|  | Walnut | 35 | 3109 | -0.09 [-0.12, -0.06] |  |
|  | Two different nuts | 2 | 100 | -0.20 [-0.47, 0.08] |  |
| Nut dose | ≤30 | 26 | 2369 | -0.05 [-0.12, 0.01] | Chi² = 1.96, df = 3 (P = 0.58), I² = 0% |
|  | 31-60 | 53 | 4989 | -0.05 [-0.08, -0.02] |  |
|  | >60 | 27 | 1297 | -0.07 [-0.13, -0.01] |  |
|  | Nut oil, butter and flour either alone or in combination with nuts | 10 | 744 | -0.16 [-0.34, 0.02] |  |
| Study duration | <12 weeks | 73 | 4061 | -0.05 [-0.08, -0.03] | Chi² = 0.04, df = 1 (P = 0.85), I² = 0% |
|  | ≥12 weeks | 49 | 5683 | -0.06 [-0.10, -0.02] |  |
| Health status | CAD | 4 | 360 | -0.12 [-0.27, 0.02] | Chi² = 2.28, df = 8 (P = 0.97), I² = 0% |
|  | CVD and/or risk of CVD | 3 | 203 | -0.03 [-0.17, 0.11] |  |
|  | Healthy | 29 | 2188 | -0.06 [-0.10, -0.02] |  |
|  | Hypercholesterolemia/ hyperlipidemia | 23 | 1383 | -0.05 [-0.11, 0.01] |  |
|  | MetS and/or at risk of MetS | 8 | 1327 | -0.01 [-0.15, 0.12] |  |
|  | Overweight/obesity | 29 | 2147 | -0.07 [-0.15, -0.00] |  |
|  | Prediabetes | 6 | 572 | -0.07 [-0.14, -0.01] |  |
|  | T2DM | 15 | 974 | -0.09 [-0.20, 0.02] |  |
|  | Multiple | 5 | 590 | -0.07 [-0.31, 0.16] |  |

Abbreviations: CAD, coronary artery disease; CVD, cardiovascular disease; MetS, metabolic syndrome; T2DM, type 2 diabetes mellitus.

# **Supplementary Table 8:** Results of sub-group analyses for total cholesterol to HDL cholesterol ratio

| **Sub-group analysis category** | **Sub-group** | **Number of analyses** | **Number of participants** | **Effect estimate** | **Test for sub-group differences** |
| --- | --- | --- | --- | --- | --- |
| Study design | Cross-over | 25 | 1535 | -0.17 [-0.24, -0.09] | Chi² = 0.04, df = 1 (P = 0.84), I² = 0% |
|  | Parallel | 26 | 2302 | -0.15 [-0.31, 0.02] |  |
| Nut group | Almond | 16 | 1372 | -0.11 [-0.23, 0.02] | Chi² = 7.16, df = 8 (P = 0.52), I² = 0% |
|  | Cashew nut | 3 | 396 | -0.18 [-0.41, 0.05] |  |
|  | Hazelnut | 1 | 107 | 0.15 [-0.29, 0.58] |  |
|  | Macadamia | 1 | 48 | -0.29 [-0.96, 0.38] |  |
|  | Mixed nuts | 4 | 411 | 0.02 [-0.19, 0.22] |  |
|  | Peanut | 5 | 329 | -0.19 [-0.65, 0.27] |  |
|  | Pecan | 2 | 187 | -0.42 [-0.79, -0.05] |  |
|  | Pistachio | 7 | 355 | -0.29 [-0.64, 0.05] |  |
|  | Walnut | 12 | 632 | -0.12 [-0.23, -0.01] |  |
| Nut dose | ≤30 | 10 | 1063 | -0.12 [-0.25, 0.01] | Chi² = 2.02 df = 3 (P = 0.57), I² = 0% |
|  | 31-60 | 26 | 1934 | -0.11 [-0.20, -0.02] |  |
|  | >60 | 11 | 638 | -0.32 [-0.60, -0.04] |  |
|  | Nut oil, butter and flour either alone or in combination with nuts | 3 | 142 | -0.09 [-0.52, 0.34] |  |
| Study duration | <12 weeks | 32 | 1937 | -0.19 [-0.30, -0.08] | Chi² = 1.27, df = 1 (P = 0.26), I² = 21.4% |
|  | ≥12 weeks | 19 | 1900 | -0.09 [-0.22, 0.04] |  |
| Health status | CAD | 3 | 315 | -0.23 [-0.43, -0.02] | Chi² = 7.20, df = 7 (P = 0.41), I² = 2.7% |
|  | CVD and/or risk of CVD | 2 | 153 | -0.29 [-1.00, 0.41] |  |
|  | Healthy | 7 | 413 | -0.12 [-0.57, 0.32] |  |
|  | Hypercholesterolemia/ hyperlipidemia | 12 | 632 | -0.27 [-0.41, -0.12] |  |
|  | Overweight/obesity | 14 | 1207 | -0.10 [-0.23, 0.03] |  |
|  | Prediabetes | 4 | 253 | -0.14 [-0.32, 0.03] |  |
|  | T2DM | 8 | 679 | -0.11 [-0.24, 0.01] |  |
|  | Multiple | 1 | 185 | 0.20 [-0.19, 0.59] |  |

Abbreviations: CAD, coronary artery disease; CVD, cardiovascular disease; MetS, metabolic syndrome; T2DM, type 2 diabetes mellitus.

# **Supplementary Table 9:** Results of sub-group analyses for LDL cholesterol to HDL cholesterol ratio

| **Sub-group analysis category** | **Sub-group** | **Number of analyses** | **Number of participants** | **Effect estimate** | **Test for sub-group differences** |
| --- | --- | --- | --- | --- | --- |
| Study design | Cross-over | 26 | 1679 | -0.14 [-0.21, -0.08] | Chi² = 0.27, df = 1 (P = 0.60), I² = 0% |
|  | Parallel | 13 | 860 | -0.10 [-0.25, 0.04] |  |
| Nut group | Almond | 11 | 818 | -0.18 [-0.22, -0.15] | Chi² = 90.28, df = 8 (P < 0.00001), I² = 91.1% |
|  | Cashew nut | 1 | 43 | -0.43 [-0.79, -0.07] |  |
|  | Macadamia | 1 | 48 | -0.18 [-0.67, 0.31] |  |
|  | Mixed nuts | 3 | 200 | -0.02 [-0.19, 0.14] |  |
|  | Peanut | 6 | 412 | -0.04 [-0.36, 0.29] |  |
|  | Pecan | 2 | 181 | -0.25 [-0.48, -0.03] |  |
|  | Pistachio | 5 | 253 | -0.25 [-0.27, -0.22] |  |
|  | Walnut | 9 | 548 | -0.07 [-0.10, -0.04] |  |
|  | Two different nuts | 1 | 36 | -0.35 [-0.82, 0.12] |  |
| Nut dose | ≤30 | 5 | 377 | -0.24 [-0.40, -0.09] | Chi² = 3.41, df = 3 (P = 0.33), I² = 11.9% |
|  | 31-60 | 19 | 1355 | -0.11 [-0.18, -0.03] |  |
|  | >60 | 13 | 689 | -0.18 [-0.31, -0.06] |  |
|  | Nut oil, butter and flour either alone or in combination with nuts | 2 | 118 | 0.13 [-0.59, 0.86] |  |
| Study duration | <12 weeks | 28 | 1583 | -0.16 [-0.23, -0.10] | Chi² = 8.49, df = 1 (P = 0.004), I² = 88.2% |
|  | ≥12 weeks | 11 | 956 | -0.06 [-0.09, -0.03] |  |
| Health status | CAD | 3 | 315 | -0.21 [-0.48, 0.07] | Chi² = 12.31, df = 5 (P = 0.03), I² = 59.4% |
|  | Healthy | 10 | 608 | -0.16 [-0.27, -0.04] |  |
|  | Hypercholesterolemia/ hyperlipidemia | 12 | 629 | -0.28 [-0.41, -0.16] |  |
|  | Overweight/obesity | 6 | 457 | 0.09 [-0.08, 0.26] |  |
|  | Prediabetes | 1 | 98 | -0.11 [-0.33, 0.11] |  |
|  | T2DM | 7 | 432 | -0.11 [-0.25, 0.02] |  |

Abbreviations: CAD, coronary artery disease; T2DM, type 2 diabetes mellitus.

# **Supplementary Table 10:** Results of sub-group analyses for apolipoprotein B

| **Sub-group analysis category** | **Sub-group** | **Number of analyses** | **Number of participants** | **Effect estimate** | **Test for sub-group differences** |
| --- | --- | --- | --- | --- | --- |
| Study design | Cross-over | 27 | 1970 | -3.81 [-5.44, -2.18] | Chi² = 6.12, df = 1 (P = 0.01), I² = 83.7% |
|  | Parallel | 12 | 1099 | -0.50 [-2.56, 1.55 |  |
| Nut group | Almond | 10 | 675 | -4.95 [-5.73, -4.17] | Chi² = 32.31, df = 8 (P < 0.0001), I² = 75.2% |
|  | Brazil nut | 1 | 77 | -0.20 [-11.32, 10.92] |  |
|  | Cashew nut | 1 | 79 | 1.50 [-2.38, 5.38] |  |
|  | Hazelnut | 3 | 205 | -1.16 [-5.67, 3.34] |  |
|  | Mixed nuts | 3 | 245 | -0.53 [-6.33, 5.27] |  |
|  | Peanut | 4 | 439 | 2.48 [-0.90, 5.85] |  |
|  | Pecan | 2 | 98 | -8.96 [-17.30, -0.61] |  |
|  | Pistachio | 4 | 153 | -4.99 [-9.48, -0.49] |  |
|  | Walnut | 11 | 1098 | -4.76 [-6.58, -2.93] |  |
| Nut dose | ≤30 | 8 | 650 | -0.11 [-3.87, 3.65] | Chi² = 4.71, df = 3 (P = 0.19), I² = 36.4% |
|  | 31-60 | 16 | 1525 | -4.32 [-5.61, -3.03] |  |
|  | >60 | 10 | 461 | -4.70 [-7.97, -1.43] |  |
|  | Nut oil, butter and flour either alone or in combination with nuts | 4 | 385 | -2.61 [-8.52, 3.29] |  |
| Study duration | <12 weeks | 26 | 1503 | -3.55 [-5.67, -1.44] | Chi² = 0.00, df = 1 (P = 0.98), I² = 0% |
|  | ≥12 weeks | 13 | 1566 | -3.52 [-5.05, -1.98] |  |
| Health status | CAD | 1 | 45 | 4.80 [-6.55, 16.15] | Chi² = 40.24, df = 8 (P < 0.00001), I² = 80.1% |
|  | CVD and/or risk of CVD | 1 | 52 | -7.83 [-19.87, 4.21] |  |
|  | Healthy | 9 | 883 | -4.34 [-6.82, -1.86] |  |
|  | Hypercholesterolemia/ hyperlipidemia | 13 | 805 | -5.05 [-5.82, -4.27] |  |
|  | MetS and/or at risk of MetS | 2 | 273 | 0.71 [-3.26, 4.69] |  |
|  | Overweight/obesity | 7 | 569 | -1.29 [-3.88, 1.30] |  |
|  | Prediabetes | 1 | 100 | 5.60 [1.17, 10.03] |  |
|  | T2DM | 3 | 209 | -5.71 [-17.05, 5.63] |  |
|  | Multiple | 2 | 133 | 0.36 [-4.55, 5.27] |  |

Abbreviations: CAD, coronary artery disease; CVD, cardiovascular disease; MetS, metabolic syndrome; T2DM, type 2 diabetes mellitus.

# **Supplementary Table 11:** Results of sub-group analyses for apolipoprotein A-I

| **Sub-group analysis category** | **Sub-group** | **Number of analyses** | **Number of participants** | **Effect estimate** | **Test for sub-group differences** |
| --- | --- | --- | --- | --- | --- |
| Study design | Cross-over | 24 | 1332 | -0.61 [-2.37, 1.14] | Chi² = 0.44, df = 1 (P = 0.51), I² = 0% |
|  | Parallel | 9 | 915 | -2.26 [-6.78, 2.26] |  |
| Nut group | Almond | 10 | 663 | -0.30 [-3.26, 2.67] | Chi² = 2.44, df = 8 (P = 0.96), I² = 0% |
|  | Brazil nut | 1 | 77 | -2.30 [-14.50, 9.90] |  |
|  | Cashew nut | 1 | 79 | -0.60 [-5.60, 4.40] |  |
|  | Hazelnut | 3 | 205 | 1.04 [-6.39, 8.48] |  |
|  | Mixed nuts | 2 | 159 | -1.10 [-8.54, 6.34] |  |
|  | Peanut | 2 | 287 | -4.53 [-11.55, 2.49] |  |
|  | Pecan | 1 | 46 | 3.00 [-8.85, 14.85] |  |
|  | Pistachio | 5 | 205 | -2.27 [-9.13, 4.58] |  |
|  | Walnut | 8 | 526 | -1.84 [-4.62, 0.95] |  |
| Nut dose | ≤30 | 6 | 464 | 0.59 [-5.04, 6.22] | Chi² = 2.67, df = 3 (P = 0.44), I² = 0% |
|  | 31-60 | 13 | 941 | -1.43 [-3.61, 0.75] |  |
|  | >60 | 9 | 409 | 0.70 [-3.73, 5.14] |  |
|  | Nut oil, butter and flour either alone or in combination with nuts | 4 | 385 | -4.34 [-9.29, 0.62] |  |
| Study duration | <12 weeks | 25 | 1442 | -0.57 [-2.28, 1.14] | Chi² = 1.16, df = 1 (P = 0.28), I² = 14.0% |
|  | ≥12 weeks | 8 | 805 | -2.78 [-6.41, 0.85] |  |
| Health status | CAD | 1 | 45 | -2.60 [-17.29, 12.09] | Chi² = 0.90, df = 6 (P = 0.99), I² = 0% |
|  | Healthy | 7 | 395 | -1.24 [-4.51, 2.02] |  |
|  | Hypercholesterolemia/ hyperlipidemia | 13 | 805 | -0.88 [-3.76, 2.01] |  |
|  | MetS and/or at risk of MetS | 1 | 189 | 2.00 [-6.49, 10.49] |  |
|  | Overweight/obesity | 6 | 471 | -1.69 [-6.35, 2.97] |  |
|  | T2DM | 3 | 209 | -0.41 [-6.89, 6.06] |  |
|  | Multiple | 2 | 133 | 0.50 [-5.27, 6.27] |  |

Abbreviations: CAD, coronary artery disease; MetS, metabolic syndrome; T2DM, type 2 diabetes mellitus.

# **Supplementary Table 12:** Results of sub-group analyses for systolic blood pressure

| **Sub-group analysis category** | **Sub-group** | **Number of analyses** | **Number of participants** | **Effect estimate** | **Test for sub-group differences** |
| --- | --- | --- | --- | --- | --- |
| Study design | Cross-over | 29 | 1966 | -0.79 [-1.73, 0.16] | Chi² = 2.40, df = 1 (P = 0.12), I² = 58.4% |
|  | Parallel | 42 | 4543 | 0.34 [-0.73, 1.41] |  |
| Nut group | Almond | 20 | 1575 | -0.12 [-1.90, 1.66] | Chi² = 8.32, df = 10 (P = 0.60), I² = 0% |
|  | Brazil nut | 3 | 290 | 3.90 [-1.51, 9.32] |  |
|  | Cashew nut | 2 | 348 | -1.70 [-4.64, 1.24] |  |
|  | Hazelnut | 2 | 163 | 2.32 [-3.06, 7.70] |  |
|  | Macadamia | 2 | 206 | -2.08 [-7.21, 3.05] |  |
|  | Mixed nuts | 9 | 715 | 1.20 [-1.20, 3.59] |  |
|  | Peanut | 4 | 675 | 0.03 [-2.04, 2.09] |  |
|  | Pecan | 3 | 213 | 0.01 [-4.11, 4.14] |  |
|  | Pistachio | 7 | 423 | -1.51 [-3.37, 0.35] |  |
|  | Walnut | 17 | 1801 | -0.24 [-1.04, 0.57] |  |
|  | Two different nuts | 2 | 100 | -1.96 [-6.65, 2.73] |  |
| Nut dose | ≤30 | 19 | 2035 | 0.82 [-0.68, 2.31] | Chi² = 2.43, df = 3 (P = 0.49), I² = 0% |
|  | 31-60 | 31 | 3129 | -0.76 [-2.11, 0.59] |  |
|  | >60 | 14 | 733 | 0.04 [-1.47, 1.56] |  |
|  | Nut oil, butter and flour either alone or in combination with nuts | 4 | 414 | 0.17 [-0.85, 1.18] |  |
| Study duration | <12 weeks | 35 | 2150 | 0.63 [-0.46, 1.72] | Chi² = 2.86, df = 1 (P = 0.09), I² = 65.0% |
|  | ≥12 weeks | 36 | 4359 | -0.62 [-1.59, 0.34] |  |
| Health status | CAD | 3 | 293 | -1.39 [-5.04, 2.26] | Chi² = 7.45, df = 8 (P = 0.49), I² = 0% |
|  | CVD and/or risk of CVD | 3 | 203 | 0.03 [-3.21, 3.27] |  |
|  | Healthy | 9 | 704 | 1.92 [-0.22, 4.07] |  |
|  | Hypercholesterolemia/ hyperlipidemia | 10 | 831 | -0.05 [-1.96, 1.87] |  |
|  | MetS and/or at risk of MetS | 8 | 1327 | -1.08 [-2.62, 0.46] |  |
|  | Overweight/obesity | 22 | 1793 | -0.23 [-1.66, 1.20] |  |
|  | Prediabetes | 5 | 353 | -1.24 [-3.46, 0.98] |  |
|  | T2DM | 8 | 687 | -0.94 [-2.39, 0.51] |  |
|  | Multiple | 3 | 318 | 2.25 [-4.58, 9.08] |  |

Abbreviations: CAD, coronary artery disease; CVD, cardiovascular disease; MetS, metabolic syndrome; T2DM, type 2 diabetes mellitus.

# **Supplementary Table 13:** Results of sub-group analyses for HDL cholesterol

| **Sub-group analysis category** | **Sub-group** | **Number of analyses** | **Number of participants** | **Effect estimate** | **Test for sub-group differences** |
| --- | --- | --- | --- | --- | --- |
| Study design | Cross-over | 56 | 3948 | 0.01 [-0.01, 0.02] | Chi² = 0.09, df = 1 (P = 0.76), I² = 0% |
|  | Parallel | 70 | 6154 | 0.00 [-0.02, 0.03] |  |
| Nut group | Almond | 31 | 2409 | -0.02 [-0.06, 0.02] | Chi² = 16.34, df = 10 (P = 0.09), I² = 38.8% |
|  | Brazil nut | 4 | 307 | -0.03 [-0.08, 0.02] |  |
|  | Cashew nut | 3 | 432 | 0.02 [-0.02, 0.07] |  |
|  | Hazelnut | 5 | 313 | 0.09 [-0.01, 0.19] |  |
|  | Macadamia | 6 | 410 | -0.03 [-0.08, 0.03] |  |
|  | Mixed nuts | 11 | 888 | 0.01 [-0.02, 0.03] |  |
|  | Peanut | 10 | 1021 | 0.03 [-0.02, 0.08] |  |
|  | Pecan | 6 | 295 | 0.03 [-0.03, 0.08] |  |
|  | Pistachio | 11 | 652 | -0.01 [-0.03, 0.02] |  |
|  | Walnut | 37 | 3275 | 0.01 [-0.01, 0.04] |  |
|  | Two different nuts | 2 | 100 | -0.07 [-0.14, -0.01] |  |
| Nut dose | ≤30 | 29 | 2568 | 0.01 [-0.01, 0.04] | Chi² = 1.21, df = 3 (P = 0.75), I² = 0% |
|  | 31-60 | 55 | 5143 | -0.00 [-0.03, 0.02] |  |
|  | >60 | 27 | 1286 | 0.01 [-0.02, 0.04] |  |
|  | Nut oil, butter and flour either alone or in combination with nuts | 11 | 835 | 0.02 [-0.03, 0.07] |  |
| Study duration | <12 weeks | 74 | 4166 | 0.01 [-0.02, 0.03] | Chi² = 0.04, df = 1 (P = 0.85), I² = 0% |
|  | ≥12 weeks | 52 | 5936 | 0.00 [-0.01, 0.02] |  |
| Health status | CAD | 4 | 360 | 0.02 [-0.02, 0.07] | Chi² = 17.14, df = 8 (P = 0.03), I² = 53.3% |
|  | CVD and/or risk of CVD | 3 | 203 | -0.02 [-0.11, 0.06] |  |
|  | Healthy | 28 | 2158 | -0.02 [-0.06, 0.02] |  |
|  | Hypercholesterolemia/ hyperlipidemia | 24 | 1474 | 0.02 [-0.02, 0.06] |  |
|  | MetS and/or at risk of MetS | 10 | 1481 | -0.04 [-0.06, -0.01] |  |
|  | Overweight/obesity | 31 | 2290 | 0.02 [-0.01, 0.04] |  |
|  | Prediabetes | 6 | 572 | -0.00 [-0.04, 0.03] |  |
|  | T2DM | 15 | 974 | 0.04 [0.00, 0.07] |  |
|  | Multiple | 5 | 590 | -0.00 [-0.06, 0.05] |  |

Abbreviations: CAD, coronary artery disease; CVD, cardiovascular disease; MetS, metabolic syndrome; T2DM, type 2 diabetes mellitus.

# **Supplementary Table 14:** Results of sub-group analyses for diastolic blood pressure

| **Sub-group analysis category** | **Sub-group** | **Number of analyses** | **Number of participants** | **Effect estimate** | **Test for sub-group differences** |
| --- | --- | --- | --- | --- | --- |
| Study design | Cross-over | 26 | 1724 | -0.34 [-0.97, 0.30] | Chi² = 0.68, df = 1 (P = 0.41), I² = 0% |
|  | Parallel | 41 | 4474 | 0.03 [-0.57, 0.64] |  |
| Nut group | Almond | 20 | 1641 | -0.58 [-1.58, 0.42] | Chi² = 9.41, df = 10 (P = 0.49), I² = 0% |
|  | Brazil nut | 3 | 290 | 1.72 [-0.00, 3.45] |  |
|  | Cashew nut | 2 | 348 | -0.25 [-2.80, 2.29] |  |
|  | Hazelnut | 2 | 163 | 1.89 [-1.34, 5.13] |  |
|  | Macadamia | 1 | 64 | -2.83 [-8.97, 3.31] |  |
|  | Mixed nuts | 8 | 654 | 0.12 [-1.16, 1.39] |  |
|  | Peanut | 4 | 675 | 0.07 [-1.29, 1.43] |  |
|  | Pecan | 3 | 213 | 1.69 [-1.70, 5.09] |  |
|  | Pistachio | 7 | 423 | -0.37 [-1.48, 0.74] |  |
|  | Walnut | 15 | 1627 | -0.38 [-1.14, 0.38] |  |
|  | Two different nuts | 2 | 100 | -0.38 [-3.50, 2.75] |  |
| Nut dose | ≤30 | 17 | 1890 | 0.21 [-0.62, 1.04] | Chi² = 5.02, df = 3 (P = 0.17), I² = 40.2% |
|  | 31-60 | 30 | 3003 | -0.24 [-0.91, 0.44] |  |
|  | >60 | 14 | 733 | -0.61 [-1.54, 0.32] |  |
|  | Nut oil, butter and flour either alone or in combination with nuts | 3 | 374 | 1.06 [-0.20, 2.33] |  |
| Study duration | <12 weeks | 32 | 1908 | 0.39 [-0.18, 0.96] | Chi² = 4.22, df = 1 (P = 0.04), I² = 76.1% |
|  | ≥12 weeks | 35 | 4290 | -0.43 [-0.95, 0.10] |  |
| Health status | CAD | 3 | 293 | -0.57 [-3.90, 2.76] | Chi² = 21.33, df = 8 (P = 0.006), I² = 62.5% |
|  | CVD and/or risk of CVD | 3 | 203 | 1.12 [-3.19, 5.44] |  |
|  | Healthy | 8 | 562 | 0.98 [0.55, 1.41] |  |
|  | Hypercholesterolemia/ hyperlipidemia | 8 | 609 | -0.50 [-1.97, 0.97] |  |
|  | MetS and/or at risk of MetS | 7 | 1267 | 0.28 [-0.75, 1.30] |  |
|  | Overweight/obesity | 21 | 1725 | -0.63 [-1.44, 0.19] |  |
|  | Prediabetes | 5 | 353 | 0.18 [-1.12, 1.47] |  |
|  | T2DM | 8 | 686 | -0.79 [-1.72, 0.14] |  |
|  | Multiple | 4 | 500 | 0.91 [-1.75, 3.58] |  |

Abbreviations: CAD, coronary artery disease; CVD, cardiovascular disease; MetS, metabolic syndrome; T2DM, type 2 diabetes mellitus.

# **Supplementary Table 15:** Health Canada quality appraisal of included studies

Health Canada Quality Appraisal Tools for included studies (Health Canada, 2009)

| **Quality Appraisal Tool for Experimental Studies** | | | |
| --- | --- | --- | --- |
| **Study reference:** Abarzafard et al. (2014) | | | |
| **Item** | **Question** | **Score** | |
|  |  | YES (1) | NO /NR(0) |
| 1. Inclusion/exclusion criteria | Were the inclusion and exclusion criteria for study participation reported? (eg. Age greater than 50 years, no history of heart disease)? | 1 |  |
| 2. Group allocation | Was the study described as randomized? | 1 |  |
|  | Was the randomization method reported? | 1 |  |
|  | Was the randomization appropriate? | 1 |  |
|  | Was the allocation concealed? |  | 0 (NR) |
| 3. Blinding | Were the study subjects blinded to the intervention received? |  | 0 (NR) |
|  | Were the researcher personnel blinded to the intervention received by the subjects? |  | 0 (NR) |
| 4. Attrition | Were attrition numerically reported? | 1 |  |
|  | Were the reasons for withdrawals and dropouts provided? | 1 |  |
| 5. Exposure/intervention | Was the type of food described (eg. Composition, matrix)? | 1 |  |
|  | Was the amount of food described (i.e. dose)? | 1 |  |
| 6. Health effect | Was the methodology used to measure the health effect reported? | 1 |  |
| 7. Statistical analysis | Was between group statistical analysis of the health effect reported? | 1 |  |
|  | Was an intention-to-treat analysis conducted? |  | 0 |
| 8. Potential confounders | Were potential confounders of the food health relationship considered? | 1 |  |
| TOTAL SCORE (maximum of 15) | | 11/15 |  |
| Higher quality (Score ≥ 8) | | X | |
| Lower quality (Score ≤ 7) | |  | |

Confounders: fasting blood sugar, BMI and systolic blood pressure

| **Quality Appraisal Tool for Experimental Studies** | | | |
| --- | --- | --- | --- |
| **Study reference:** Abbaspour et al. (2019) | |  | |
| **Item** | **Question** | **Score** | |
|  |  | YES (1) | NO /NR(0) |
| 1. Inclusion/ Exclusion Criteria | Were the inclusion and/or exclusion criteria for study participation reported (*e.g.*, age greater than 50 years, no history of heart disease)? | 1 |  |
| 2. Group Allocation | Was the study described as randomized? | 1 |  |
|  | Was the randomization method reported? |  | 0 (NR) |
|  | Was the randomization method appropriate? |  | 0 (NR) |
|  | Was allocation concealed? |  | 0 (NR) |
| 3. Blinding | Were the study subjects blinded to the intervention received? |  | 0 (NR) |
|  | Were the research personnel blinded to the intervention received by the subjects? |  | 0 (NR) |
| 4. Attrition | Was attrition numerically reported? | 1 |  |
|  | Were the reasons for withdrawals and dropouts provided? | 1 |  |
| 5. Exposure/  Intervention | Was the type of food described (e.g., composition, matrix)? | 1 |  |
|  | Was the amount of food described (i.e., dose)? | 1 |  |
| 6. Health Effect | Was the methodology used to measure the health effect reported? | 1 |  |
| 7. Statistical Analysis | Was a between-group statistical analysis of the health effect conducted (*i.e.*, control vs. intervention)? | 1 |  |
|  | Was an intention-to-treat analysis conducted? |  | 0 |
| 8. Potential Confounders | Were potential confounders of the food/health relationship considered? | 1 |  |
| TOTAL SCORE (maximum of 15): | | 9/15 | |
| Higher quality (Score ≥ 8) | | X | |
| Lower quality (Score ≤ 7) | |  | |

Confounders: Participants were asked to continue with their usual dietary habits and physical activities during the study

| **Quality Appraisal Tool for Experimental Studies** | | | |
| --- | --- | --- | --- |
| **Study reference:** Agebratt et al (2016) | | | |
| **Item** | **Question** | **Score** | |
|  |  | YES (1) | NO /NR(0) |
| 1. Inclusion/exclusion criteria | Were the inclusion and exclusion criteria for study participation reported? (eg. Age greater than 50 years, no history of heart disease)? | 1 |  |
| 2. Group allocation | Was the study described as randomized? | 1 |  |
|  | Was the randomization method reported? | 1 |  |
|  | Was the randomization appropriate? | 1 |  |
|  | Was the allocation concealed? |  | 0(NR) |
| 3. Blinding | Were the study subjects blinded to the intervention received? |  | 0(NR) |
|  | Were the researcher personnel blinded to the intervention received by the subjects? | 1 |  |
| 4. Attrition | Were attrition numerically reported? | 1 |  |
|  | Were the reasons for withdrawals and dropouts provided? | 1 |  |
| 5. Exposure/intervention | Was the type of food described (e.g.. Composition, matrix)? | 1 |  |
|  | Was the amount of food described (i.e. dose)? | 1 |  |
| 6. Health effect | Was the methodology used to measure the health effect reported? | 1 |  |
| 7. Statistical analysis | Was between group statistical analysis of the health effect reported? | 1 |  |
|  | Was an intention-to-treat analysis conducted? |  | 0(NR) |
| 8. Potential confounders | Were potential confounders of the food health relationship considered? |  | 0 |
| TOTAL SCORE (maximum of 15) | | 11 |  |
| Higher quality (Score ≥ 8) | | X | |
| Lower quality (Score ≤ 7) | |  | |

Confounders: Although BMIs at baseline of two groups were similar, there was a 7kg difference on body weight between groups at baseline. It may be a confounder of basal metabolic rate measurement, as there was 144kcal/24h difference between groups at baseline.

| **Quality Appraisal Tool for Experimental Studies** | | | |
| --- | --- | --- | --- |
| **Study reference:**   Al Abdrabalnabi et al. (2020) | |  | |
| **Item** | **Question** | **Score** | |
|  |  | YES (1) | NO /NR(0) |
| 1. Inclusion/ Exclusion Criteria | Were the inclusion and/or exclusion criteria for study participation reported (*e.g.*, age greater than 50 years, no history of heart disease)? | 1 |  |
| 2. Group Allocation | Was the study described as randomized? | 1 |  |
|  | Was the randomization method reported? | 1 |  |
|  | Was the randomization method appropriate? | 1 |  |
|  | Was allocation concealed? |  | 0 (NR) |
| 3. Blinding | Were the study subjects blinded to the intervention received? |  | 0 (NR) |
|  | Were the research personnel blinded to the intervention received by the subjects? | 1 |  |
| 4. Attrition | Was attrition numerically reported? | 1 |  |
|  | Were the reasons for withdrawals and dropouts provided?^4^ | 1 |  |
| 5. Exposure/  Intervention | Was the type of food described (e.g., composition, matrix)? | 1 |  |
|  | Was the amount of food described (i.e., dose)? | 1 |  |
| 6. Health Effect | Was the methodology used to measure the health effect reported? | 1 |  |
| 7. Statistical Analysis | Was a between-group statistical analysis of the health effect conducted (*i.e.*, control vs. intervention)? | 1 |  |
|  | Was an intention-to-treat analysis conducted? |  | 0 |
| 8. Potential Confounders | Were potential confounders of the food/health relationship considered?^6^ | 1 |  |
| TOTAL SCORE (maximum of 15) | | 12/15 | |
| Higher quality (Score ≥ 8) | | X | |
| Lower quality (Score ≤ 7) | |  | |

Confounders: Physical activity collected

| **Quality Appraisal Tool for Experimental Studies** | | | |
| --- | --- | --- | --- |
| **Study reference: Alves et al. (2014)** | | | |
| **Item** | **Question** | **Score** | |
|  |  | YES (1) | NO /NR(0) |
| 1. Inclusion/exclusion criteria | Were the inclusion and exclusion criteria for study participation reported? (eg. Age greater than 50 years, no history of heart disease)? | 1 |  |
| 2. Group allocation | Was the study described as randomized? | 1 |  |
|  | Was the randomization method reported? |  | 0 NR |
|  | Was the randomization appropriate? |  | 0 NR |
|  | Was the allocation concealed? |  | 0 NR |
| 3. Blinding | Were the study subjects blinded to the intervention received? |  | 0 |
|  | Were the researcher personnel blinded to the intervention received by the subjects? |  | 0 |
| 4. Attrition | Were attrition numerically reported? | 1 |  |
|  | Were the reasons for withdrawals and dropouts provided? | 1 |  |
| 5. Exposure/intervention | Was the type of food described (eg. Composition, matrix)? | 1 |  |
|  | Was the amount of food described (i.e. dose)? | 1 |  |
| 6. Health effect | Was the methodology used to measure the health effect reported? | 1 |  |
| 7. Statistical analysis | Was between group statistical analysis of the health effect reported? | 1 |  |
|  | Was an intention-to-treat analysis conducted? |  | 0 |
| 8. Potential confounders | Were potential confounders of the food health relationship considered? | 1 |  |
| TOTAL SCORE (maximum of 15) | | 9/15 |  |
| Higher quality (Score ≥ 8) | | X | |
| Lower quality (Score ≤ 7) | |  | |

Confounders: Assessed whether changes in biochemical variables occurred independently to changes in body composition. Dietary data compared between groups, only significant difference was in MUFA content of diet

| **Quality Appraisal Tool for Experimental Studies** | | | |
| --- | --- | --- | --- |
| **Study reference:** Baer and Novotny (2019) | |  | |
| **Item** | **Question** | **Score** | |
|  |  | YES (1) | NO /NR(0) |
| 1. Inclusion/ Exclusion Criteria | Were the inclusion and/or exclusion criteria for study participation reported (*e.g.*, age greater than 50 years, no history of heart disease)? | 1 |  |
| 2. Group Allocation | Was the study described as randomized? | 1 |  |
|  | Was the randomization method reported? | 1 |  |
|  | Was the randomization method appropriate? | 1 |  |
|  | Was allocation concealed? | 1 |  |
| 3. Blinding | Were the study subjects blinded to the intervention received? |  | 0 (NR) |
|  | Were the research personnel blinded to the intervention received by the subjects? | 1 |  |
| 4. Attrition | Was attrition numerically reported? | 1 |  |
|  | Were the reasons for withdrawals and dropouts provided? | 1 |  |
| 5. Exposure/  Intervention | Was the type of food described (e.g., composition, matrix)? | 1 |  |
|  | Was the amount of food described (i.e., dose)? | 1 |  |
| 6. Health Effect | Was the methodology used to measure the health effect reported? | 1 |  |
| 7. Statistical Analysis | Was a between-group statistical analysis of the health effect conducted (*i.e.*, control vs. intervention)? | 1 |  |
|  | Was an intention-to-treat analysis conducted? |  | 0 |
| 8. Potential Confounders | Were potential confounders of the food/health relationship considered? | 1 |  |
| TOTAL SCORE (maximum of 15): | | 13/15 | |
| Higher quality (Score ≥ 8) | | X | |
| Lower quality (Score ≤ 7) | |  | |

Confounders: not applicable as randomised cross-over design

| **Quality Appraisal Tool for Experimental Studies** | | | |
| --- | --- | --- | --- |
| **Study reference:** Bamberger et al (2017) | | | |
| **Item** | **Question** | **Score** | |
|  |  | YES (1) | NO /NR(0) |
| 1. Inclusion/exclusion criteria | Were the inclusion and exclusion criteria for study participation reported? (eg. Age greater than 50 years, no history of heart disease)? | 1 |  |
| 2. Group allocation | Was the study described as randomized? | 1 |  |
|  | Was the randomization method reported? | 1 |  |
|  | Was the randomization appropriate? | 1 |  |
|  | Was the allocation concealed? | 1 |  |
| 3. Blinding | Were the study subjects blinded to the intervention received? |  | 0(NR) |
|  | Were the researcher personnel blinded to the intervention received by the subjects? | 1 |  |
| 4. Attrition | Were attrition numerically reported? | 1 |  |
|  | Were the reasons for withdrawals and dropouts provided? | 1 |  |
| 5. Exposure/intervention | Was the type of food described (e.g.. Composition, matrix)? | 1 |  |
|  | Was the amount of food described (i.e. dose)? | 1 |  |
| 6. Health effect | Was the methodology used to measure the health effect reported? | 1 |  |
| 7. Statistical analysis | Was between group statistical analysis of the health effect reported? | 1 |  |
|  | Was an intention-to-treat analysis conducted? | 1 |  |
| 8. Potential confounders | Were potential confounders of the food health relationship considered? | 1 |  |
| TOTAL SCORE (maximum of 15) | | 14/15 |  |
| Higher quality (Score ≥ 8) | | X | |
| Lower quality (Score ≤ 7) | |  | |

Confounders: The adherence of micronutrient composition in the diet was evaluated.

| **Quality Appraisal Tool for Experimental Studies** | | | |
| --- | --- | --- | --- |
| **Study reference:** Barbour et al (2015) | | | |
| **Item** | **Question** | **Score** | |
|  |  | YES (1) | NO /NR(0) |
| 1. Inclusion/exclusion criteria | Were the inclusion and exclusion criteria for study participation reported? (eg. Age greater than 50 years, no history of heart disease)? | 1 |  |
| 2. Group allocation | Was the study described as randomized? | 1 |  |
|  | Was the randomization method reported? | 1 |  |
|  | Was the randomization appropriate? | 1 |  |
|  | Was the allocation concealed? | 1 |  |
| 3. Blinding | Were the study subjects blinded to the intervention received? |  | 0(NR) |
|  | Were the researcher personnel blinded to the intervention received by the subjects? | 1 |  |
| 4. Attrition | Were attrition numerically reported? | 1 |  |
|  | Were the reasons for withdrawals and dropouts provided? | 1 |  |
| 5. Exposure/intervention | Was the type of food described (e.g.. Composition, matrix)? | 1 |  |
|  | Was the amount of food described (i.e. dose)? | 1 |  |
| 6. Health effect | Was the methodology used to measure the health effect reported? | 1 |  |
| 7. Statistical analysis | Was between group statistical analysis of the health effect reported? | 1 |  |
|  | Was an intention-to-treat analysis conducted? |  | 0 |
| 8. Potential confounders | Were potential confounders of the food health relationship considered? | 1 |  |
| TOTAL SCORE (maximum of 15) | | 13/15 |  |
| Higher quality (Score ≥ 8) | | X | |
| Lower quality (Score ≤ 7) | |  | |

Confounders: Physical activity was measured.

| **Quality Appraisal Tool for Experimental Studies** | | | |
| --- | --- | --- | --- |
| **Study reference:** Berryman et al (2015) | | | |
| **Item** | **Question** | **Score** | |
|  |  | YES (1) | NO /NR(0) |
| 1. Inclusion/exclusion criteria | Were the inclusion and exclusion criteria for study participation reported? (eg. Age greater than 50 years, no history of heart disease)? | 1 |  |
| 2. Group allocation | Was the study described as randomized? | 1 |  |
|  | Was the randomization method reported? | 1 |  |
|  | Was the randomization appropriate? | 1 |  |
|  | Was the allocation concealed? | 1 |  |
| 3. Blinding | Were the study subjects blinded to the intervention received? |  | 0(NR) |
|  | Were the researcher personnel blinded to the intervention received by the subjects? |  | 0(NR) |
| 4. Attrition | Were attrition numerically reported? | 1 |  |
|  | Were the reasons for withdrawals and dropouts provided? | 1 |  |
| 5. Exposure/intervention | Was the type of food described (e.g. Composition, matrix)? | 1 |  |
|  | Was the amount of food described (i.e. dose)? | 1 |  |
| 6. Health effect | Was the methodology used to measure the health effect reported? | 1 |  |
| 7. Statistical analysis | Was between group statistical analysis of the health effect reported? | 1 |  |
|  | Was an intention-to-treat analysis conducted? |  | 0 |
| 8. Potential confounders | Were potential confounders of the food health relationship considered? | 1 |  |
| TOTAL SCORE (maximum of 15) | | 12/15 |  |
| Higher quality (Score ≥ 8) | | X | |
| Lower quality (Score ≤ 7) | |  | |

Confounders: No identified potential confounders, the adherence of dietary intervention prescription was reported.

| **Quality Appraisal Tool for Experimental Studies** | | | |
| --- | --- | --- | --- |
| **Study reference:**  Bowen et al (2019) | |  | |
| **Item** | **Question** | **Score** | |
|  |  | YES (1) | NO /NR(0) |
| 1. Inclusion/ Exclusion Criteria | Were the inclusion and/or exclusion criteria for study participation reported (*e.g.*, age greater than 50 years, no history of heart disease)? | 1 |  |
| 2. Group Allocation | Was the study described as randomized? | 1 |  |
|  | Was the randomization method reported? | 1 |  |
|  | Was the randomization method appropriate? | 1 |  |
|  | Was allocation concealed? | 1 |  |
| 3. Blinding | Were the study subjects blinded to the intervention received? |  | 0 (NR) |
|  | Were the research personnel blinded to the intervention received by the subjects? | 1 |  |
| 4. Attrition | Was attrition numerically reported? | 1 |  |
|  | Were the reasons for withdrawals and dropouts provided? | 1 |  |
| 5. Exposure/  Intervention | Was the type of food described (e.g., composition, matrix)? | 1 |  |
|  | Was the amount of food described (i.e., dose)? | 1 |  |
| 6. Health Effect | Was the methodology used to measure the health effect reported? | 1 |  |
| 7. Statistical Analysis | Was a between-group statistical analysis of the health effect conducted (*i.e.*, control vs. intervention)? | 1 |  |
|  | Was an intention-to-treat analysis conducted? | 1 |  |
| 8. Potential Confounders | Were potential confounders of the food/health relationship considered? | 1 |  |
| TOTAL SCORE (maximum of 15): | | 14/15 | |
| Higher quality (Score ≥ 8) | | X | |
| Lower quality (Score ≤ 7) | |  | |

 Confounders: participants instructed to maintain usual diet and physical activity patterns

| **Quality Appraisal Tool for Experimental Studies** | | | |
| --- | --- | --- | --- |
| **Study reference:** Burns-Whitmore et al. (2014) | | | |
| **Item** | **Question** | **Score** | |
|  |  | YES (1) | NO /NR(0) |
| 1. Inclusion/exclusion criteria | Were the inclusion and exclusion criteria for study participation reported? (eg. Age greater than 50 years, no history of heart disease)? | 1 |  |
| 2. Group allocation | Was the study described as randomized? | 1 |  |
|  | Was the randomization method reported? |  | 0 |
|  | Was the randomization appropriate? |  | NR 0 |
|  | Was the allocation concealed? |  | NR 0 |
| 3. Blinding | Were the study subjects blinded to the intervention received? |  | 0 |
|  | Were the researcher personnel blinded to the intervention received by the subjects? |  | 0 |
| 4. Attrition | Were attrition numerically reported? | 1 |  |
|  | Were the reasons for withdrawals and dropouts provided? | 1 |  |
| 5. Exposure/intervention | Was the type of food described (eg. Composition, matrix)? | 1 |  |
|  | Was the amount of food described (i.e. dose)? | 1 |  |
| 6. Health effect | Was the methodology used to measure the health effect reported? | 1 |  |
| 7. Statistical analysis | Was between group statistical analysis of the health effect reported? | 1 |  |
|  | Was an intention-to-treat analysis conducted? |  | 0 |
| 8. Potential confounders | Were potential confounders of the food health relationship considered? | 1 |  |
| TOTAL SCORE (maximum of 15): | | 9/15 |  |
| Higher quality (Score ≥ 8) | | X | |
| Lower quality (Score ≤ 7) | |  | |

Confounders: Dietary intake investigated (no difference in energy intake between periods), analysis adjusted for baseline outcome value

| **Quality Appraisal Tool for Experimental Studies** | | | |
| --- | --- | --- | --- |
| **Study reference:** Campos et al (2020) | |  | |
| **Item** | **Question** | **Score** | |
|  |  | YES (1) | NO /NR(0) |
| 1. Inclusion/ Exclusion Criteria | Were the inclusion and/or exclusion criteria for study participation reported (*e.g.*, age greater than 50 years, no history of heart disease)? | 1 |  |
| 2. Group Allocation | Was the study described as randomized? | 1 |  |
|  | Was the randomization method reported? | 1 |  |
|  | Was the randomization method appropriate? | 1 |  |
|  | Was allocation concealed? | 1 |  |
| 3. Blinding | Were the study subjects blinded to the intervention received? | 1 |  |
|  | Were the research personnel blinded to the intervention received by the subjects? | 1 |  |
| 4. Attrition | Was attrition numerically reported? | 1 |  |
|  | Were the reasons for withdrawals and dropouts provided? | 1 |  |
| 5. Exposure/  Intervention | Was the type of food described (e.g., composition, matrix)? | 1 |  |
|  | Was the amount of food described (i.e., dose)? | 1 |  |
| 6. Health Effect | Was the methodology used to measure the health effect reported? | 1 |  |
| 7. Statistical Analysis | Was a between-group statistical analysis of the health effect conducted (*i.e.*, control vs. intervention)? | 1 |  |
|  | Was an intention-to-treat analysis conducted? | 1 |  |
| 8. Potential Confounders | Were potential confounders of the food/health relationship considered? | 1 |  |
| TOTAL SCORE (maximum of 15): | | 15/15 | |
| Higher quality (Score ≥ 8) | | X | |
| Lower quality (Score ≤ 7) | |  | |

Confounders: Physical activity collected

| **Quality Appraisal Tool for Experimental Studies** | | | |
| --- | --- | --- | --- |
| **Study reference:** Canales et al. (2007) | | | |
| **Item** | **Question** | **Score** | |
|  |  | YES (1) | NO /NR(0) |
| 1. Inclusion/exclusion criteria | Were the inclusion and exclusion criteria for study participation reported? (eg. Age greater than 50 years, no history of heart disease)? | 1 |  |
| 2. Group allocation | Was the study described as randomized? | 1 |  |
|  | Was the randomization method reported? |  | N0 0 |
|  | Was the randomization appropriate? |  | NR 0 |
|  | Was the allocation concealed? |  | 0 |
| 3. Blinding | Were the study subjects blinded to the intervention received? |  | 0 |
|  | Were the researcher personnel blinded to the intervention received by the subjects? |  | 0 |
| 4. Attrition | Were attrition numerically reported? | 1 |  |
|  | Were the reasons for withdrawals and dropouts provided? | 1 |  |
| 5. Exposure/intervention | Was the type of food described (eg. Composition, matrix)? | 1 |  |
|  | Was the amount of food described (i.e. dose)? | 1 |  |
| 6. Health effect | Was the methodology used to measure the health effect reported? | 1 |  |
| 7. Statistical analysis | Was between group statistical analysis of the health effect reported? | 1 |  |
|  | Was an intention-to-treat analysis conducted? |  | 0 |
| 8. Potential confounders | Were potential confounders of the food health relationship considered? |  | 0 |
| TOTAL SCORE (maximum of 15): | | 8/15 |  |
| Higher quality (Score ≥ 8) | | X | |
| Lower quality (Score ≤ 7) | |  | |

Confounders: Physical activity effects not considered

| **Quality Appraisal Tool for Experimental Studies** | | | |
| --- | --- | --- | --- |
| **Study reference:** Carvalho et al (2015) | |  | |
| **Item** | **Question** | **Score** | |
|  |  | YES (1) | NO /NR(0) |
| 1. Inclusion/ Exclusion Criteria | Were the inclusion and/or exclusion criteria for study participation reported (*e.g.*, age greater than 50 years, no history of heart disease)? | 1 |  |
| 2. Group Allocation | Was the study described as randomized? | 1 |  |
|  | Was the randomization method reported? | 1 |  |
|  | Was the randomization method appropriate? | 1 |  |
|  | Was allocation concealed? | 1 |  |
| 3. Blinding | Were the study subjects blinded to the intervention received? | 1 |  |
|  | Were the research personnel blinded to the intervention received by the subjects? | 1 |  |
| 4. Attrition | Was attrition numerically reported? | 1 |  |
|  | Were the reasons for withdrawals and dropouts provided? | 1 |  |
| 5. Exposure/  Intervention | Was the type of food described (e.g., composition, matrix)? | 1 |  |
|  | Was the amount of food described (i.e., dose)? | 1 |  |
| 6. Health Effect | Was the methodology used to measure the health effect reported? | 1 |  |
| 7. Statistical Analysis | Was a between-group statistical analysis of the health effect conducted (*i.e.*, control vs. intervention)? | 1 |  |
|  | Was an intention-to-treat analysis conducted? |  | 0 |
| 8. Potential Confounders | Were potential confounders of the food/health relationship considered? | 1 |  |
| TOTAL SCORE (maximum of 15): | | 14/15 | |
| Higher quality (Score ≥ 8) | | X | |
| Lower quality (Score ≤ 7) | |  | |

Confounders: no differences at baseline, physical activity recorded and taken into account

| **Quality Appraisal Tool for Experimental Studies** | | | |
| --- | --- | --- | --- |
| **Study reference:** Casas-Agustench et al. (2011) | | | |
| **Item** | **Question** | **Score** | |
|  |  | YES (1) | NO /NR(0) |
| 1. Inclusion/exclusion criteria | Were the inclusion and exclusion criteria for study participation reported? (eg. Age greater than 50 years, no history of heart disease)? | 1 |  |
| 2. Group allocation | Was the study described as randomized? | 1 |  |
|  | Was the randomization method reported? |  | 0 |
|  | Was the randomization appropriate? |  | 0 (NR) |
|  | Was the allocation concealed? |  | 0 (NR) |
| 3. Blinding | Were the study subjects blinded to the intervention received? |  | 0 (NR) |
|  | Were the researcher personnel blinded to the intervention received by the subjects? |  | 0 |
| 4. Attrition | Were attrition numerically reported? | 1 |  |
|  | Were the reasons for withdrawals and dropouts provided? | 1 |  |
| 5. Exposure/intervention | Was the type of food described (eg. Composition, matrix)? | 1 |  |
|  | Was the amount of food described (i.e. dose)? | 1 |  |
| 6. Health effect | Was the methodology used to measure the health effect reported? | 1 |  |
| 7. Statistical analysis | Was between group statistical analysis of the health effect reported? | 1 |  |
|  | Was an intention-to-treat analysis conducted? |  | 0 |
| 8. Potential confounders | Were potential confounders of the food health relationship considered?^1^ | 1 |  |
| TOTAL SCORE (maximum of 15): | | 9/15 |  |
| Higher quality (Score ≥ 8) | | X | |
| Lower quality (Score ≤ 7) | |  | |

Confounders: Randomisation stratified by sex and age (≤ or >50yrs). Body weight changes used as covariate in ANCOVA

| **Quality Appraisal Tool for Experimental Studies** | | | |
| --- | --- | --- | --- |
| **Study reference:** Chen et al (2015) | | | |
| **Item** | **Question** | **Score** | |
|  |  | YES (1) | NO/NR(0) |
| 1. Inclusion/exclusion criteria | Were the inclusion and exclusion criteria for study participation reported? (eg. Age greater than 50 years, no history of heart disease)? | 1 |  |
| 2. Group allocation | Was the study described as randomized? | 1 |  |
|  | Was the randomization method reported? | 1 |  |
|  | Was the randomization appropriate? | 1 |  |
|  | Was the allocation concealed? | 1 |  |
| 3. Blinding | Were the study subjects blinded to the intervention received? |  | 0(NR) |
|  | Were the researcher personnel blinded to the intervention received by the subjects? |  | 0(NR) |
| 4. Attrition | Were attrition numerically reported? | 1 |  |
|  | Were the reasons for withdrawals and dropouts provided? | 1 |  |
| 5. Exposure/intervention | Was the type of food described (e.g.. Composition, matrix)? | 1 |  |
|  | Was the amount of food described (i.e. dose)? | 1 |  |
| 6. Health effect | Was the methodology used to measure the health effect reported? | 1 |  |
| 7. Statistical analysis | Was between group statistical analysis of the health effect reported? | 1 |  |
|  | Was an intention-to-treat analysis conducted? |  | 0 |
| 8. Potential confounders | Were potential confounders of the food health relationship considered? |  | 0 |
| TOTAL SCORE (maximum of 15): | | 11/15 |  |
| Higher quality (Score ≥ 8) | | X | |
| Lower quality (Score ≤ 7) | |  | |

Confounders: There was a significant difference on caloric, protein, carbohydrate intakes between control diet and almond diet, although the study protocol described to replace the almond intake to maintain isocaloric intake in the diet.

| **Quality Appraisal Tool for Experimental Studies** | | | |
| --- | --- | --- | --- |
| **Study reference:** Chen et al (2017) | | | |
| **Item** | **Question** | **Score** | |
|  |  | YES (1) | NO /NR(0) |
| 1. Inclusion/exclusion criteria | Were the inclusion and exclusion criteria for study participation reported? (eg. Age greater than 50 years, no history of heart disease)? | 1 |  |
| 2. Group allocation | Was the study described as randomized? | 1 |  |
|  | Was the randomization method reported? | 1 |  |
|  | Was the randomization appropriate? | 1 |  |
|  | Was the allocation concealed? | 1 |  |
| 3. Blinding | Were the study subjects blinded to the intervention received? |  | 0(NR) |
|  | Were the researcher personnel blinded to the intervention received by the subjects? |  | 0(NR) |
| 4. Attrition | Were attrition numerically reported? | 1 |  |
|  | Were the reasons for withdrawals and dropouts provided? | 1 |  |
| 5. Exposure/intervention | Was the type of food described (e.g.. Composition, matrix)? | 1 |  |
|  | Was the amount of food described (i.e. dose)? | 1 |  |
| 6. Health effect | Was the methodology used to measure the health effect reported? | 1 |  |
| 7. Statistical analysis | Was between group statistical analysis of the health effect reported? | 1 |  |
|  | Was an intention-to-treat analysis conducted? |  | 0 |
| 8. Potential confounders | Were potential confounders of the food health relationship considered? | 1 |  |
| TOTAL SCORE (maximum of 15): | | 13/15 |  |
| Higher quality (Score ≥ 8) | | X | |
| Lower quality (Score ≤ 7) | |  | |

Confounders: No identified potential confounders. Body weight was monitored weekly to adjust caloric intake to maintain weight during the study.

| **Quality Appraisal Tool for Experimental Studies** | | | |
| --- | --- | --- | --- |
| **Study reference:** Chen et al (2020) | |  | |
| **Item** | **Question** | **Score** | |
|  |  | YES (1) | NO /NR(0) |
| 1. Inclusion/ Exclusion Criteria | Were the inclusion and/or exclusion criteria for study participation reported (*e.g.*, age greater than 50 years, no history of heart disease)? | 1 |  |
| 2. Group Allocation | Was the study described as randomized? | 1 |  |
|  | Was the randomization method reported? | 1 |  |
|  | Was the randomization method appropriate? | 1 |  |
|  | Was allocation concealed? |  | 0 (NR) |
| 3. Blinding | Were the study subjects blinded to the intervention received? | 1 |  |
|  | Were the research personnel blinded to the intervention received by the subjects? | 1 |  |
| 4. Attrition | Was attrition numerically reported? | 1 |  |
|  | Were the reasons for withdrawals and dropouts provided? |  | 0 |
| 5. Exposure/  Intervention | Was the type of food described (e.g., composition, matrix)? | 1 |  |
|  | Was the amount of food described (i.e., dose)? | 1 |  |
| 6. Health Effect | Was the methodology used to measure the health effect reported? | 1 |  |
| 7. Statistical Analysis | Was a between-group statistical analysis of the health effect conducted (*i.e.*, control vs. intervention)? | 1 |  |
|  | Was an intention-to-treat analysis conducted? | 1 |  |
| 8. Potential Confounders | Were potential confounders of the food/health relationship considered? |  | 0 (NR) |
| TOTAL SCORE (maximum of 15): | | 12/15 | |
| Higher quality (Score ≥ 8) | | X | |
| Lower quality (Score ≤ 7) | |  | |

Confounders: changes in lifestyle factors not reported

| **Quality Appraisal Tool for Experimental Studies** | | | |
| --- | --- | --- | --- |
| **Study reference:** Chisholm et al. (1998) | | | |
| **Item** | **Question** | **Score** | |
|  |  | YES (1) | NO /NR(0) |
| 1. Inclusion/exclusion criteria | Were the inclusion and exclusion criteria for study participation reported? (eg. Age greater than 50 years, no history of heart disease)? | 1 |  |
| 2. Group allocation | Was the study described as randomized? | 1 |  |
|  | Was the randomization method reported? |  | 0 |
|  | Was the randomization appropriate? |  | 0 |
|  | Was the allocation concealed? |  | 0 |
| 3. Blinding | Were the study subjects blinded to the intervention received? |  | 0 |
|  | Were the researcher personnel blinded to the intervention received by the subjects? |  | 0 |
| 4. Attrition | Were attrition numerically reported? | 1 |  |
|  | Were the reasons for withdrawals and dropouts provided? | 1 |  |
| 5. Exposure/intervention | Was the type of food described (eg. Composition, matrix)? | 1 |  |
|  | Was the amount of food described (i.e. dose)? | 1 |  |
| 6. Health effect | Was the methodology used to measure the health effect reported? | 1 |  |
| 7. Statistical analysis | Was between group statistical analysis of the health effect reported? | 1 |  |
|  | Was an intention-to-treat analysis conducted? |  | 0 |
| 8. Potential confounders | Were potential confounders of the food health relationship considered? | 1 |  |
| TOTAL SCORE (maximum of 15): | | 9/15 |  |
| Higher quality (Score ≥ 8) | | X | |
| Lower quality (Score ≤ 7) | |  | |

Confounders: No significant difference in energy intake between study periods

| **Quality Appraisal Tool for Experimental Studies** | | | |
| --- | --- | --- | --- |
| **Study reference: Chisholm et al. (2005)** | | | |
| **Item** | **Question** | **Score** | |
|  |  | YES (1) | NO /NR(0) |
| 1. Inclusion/exclusion criteria | Were the inclusion and exclusion criteria for study participation reported? (eg. Age greater than 50 years, no history of heart disease)? | 1 |  |
| 2. Group allocation | Was the study described as randomized? | 1 |  |
|  | Was the randomization method reported? |  | 0 No |
|  | Was the randomization appropriate? |  | 0 NR |
|  | Was the allocation concealed? |  | 0NR |
| 3. Blinding | Were the study subjects blinded to the intervention received? |  | 0 |
|  | Were the researcher personnel blinded to the intervention received by the subjects? |  | 0 |
| 4. Attrition | Were attrition numerically reported? |  | 0 |
|  | Were the reasons for withdrawals and dropouts provided? |  | 0 |
| 5. Exposure/intervention | Was the type of food described (eg. Composition, matrix)? | 1 |  |
|  | Was the amount of food described (i.e. dose)? | 1 |  |
| 6. Health effect | Was the methodology used to measure the health effect reported? | 1 |  |
| 7. Statistical analysis | Was between group statistical analysis of the health effect reported? | 1 |  |
|  | Was an intention-to-treat analysis conducted? |  | 0 |
| 8. Potential confounders | Were potential confounders of the food health relationship considered? |  | 0 |
| TOTAL SCORE (maximum of 15): | | 6/15 |  |
| Higher quality (Score ≥ 8) | |  | |
| Lower quality (Score ≤ 7) | | X | |

Confounders: No information on physical activity

| **Quality Appraisal Tool for Experimental Studies** | | | |
| --- | --- | --- | --- |
| **Study reference:** Choudhury et al. (2014) | | | |
| **Item** | **Question** | **Score** | |
|  |  | YES (1) | NO /NR(0) |
| 1. Inclusion/exclusion criteria | Were the inclusion and exclusion criteria for study participation reported? (eg. Age greater than 50 years, no history of heart disease)? | 1 |  |
| 2. Group allocation | Was the study described as randomized? |  | 0 unclear |
|  | Was the randomization method reported? |  | 0 |
|  | Was the randomization appropriate? |  | 0 |
|  | Was the allocation concealed? |  | 0 |
| 3. Blinding | Were the study subjects blinded to the intervention received? |  | 0 |
|  | Were the researcher personnel blinded to the intervention received by the subjects? |  | 0 |
| 4. Attrition | Were attrition numerically reported? | 1 |  |
|  | Were the reasons for withdrawals and dropouts provided? |  | 0 |
| 5. Exposure/intervention | Was the type of food described (eg. Composition, matrix)? | 1 |  |
|  | Was the amount of food described (i.e. dose)? | 1 |  |
| 6. Health effect | Was the methodology used to measure the health effect reported? | 1 |  |
| 7. Statistical analysis | Was between group statistical analysis of the health effect reported? |  | 0 |
|  | Was an intention-to-treat analysis conducted? |  | 0 |
| 8. Potential confounders | Were potential confounders of the food health relationship considered? |  | 0 |
| TOTAL SCORE (maximum of 15): | | 5/15 |  |
| Higher quality (Score ≥ 8) | |  | |
| Lower quality (Score ≤ 7) | | X | |

Confounders: Baseline subject characteristics differed significantly – meaning results subject to residual confounding. Creation of control group involving sub-sample of each age/disease category not scientifically robust. No advice regarding physical activity provided to participants

| **Quality Appraisal Tool for Experimental Studies** | | | |
| --- | --- | --- | --- |
| **Study reference:** Coates et al (2020) | |  | |
| **Item** | **Question** | **Score** | |
|  |  | YES (1) | NO /NR(0) |
| 1. Inclusion/ Exclusion Criteria | Were the inclusion and/or exclusion criteria for study participation reported (*e.g.*, age greater than 50 years, no history of heart disease)? | 1 |  |
| 2. Group Allocation | Was the study described as randomized? | 1 |  |
|  | Was the randomization method reported? | 1 |  |
|  | Was the randomization method appropriate? | 1 |  |
|  | Was allocation concealed? | 1 |  |
| 3. Blinding | Were the study subjects blinded to the intervention received? |  | 0 (NR) |
|  | Were the research personnel blinded to the intervention received by the subjects? | 1 |  |
| 4. Attrition | Was attrition numerically reported? | 1 |  |
|  | Were the reasons for withdrawals and dropouts provided? | 1 |  |
| 5. Exposure/  Intervention | Was the type of food described (e.g., composition, matrix)? | 1 |  |
|  | Was the amount of food described (i.e., dose)? | 1 |  |
| 6. Health Effect | Was the methodology used to measure the health effect reported? | 1 |  |
| 7. Statistical Analysis | Was a between-group statistical analysis of the health effect conducted (*i.e.*, control vs. intervention)? | 1 |  |
|  | Was an intention-to-treat analysis conducted? |  | 0 |
| 8. Potential Confounders | Were potential confounders of the food/health relationship considered? | 1 |  |
| TOTAL SCORE (maximum of 15): | | 13/15 | |
| Higher quality (Score ≥ 8) | | X | |
| Lower quality (Score ≤ 7) | |  | |

Confounders: Physical activity collected

| **Quality Appraisal Tool for Experimental Studies** | | | |
| --- | --- | --- | --- |
| **Study reference:** Colquhoun et al. (1996) | | | |
| **Item** | **Question** | **Score** | |
|  |  | YES (1) | NO /NR(0) |
| 1. Inclusion/exclusion criteria | Were the inclusion and exclusion criteria for study participation reported? (eg. Age greater than 50 years, no history of heart disease)? | 1 |  |
| 2. Group allocation | Was the study described as randomized? | 1 |  |
|  | Was the randomization method reported? |  | 0 |
|  | Was the randomization appropriate? |  | 0 |
|  | Was the allocation concealed? |  | 0 |
| 3. Blinding | Were the study subjects blinded to the intervention received? |  | 0 |
|  | Were the researcher personnel blinded to the intervention received by the subjects? |  | 0 |
| 4. Attrition | Were attrition numerically reported? | 1 |  |
|  | Were the reasons for withdrawals and dropouts provided? | 1 |  |
| 5. Exposure/intervention | Was the type of food described (eg. Composition, matrix)? | 1 |  |
|  | Was the amount of food described (i.e. dose)? | 1 |  |
| 6. Health effect | Was the methodology used to measure the health effect reported? | 1 |  |
| 7. Statistical analysis | Was between group statistical analysis of the health effect reported? |  | 0 |
|  | Was an intention-to-treat analysis conducted? |  | 0 |
| 8. Potential confounders | Were potential confounders of the food health relationship considered? |  | 0 |
| TOTAL SCORE (maximum of 15): | | 7/15 |  |
| Higher quality (Score ≥ 8) | |  | |
| Lower quality (Score ≤ 7) | | X | |

Confounders: no controlling for PA, few exclusion criterion

| **Quality Appraisal Tool for Experimental Studies** | | | |
| --- | --- | --- | --- |
| **Study reference:** Costa e Silva et al. (2020) | |  | |
| **Item** | **Question** | **Score** | |
|  |  | YES (1) | NO /NR(0) |
| 1. Inclusion/ Exclusion Criteria | Were the inclusion and/or exclusion criteria for study participation reported (*e.g.*, age greater than 50 years, no history of heart disease)? | 1 |  |
| 2. Group Allocation | Was the study described as randomized? | 1 |  |
|  | Was the randomization method reported? |  | 0 (NR) |
|  | Was the randomization method appropriate? |  | 0 (NR) |
|  | Was allocation concealed? |  | 0 (NR) |
| 3. Blinding | Were the study subjects blinded to the intervention received? | 1 |  |
|  | Were the research personnel blinded to the intervention received by the subjects? | 1 |  |
| 4. Attrition | Was attrition numerically reported? | 1 |  |
|  | Were the reasons for withdrawals and dropouts provided? |  | 0 |
| 5. Exposure/  Intervention | Was the type of food described (e.g., composition, matrix)? | 1 |  |
|  | Was the amount of food described (i.e., dose)? | 1 |  |
| 6. Health Effect | Was the methodology used to measure the health effect reported? | 1 |  |
| 7. Statistical Analysis | Was a between-group statistical analysis of the health effect conducted (*i.e.*, control vs. intervention)? | 1 |  |
|  | Was an intention-to-treat analysis conducted? |  | 0 |
| 8. Potential Confounders | Were potential confounders of the food/health relationship considered? | 1 |  |
| TOTAL SCORE (maximum of 15): | | 10/15 | |
| Higher quality (Score ≥ 8) | | X | |
| Lower quality (Score ≤ 7) | |  | |

Confounders: The lifestyle habits of each subject were assessed through an interview and clinical consultation. Subjects were guided to maintain their eating habits and lifestyle, and to avoid food supplements.

| **Quality Appraisal Tool for Experimental Studies** | | | |
| --- | --- | --- | --- |
| **Study reference:** Curb et al. (2000) | | | |
| **Item** | **Question** | **Score** | |
|  |  | YES (1) | NO /NR(0) |
| 1. Inclusion/exclusion criteria | Were the inclusion and exclusion criteria for study participation reported? (eg. Age greater than 50 years, no history of heart disease)? | 1 |  |
| 2. Group allocation | Was the study described as randomized? | 1 |  |
|  | Was the randomization method reported? | 1 |  |
|  | Was the randomization appropriate? | 1 |  |
|  | Was the allocation concealed? | 1 |  |
| 3. Blinding | Were the study subjects blinded to the intervention received? |  | 0 |
|  | Were the researcher personnel blinded to the intervention received by the subjects? | 1 |  |
| 4. Attrition | Were attrition numerically reported? | 1 |  |
|  | Were the reasons for withdrawals and dropouts provided? |  | 0 |
| 5. Exposure/intervention | Was the type of food described (eg. Composition, matrix)? | 1 |  |
|  | Was the amount of food described (i.e. dose)? |  | 0 |
| 6. Health effect | Was the methodology used to measure the health effect reported? | 1 |  |
| 7. Statistical analysis | Was between group statistical analysis of the health effect reported? | 1 |  |
|  | Was an intention-to-treat analysis conducted? |  | 0 |
| 8. Potential confounders | Were potential confounders of the food health relationship considered? | 1 |  |
| TOTAL SCORE (maximum of 15): | | 11/15 |  |
| Higher quality (Score ≥ 8) | | X | |
| Lower quality (Score ≤ 7) | |  | |

Confounders: Both randomizations were stratified by sex. Assessed for effect of period and for potential carryover effects (none found)

| **Quality Appraisal Tool for Experimental Studies** | | | |
| --- | --- | --- | --- |
| **Study reference:** Damasceno et al. (2011) | | | |
| **Item** | **Question** | **Score** |  |
|  |  | YES (1) | NO /NR(0) |
| 1. Inclusion/exclusion criteria | Were the inclusion and exclusion criteria for study participation reported? (eg. Age greater than 50 years, no history of heart disease)? | 1 |  |
| 2. Group allocation | Was the study described as randomized? | 1 |  |
|  | Was the randomization method reported? | 1 |  |
|  | Was the randomization appropriate? | 1 |  |
|  | Was the allocation concealed? | 1 |  |
| 3. Blinding | Were the study subjects blinded to the intervention received? |  | 0 |
|  | Were the researcher personnel blinded to the intervention received by the subjects? | 1 |  |
| 4. Attrition | Were attrition numerically reported? | 1 |  |
|  | Were the reasons for withdrawals and dropouts provided? | 1 |  |
| 5. Exposure/intervention | Was the type of food described (eg. Composition, matrix)? | 1 |  |
|  | Was the amount of food described (i.e. dose)? | 1 |  |
| 6. Health effect | Was the methodology used to measure the health effect reported? | 1 |  |
| 7. Statistical analysis | Was between group statistical analysis of the health effect reported? |  | 0 |
|  | Was an intention-to-treat analysis conducted? |  | 0 |
| 8. Potential confounders | Were potential confounders of the food health relationship considered? | 1 |  |
| TOTAL SCORE (maximum of 15): | | 12/15 |  |
| Higher quality (Score ≥ 8) | | X | |
| Lower quality (Score ≤ 7) | |  | |

Confounders: Predictive model used which took into account differences in fat contents of the diet. Also assessed if treatment sequence or gender influenced results

| **Quality Appraisal Tool for Experimental Studies** | | | |
| --- | --- | --- | --- |
| **Study reference: Damavandi et al. (2013)** | | | |
| **Item** | **Question** | **Score** | |
|  |  | YES (1) | NO /NR(0) |
| 1. Inclusion/exclusion criteria | Were the inclusion and exclusion criteria for study participation reported? (eg. Age greater than 50 years, no history of heart disease)? | 1 |  |
| 2. Group allocation | Was the study described as randomized? | 1 |  |
|  | Was the randomization method reported? | 1 |  |
|  | Was the randomization appropriate? | 1 |  |
|  | Was the allocation concealed? |  | 0 NR |
| 3. Blinding | Were the study subjects blinded to the intervention received? |  | 0 |
|  | Were the researcher personnel blinded to the intervention received by the subjects? |  | 0 |
| 4. Attrition | Were attrition numerically reported? | 1 |  |
|  | Were the reasons for withdrawals and dropouts provided? | 1 |  |
| 5. Exposure/intervention | Was the type of food described (eg. Composition, matrix)? | 1 |  |
|  | Was the amount of food described (i.e. dose)? | 1 |  |
| 6. Health effect | Was the methodology used to measure the health effect reported? | 1 |  |
| 7. Statistical analysis | Was between group statistical analysis of the health effect reported? | 1 |  |
|  | Was an intention-to-treat analysis conducted? |  | 0 |
| 8. Potential confounders | Were potential confounders of the food health relationship considered? | 1 |  |
| TOTAL SCORE (maximum of 15): | | 11/15 |  |
| Higher quality (Score ≥ 8) | | X | |
| Lower quality (Score ≤ 7) | |  | |

Confounders: No significant differences between groups at baseline, no changes in physical activity found during the study. Analyses included fat and carbohydrate intake as covariates

| **Quality Appraisal Tool for Experimental Studies** | | | |
| --- | --- | --- | --- |
| **Study reference:** Damavandi et al. (2019) | |  | |
| **Item** | **Question** | **Score** | |
|  | YES (1) | YES (1) | NO /NR(0) |
| 1. Inclusion/ Exclusion Criteria | Were the inclusion and/or exclusion criteria for study participation reported (*e.g.*, age greater than 50 years, no history of heart disease)? | 1 |  |
| 2. Group Allocation | Was the study described as randomized? | 1 |  |
|  | Was the randomization method reported? |  | 0 (NR) |
|  | Was the randomization method appropriate? |  | 0 (NR) |
|  | Was allocation concealed? |  | 0 (NR) |
| 3. Blinding | Were the study subjects blinded to the intervention received? |  | 0 (NR) |
|  | Were the research personnel blinded to the intervention received by the subjects? |  | 0 (NR) |
| 4. Attrition | Was attrition numerically reported? | 1 |  |
|  | Were the reasons for withdrawals and dropouts provided? | 1 |  |
| 5. Exposure/  Intervention | Was the type of food described (e.g., composition, matrix)? | 1 |  |
|  | Was the amount of food described (i.e., dose)? | 1 |  |
| 6. Health Effect | Was the methodology used to measure the health effect reported? | 1 |  |
| 7. Statistical Analysis | Was a between-group statistical analysis of the health effect conducted (*i.e.*, control vs. intervention)? | 1 |  |
|  | Was an intention-to-treat analysis conducted? |  | 0 |
| 8. Potential Confounders | Were potential confounders of the food/health relationship considered? | 1 |  |
| TOTAL SCORE (maximum of 15): | | 9/15 | |
| Higher quality (Score ≥ 8) | | X | |
| Lower quality (Score ≤ 7) | |  | |

Confounders: changes to physical activity recorded, participants advised not to change activity

| **Quality Appraisal Tool for Experimental Studies** | | | |
| --- | --- | --- | --- |
| **Study reference:** de Souza et al. (2018) | |  | |
| **Item** | **Question** | **Score** | |
|  |  | Yes | No/NR |
| 1. Inclusion/ Exclusion Criteria | Were the inclusion and/or exclusion criteria for study participation reported (*e.g.*, age greater than 50 years, no history of heart disease)? | 1 |  |
| 2. Group Allocation | Was the study described as randomized? | 1 |  |
|  | Was the randomization method reported? | 1 |  |
|  | Was the randomization method appropriate? | 1 |  |
|  | Was allocation concealed? |  | 0 |
| 3. Blinding | Were the study subjects blinded to the intervention received? |  | 0 (NR) |
|  | Were the research personnel blinded to the intervention received by the subjects? |  | 0 (NR) |
| 4. Attrition | Was attrition numerically reported? | 1 |  |
|  | Were the reasons for withdrawals and dropouts provided? | 1 |  |
| 5. Exposure/  Intervention | Was the type of food described (e.g., composition, matrix)? | 1 |  |
|  | Was the amount of food described (i.e., dose)? | 1 |  |
| 6. Health Effect | Was the methodology used to measure the health effect reported? | 1 |  |
| 7. Statistical Analysis | Was a between-group statistical analysis of the health effect conducted (*i.e.*, control vs. intervention)? | 1 |  |
|  | Was an intention-to-treat analysis conducted? |  | 0 |
| 8. Potential Confounders | Were potential confounders of the food/health relationship considered? | 1 |  |
| TOTAL SCORE (maximum of 15): | | 11/15 | |
| Higher quality (Score ≥ 8) | | X | |
| Lower quality (Score ≤ 7) | |  | |

Confounders: participants advised to maintain usual physical activity

| **Quality Appraisal Tool for Experimental Studies** | | | |
| --- | --- | --- | --- |
| **Study reference:** Deon et al (2018) | | | |
| **Item** | **Question** | **Score** | |
|  |  | YES (1) | NO /NR(0) |
| 1. Inclusion/exclusion criteria | Were the inclusion and exclusion criteria for study participation reported? (eg. Age greater than 50 years, no history of heart disease)? | 1 |  |
| 2. Group allocation | Was the study described as randomized? | 1 |  |
|  | Was the randomization method reported? | 1 |  |
|  | Was the randomization appropriate? | 1 |  |
|  | Was the allocation concealed? | 1 |  |
| 3. Blinding | Were the study subjects blinded to the intervention received? | 1 |  |
|  | Were the researcher personnel blinded to the intervention received by the subjects? |  | 0 |
| 4. Attrition | Were attrition numerically reported? | 1 |  |
|  | Were the reasons for withdrawals and dropouts provided? | 1 |  |
| 5. Exposure/intervention | Was the type of food described (e.g.. Composition, matrix)? | 1 |  |
|  | Was the amount of food described (i.e. dose)? | 1 |  |
| 6. Health effect | Was the methodology used to measure the health effect reported? | 1 |  |
| 7. Statistical analysis | Was between group statistical analysis of the health effect reported? | 1 |  |
|  | Was an intention-to-treat analysis conducted? |  | 0 |
| 8. Potential confounders | Were potential confounders of the food health relationship considered? |  | 0 |
| TOTAL SCORE (maximum of 15): | | 12/15 |  |
| Higher quality (Score ≥ 8) | | X | |
| Lower quality (Score ≤ 7) | |  | |

Confounders: There was ~10% weight difference between control and HZN+S groups at baseline.

| **Quality Appraisal Tool for Experimental Studies** | | | |
| --- | --- | --- | --- |
| **Study reference:** Dhillon et al (2016) | | | |
| **Item** | **Question** | **Score** | |
|  |  | YES (1) | NO /NR(0) |
| 1. Inclusion/exclusion criteria | Were the inclusion and exclusion criteria for study participation reported? (eg. Age greater than 50 years, no history of heart disease)? | 1 |  |
| 2. Group allocation | Was the study described as randomized? | 1 |  |
|  | Was the randomization method reported? |  | 0 (NR) |
|  | Was the randomization appropriate? |  | 0 (NR) |
|  | Was the allocation concealed? |  | 0(NR) |
| 3. Blinding | Were the study subjects blinded to the intervention received? |  | 0(NR) |
|  | Were the researcher personnel blinded to the intervention received by the subjects? |  | 0(NR) |
| 4. Attrition | Were attrition numerically reported? | 1 |  |
|  | Were the reasons for withdrawals and dropouts provided? | 1 |  |
| 5. Exposure/intervention | Was the type of food described (e.g. Composition, matrix)? | 1 |  |
|  | Was the amount of food described (i.e. dose)? | 1 |  |
| 6. Health effect | Was the methodology used to measure the health effect reported? | 1 |  |
| 7. Statistical analysis | Was between group statistical analysis of the health effect reported? | 1 |  |
|  | Was an intention-to-treat analysis conducted? | 1 |  |
| 8. Potential confounders | Were potential confounders of the food health relationship considered? |  | 0 |
| TOTAL SCORE (maximum of 15): | | 9/15 |  |
| Higher quality (Score ≥ 8) | | X | |
| Lower quality (Score ≤ 7) | |  | |

Confounders: Imbalance of BMI between groups at baseline (29.9 ± 3.2 vs. 40 ± 4.5 kg/m^2^).

| **Quality Appraisal Tool for Experimental Studies** | | | |
| --- | --- | --- | --- |
| **Study reference:** Dhillon et al. (2018) | |  | |
| **Item** | **Question** | **Score** | |
|  |  | YES (1) | NO /NR(0) |
| 1. Inclusion/ Exclusion Criteria | Were the inclusion and/or exclusion criteria for study participation reported (*e.g.*, age greater than 50 years, no history of heart disease)? | 1 |  |
| 2. Group Allocation | Was the study described as randomized? | 1 |  |
|  | Was the randomization method reported? | 1 |  |
|  | Was the randomization method appropriate? | 1 |  |
|  | Was allocation concealed? | 1 |  |
| 3. Blinding | Were the study subjects blinded to the intervention received? |  | 0 (NR) |
|  | Were the research personnel blinded to the intervention received by the subjects? |  | 0 (NR) |
| 4. Attrition | Was attrition numerically reported? | 1 |  |
|  | Were the reasons for withdrawals and dropouts provided? | 1 |  |
| 5. Exposure/  Intervention | Was the type of food described (e.g., composition, matrix)? | 1 |  |
|  | Was the amount of food described (i.e., dose)? | 1 |  |
| 6. Health Effect | Was the methodology used to measure the health effect reported? | 1 |  |
| 7. Statistical Analysis | Was a between-group statistical analysis of the health effect conducted (*i.e.*, control vs. intervention)? | 1 |  |
|  | Was an intention-to-treat analysis conducted? |  | 0 |
| 8. Potential Confounders | Were potential confounders of the food/health relationship considered? | 1 |  |
| TOTAL SCORE (maximum of 15): | | 12/15 | |
| Higher quality (Score ≥ 8) | | X | |
| Lower quality (Score ≤ 7) | |  | |

Confounders: physical activity measured during study

| **Quality Appraisal Tool for Experimental Studies** | | | |
| --- | --- | --- | --- |
| **Study reference:** Dikariyanto et al (2020) | |  | |
| **Item** | **Question** | **Score** | |
|  |  | YES (1) | NO /NR(0) |
| 1. Inclusion/ Exclusion Criteria | Were the inclusion and/or exclusion criteria for study participation reported (*e.g.*, age greater than 50 years, no history of heart disease)? | 1 |  |
| 2. Group Allocation | Was the study described as randomized? | 1 |  |
|  | Was the randomization method reported? | 1 |  |
|  | Was the randomization method appropriate? | 1 |  |
|  | Was allocation concealed? |  | 0 (NR) |
| 3. Blinding | Were the study subjects blinded to the intervention received? |  | 0 (NR) |
|  | Were the research personnel blinded to the intervention received by the subjects? | 1 |  |
| 4. Attrition | Was attrition numerically reported? | 1 |  |
|  | Were the reasons for withdrawals and dropouts provided? | 1 |  |
| 5. Exposure/  Intervention | Was the type of food described (e.g., composition, matrix)? | 1 |  |
|  | Was the amount of food described (i.e., dose)? | 1 |  |
| 6. Health Effect | Was the methodology used to measure the health effect reported? | 1 |  |
| 7. Statistical Analysis | Was a between-group statistical analysis of the health effect conducted (*i.e.*, control vs. intervention)? | 1 |  |
|  | Was an intention-to-treat analysis conducted? |  | 0 |
| 8. Potential Confounders | Were potential confounders of the food/health relationship considered? | 1 |  |
| TOTAL SCORE (maximum of 15): | | 12/15 | |
| Higher quality (Score ≥ 8) | | X | |
| Lower quality (Score ≤ 7) | |  | |

Confounders: Physical activity collected

| **Quality Appraisal Tool for Experimental Studies** | | | |
| --- | --- | --- | --- |
| **Study reference:** Din et al. (2011) | | | |
| **Item** | **Question** | **Score** | |
|  |  | YES (1) | NO /NR(0) |
| 1. Inclusion/exclusion criteria | Were the inclusion and exclusion criteria for study participation reported? (eg. Age greater than 50 years, no history of heart disease)? | 1 |  |
| 2. Group allocation | Was the study described as randomized? | 1 |  |
|  | Was the randomization method reported? |  |  |
|  | Was the randomization appropriate? | 1 |  |
|  | Was the allocation concealed? |  | 0 NR |
| 3. Blinding | Were the study subjects blinded to the intervention received? |  | 0 |
|  | Were the researcher personnel blinded to the intervention received by the subjects? | 1 |  |
| 4. Attrition | Were attrition numerically reported? |  | 0 |
|  | Were the reasons for withdrawals and dropouts provided? |  | 0 |
| 5. Exposure/intervention | Was the type of food described (eg. Composition, matrix)? | 1 |  |
|  | Was the amount of food described (i.e. dose)? | 1 |  |
| 6. Health effect | Was the methodology used to measure the health effect reported? | 1 |  |
| 7. Statistical analysis | Was between group statistical analysis of the health effect reported? | 1 |  |
|  | Was an intention-to-treat analysis conducted? | 1 |  |
| 8. Potential confounders | Were potential confounders of the food health relationship considered? |  | 0 |
| TOTAL SCORE (maximum of 15): | | 9/15 |  |
| Higher quality (Score ≥ 8) | | X | |
| Lower quality (Score ≤ 7) | |  | |

Confounders: Dietary data collected, but results not discussed for total energy/other macronutrient

| **Quality Appraisal Tool for Experimental Studies** | | | |
| --- | --- | --- | --- |
| **Reference (Author, year):**   Dusanov et al (2019) | |  | |
| **Item** | **Question** | **Score** | |
|  |  | YES (1) | NO /NR(0) |
| 1. Inclusion/ Exclusion Criteria | Were the inclusion and/or exclusion criteria for study participation reported (*e.g.*, age greater than 50 years, no history of heart disease)? | 1 |  |
| 2. Group Allocation | Was the study described as randomized? | 1 |  |
|  | Was the randomization method reported? | 1 |  |
|  | Was the randomization method appropriate? | 1 |  |
|  | Was allocation concealed? | 1 |  |
| 3. Blinding | Were the study subjects blinded to the intervention received? |  | 0 (NR) |
|  | Were the research personnel blinded to the intervention received by the subjects? |  | 0 (NR) |
| 4. Attrition | Was attrition numerically reported? | 1 |  |
|  | Were the reasons for withdrawals and dropouts provided? | 1 |  |
| 5. Exposure/  Intervention | Was the type of food described (e.g., composition, matrix)? | 1 |  |
|  | Was the amount of food described (i.e., dose)? | 1 |  |
| 6. Health Effect | Was the methodology used to measure the health effect reported? | 1 |  |
| 7. Statistical Analysis | Was a between-group statistical analysis of the health effect conducted (*i.e.*, control vs. intervention)? | 1 |  |
|  | Was an intention-to-treat analysis conducted? | 1 |  |
| 8. Potential Confounders | Were potential confounders of the food/health relationship considered? |  | 0 (NR) |
| TOTAL SCORE (maximum of 15): | | 12/15 | |
| Higher quality (Score ≥ 8) | | X | |
| Lower quality (Score ≤ 7) | |  | |

Confounders: changes in lifestyle factors not reported

| **Quality Appraisal Tool for Experimental Studies** | | | |
| --- | --- | --- | --- |
| **Study reference:** Eastman & Clayshulte (2005) | | | |
| **Item** | **Question** | **Score** | |
|  |  | YES (1) | NO /NR(0) |
| 1. Inclusion/exclusion criteria | Were the inclusion and exclusion criteria for study participation reported? (eg. Age greater than 50 years, no history of heart disease)? | 1 |  |
| 2. Group allocation | Was the study described as randomized? | 1 |  |
|  | Was the randomization method reported? |  |  |
|  | Was the randomization appropriate? | 1 |  |
|  | Was the allocation concealed? |  | 0 |
| 3. Blinding | Were the study subjects blinded to the intervention received? |  | 0 |
|  | Were the researcher personnel blinded to the intervention received by the subjects? |  | 0 |
| 4. Attrition | Were attrition numerically reported? | 1 |  |
|  | Were the reasons for withdrawals and dropouts provided? | 1 |  |
| 5. Exposure/intervention | Was the type of food described (eg. Composition, matrix)? | 1 |  |
|  | Was the amount of food described (i.e. dose)? | 1 |  |
| 6. Health effect | Was the methodology used to measure the health effect reported? | 1 |  |
| 7. Statistical analysis | Was between group statistical analysis of the health effect reported? | 1 |  |
|  | Was an intention-to-treat analysis conducted? |  | 0 |
| 8. Potential confounders | Were potential confounders of the food health relationship considered? |  | 0 |
| TOTAL SCORE (maximum of 15): | | 9/15 |  |
| Higher quality (Score ≥ 8) | | X | |
| Lower quality (Score ≤ 7) | |  | |

Confounders: The two groups were not matched at baseline (the pecan group had non-significantly lower total-cholesterol, LDL cholesterol, and HDL-cholesterol levels than the control group and were younger). Also no information on physical activity – no advice provided to participants, may have introduced source of error

| **Quality Appraisal Tool for Experimental Studies** | | | |
| --- | --- | --- | --- |
| **Study reference:** Fatahi et al. (2019) | |  | |
| **Item** | **Question** | **Score** | |
|  |  | Yes | No/NR |
| 1. Inclusion/ Exclusion Criteria | Were the inclusion and/or exclusion criteria for study participation reported (*e.g.*, age greater than 50 years, no history of heart disease)? | 1 |  |
| 2. Group Allocation | Was the study described as randomized? | 1 |  |
|  | Was the randomization method reported? | 1 |  |
|  | Was the randomization method appropriate? | 1 |  |
|  | Was allocation concealed? |  | 0 (NR) |
| 3. Blinding | Were the study subjects blinded to the intervention received? |  | 0 |
|  | Were the research personnel blinded to the intervention received by the subjects? |  | 0 (NR) |
| 4. Attrition | Was attrition numerically reported? | 1 |  |
|  | Were the reasons for withdrawals and dropouts provided? | 1 |  |
| 5. Exposure/  Intervention | Was the type of food described (e.g., composition, matrix)? | 1 |  |
|  | Was the amount of food described (i.e., dose)? | 1 |  |
| 6. Health Effect | Was the methodology used to measure the health effect reported? | 1 |  |
| 7. Statistical Analysis | Was a between-group statistical analysis of the health effect conducted (*i.e.*, control vs. intervention)? | 1 |  |
|  | Was an intention-to-treat analysis conducted? | 1 |  |
| 8. Potential Confounders | Were potential confounders of the food/health relationship considered? | 1 |  |
| TOTAL SCORE (maximum of 15): | | 12/15 | |
| Higher quality (Score ≥ 8) | | X | |
| Lower quality (Score ≤ 7) | |  | |

Confounders: changes to physical activity recorded

| **Quality Appraisal Tool for Experimental Studies** | | | |
| --- | --- | --- | --- |
| **Study reference:** Foster et al. (2012) | | | |
| **Item** | **Question** | **Score** | |
|  |  | YES (1) | NO /NR(0) |
| 1. Inclusion/exclusion criteria | Were the inclusion and exclusion criteria for study participation reported? (eg. Age greater than 50 years, no history of heart disease)? | 1 |  |
| 2. Group allocation | Was the study described as randomized? | 1 |  |
|  | Was the randomization method reported? | 1 |  |
|  | Was the randomization appropriate? | 1 |  |
|  | Was the allocation concealed? | 0 |  |
| 3. Blinding | Were the study subjects blinded to the intervention received? |  | 0 |
|  | Were the researcher personnel blinded to the intervention received by the subjects? |  | O NR |
| 4. Attrition | Were attrition numerically reported? | 1 |  |
|  | Were the reasons for withdrawals and dropouts provided? | 1 |  |
| 5. Exposure/intervention | Was the type of food described (eg. Composition, matrix)? | 1 |  |
|  | Was the amount of food described (i.e. dose)? | 1 |  |
| 6. Health effect | Was the methodology used to measure the health effect reported? | 1 |  |
| 7. Statistical analysis | Was between group statistical analysis of the health effect reported? | 1 |  |
|  | Was an intention-to-treat analysis conducted? | 1 |  |
| 8. Potential confounders | Were potential confounders of the food health relationship considered? |  | 0 |
| TOTAL SCORE (maximum of 15): | | 11/15 |  |
| Higher quality (Score ≥ 8) | | X | |
| Lower quality (Score ≤ 7) | |  | |

Confounders: no diet data provided

| **Quality Appraisal Tool for Experimental Studies** | | | |
| --- | --- | --- | --- |
| **Study reference:** Ghanavati et al.(2021a)/Ghanavati et al. (2021b) | |  | |
| **Item** | **Question** | **Score** | |
|  |  | YES (1) | NO /NR(0) |
| 1. Inclusion/ Exclusion Criteria | Were the inclusion and/or exclusion criteria for study participation reported (*e.g.*, age greater than 50 years, no history of heart disease)? | 1 |  |
| 2. Group Allocation | Was the study described as randomized? | 1 |  |
|  | Was the randomization method reported? |  | 0 |
|  | Was the randomization method appropriate? |  | 0 |
|  | Was allocation concealed? |  | 0 |
| 3. Blinding | Were the study subjects blinded to the intervention received? |  | 0 |
|  | Were the research personnel blinded to the intervention received by the subjects? |  | 0 |
| 4. Attrition | Was attrition numerically reported? | 1 |  |
|  | Were the reasons for withdrawals and dropouts provided? | 1 |  |
| 5. Exposure/  Intervention | Was the type of food described (e.g., composition, matrix)? | 1 |  |
|  | Was the amount of food described (i.e., dose)? | 1 |  |
| 6. Health Effect | Was the methodology used to measure the health effect reported? | 1 |  |
| 7. Statistical Analysis | Was a between-group statistical analysis of the health effect conducted (*i.e.*, control vs. intervention)? | 1 |  |
|  | Was an intention-to-treat analysis conducted? |  | 0 |
| 8. Potential Confounders | Were potential confounders of the food/health relationship considered? | 1 |  |
| TOTAL SCORE (maximum of 15): | | 9/15 | |
| Higher quality (Score ≥ 8) | | X | |
| Lower quality (Score ≤ 7) | |  | |

Confounders: advised to maintain usual physical activity (assessed)

| **Quality Appraisal Tool for Experimental Studies** | | | |
| --- | --- | --- | --- |
| **Study reference:** Gozde et al. (2019) | |  | |
| **Item** | **Question** | **Score** | |
|  |  | YES (1) | NO /NR(0) |
| 1. Inclusion/ Exclusion Criteria | Were the inclusion and/or exclusion criteria for study participation reported (*e.g.*, age greater than 50 years, no history of heart disease)? | 1 |  |
| 2. Group Allocation | Was the study described as randomized? | 1 |  |
|  | Was the randomization method reported? |  | 0 (NR) |
|  | Was the randomization method appropriate? |  | 0 (NR) |
|  | Was allocation concealed? |  | 0 (NR) |
| 3. Blinding | Were the study subjects blinded to the intervention received? |  | 0 (NR) |
|  | Were the research personnel blinded to the intervention received by the subjects? |  | 0 (NR) |
| 4. Attrition | Was attrition numerically reported? | 1 |  |
|  | Were the reasons for withdrawals and dropouts provided? | 1 |  |
| 5. Exposure/  Intervention | Was the type of food described (e.g., composition, matrix)? | 1 |  |
|  | Was the amount of food described (i.e., dose)? | 1 |  |
| 6. Health Effect | Was the methodology used to measure the health effect reported? | 1 |  |
| 7. Statistical Analysis | Was a between-group statistical analysis of the health effect conducted (*i.e.*, control vs. intervention)? | 1 |  |
|  | Was an intention-to-treat analysis conducted? |  | 0 |
| 8. Potential Confounders | Were potential confounders of the food/health relationship considered? | 1 |  |
| TOTAL SCORE (maximum of 15): | | 9/15 | |
| Higher quality (Score ≥ 8) | | X | |
| Lower quality (Score ≤ 7) | |  | |

Confounders: changes to physical activity recorded

| **Quality Appraisal Tool for Experimental Studies** | | | |
| --- | --- | --- | --- |
| **Study reference:** Griel et al. (2008) | | | |
| **Item** | **Question** | **Score** | |
|  |  | YES (1) | NO /NR(0) |
| 1. Inclusion/exclusion criteria | Were the inclusion and exclusion criteria for study participation reported? (eg. Age greater than 50 years, no history of heart disease)? | 1 |  |
| 2. Group allocation | Was the study described as randomized? | 1 |  |
|  | Was the randomization method reported? |  | 0 |
|  | Was the randomization appropriate? |  | 0 |
|  | Was the allocation concealed? |  | 0 |
| 3. Blinding | Were the study subjects blinded to the intervention received? |  | 0 |
|  | Were the researcher personnel blinded to the intervention received by the subjects? |  | 0 |
| 4. Attrition | Were attrition numerically reported? | 1 |  |
|  | Were the reasons for withdrawals and dropouts provided? | 1 |  |
| 5. Exposure/intervention | Was the type of food described (eg. Composition, matrix)? | 1 |  |
|  | Was the amount of food described (i.e. dose)? | 1 |  |
| 6. Health effect | Was the methodology used to measure the health effect reported? | 1 |  |
| 7. Statistical analysis | Was between group statistical analysis of the health effect reported? | 1 |  |
|  | Was an intention-to-treat analysis conducted? | 1 |  |
| 8. Potential confounders | Were potential confounders of the food health relationship considered? | 1 |  |
| TOTAL SCORE (maximum of 15): | | 10/15 |  |
| Higher quality (Score ≥ 8) | | X | |
| Lower quality (Score ≤ 7) | |  | |

Confounders: Effect of diet, gender, order of diet presentation, period, and their interactions investigated

| **Quality Appraisal Tool for Experimental Studies** | | | |
| --- | --- | --- | --- |
| **Study reference:** Guarneiri et al.(2021) | |  | |
| **Item** | **Question** | **Score** | |
|  |  | YES (1) | NO /NR(0) |
| 1. Inclusion/ Exclusion Criteria | Were the inclusion and/or exclusion criteria for study participation reported (*e.g.*, age greater than 50 years, no history of heart disease)? | 1 |  |
| 2. Group Allocation | Was the study described as randomized? | 1 |  |
|  | Was the randomization method reported? | 1 |  |
|  | Was the randomization method appropriate? | 1 |  |
|  | Was allocation concealed? |  | 0 |
| 3. Blinding | Were the study subjects blinded to the intervention received? |  | 0 |
|  | Were the research personnel blinded to the intervention received by the subjects? |  | 0 |
| 4. Attrition | Was attrition numerically reported? | 1 |  |
|  | Were the reasons for withdrawals and dropouts provided? | 1 |  |
| 5. Exposure/  Intervention | Was the type of food described (e.g., composition, matrix)? | 1 |  |
|  | Was the amount of food described (i.e., dose)? | 1 |  |
| 6. Health Effect | Was the methodology used to measure the health effect reported? | 1 |  |
| 7. Statistical Analysis | Was a between-group statistical analysis of the health effect conducted (*i.e.*, control vs. intervention)? | 1 |  |
|  | Was an intention-to-treat analysis conducted? |  | 0 |
| 8. Potential Confounders | Were potential confounders of the food/health relationship considered? | 1 |  |
| TOTAL SCORE (maximum of 15): | | 10/15 | |
| Higher quality (Score ≥ 8) | | X | |
| Lower quality (Score ≤ 7) | |  | |

Confounders: participants advised to maintain normal lifestyle

| **Quality Appraisal Tool for Experimental Studies** | | | |
| --- | --- | --- | --- |
| **Study reference:** Gulati et al. (2014) | | | |
| **Item** | **Question** | **Score** | |
|  |  | YES (1) | NO /NR(0) |
| 1. Inclusion/exclusion criteria | Were the inclusion and exclusion criteria for study participation reported? (eg. Age greater than 50 years, no history of heart disease)? | 1 |  |
| 2. Group allocation | Was the study described as randomized? | 1 |  |
|  | Was the randomization method reported? |  | 0 (NR) |
|  | Was the randomization appropriate? |  | 0 (NR) |
|  | Was the allocation concealed? |  | 0 (NR) |
| 3. Blinding | Were the study subjects blinded to the intervention received? |  | 0 (NR) |
|  | Were the researcher personnel blinded to the intervention received by the subjects? |  | 0 (NR) |
| 4. Attrition | Were attrition numerically reported? | 1 |  |
|  | Were the reasons for withdrawals and dropouts provided? |  | 0 |
| 5. Exposure/intervention | Was the type of food described (eg. Composition, matrix)? | 1 |  |
|  | Was the amount of food described (i.e. dose)? | 1 |  |
| 6. Health effect | Was the methodology used to measure the health effect reported? | 1 |  |
| 7. Statistical analysis | Was between group statistical analysis of the health effect reported? | 1 |  |
|  | Was an intention-to-treat analysis conducted? | 1 |  |
| 8. Potential confounders | Were potential confounders of the food health relationship considered? | 1 |  |
| TOTAL SCORE (maximum of 15): | | 9/15 |  |
| Higher quality (Score ≥ 8) | | X | |
| Lower quality (Score ≤ 7) | |  | |

Confounder: Analyses were adjusted for sex and baseline value of outcome. No difference between baseline measures of obesity and metabolic parameters

| **Quality Appraisal Tool for Experimental Studies** | | | |
| --- | --- | --- | --- |
| **Study reference:** Hernández-Alonso et al (2014) | | | |
| **Item** | **Question** | **Score** | |
|  |  | YES (1) | NO /NR(0) |
| 1. Inclusion/exclusion criteria | Were the inclusion and exclusion criteria for study participation reported? (eg. Age greater than 50 years, no history of heart disease)? | 1 |  |
| 2. Group allocation | Was the study described as randomized? | 1 |  |
|  | Was the randomization method reported? | 1 |  |
|  | Was the randomization appropriate? | 1 |  |
|  | Was the allocation concealed? | 1 |  |
| 3. Blinding | Were the study subjects blinded to the intervention received? |  | 0(NR) |
|  | Were the researcher personnel blinded to the intervention received by the subjects? |  | 0(NR) |
| 4. Attrition | Were attrition numerically reported? | 1 |  |
|  | Were the reasons for withdrawals and dropouts provided? | 1 |  |
| 5. Exposure/intervention | Was the type of food described (e.g.. Composition, matrix)? | 1 |  |
|  | Was the amount of food described (i.e. dose)? | 1 |  |
| 6. Health effect | Was the methodology used to measure the health effect reported? | 1 |  |
| 7. Statistical analysis | Was between group statistical analysis of the health effect reported? | 1 |  |
|  | Was an intention-to-treat analysis conducted? | 1 |  |
| 8. Potential confounders | Were potential confounders of the food health relationship considered? | 1 |  |
| TOTAL SCORE (maximum of 15): | | 13/15 |  |
| Higher quality (Score ≥ 8) | | X | |
| Lower quality (Score ≤ 7) | |  | |

Confounder: The adherence of dietary intervention was assessed using physical checking the package and biomarkers. Physical activity during the study was also assessed.

| **Quality Appraisal Tool for Experimental Studies** | | | |
| --- | --- | --- | --- |
| **Study reference:** Hiraoka-Yamamoto et al. (2004) | | | |
| **Item** | **Question** | **Score** | |
|  |  | YES (1) | NO /NR(0) |
| 1. Inclusion/exclusion criteria | Were the inclusion and exclusion criteria for study participation reported? (eg. Age greater than 50 years, no history of heart disease)? | 1 |  |
| 2. Group allocation | Was the study described as randomized? | 1 |  |
|  | Was the randomization method reported? |  | 0 |
|  | Was the randomization appropriate? |  | 0 (NR) |
|  | Was the allocation concealed? |  | 0 (NR) |
| 3. Blinding | Were the study subjects blinded to the intervention received? |  | 0 (NR) |
|  | Were the researcher personnel blinded to the intervention received by the subjects? |  | 0 (NR) |
| 4. Attrition | Were attrition numerically reported? |  | 0 (NR) |
|  | Were the reasons for withdrawals and dropouts provided? |  | 0 (NR) |
| 5. Exposure/intervention | Was the type of food described (eg. Composition, matrix)? | 1 |  |
|  | Was the amount of food described (i.e. dose)? | 1 |  |
| 6. Health effect | Was the methodology used to measure the health effect reported? | 1 |  |
| 7. Statistical analysis | Was between group statistical analysis of the health effect reported? |  | 0 |
|  | Was an intention-to-treat analysis conducted? |  | 0 (NR) |
| 8. Potential confounders | Were potential confounders of the food health relationship considered? |  | 0 |
| TOTAL SCORE (maximum of 15): | | 4/15 |  |
| Higher quality (Score ≥ 8) | |  | |
| Lower quality (Score ≤ 7) | | X | |

Confounders: No confounders included in analysis, and background diet was not measured. There were no significant differences in outcomes between groups at baseline, however due to lack of consideration of background diet this study was deemed to not have considered confounders

| **Quality Appraisal Tool for Experimental Studies** | | | |
| --- | --- | --- | --- |
| **Study reference:** Holligan et al (2014)/West et al. (2012)/Gebauer et al. (2008) | | | |
| **Item** | **Question** | **Score** | |
|  |  | YES (1) | NO /NR(0) |
| 1. Inclusion/exclusion criteria | Were the inclusion and exclusion criteria for study participation reported? (eg. Age greater than 50 years, no history of heart disease)? | 1 |  |
| 2. Group allocation | Was the study described as randomized? | 1 |  |
|  | Was the randomization method reported? | 1 |  |
|  | Was the randomization appropriate? | 1 |  |
|  | Was the allocation concealed? |  | 0 NR |
| 3. Blinding | Were the study subjects blinded to the intervention received? |  | 0 NO |
|  | Were the researcher personnel blinded to the intervention received by the subjects? |  | 0 NR |
| 4. Attrition | Were attrition numerically reported? | 1 |  |
|  | Were the reasons for withdrawals and dropouts provided? | 1 |  |
| 5. Exposure/intervention | Was the type of food described (eg. Composition, matrix)? | 1 |  |
|  | Was the amount of food described (i.e. dose)? | 1 |  |
| 6. Health effect | Was the methodology used to measure the health effect reported? | 1 |  |
| 7. Statistical analysis | Was between group statistical analysis of the health effect reported? | 1 |  |
|  | Was an intention-to-treat analysis conducted? |  | 0 |
| 8. Potential confounders | Were potential confounders of the food health relationship considered? | 1 |  |
| TOTAL SCORE (maximum of 15): | | 11/15 |  |
| Higher quality (Score ≥ 8) | | X | |
| Lower quality (Score ≤ 7) | |  | |

Confounders considered in analysis: treatment period. Dietary intake also investigated, no difference in energy intake between diets

| **Quality Appraisal Tool for Experimental Studies** | | | |
| --- | --- | --- | --- |
| **Study reference:** Holscher et al (2018) | |  | |
| **Item** | **Question** | **Score** | |
|  |  | YES (1) | NO /NR(0) |
| 1. Inclusion/ Exclusion Criteria | Were the inclusion and/or exclusion criteria for study participation reported (*e.g.*, age greater than 50 years, no history of heart disease)? | 1 |  |
| 2. Group Allocation | Was the study described as randomized? | 1 |  |
|  | Was the randomization method reported? | 1 |  |
|  | Was the randomization method appropriate? | 1 |  |
|  | Was allocation concealed? |  | 0 (NR) |
| 3. Blinding | Were the study subjects blinded to the intervention received? |  | 0 (NR) |
|  | Were the research personnel blinded to the intervention received by the subjects? |  | 0 (NR) |
| 4. Attrition | Was attrition numerically reported? | 1 |  |
|  | Were the reasons for withdrawals and dropouts provided? | 1 |  |
| 5. Exposure/  Intervention | Was the type of food described (e.g., composition, matrix)? | 1 |  |
|  | Was the amount of food described (i.e., dose)? | 1 |  |
| 6. Health Effect | Was the methodology used to measure the health effect reported? | 1 |  |
| 7. Statistical Analysis | Was a between-group statistical analysis of the health effect conducted (*i.e.*, control vs. intervention)? | 1 |  |
|  | Was an intention-to-treat analysis conducted? | 1 |  |
| 8. Potential Confounders | Were potential confounders of the food/health relationship considered? | 1 |  |
| TOTAL SCORE (maximum of 15): | | 12/15 | |
| Higher quality (Score ≥ 8) | | X | |
| Lower quality (Score ≤ 7) | |  | |

Confounders: Participants completed daily questionnaires for dietary deviations, medications, unusual exercise

| **Quality Appraisal Tool for Experimental Studies** | | | |
| --- | --- | --- | --- |
| **Study reference:** Holt et al (2015) | | | |
| **Item** | **Question** | **Score** | |
|  |  | YES (1) | NO /NR(0) |
| 1. Inclusion/exclusion criteria | Were the inclusion and exclusion criteria for study participation reported? (eg. Age greater than 50 years, no history of heart disease)? | 1 |  |
| 2. Group allocation | Was the study described as randomized? | 1 |  |
|  | Was the randomization method reported? | 1 |  |
|  | Was the randomization appropriate? | 1 |  |
|  | Was the allocation concealed? | 1 |  |
| 3. Blinding | Were the study subjects blinded to the intervention received? |  | 0 |
|  | Were the researcher personnel blinded to the intervention received by the subjects? |  | 0 |
| 4. Attrition | Were attrition numerically reported? | 1 |  |
|  | Were the reasons for withdrawals and dropouts provided? | 1 |  |
| 5. Exposure/intervention | Was the type of food described (e.g.. Composition, matrix)? | 1 |  |
|  | Was the amount of food described (i.e. dose)? | 1 |  |
| 6. Health effect | Was the methodology used to measure the health effect reported? | 1 |  |
| 7. Statistical analysis | Was between group statistical analysis of the health effect reported? | 1 |  |
|  | Was an intention-to-treat analysis conducted? |  | 0 |
| 8. Potential confounders | Were potential confounders of the food health relationship considered? |  | 0 |
| TOTAL SCORE (maximum of 15): | | 11/15 |  |
| Higher quality (Score ≥ 8) | | X | |
| Lower quality (Score ≤ 7) | |  | |

Confounders: There was a difference on triglycerides at baseline between groups (2.04±2.97 vs. 0.94±0.46 mmol/L)

| **Quality Appraisal Tool for Experimental Studies** | | | |
| --- | --- | --- | --- |
| **Study reference:** Huguenin et al (2015) | | | |
| **Item** | **Question** | **Score** | |
|  |  | YES (1) | NO /NR(0) |
| 1. Inclusion/exclusion criteria | Were the inclusion and exclusion criteria for study participation reported? (eg. Age greater than 50 years, no history of heart disease)? | 1 |  |
| 2. Group allocation | Was the study described as randomized? | 1 |  |
|  | Was the randomization method reported? | 1 |  |
|  | Was the randomization appropriate? | 1 |  |
|  | Was the allocation concealed? | 1 |  |
| 3. Blinding | Were the study subjects blinded to the intervention received? | 1 |  |
|  | Were the researcher personnel blinded to the intervention received by the subjects? | 1 |  |
| 4. Attrition | Were attrition numerically reported? | 1 |  |
|  | Were the reasons for withdrawals and dropouts provided? | 1 |  |
| 5. Exposure/intervention | Was the type of food described (e.g.. Composition, matrix)? | 1 |  |
|  | Was the amount of food described (i.e. dose)? | 1 |  |
| 6. Health effect | Was the methodology used to measure the health effect reported? | 1 |  |
| 7. Statistical analysis | Was between group statistical analysis of the health effect reported? | 1 |  |
|  | Was an intention-to-treat analysis conducted? |  | 0 |
| 8. Potential confounders | Were potential confounders of the food health relationship considered? |  | 0 |
| TOTAL SCORE (maximum of 15): | | 13/15 |  |
| Higher quality (Score ≥ 8) | | X | |
| Lower quality (Score ≤ 7) | |  | |

Confounders: There was no food provided to control group, the real double-blind might be unable to achieve.

| **Quality Appraisal Tool for Experimental Studies** | | | |
| --- | --- | --- | --- |
| **Study reference:** Hwang et al (2019) | |  | |
| **Item** | **Question** | **Score** | |
|  |  | YES (1) | NO /NR(0) |
| 1. Inclusion/ Exclusion Criteria | Were the inclusion and/or exclusion criteria for study participation reported (*e.g.*, age greater than 50 years, no history of heart disease)? | 1 |  |
| 2. Group Allocation | Was the study described as randomized? | 1 |  |
|  | Was the randomization method reported? | 1 |  |
|  | Was the randomization method appropriate? | 1 |  |
|  | Was allocation concealed? |  | 0 (NR) |
| 3. Blinding | Were the study subjects blinded to the intervention received? |  | 0 (NR) |
|  | Were the research personnel blinded to the intervention received by the subjects? |  | 0 (NR) |
| 4. Attrition | Was attrition numerically reported? | 1 |  |
|  | Were the reasons for withdrawals and dropouts provided? | 1 |  |
| 5. Exposure/  Intervention | Was the type of food described (e.g., composition, matrix)? | 1 |  |
|  | Was the amount of food described (i.e., dose)? | 1 |  |
| 6. Health Effect | Was the methodology used to measure the health effect reported? | 1 |  |
| 7. Statistical Analysis | Was a between-group statistical analysis of the health effect conducted (*i.e.*, control vs. intervention)? | 1 |  |
|  | Was an intention-to-treat analysis conducted? |  | 0 |
| 8. Potential Confounders | Were potential confounders of the food/health relationship considered? | 1 |  |
| TOTAL SCORE (maximum of 15): | | 11/15 | |
| Higher quality (Score ≥ 8) | | X | |
| Lower quality (Score ≤ 7) | |  | |

Confounders: not applicable as randomised cross-over design

| **Quality Appraisal Tool for Experimental Studies** | | | |
| --- | --- | --- | --- |
| **Study reference:** Iwamoto et al. (2002) | | | |
| **Item** | **Question** | **Score** | |
|  |  | YES (1) | NO /NR(0) |
| 1. Inclusion/exclusion criteria | Were the inclusion and exclusion criteria for study participation reported? (eg. Age greater than 50 years, no history of heart disease)? | 1 |  |
| 2. Group allocation | Was the study described as randomized? | 1 |  |
|  | Was the randomization method reported? |  | 0 |
|  | Was the randomization appropriate? |  | 0 (NR) |
|  | Was the allocation concealed? |  | 0 (NR) |
| 3. Blinding | Were the study subjects blinded to the intervention received? | 1 |  |
|  | Were the researcher personnel blinded to the intervention received by the subjects? |  | 0 |
| 4. Attrition | Were attrition numerically reported? | 1 |  |
|  | Were the reasons for withdrawals and dropouts provided? | 1 |  |
| 5. Exposure/intervention | Was the type of food described (eg. Composition, matrix)? | 1 |  |
|  | Was the amount of food described (i.e. dose)? | 1 |  |
| 6. Health effect | Was the methodology used to measure the health effect reported? | 1 |  |
| 7. Statistical analysis | Was between group statistical analysis of the health effect reported? | 1 |  |
|  | Was an intention-to-treat analysis conducted? | 1 |  |
| 8. Potential confounders | Were potential confounders of the food health relationship considered? | 1 |  |
| TOTAL SCORE (maximum of 15): | | 11/15 |  |
| Higher quality (Score ≥ 8) | | X | |
| Lower quality (Score ≤ 7) | |  | |

Confounders: Nutrient composition of diets was similar in both periods. Assessed for evidence of a carry-over effect (none found)

| **Quality Appraisal Tool for Experimental Studies** | | | |
| --- | --- | --- | --- |
| **Study reference:** Jaceldo-Siegl et al. (2011)/ Fraser et al. (2002) | | | |
| **Item** | **Question** | **Score** | |
|  |  | YES (1) | NO /NR(0) |
| 1. Inclusion/exclusion criteria | Were the inclusion and exclusion criteria for study participation reported? (eg. Age greater than 50 years, no history of heart disease)? | 1 |  |
| 2. Group allocation | Was the study described as randomized? |  | 0^1^ |
|  | Was the randomization method reported? |  | 0 (NR) |
|  | Was the randomization appropriate? |  | 0 (NR) |
|  | Was the allocation concealed? |  | 0 |
| 3. Blinding | Were the study subjects blinded to the intervention received? |  | 0 |
|  | Were the researcher personnel blinded to the intervention received by the subjects? |  | 0 |
| 4. Attrition | Were attrition numerically reported? | 1 |  |
|  | Were the reasons for withdrawals and dropouts provided? | 1 |  |
| 5. Exposure/intervention | Was the type of food described (eg. Composition, matrix)? | 1 |  |
|  | Was the amount of food described (i.e. dose)? | 1 |  |
| 6. Health effect | Was the methodology used to measure the health effect reported? | 1 |  |
| 7. Statistical analysis | Was between group statistical analysis of the health effect reported? | 1 |  |
|  | Was an intention-to-treat analysis conducted? |  | 0 |
| 8. Potential confounders | Were potential confounders of the food health relationship considered? |  | 0 |
| TOTAL SCORE (maximum of 15): | | 7/15 |  |
| Higher quality (Score ≥ 8) | |  | |
| Lower quality (Score ≤ 7) | | X | |

^1^ Not randomised to receive study treatment but randomised for start date to avoid effect of season on weight gain

Confounders: Viewed as not being considered due to potential effect of allocation order (not discussed in paper, methods states analyses adjusted for ‘other factors’ but no additional details given). Jaceldo-Siegl et al. (2011) adjusted for age, sex and change in body weight, however due to effect of diet order this study is deemed to be lower quality in this area.

| **Quality Appraisal Tool for Experimental Studies** | | | |
| --- | --- | --- | --- |
| **Study reference:** Jamshed et al (2015) | | | |
| **Item** | **Question** | **Score** | |
|  |  | YES (1) | NO /NR(0) |
| 1. Inclusion/exclusion criteria | Were the inclusion and exclusion criteria for study participation reported? (eg. Age greater than 50 years, no history of heart disease)? | 1 |  |
| 2. Group allocation | Was the study described as randomized? | 1 |  |
|  | Was the randomization method reported? | 1 |  |
|  | Was the randomization appropriate? | 1 |  |
|  | Was the allocation concealed? | 1 |  |
| 3. Blinding | Were the study subjects blinded to the intervention received? |  | 0(NR) |
|  | Were the researcher personnel blinded to the intervention received by the subjects? |  | 0(NR) |
| 4. Attrition | Were attrition numerically reported? | 1 |  |
|  | Were the reasons for withdrawals and dropouts provided? | 1 |  |
| 5. Exposure/intervention | Was the type of food described (e.g.. Composition, matrix)? | 1 |  |
|  | Was the amount of food described (i.e. dose)? | 1 |  |
| 6. Health effect | Was the methodology used to measure the health effect reported? | 1 |  |
| 7. Statistical analysis | Was between group statistical analysis of the health effect reported? | 1 |  |
|  | Was an intention-to-treat analysis conducted? |  | 0 |
| 8. Potential confounders | Were potential confounders of the food health relationship considered? | 1 |  |
| TOTAL SCORE (maximum of 15): | | 12/15 |  |
| Higher quality (Score ≥ 8) | | X | |
| Lower quality (Score ≤ 7) | |  | |

Confounders: Physical activity and dietary intake during the study were assessed.

| **Quality Appraisal Tool for Experimental Studies** | | | |
| --- | --- | --- | --- |
| **Study reference:** Jenkins et al. (2002) | | | |
| **Item** | **Question** | **Score** | |
|  |  | YES (1) | NO /NR(0) |
| 1. Inclusion/exclusion criteria | Were the inclusion and exclusion criteria for study participation reported? (eg. Age greater than 50 years, no history of heart disease)? | 1 |  |
| 2. Group allocation | Was the study described as randomized? | 1 |  |
|  | Was the randomization method reported? |  | 0 |
|  | Was the randomization appropriate? |  | 0 (NR) |
|  | Was the allocation concealed? |  | 0 (NR) |
| 3. Blinding | Were the study subjects blinded to the intervention received? |  | 0 (NR) |
|  | Were the researcher personnel blinded to the intervention received by the subjects? |  | 0 (NR) |
| 4. Attrition | Were attrition numerically reported? | 1 |  |
|  | Were the reasons for withdrawals and dropouts provided? | 1 |  |
| 5. Exposure/intervention | Was the type of food described (eg. Composition, matrix)? | 1 |  |
|  | Was the amount of food described (i.e. dose)? | 1 |  |
| 6. Health effect | Was the methodology used to measure the health effect reported? | 1 |  |
| 7. Statistical analysis | Was between group statistical analysis of the health effect reported? | 1 |  |
|  | Was an intention-to-treat analysis conducted? |  | 0 |
| 8. Potential confounders | Were potential confounders of the food health relationship considered? |  | 0 |
| TOTAL SCORE (maximum of 15): | | 8/15 |  |
| Higher quality (Score ≥ 8) | | X | |
| Lower quality (Score ≤ 7) | |  | |

Confounders: Noted analysis included diet, interaction term of diet by sex and sequence, individual, and baseline values, however study collected dietary data but did not report, therefore not clear if changes due to background diet or almonds

| **Quality Appraisal Tool for Experimental Studies** | | | |
| --- | --- | --- | --- |
| **Study reference:** Jenkins et al. (2018) | | | |
| **Item** | **Question** | **Score** | |
|  |  | YES (1) | NO /NR(0) |
| 1. Inclusion/exclusion criteria | Were the inclusion and exclusion criteria for study participation reported? (eg. Age greater than 50 years, no history of heart disease)? | 1 |  |
| 2. Group allocation | Was the study described as randomized? | 1 |  |
|  | Was the randomization method reported? |  | 0 |
|  | Was the randomization appropriate? |  | 0 (NR) |
|  | Was the allocation concealed? |  | 0 (NR) |
| 3. Blinding | Were the study subjects blinded to the intervention received? |  | 0 |
|  | Were the researcher personnel blinded to the intervention received by the subjects? |  | 0 |
| 4. Attrition | Were attrition numerically reported? | 1 |  |
|  | Were the reasons for withdrawals and dropouts provided? | 1 |  |
| 5. Exposure/intervention | Was the type of food described (eg. Composition, matrix)? | 1 |  |
|  | Was the amount of food described (i.e. dose)? | 1 |  |
| 6. Health effect | Was the methodology used to measure the health effect reported? | 1 |  |
| 7. Statistical analysis | Was between group statistical analysis of the health effect reported? | 1 |  |
|  | Was an intention-to-treat analysis conducted? | 1 |  |
| 8. Potential confounders | Were potential confounders of the food health relationship considered?^1^ | 1 |  |
| TOTAL SCORE (maximum of 15): | | 10/15 |  |
| Higher quality (Score ≥ 8) | | X | |
| Lower quality (Score ≤ 7) | |  | |

Confounders: Randomisation was stratified by sex and HbA1c. Statistical model used baseline values as a covariate. No difference between groups in baseline measures, with the exception of cholesterol-lowering medication (significantly lower percentage of participants in full-dose nut group, compared to half-dose). No difference between groups in energy intake

| **Quality Appraisal Tool for Experimental Studies** | | | |
| --- | --- | --- | --- |
| **Study reference:** Jia et al. (2006) | | | |
| **Item** | **Question** | **Score** | |
|  |  | YES (1) | NO /NR(0) |
| 1. Inclusion/exclusion criteria | Were the inclusion and exclusion criteria for study participation reported? (eg. Age greater than 50 years, no history of heart disease)? | 1 |  |
| 2. Group allocation | Was the study described as randomized? | 1 |  |
|  | Was the randomization method reported? |  | 0 |
|  | Was the randomization appropriate? |  | 0 |
|  | Was the allocation concealed? |  | 0 |
| 3. Blinding | Were the study subjects blinded to the intervention received? |  | 0 |
|  | Were the researcher personnel blinded to the intervention received by the subjects? |  | 0 |
| 4. Attrition | Were attrition numerically reported? |  | 0 NR |
|  | Were the reasons for withdrawals and dropouts provided? |  | 0 NR |
| 5. Exposure/intervention | Was the type of food described (eg. Composition, matrix)? | 1 |  |
|  | Was the amount of food described (i.e. dose)? | 1 |  |
| 6. Health effect | Was the methodology used to measure the health effect reported? | 1 |  |
| 7. Statistical analysis | Was between group statistical analysis of the health effect reported? | 1 |  |
|  | Was an intention-to-treat analysis conducted? |  | 0 |
| 8. Potential confounders | Were potential confounders of the food health relationship considered? |  | 0 |
| TOTAL SCORE (maximum of 15): | | 6/15 |  |
| Higher quality (Score ≥ 8) | |  | |
| Lower quality (Score ≤ 7) | | X | |

Confounders: Nutrition analysis not reported (although all volunteers eating the same diet from the same canteen). Although subjects recorded lapses of compliance with the protocol in addition to any signs of illness, medications taken, alcohol, and foods eaten other than those provided – no data on this is provided. No physical activity analysis provided

| **Quality Appraisal Tool for Experimental Studies** | | | |
| --- | --- | --- | --- |
| **Study reference:** Johnston et al, (2017) | | | |
| **Item** | **Question** | **Score** | |
|  |  | YES (1) | NO /NR(0) |
| 1. Inclusion/exclusion criteria | Were the inclusion and exclusion criteria for study participation reported? (eg. Age greater than 50 years, no history of heart disease)? | 1 |  |
| 2. Group allocation | Was the study described as randomized? | 1 |  |
|  | Was the randomization method reported? | 1 |  |
|  | Was the randomization appropriate? | 1 |  |
|  | Was the allocation concealed? |  | 0(NR) |
| 3. Blinding | Were the study subjects blinded to the intervention received? |  | 0(NR) |
|  | Were the researcher personnel blinded to the intervention received by the subjects? |  | 0(NR) |
| 4. Attrition | Were attrition numerically reported? | 1 |  |
|  | Were the reasons for withdrawals and dropouts provided? | 1 |  |
| 5. Exposure/intervention | Was the type of food described (e.g.. Composition, matrix)? | 1 |  |
|  | Was the amount of food described (i.e. dose)? | 1 |  |
| 6. Health effect | Was the methodology used to measure the health effect reported? | 1 |  |
| 7. Statistical analysis | Was between group statistical analysis of the health effect reported? | 1 |  |
|  | Was an intention-to-treat analysis conducted? |  | 0 |
| 8. Potential confounders | Were potential confounders of the food health relationship considered? |  | 0 |
| TOTAL SCORE (maximum of 15): | | 10/15 |  |
| Higher quality (Score ≥ 8) | | X | |
| Lower quality (Score ≤ 7) | |  | |

Confounders: The sample size was small (n=12)

| **Quality Appraisal Tool for Experimental Studies** | | | |
| --- | --- | --- | --- |
| **Study reference:** Jung et al. (2017) | | | |
| **Item** | **Question** | **Score** | |
|  |  | YES (1) | NO /NR(0) |
| 1. Inclusion/exclusion criteria | Were the inclusion and exclusion criteria for study participation reported? (eg. Age greater than 50 years, no history of heart disease)? | 1 |  |
| 2. Group allocation | Was the study described as randomized? | 1 |  |
|  | Was the randomization method reported? | 1 |  |
|  | Was the randomization appropriate? | 1 |  |
|  | Was the allocation concealed? |  | 0(NR) |
| 3. Blinding | Were the study subjects blinded to the intervention received? |  | 0(NR) |
|  | Were the researcher personnel blinded to the intervention received by the subjects? |  | 0(NR) |
| 4. Attrition | Were attrition numerically reported? | 1 |  |
|  | Were the reasons for withdrawals and dropouts provided? | 1 |  |
| 5. Exposure/intervention | Was the type of food described (e.g.. Composition, matrix)? | 1 |  |
|  | Was the amount of food described (i.e. dose)? | 1 |  |
| 6. Health effect | Was the methodology used to measure the health effect reported? | 1 |  |
| 7. Statistical analysis | Was between group statistical analysis of the health effect reported? | 1 |  |
|  | Was an intention-to-treat analysis conducted? |  | 0 |
| 8. Potential confounders | Were potential confounders of the food health relationship considered? |  | 0 |
| TOTAL SCORE (maximum of 15): | | 10/15 |  |
| Higher quality (Score ≥ 8) | | X | |
| Lower quality (Score ≤ 7) | |  | |

Confounders: Saturated fat intake in the control period increased from 6.3g (3.4% of energy intake) to 16.1g (8.2% of energy intake)

| **Quality Appraisal Tool for Experimental Studies** | | | |
| --- | --- | --- | --- |
| **Study reference:** Kamoun et al. (2021) | |  | |
| **Item** | **Question** | **Score** | |
|  |  | YES (1) | NO /NR(0) |
| 1. Inclusion/ Exclusion Criteria | Were the inclusion and/or exclusion criteria for study participation reported (*e.g.*, age greater than 50 years, no history of heart disease)? | 1 |  |
| 2. Group Allocation | Was the study described as randomized? | 1 |  |
|  | Was the randomization method reported? |  | 0 (NR) |
|  | Was the randomization method appropriate? |  | 0 (NR) |
|  | Was allocation concealed? |  | 0 (NR) |
| 3. Blinding | Were the study subjects blinded to the intervention received? |  | 0 (NR) |
|  | Were the research personnel blinded to the intervention received by the subjects? |  | 0 (NR) |
| 4. Attrition | Was attrition numerically reported? | 1 |  |
|  | Were the reasons for withdrawals and dropouts provided? | 1 |  |
| 5. Exposure/  Intervention | Was the type of food described (e.g., composition, matrix)? | 1 |  |
|  | Was the amount of food described (i.e., dose)? | 1 |  |
| 6. Health Effect | Was the methodology used to measure the health effect reported? | 1 |  |
| 7. Statistical Analysis | Was a between-group statistical analysis of the health effect conducted (*i.e.*, control vs. intervention)? | 1 |  |
|  | Was an intention-to-treat analysis conducted? |  | 0 |
| 8. Potential Confounders | Were potential confounders of the food/health relationship considered? |  | 0 |
| TOTAL SCORE (maximum of 15): | | 8/15 | |
| Higher quality (Score ≥ 8) | | X | |
| Lower quality (Score ≤ 7) | |  | |

| **Quality Appraisal Tool for Experimental Studies** | | | |
| --- | --- | --- | --- |
| **Study reference: Kasliwal et al (2015)** | | | |
| **Item** | **Question** | **Score** |  |
|  |  | YES (1) | NO /NR(0) |
| 1. Inclusion/exclusion criteria | Were the inclusion and exclusion criteria for study participation reported? (eg. Age greater than 50 years, no history of heart disease)? | 1 |  |
| 2. Group allocation | Was the study described as randomized? | 1 |  |
|  | Was the randomization method reported? |  | 0(NR) |
|  | Was the randomization appropriate? |  | 0(NR) |
|  | Was the allocation concealed? |  | 0(NR) |
| 3. Blinding | Were the study subjects blinded to the intervention received? |  | 0 |
|  | Were the researcher personnel blinded to the intervention received by the subjects? |  | 0 |
| 4. Attrition | Were attrition numerically reported? | 1 |  |
|  | Were the reasons for withdrawals and dropouts provided? | 1 |  |
| 5. Exposure/intervention | Was the type of food described (e.g.. Composition, matrix)? | 1 |  |
|  | Was the amount of food described (i.e. dose)? | 1 |  |
| 6. Health effect | Was the methodology used to measure the health effect reported? | 1 |  |
| 7. Statistical analysis | Was between group statistical analysis of the health effect reported? | 1 |  |
|  | Was an intention-to-treat analysis conducted? |  | 0 |
| 8. Potential confounders | Were potential confounders of the food health relationship considered? |  | 0 |
| TOTAL SCORE (maximum of 15): | | 8/15 |  |
| Higher quality (Score ≥ 8) | | X | |
| Lower quality (Score ≤ 7) | |  | |

Confounders: Physical activity during the study was not assessed. Therapeutic lifestyle change diet based on the American Heart Association was prescribed, but the adherence of the dietary prescription was not assessed.

| **Quality Appraisal Tool for Experimental Studies** | | | |
| --- | --- | --- | --- |
| **Study reference: Katz et al. (2012)** | | | |
| **Item** | **Question** | **Score** | |
|  |  | YES (1) | NO /NR(0) |
| 1. Inclusion/exclusion criteria | Were the inclusion and exclusion criteria for study participation reported? (eg. Age greater than 50 years, no history of heart disease)? | 1 |  |
| 2. Group allocation | Was the study described as randomized? | 1 |  |
|  | Was the randomization method reported? |  | 0 (NR) |
|  | Was the randomization appropriate? |  | 0 (NR) |
|  | Was the allocation concealed? |  | 0 (NR) |
| 3. Blinding | Were the study subjects blinded to the intervention received? |  | 0 (NR) |
|  | Were the researcher personnel blinded to the intervention received by the subjects? |  | 0 (NR) |
| 4. Attrition | Were attrition numerically reported? | 1 |  |
|  | Were the reasons for withdrawals and dropouts provided? | 1 |  |
| 5. Exposure/intervention | Was the type of food described (eg. Composition, matrix)? | 1 |  |
|  | Was the amount of food described (i.e. dose)? | 1 |  |
| 6. Health effect | Was the methodology used to measure the health effect reported? | 1 |  |
| 7. Statistical analysis | Was between group statistical analysis of the health effect reported? | 1 |  |
|  | Was an intention-to-treat analysis conducted? | 1 |  |
| 8. Potential confounders | Were potential confounders of the food health relationship considered? | 1 |  |
| TOTAL SCORE (maximum of 15): | | 10/15 |  |
| Higher quality (Score ≥ 8) | | X | |
| Lower quality (Score ≤ 7) | |  | |

Confounders considered included: age, race, BMI, hypertensive status, dyslipidaemia, treatment sequence

| **Quality Appraisal Tool for Experimental Studies** | | | |
| --- | --- | --- | --- |
| **Study reference:** Kay et al. (2010) | |  | |
| **Item** | **Question** | **Score** | |
|  |  | YES (1) | NO /NR(0) |
| 1. Inclusion/ Exclusion Criteria | Were the inclusion and/or exclusion criteria for study participation reported (*e.g.*, age greater than 50 years, no history of heart disease)? | 1 |  |
| 2. Group Allocation | Was the study described as randomized? | 1 |  |
|  | Was the randomization method reported? |  | 0 (NR) |
|  | Was the randomization method appropriate? |  | 0 (NR) |
|  | Was allocation concealed? |  | 0 (NR) |
| 3. Blinding | Were the study subjects blinded to the intervention received? |  | 0 (NR) |
|  | Were the research personnel blinded to the intervention received by the subjects? |  | 0 (NR) |
| 4. Attrition | Was attrition numerically reported? | 1 |  |
|  | Were the reasons for withdrawals and dropouts provided? | 1 |  |
| 5. Exposure/  Intervention | Was the type of food described (e.g., composition, matrix)? | 1 |  |
|  | Was the amount of food described (i.e., dose)? | 1 |  |
| 6. Health Effect | Was the methodology used to measure the health effect reported? | 1 |  |
| 7. Statistical Analysis | Was a between-group statistical analysis of the health effect conducted (*i.e.*, control vs. intervention)? | 1 |  |
|  | Was an intention-to-treat analysis conducted? |  | 0 (NR) |
| 8. Potential Confounders | Were potential confounders of the food/health relationship considered? | 1 |  |
| TOTAL SCORE (maximum of 15): | | 9/15 | |
| Higher quality (Score ≥ 8) | | X | |
| Lower quality (Score ≤ 7) | |  | |

Confounders: not applicable as randomised cross-over design

| **Quality Appraisal Tool for Experimental Studies** | | | |
| --- | --- | --- | --- |
| **Reference (Author, year):**  Khorramirad et al. (2021) | |  | |
| **Item** | **Question** | **Score** | |
|  |  | YES (1) | NO /NR(0) |
|  |  |  |  |
| 1. Inclusion/ Exclusion Criteria | Were the inclusion and/or exclusion criteria for study participation reported (*e.g.*, age greater than 50 years, no history of heart disease)? | 1 |  |
| 2. Group Allocation | Was the study described as randomized? | 1 |  |
|  | Was the randomization method reported? |  | 0 (NR) |
|  | Was the randomization method appropriate? |  | 0 (NR) |
|  | Was allocation concealed? |  | 0 (NR) |
| 3. Blinding | Were the study subjects blinded to the intervention received? |  | 0 (NR) |
|  | Were the research personnel blinded to the intervention received by the subjects? |  | 0 (NR) |
| 4. Attrition | Was attrition numerically reported? | 1 |  |
|  | Were the reasons for withdrawals and dropouts provided? | 1 |  |
| 5. Exposure/  Intervention | Was the type of food described (e.g., composition, matrix)? | 1 |  |
|  | Was the amount of food described (i.e., dose)? | 1 |  |
| 6. Health Effect | Was the methodology used to measure the health effect reported? | 1 |  |
| 7. Statistical Analysis | Was a between-group statistical analysis of the health effect conducted (*i.e.*, control vs. intervention)? | 1 |  |
|  | Was an intention-to-treat analysis conducted? |  | 0 |
| 8. Potential Confounders | Were potential confounders of the food/health relationship considered? | 1 |  |
| TOTAL SCORE (maximum of 15): | | 9/15 | |
| Higher quality (Score ≥ 8) | | X | |
| Lower quality (Score ≤ 7) | |  | |

Confounders: Described as single-blinded, however unclear if this referred to participants or personnel

Confounders: participants instructed not to alter physical activity

| **Quality Appraisal Tool for Experimental Studies** | | | |
| --- | --- | --- | --- |
| **Study reference: Kocyigit et al. (2006)** | | | |
| **Item** | **Question** | **Score** | |
|  |  | YES (1) | NO /NR(0) |
| 1. Inclusion/exclusion criteria | Were the inclusion and exclusion criteria for study participation reported? (eg. Age greater than 50 years, no history of heart disease)? | 1 |  |
| 2. Group allocation | Was the study described as randomized? | 1 |  |
|  | Was the randomization method reported? |  | 0 (NR) |
|  | Was the randomization appropriate? |  | 0 (NR) |
|  | Was the allocation concealed? |  | 0 (NR) |
| 3. Blinding | Were the study subjects blinded to the intervention received? |  | 0 (NR) |
|  | Were the researcher personnel blinded to the intervention received by the subjects? |  | 0 |
| 4. Attrition | Were attrition numerically reported? | 1 |  |
|  | Were the reasons for withdrawals and dropouts provided? | 1 |  |
| 5. Exposure/intervention | Was the type of food described (eg. Composition, matrix)? | 1 |  |
|  | Was the amount of food described (i.e. dose)? | 1 |  |
| 6. Health effect | Was the methodology used to measure the health effect reported? | 1 |  |
| 7. Statistical analysis | Was between group statistical analysis of the health effect reported? | 1 |  |
|  | Was an intention-to-treat analysis conducted? | 1 |  |
| 8. Potential confounders | Were potential confounders of the food health relationship considered? | 1 |  |
| TOTAL SCORE (maximum of 15): | | 10/15 |  |
| Higher quality (Score ≥ 8) | | X | |
| Lower quality (Score ≤ 7) | |  | |

Confounders: Compared dietary intake between groups, no difference in found in total energy or macronutrients between groups. No difference in baseline biochemical values between groups

| **Quality Appraisal Tool for Experimental Studies** | | | |
| --- | --- | --- | --- |
| **Study reference: Kris-Etherton et al. (1999)** | | | |
| **Item** | **Question** | **Score** | |
|  |  | YES (1) | NO /NR(0) |
| 1. Inclusion/exclusion criteria | Were the inclusion and exclusion criteria for study participation reported? (eg. Age greater than 50 years, no history of heart disease)? | 1 |  |
| 2. Group allocation | Was the study described as randomized? | 1 |  |
|  | Was the randomization method reported? |  | 0 (NR) |
|  | Was the randomization appropriate? |  | 0 (NR) |
|  | Was the allocation concealed? |  | 0 (NR) |
| 3. Blinding | Were the study subjects blinded to the intervention received? |  | 0 (NR) |
|  | Were the researcher personnel blinded to the intervention received by the subjects? |  | 0 (NR) |
| 4. Attrition | Were attrition numerically reported? | 1 |  |
|  | Were the reasons for withdrawals and dropouts provided? | 1 |  |
| 5. Exposure/intervention | Was the type of food described (eg. Composition, matrix)? | 1 |  |
|  | Was the amount of food described (i.e. dose)? |  | 0 |
| 6. Health effect | Was the methodology used to measure the health effect reported? | 1 |  |
| 7. Statistical analysis | Was between group statistical analysis of the health effect reported? | 1 |  |
|  | Was an intention-to-treat analysis conducted? |  | 0 |
| 8. Potential confounders | Were potential confounders of the food health relationship considered? | 1 |  |
| TOTAL SCORE (maximum of 15): | | 8/15 |  |
| Higher quality (Score ≥ 8) | | X | |
| Lower quality (Score ≤ 7) | |  | |

Confounders: Study states that no difference in cholesterol responses by sex and age has been previously reported, therefore it did not adjust for these factors.

| **Quality Appraisal Tool for Experimental Studies** | | | |
| --- | --- | --- | --- |
| **Study reference: Kurlandsky and Stote (2006)** | | | |
| **Item** | **Question** | **Score** | |
|  |  | YES (1) | NO /NR(0) |
| 1. Inclusion/exclusion criteria | Were the inclusion and exclusion criteria for study participation reported? (eg. Age greater than 50 years, no history of heart disease)? | 1 |  |
| 2. Group allocation | Was the study described as randomized? | 1 |  |
|  | Was the randomization method reported? |  | 0 (NR) |
|  | Was the randomization appropriate? |  | 0 (NR) |
|  | Was the allocation concealed? |  | 0 (NR) |
| 3. Blinding | Were the study subjects blinded to the intervention received? |  | 0 (NR) |
|  | Were the researcher personnel blinded to the intervention received by the subjects? |  | 0 |
| 4. Attrition | Were attrition numerically reported? | 1 |  |
|  | Were the reasons for withdrawals and dropouts provided? | 1 |  |
| 5. Exposure/intervention | Was the type of food described (eg. Composition, matrix)? | 1 |  |
|  | Was the amount of food described (i.e. dose)? | 1 |  |
| 6. Health effect | Was the methodology used to measure the health effect reported? | 1 |  |
| 7. Statistical analysis | Was between group statistical analysis of the health effect reported? | 1 |  |
|  | Was an intention-to-treat analysis conducted? |  | 0 |
| 8. Potential confounders | Were potential confounders of the food health relationship considered? | 1^1^ |  |
| TOTAL SCORE (maximum of 15): | | 9/15 |  |
| Higher quality (Score ≥ 8) | | X | |
| Lower quality (Score ≤ 7) | |  | |

Confounders: Instructed to maintain typical exercise patterns. Stanford 7 day PA survey completed at week 0 and 6 to confirm PA remained unchanged – “did not change”. Statistical analyses included stratification of the sample population by baseline LDL, age and use of HRT. Dietary energy significantly higher in almond than control group

| **Quality Appraisal Tool for Experimental Studies** | | | |
| --- | --- | --- | --- |
| **Study reference: Le et al (2016)/Rock et al (2016)** | | | |
| **Item** | **Question** | **Score** | |
|  |  | YES (1) | NO /NR(0) |
| 1. Inclusion/exclusion criteria | Were the inclusion and exclusion criteria for study participation reported? (eg. Age greater than 50 years, no history of heart disease)? | 1 |  |
| 2. Group allocation | Was the study described as randomized? | 1 |  |
|  | Was the randomization method reported? | 1 |  |
|  | Was the randomization appropriate? | 1 |  |
|  | Was the allocation concealed? | 1 |  |
| 3. Blinding | Were the study subjects blinded to the intervention received? |  | 0 (NR) |
|  | Were the researcher personnel blinded to the intervention received by the subjects? |  | 0 (NR) |
| 4. Attrition | Were attrition numerically reported? | 1 |  |
|  | Were the reasons for withdrawals and dropouts provided? |  | 0 (NR) |
| 5. Exposure/intervention | Was the type of food described (e.g.. Composition, matrix)? | 1 |  |
|  | Was the amount of food described (i.e. dose)? | 1 |  |
| 6. Health effect | Was the methodology used to measure the health effect reported? | 1 |  |
| 7. Statistical analysis | Was between group statistical analysis of the health effect reported? | 1 |  |
|  | Was an intention-to-treat analysis conducted? |  | 0 |
| 8. Potential confounders | Were potential confounders of the food health relationship considered? |  | 0 |
| TOTAL SCORE (maximum of 15): | | 10/15 |  |
| Higher quality (Score ≥ 8) | | X | |
| Lower quality (Score ≤ 7) | |  | |

Confounders: Physical activity during the study was not assessed.

| **Quality Appraisal Tool for Experimental Studies** | | | |
| --- | --- | --- | --- |
| **Study reference: Lee et al. (2014)** | | | |
| **Item** | **Question** | **Score** | |
|  |  | YES (1) | NO /NR(0) |
| 1. Inclusion/exclusion criteria | Were the inclusion and exclusion criteria for study participation reported? (eg. Age greater than 50 years, no history of heart disease)? | 1 |  |
| 2. Group allocation | Was the study described as randomized? | 1 |  |
|  | Was the randomization method reported? |  | 0 (NR) |
|  | Was the randomization appropriate? |  | 0 (NR) |
|  | Was the allocation concealed? |  | 0 (NR) |
| 3. Blinding | Were the study subjects blinded to the intervention received? |  | 0 (NR) |
|  | Were the researcher personnel blinded to the intervention received by the subjects? |  | 0 (NR) |
| 4. Attrition | Were attrition numerically reported? | 1 |  |
|  | Were the reasons for withdrawals and dropouts provided? | 1 |  |
| 5. Exposure/intervention | Was the type of food described (eg. Composition, matrix)? | 1 |  |
|  | Was the amount of food described (i.e. dose)? | 1 |  |
| 6. Health effect | Was the methodology used to measure the health effect reported? | 1 |  |
| 7. Statistical analysis | Was between group statistical analysis of the health effect reported? | 1 |  |
|  | Was an intention-to-treat analysis conducted? |  | 0 |
| 8. Potential confounders | Were potential confounders of the food health relationship considered? | 1 |  |
| TOTAL SCORE (maximum of 15): | | 9/15 |  |
| Higher quality (Score ≥ 8) | | X | |
| Lower quality (Score ≤ 7) | |  | |

Confounders: Analyses adjusted for baseline values, and sex –specific analyses were conducted for outcomes with significant results. No difference in energy intake between groups

| **Quality Appraisal Tool for Experimental Studies** | | | |
| --- | --- | --- | --- |
| **Study reference: Lee et al, (2017)** | | | |
| **Item** | **Question** | **Score** | |
|  |  | YES (1) | NO /NR(0) |
| 1. Inclusion/exclusion criteria | Were the inclusion and exclusion criteria for study participation reported? (eg. Age greater than 50 years, no history of heart disease)? | 1 |  |
| 2. Group allocation | Was the study described as randomized? | 1 |  |
|  | Was the randomization method reported? | 1 |  |
|  | Was the randomization appropriate? | 1 |  |
|  | Was the allocation concealed? | 1 |  |
| 3. Blinding | Were the study subjects blinded to the intervention received? |  | 0 |
|  | Were the researcher personnel blinded to the intervention received by the subjects? | 1 |  |
| 4. Attrition | Were attrition numerically reported? | 1 |  |
|  | Were the reasons for withdrawals and dropouts provided? | 1 |  |
| 5. Exposure/intervention | Was the type of food described (e.g.. Composition, matrix)? | 1 |  |
|  | Was the amount of food described (i.e. dose)? | 1 |  |
| 6. Health effect | Was the methodology used to measure the health effect reported? | 1 |  |
| 7. Statistical analysis | Was between group statistical analysis of the health effect reported? | 1 |  |
|  | Was an intention-to-treat analysis conducted? |  | 0 |
| 8. Potential confounders | Were potential confounders of the food health relationship considered? | 1 |  |
| TOTAL SCORE (maximum of 15): | | 13/15 |  |
| Higher quality (Score ≥ 8) | | X | |
| Lower quality (Score ≤ 7) | |  | |

Confounders: There was no identified potential confounders. The consumed foods were provided by the study, and intake adherence was assessed.

| **Quality Appraisal Tool for Experimental Studies** | | | |
| --- | --- | --- | --- |
| **Study reference: Li et al. (2010)** | | | |
| **Item** | **Question** | **Score** | |
|  |  | YES (1) | NO /NR(0) |
| 1. Inclusion/exclusion criteria | Were the inclusion and exclusion criteria for study participation reported? (eg. Age greater than 50 years, no history of heart disease)? | 1 |  |
| 2. Group allocation | Was the study described as randomized? | 1 |  |
|  | Was the randomization method reported? | 1 |  |
|  | Was the randomization appropriate? | 1 |  |
|  | Was the allocation concealed? |  | 0 (NR) |
| 3. Blinding | Were the study subjects blinded to the intervention received? |  | 0 (NR) |
|  | Were the researcher personnel blinded to the intervention received by the subjects? |  | 0 (NR) |
| 4. Attrition | Were attrition numerically reported? | 1 |  |
|  | Were the reasons for withdrawals and dropouts provided? | 1 |  |
| 5. Exposure/intervention | Was the type of food described (eg. Composition, matrix)? | 1 |  |
|  | Was the amount of food described (i.e. dose)? | 1 |  |
| 6. Health effect | Was the methodology used to measure the health effect reported? | 1 |  |
| 7. Statistical analysis | Was between group statistical analysis of the health effect reported? | 1 |  |
|  | Was an intention-to-treat analysis conducted? |  | 0 |
| 8. Potential confounders | Were potential confounders of the food health relationship considered? |  | 0 |
| TOTAL SCORE (maximum of 15): | | 10/15 |  |
| Higher quality (Score ≥ 8) | | X | |
| Lower quality (Score ≤ 7) | |  | |

Confounders: Similar baseline health characteristics found. However, as dietary information was collected but not reported, the study was deemed to have not considered dietary confounders

| **Quality Appraisal Tool for Experimental Studies** | | | |
| --- | --- | --- | --- |
| **Study reference: Lima et al, (2017)** | | | |
| **Item** | **Question** | **Score** | |
|  |  | YES (1) | NO /NR(0) |
| 1. Inclusion/exclusion criteria | Were the inclusion and exclusion criteria for study participation reported? (eg. Age greater than 50 years, no history of heart disease)? | 1 |  |
| 2. Group allocation | Was the study described as randomized? | 1 |  |
|  | Was the randomization method reported? |  | 0(NR) |
|  | Was the randomization appropriate? |  | 0(NR) |
|  | Was the allocation concealed? |  | 0(NR) |
| 3. Blinding | Were the study subjects blinded to the intervention received? | 1 |  |
|  | Were the researcher personnel blinded to the intervention received by the subjects? | 1 |  |
| 4. Attrition | Were attrition numerically reported? |  | 0(NR) |
|  | Were the reasons for withdrawals and dropouts provided? |  | 0(NR) |
| 5. Exposure/intervention | Was the type of food described (e.g.. Composition, matrix)? | 1 |  |
|  | Was the amount of food described (i.e. dose)? | 1 |  |
| 6. Health effect | Was the methodology used to measure the health effect reported? | 1 |  |
| 7. Statistical analysis | Was between group statistical analysis of the health effect reported? | 1 |  |
|  | Was an intention-to-treat analysis conducted? |  | 0 |
| 8. Potential confounders | Were potential confounders of the food health relationship considered? |  | 0 |
| TOTAL SCORE (maximum of 15): | | 7/15 |  |
| Higher quality (Score ≥ 8) | |  | |
| Lower quality (Score ≤ 7) | | X | |

Confounders: There were differences on the levels of LDL-C (107.7 vs 125.4) and HDL-C (49.6 vs 43) between groups at baseline.

| **Quality Appraisal Tool for Experimental Studies** | | | |
| --- | --- | --- | --- |
| **Study reference: Li et al. (2011)/Liu et al. (2013)** | | | |
| **Item** | **Question** | **Score** | |
|  |  | YES (1) | NO /NR(0) |
| 1. Inclusion/exclusion criteria | Were the inclusion and exclusion criteria for study participation reported? (eg. Age greater than 50 years, no history of heart disease)? | 1 |  |
| 2. Group allocation | Was the study described as randomized? | 1 |  |
|  | Was the randomization method reported? |  | 0 |
|  | Was the randomization appropriate? |  | 0 (NR) |
|  | Was the allocation concealed? |  | 0 (NR) |
| 3. Blinding | Were the study subjects blinded to the intervention received? |  | 0 |
|  | Were the researcher personnel blinded to the intervention received by the subjects? |  | 0 |
| 4. Attrition | Were attrition numerically reported? | 1 |  |
|  | Were the reasons for withdrawals and dropouts provided? | 1 |  |
| 5. Exposure/intervention | Was the type of food described (eg. Composition, matrix)? | 1 |  |
|  | Was the amount of food described (i.e. dose)? | 1 |  |
| 6. Health effect | Was the methodology used to measure the health effect reported? | 1 |  |
| 7. Statistical analysis | Was between group statistical analysis of the health effect reported? | 1 |  |
|  | Was an intention-to-treat analysis conducted? |  | 0 |
| 8. Potential confounders | Were potential confounders of the food health relationship considered? | 1 |  |
| TOTAL SCORE (maximum of 15): | | 9/15 |  |
| Higher quality (Score ≥ 8) | |  | |
| Lower quality (Score ≤ 7) | |  | |

Confounders: Effect of sex assessed (no effect). Sequence and study period included in analysis

| **Quality Appraisal Tool for Experimental Studies** | | | |
| --- | --- | --- | --- |
| **Study reference:** Liu et al, (2018) | | | |
| **Item** | **Question** | **Score** | |
|  |  | YES (1) | NO /NR(0) |
| 1. Inclusion/exclusion criteria | Were the inclusion and exclusion criteria for study participation reported? (eg. Age greater than 50 years, no history of heart disease)? | 1 |  |
| 2. Group allocation | Was the study described as randomized? | 1 |  |
|  | Was the randomization method reported? |  | 0 (NR) |
|  | Was the randomization appropriate? |  | 0 (NR) |
|  | Was the allocation concealed? |  | 0 (NR) |
| 3. Blinding | Were the study subjects blinded to the intervention received? |  | 0 (NR) |
|  | Were the researcher personnel blinded to the intervention received by the subjects? |  | 0 (NR) |
| 4. Attrition | Were attrition numerically reported? | 1 |  |
|  | Were the reasons for withdrawals and dropouts provided? |  | 0 (NR) |
| 5. Exposure/intervention | Was the type of food described (e.g.. Composition, matrix)? | 1 |  |
|  | Was the amount of food described (i.e. dose)? | 1 |  |
| 6. Health effect | Was the methodology used to measure the health effect reported? | 1 |  |
| 7. Statistical analysis | Was between group statistical analysis of the health effect reported? | 1 |  |
|  | Was an intention-to-treat analysis conducted? |  | 0 |
| 8. Potential confounders | Were potential confounders of the food health relationship considered? |  | 0 |
| TOTAL SCORE (maximum of 15): | | 7/15 |  |
| Higher quality (Score ≥ 8) | |  | |
| Lower quality (Score ≤ 7) | | X | |

Confounders: There was a significant difference on TC at baseline between groups (p=0.041). The total fat and PUFA intakes between two groups was 15g and 4.3g different at baseline.

| **Quality Appraisal Tool for Experimental Studies** | | | |
| --- | --- | --- | --- |
| **Study reference: Lovejoy et al. (2002)** | | | |
| **Item** | **Question** | **Score** | |
|  |  | YES (1) | NO /NR(0) |
| 1. Inclusion/exclusion criteria | Were the inclusion and exclusion criteria for study participation reported? (eg. Age greater than 50 years, no history of heart disease)? | 1 |  |
| 2. Group allocation | Was the study described as randomized? | 1 |  |
|  | Was the randomization method reported? |  | 0 (NR) |
|  | Was the randomization appropriate? |  | 0 (NR) |
|  | Was the allocation concealed? |  | 0 (NR) |
| 3. Blinding | Were the study subjects blinded to the intervention received? | 1 |  |
|  | Were the researcher personnel blinded to the intervention received by the subjects? | 1 |  |
| 4. Attrition | Were attrition numerically reported? | 1 |  |
|  | Were the reasons for withdrawals and dropouts provided? | 1 |  |
| 5. Exposure/intervention | Was the type of food described (eg. Composition, matrix)? | 1 |  |
|  | Was the amount of food described (i.e. dose)? | 1 |  |
| 6. Health effect | Was the methodology used to measure the health effect reported? | 1 |  |
| 7. Statistical analysis | Was between group statistical analysis of the health effect reported? | 1 |  |
|  | Was an intention-to-treat analysis conducted? |  | 0 |
| 8. Potential confounders | Were potential confounders of the food health relationship considered? | 1 |  |
| TOTAL SCORE (maximum of 15): | | 11/15 |  |
| Higher quality (Score ≥ 8) | | X | |
| Lower quality (Score ≤ 7) | |  | |

Confounders: total energy intake, diet order was not included in the final analysis as it was not a significant covariate for any analysis

| **Quality Appraisal Tool for Experimental Studies** | | | |
| --- | --- | --- | --- |
| **Study reference: Ma et al. (2010)** | | | |
| **Item** | **Question** | **Score** | |
|  |  | YES (1) | NO /NR(0) |
| 1. Inclusion/exclusion criteria | Were the inclusion and exclusion criteria for study participation reported? (eg. Age greater than 50 years, no history of heart disease)? | 1 |  |
| 2. Group allocation | Was the study described as randomized? | 1 |  |
|  | Was the randomization method reported? |  | 0 (NR) |
|  | Was the randomization appropriate? |  | 0 (NR) |
|  | Was the allocation concealed? |  | 0 (NR) |
| 3. Blinding | Were the study subjects blinded to the intervention received? |  | 0 |
|  | Were the researcher personnel blinded to the intervention received by the subjects? | 1 |  |
| 4. Attrition | Were attrition numerically reported? | 1 |  |
|  | Were the reasons for withdrawals and dropouts provided? | 1 |  |
| 5. Exposure/intervention | Was the type of food described (eg. Composition, matrix)? | 1 |  |
|  | Was the amount of food described (i.e. dose)? | 1 |  |
| 6. Health effect | Was the methodology used to measure the health effect reported? | 1 |  |
| 7. Statistical analysis | Was between group statistical analysis of the health effect reported? | 1 |  |
|  | Was an intention-to-treat analysis conducted? | 1 |  |
| 8. Potential confounders | Were potential confounders of the food health relationship considered? | 1 |  |
| TOTAL SCORE (maximum of 15): | | 11/15 |  |
| Higher quality (Score ≥ 8) | | X | |
| Lower quality (Score ≤ 7) | |  | |

Confounders: Combined effect of independent variables (age, race, BMI, hypertension, dyslipidemia, treatment sequence) on outcome measures was assessed with multivariate models. Intention –to-treat used

| **Quality Appraisal Tool for Experimental Studies** | | | |
| --- | --- | --- | --- |
| **Reference (Author, year):**  Madan et al. (2021) | |  | |
| **Item** | **Question** | **Score** | |
|  |  | YES (1) | NO /NR(0) |
|  |  |  |  |
| 1. Inclusion/ Exclusion Criteria | Were the inclusion and/or exclusion criteria for study participation reported (*e.g.*, age greater than 50 years, no history of heart disease)? | 1 |  |
| 2. Group Allocation | Was the study described as randomized? | 1 |  |
|  | Was the randomization method reported? |  | 0 (NR) |
|  | Was the randomization method appropriate? |  | 0 (NR) |
|  | Was allocation concealed? |  | 0 (NR) |
| 3. Blinding | Were the study subjects blinded to the intervention received? |  | 0 (NR) |
|  | Were the research personnel blinded to the intervention received by the subjects? |  | 0 (NR) |
| 4. Attrition | Was attrition numerically reported? | 1 |  |
|  | Were the reasons for withdrawals and dropouts provided? | 1 |  |
| 5. Exposure/  Intervention | Was the type of food described (e.g., composition, matrix)? | 1 |  |
|  | Was the amount of food described (i.e., dose)? | 1 |  |
| 6. Health Effect | Was the methodology used to measure the health effect reported? | 1 |  |
| 7. Statistical Analysis | Was a between-group statistical analysis of the health effect conducted (*i.e.*, control vs. intervention)? | 1 |  |
|  | Was an intention-to-treat analysis conducted? | 1 |  |
| 8. Potential Confounders | Were potential confounders of the food/health relationship considered? | 1 |  |
| TOTAL SCORE (maximum of 15): | | 10/15 | |
| Higher quality (Score ≥ 8) | | X | |
| Lower quality (Score ≤ 7) | |  | |

Confounders: participants advised to continue usual diet and physical activity

| **Quality Appraisal Tool for Experimental Studies** | | | |
| --- | --- | --- | --- |
| **Study reference: Mah et al (2017)** | | | |
| **Item** | **Question** | **Score** | |
|  |  | YES (1) | NO /NR(0) |
| 1. Inclusion/exclusion criteria | Were the inclusion and exclusion criteria for study participation reported? (eg. Age greater than 50 years, no history of heart disease)? | 1 |  |
| 2. Group allocation | Was the study described as randomized? | 1 |  |
|  | Was the randomization method reported? | 1 |  |
|  | Was the randomization appropriate? | 1 |  |
|  | Was the allocation concealed? | 1 |  |
| 3. Blinding | Were the study subjects blinded to the intervention received? |  | 0 |
|  | Were the researcher personnel blinded to the intervention received by the subjects? |  | 0 |
| 4. Attrition | Were attrition numerically reported? | 1 |  |
|  | Were the reasons for withdrawals and dropouts provided? | 1 |  |
| 5. Exposure/intervention | Was the type of food described (e.g.. Composition, matrix)? | 1 |  |
|  | Was the amount of food described (i.e. dose)? | 1 |  |
| 6. Health effect | Was the methodology used to measure the health effect reported? | 1 |  |
| 7. Statistical analysis | Was between group statistical analysis of the health effect reported? | 1 |  |
|  | Was an intention-to-treat analysis conducted? | 1 |  |
| 8. Potential confounders | Were potential confounders of the food health relationship considered? | 1 |  |
| TOTAL SCORE (maximum of 15): | | 13/15 |  |
| Higher quality (Score ≥ 8) | | X | |
| Lower quality (Score ≤ 7) | |  | |

Confounders: There was no identified potential confounders. The consumed foods were provided by study.

| **Quality Appraisal Tool for Experimental Studies** | | | |
| --- | --- | --- | --- |
| **Study reference: Maranhão et al. (2011)** | | | |
| **Item** | **Question** | **Score** | |
|  |  | YES (1) | NO /NR(0) |
| 1. Inclusion/exclusion criteria | Were the inclusion and exclusion criteria for study participation reported? (eg. Age greater than 50 years, no history of heart disease)? | 1 |  |
| 2. Group allocation | Was the study described as randomized? | 1 |  |
|  | Was the randomization method reported? |  | 0 (NR) |
|  | Was the randomization appropriate? |  | 0 (NR) |
|  | Was the allocation concealed? |  | 0 (NR) |
| 3. Blinding | Were the study subjects blinded to the intervention received? |  | 0 |
|  | Were the researcher personnel blinded to the intervention received by the subjects? |  | 0 |
| 4. Attrition | Were attrition numerically reported? |  | 0 (NR) |
|  | Were the reasons for withdrawals and dropouts provided? |  | 0 (NR) |
| 5. Exposure/intervention | Was the type of food described (eg. Composition, matrix)? | 1 |  |
|  | Was the amount of food described (i.e. dose)? | 1 |  |
| 6. Health effect | Was the methodology used to measure the health effect reported? | 1 |  |
| 7. Statistical analysis | Was between group statistical analysis of the health effect reported? |  | 0 (NR) |
|  | Was an intention-to-treat analysis conducted? |  | 0 (NR) |
| 8. Potential confounders | Were potential confounders of the food health relationship considered? |  | 0 |
| TOTAL SCORE (maximum of 15): | | 5/15 |  |
| Higher quality (Score ≥ 8) | |  | |
| Lower quality (Score ≤ 7) | | X | |

Confounders: Measured dietary intake but did not report or discuss

| **Quality Appraisal Tool for Experimental Studies** | | | |
| --- | --- | --- | --- |
| **Study reference:** McKay et al. (2010) | |  | |
| **Item** | **Question** | **Score** | |
|  |  | Yes | No/NR |
| 1. Inclusion/ Exclusion Criteria | Were the inclusion and/or exclusion criteria for study participation reported (*e.g.*, age greater than 50 years, no history of heart disease)? | 1 |  |
| 2. Group Allocation | Was the study described as randomized? | 1 |  |
|  | Was the randomization method reported? | 1 |  |
|  | Was the randomization method appropriate? | 1 |  |
|  | Was allocation concealed? |  | 0 (NR) |
| 3. Blinding | Were the study subjects blinded to the intervention received? |  | 0 (NR) |
|  | Were the research personnel blinded to the intervention received by the subjects? | 1 |  |
| 4. Attrition | Was attrition numerically reported? | 1 |  |
|  | Were the reasons for withdrawals and dropouts provided? | 1 |  |
| 5. Exposure/  Intervention | Was the type of food described (e.g., composition, matrix)? | 1 |  |
|  | Was the amount of food described (i.e., dose)? | 1 |  |
| 6. Health Effect | Was the methodology used to measure the health effect reported? | 1 |  |
| 7. Statistical Analysis | Was a between-group statistical analysis of the health effect conducted (*i.e.*, control vs. intervention)? | 1 |  |
|  | Was an intention-to-treat analysis conducted? | 1 |  |
| 8. Potential Confounders | Were potential confounders of the food/health relationship considered? | 1 |  |
| TOTAL SCORE (maximum of 15): | | 13/15 | |
| Higher quality (Score ≥ 8) | | X | |
| Lower quality (Score ≤ 7) | |  | |

Confounders: not applicable as randomised cross-over design

| **Quality Appraisal Tool for Experimental Studies** | | | |
| --- | --- | --- | --- |
| **Study reference: McKay et al (2018)** | | | |
| **Item** | **Question** | **Score** | |
|  |  | YES (1) | NO /NR(0) |
| 1. Inclusion/exclusion criteria | Were the inclusion and exclusion criteria for study participation reported? (eg. Age greater than 50 years, no history of heart disease)? | 1 |  |
| 2. Group allocation | Was the study described as randomized? | 1 |  |
|  | Was the randomization method reported? | 1 |  |
|  | Was the randomization appropriate? | 1 |  |
|  | Was the allocation concealed? | 1 |  |
| 3. Blinding | Were the study subjects blinded to the intervention received? |  | 0 |
|  | Were the researcher personnel blinded to the intervention received by the subjects? | 1 |  |
| 4. Attrition | Were attrition numerically reported? | 1 |  |
|  | Were the reasons for withdrawals and dropouts provided? | 1 |  |
| 5. Exposure/intervention | Was the type of food described (e.g.. Composition, matrix)? | 1 |  |
|  | Was the amount of food described (i.e. dose)? | 1 |  |
| 6. Health effect | Was the methodology used to measure the health effect reported? | 1 |  |
| 7. Statistical analysis | Was between group statistical analysis of the health effect reported? | 1 |  |
|  | Was an intention-to-treat analysis conducted? |  | 0 |
| 8. Potential confounders | Were potential confounders of the food health relationship considered? | 1 |  |
| TOTAL SCORE (maximum of 15): | | 13/15 |  |
| Higher quality (Score ≥ 8) | | X | |
| Lower quality (Score ≤ 7) | |  | |

Confounders: There was no identified potential confounders.

| **Quality Appraisal Tool for Experimental Studies** | | | |
| --- | --- | --- | --- |
| **Study reference: Mohan et al (2018)** | | | |
| **Item** | **Question** | **Score** | |
|  |  | YES (1) | NO /NR(0) |
| 1. Inclusion/exclusion criteria | Were the inclusion and exclusion criteria for study participation reported? (eg. Age greater than 50 years, no history of heart disease)? | 1 |  |
| 2. Group allocation | Was the study described as randomized? | 1 |  |
|  | Was the randomization method reported? | 1 |  |
|  | Was the randomization appropriate? | 1 |  |
|  | Was the allocation concealed? | 1 |  |
| 3. Blinding | Were the study subjects blinded to the intervention received? |  | 0 (NR) |
|  | Were the researcher personnel blinded to the intervention received by the subjects? |  | 0 (NR) |
| 4. Attrition | Were attrition numerically reported? | 1 |  |
|  | Were the reasons for withdrawals and dropouts provided? | 1 |  |
| 5. Exposure/intervention | Was the type of food described (e.g.. Composition, matrix)? | 1 |  |
|  | Was the amount of food described (i.e. dose)? | 1 |  |
| 6. Health effect | Was the methodology used to measure the health effect reported? | 1 |  |
| 7. Statistical analysis | Was between group statistical analysis of the health effect reported? | 1 |  |
|  | Was an intention-to-treat analysis conducted? |  | 0 |
| 8. Potential confounders | Were potential confounders of the food health relationship considered? | 1 |  |
| TOTAL SCORE (maximum of 15): | | 12/15 |  |
| Higher quality (Score ≥ 8) | | X | |
| Lower quality (Score ≤ 7) | |  | |

Confounders: Biomarkers were used to assess the adherence of dietary intervention.

| **Quality Appraisal Tool for Experimental Studies** | | | |
| --- | --- | --- | --- |
| **Study reference: Morgan and Clayshulte (2000)** | | | |
| **Item** | **Question** | **Score** | |
|  |  | YES (1) | NO /NR(0) |
| 1. Inclusion/exclusion criteria | Were the inclusion and exclusion criteria for study participation reported? (eg. Age greater than 50 years, no history of heart disease)? | 1 |  |
| 2. Group allocation | Was the study described as randomized? | 1 |  |
|  | Was the randomization method reported? |  | 0 (NR) |
|  | Was the randomization appropriate? |  | 0 (NR) |
|  | Was the allocation concealed? |  | 0 (NR) |
| 3. Blinding | Were the study subjects blinded to the intervention received? |  | 0 |
|  | Were the researcher personnel blinded to the intervention received by the subjects? |  | 0 |
| 4. Attrition | Were attrition numerically reported? | 1 |  |
|  | Were the reasons for withdrawals and dropouts provided? | 1 |  |
| 5. Exposure/intervention | Was the type of food described (eg. Composition, matrix)? | 1 |  |
|  | Was the amount of food described (i.e. dose)? | 1 |  |
| 6. Health effect | Was the methodology used to measure the health effect reported? | 1 |  |
| 7. Statistical analysis | Was between group statistical analysis of the health effect reported? | 1 |  |
|  | Was an intention-to-treat analysis conducted? |  | 0 |
| 8. Potential confounders | Were potential confounders of the food health relationship considered? |  | 0 |
| TOTAL SCORE (maximum of 15): | | 8/15 |  |
| Higher quality (Score ≥ 8) | | X | |
| Lower quality (Score ≤ 7) | |  | |

Confounders: Some differences between groups in dietary intake at baseline (not controlled for), statistical analyses not adjusted for any confounder

| **Quality Appraisal Tool for Experimental Studies** | | | |
| --- | --- | --- | --- |
| **Study reference: Morgan et al. (2002)** | | | |
| **Item** | **Question** | **Score** | |
|  |  | YES (1) | NO /NR(0) |
| 1. Inclusion/exclusion criteria | Were the inclusion and exclusion criteria for study participation reported? (eg. Age greater than 50 years, no history of heart disease)? | 1 |  |
| 2. Group allocation | Was the study described as randomized? | 1 |  |
|  | Was the randomization method reported? |  | 0 (NR) |
|  | Was the randomization appropriate? |  | 0 (NR) |
|  | Was the allocation concealed? |  | 0 (NR) |
| 3. Blinding | Were the study subjects blinded to the intervention received? |  | 0 (NR) |
|  | Were the researcher personnel blinded to the intervention received by the subjects? |  | 0 (NR) |
| 4. Attrition | Were attrition numerically reported? | 1 |  |
|  | Were the reasons for withdrawals and dropouts provided? | 1 |  |
| 5. Exposure/intervention | Was the type of food described (eg. Composition, matrix)? | 1 |  |
|  | Was the amount of food described (i.e. dose)? | 1 |  |
| 6. Health effect | Was the methodology used to measure the health effect reported? | 1 |  |
| 7. Statistical analysis | Was between group statistical analysis of the health effect reported? |  | 0 (NR) |
|  | Was an intention-to-treat analysis conducted? |  | 0 |
| 8. Potential confounders | Were potential confounders of the food health relationship considered? |  | 0 |
| TOTAL SCORE (maximum of 15): | | 7/15 |  |
| Higher quality (Score ≥ 8) | |  | |
| Lower quality (Score ≤ 7) | | X | |

Confounders: None included in analysis

| **Quality Appraisal Tool for Experimental Studies** | | | |
| --- | --- | --- | --- |
| **Study reference: Mukuddem- Petersen et al. (2007)/** **Schutte et al. (2006)** | | | |
| **Item** | **Question** | **Score** | |
|  |  | YES (1) | NO /NR(0) |
| 1. Inclusion/exclusion criteria | Were the inclusion and exclusion criteria for study participation reported? (eg. Age greater than 50 years, no history of heart disease)? | 1 |  |
| 2. Group allocation | Was the study described as randomized? | 1 |  |
|  | Was the randomization method reported? | 1 |  |
|  | Was the randomization appropriate? | 1 |  |
|  | Was the allocation concealed? |  | 0 (NR) |
| 3. Blinding | Were the study subjects blinded to the intervention received? |  | 0 |
|  | Were the researcher personnel blinded to the intervention received by the subjects? |  | 0 (NR) |
| 4. Attrition | Were attrition numerically reported? | 1 |  |
|  | Were the reasons for withdrawals and dropouts provided? | 1 |  |
| 5. Exposure/intervention | Was the type of food described (eg. Composition, matrix)? | 1 |  |
|  | Was the amount of food described (i.e. dose)? | 1 |  |
| 6. Health effect | Was the methodology used to measure the health effect reported? | 1 |  |
| 7. Statistical analysis | Was between group statistical analysis of the health effect reported? | 1 |  |
|  | Was an intention-to-treat analysis conducted? |  | 0 |
| 8. Potential confounders | Were potential confounders of the food health relationship considered? | 1 |  |
| TOTAL SCORE (maximum of 15): | | 11/15 |  |
| Higher quality (Score ≥ 8) | | X | |
| Lower quality (Score ≤ 7) | |  | |

Confounders: Participants grouped by gender and age prior to randomisation. Baseline characteristics did not differ between groups.

| **Quality Appraisal Tool for Experimental Studies** | | | |
| --- | --- | --- | --- |
| **Study reference: Müllner et al. (2013)** | | | |
| **Item** | **Question** | **Score** | |
|  |  | YES (1) | NO /NR(0) |
| 1. Inclusion/exclusion criteria | Were the inclusion and exclusion criteria for study participation reported? (eg. Age greater than 50 years, no history of heart disease)? | 1 |  |
| 2. Group allocation | Was the study described as randomized? | 1 |  |
|  | Was the randomization method reported? |  | 0 NR |
|  | Was the randomization appropriate? |  | 0 NR |
|  | Was the allocation concealed? |  | 0 NR |
| 3. Blinding | Were the study subjects blinded to the intervention received? | 1 |  |
|  | Were the researcher personnel blinded to the intervention received by the subjects? | 1 |  |
| 4. Attrition | Were attrition numerically reported? | 1 |  |
|  | Were the reasons for withdrawals and dropouts provided? | 1 |  |
| 5. Exposure/intervention | Was the type of food described (eg. Composition, matrix)? | 1 |  |
|  | Was the amount of food described (i.e. dose)? | 1 |  |
| 6. Health effect | Was the methodology used to measure the health effect reported? | 1 |  |
| 7. Statistical analysis | Was between group statistical analysis of the health effect reported? | 1 |  |
|  | Was an intention-to-treat analysis conducted? |  | 0 |
| 8. Potential confounders | Were potential confounders of the food health relationship considered? |  | 0 |
| TOTAL SCORE (maximum of 15): | | 10/15 |  |
| Higher quality (Score ≥ 8) | | X | |
| Lower quality (Score ≤ 7) | |  | |

Confounders: Total diet only assessed at baseline, whilst blood data to estimate compliance was collected, it may not identify differences in intakes of other nutrients between the intervention arms

| **Quality Appraisal Tool for Experimental Studies** | | | |
| --- | --- | --- | --- |
| **Study reference: Nestel et al. (1994)** | | | |
| **Item** | **Question** | **Score** | |
|  |  | YES (1) | NO /NR(0) |
| 1. Inclusion/exclusion criteria | Were the inclusion and exclusion criteria for study participation reported? (eg. Age greater than 50 years, no history of heart disease)? | 1 |  |
| 2. Group allocation | Was the study described as randomized? | 1 |  |
|  | Was the randomization method reported? |  | 0 (NR) |
|  | Was the randomization appropriate? |  | 0 (NR) |
|  | Was the allocation concealed? |  | 0 (NR) |
| 3. Blinding | Were the study subjects blinded to the intervention received? | 1 |  |
|  | Were the researcher personnel blinded to the intervention received by the subjects? |  | 0 (NR) |
| 4. Attrition | Were attrition numerically reported? | 1 |  |
|  | Were the reasons for withdrawals and dropouts provided? | 1 |  |
| 5. Exposure/intervention | Was the type of food described (eg. Composition, matrix)? | 1 |  |
|  | Was the amount of food described (i.e. dose)? | 1 |  |
| 6. Health effect | Was the methodology used to measure the health effect reported? | 1 |  |
| 7. Statistical analysis | Was between group statistical analysis of the health effect reported? | 1 |  |
|  | Was an intention-to-treat analysis conducted? | 1 |  |
| 8. Potential confounders | Were potential confounders of the food health relationship considered? | 1 |  |
| TOTAL SCORE (maximum of 15): | | 11/15 |  |
| Higher quality (Score ≥ 8) | | X | |
| Lower quality (Score ≤ 7) | |  | |

Confounders: Analyses adjusted for time and treatment order

| **Quality Appraisal Tool for Experimental Studies** | | | |
| --- | --- | --- | --- |
| **Study reference: Nezhad et al. (2016)** | | | |
| **Item** | **Question** | **Score** | |
|  |  | YES (1) | NO /NR(0) |
| 1. Inclusion/exclusion criteria | Were the inclusion and exclusion criteria for study participation reported? (eg. Age greater than 50 years, no history of heart disease)? | 1 |  |
| 2. Group allocation | Was the study described as randomized? | 1 |  |
|  | Was the randomization method reported? | 1 |  |
|  | Was the randomization appropriate? | 1 |  |
|  | Was the allocation concealed? | 1 |  |
| 3. Blinding | Were the study subjects blinded to the intervention received? |  | 0(NR) |
|  | Were the researcher personnel blinded to the intervention received by the subjects? |  | 0(NR) |
| 4. Attrition | Were attrition numerically reported? | 1 |  |
|  | Were the reasons for withdrawals and dropouts provided? | 1 |  |
| 5. Exposure/intervention | Was the type of food described (e.g.. Composition, matrix)? | 1 |  |
|  | Was the amount of food described (i.e. dose)? | 1 |  |
| 6. Health effect | Was the methodology used to measure the health effect reported? | 1 |  |
| 7. Statistical analysis | Was between group statistical analysis of the health effect reported? | 1 |  |
|  | Was an intention-to-treat analysis conducted? |  | 0 |
| 8. Potential confounders | Were potential confounders of the food health relationship considered? | 1 |  |
| TOTAL SCORE (maximum of 15): | | 12/15 |  |
| Higher quality (Score ≥ 8) | | X | |
| Lower quality (Score ≤ 7) | |  | |

Confounders: Dietetics counselling was applied to maintain body weight during the study. The adherence of dietary intervention was assessed.

| **Quality Appraisal Tool for Experimental Studies** | | | |
| --- | --- | --- | --- |
| **Study reference: Njike et al (2015)** | | | |
| **Item** | **Question** | **Score** | |
|  |  | YES (1) | NO /NR(0) |
| 1. Inclusion/exclusion criteria | Were the inclusion and exclusion criteria for study participation reported? (eg. Age greater than 50 years, no history of heart disease)? | 1 |  |
| 2. Group allocation | Was the study described as randomized? | 1 |  |
|  | Was the randomization method reported? | 1 |  |
|  | Was the randomization appropriate? | 1 |  |
|  | Was the allocation concealed? | 1 |  |
| 3. Blinding | Were the study subjects blinded to the intervention received? |  | 0 (NR) |
|  | Were the researcher personnel blinded to the intervention received by the subjects? |  | 0 (NR) |
| 4. Attrition | Were attrition numerically reported? | 1 |  |
|  | Were the reasons for withdrawals and dropouts provided? | 1 |  |
| 5. Exposure/intervention | Was the type of food described (e.g.. Composition, matrix)? | 1 |  |
|  | Was the amount of food described (i.e. dose)? | 1 |  |
| 6. Health effect | Was the methodology used to measure the health effect reported? | 1 |  |
| 7. Statistical analysis | Was between group statistical analysis of the health effect reported? | 1 |  |
|  | Was an intention-to-treat analysis conducted? | 1 |  |
| 8. Potential confounders | Were potential confounders of the food health relationship considered? |  | 0 |
| TOTAL SCORE (maximum of 15): | | 12/15 |  |
| Higher quality (Score ≥ 8) | | X | |
| Lower quality (Score ≤ 7) | |  | |

Confounders: The adherences of calorie-adjusted or no calorie diets were not assessed.

| **Quality Appraisal Tool for Experimental Studies** | | | |
| --- | --- | --- | --- |
| **Study reference: Nouran et al. (2009)** | | | |
| **Item** | **Question** | **Score** | |
|  |  | YES (1) | NO /NR(0) |
| 1. Inclusion/exclusion criteria | Were the inclusion and exclusion criteria for study participation reported? (eg. Age greater than 50 years, no history of heart disease)? | 1 |  |
| 2. Group allocation | Was the study described as randomized? | 1 |  |
|  | Was the randomization method reported? |  | 0 |
|  | Was the randomization appropriate? |  | 0 |
|  | Was the allocation concealed? |  | 0 |
| 3. Blinding | Were the study subjects blinded to the intervention received? |  | 0 |
|  | Were the researcher personnel blinded to the intervention received by the subjects? |  | 0 |
| 4. Attrition | Were attrition numerically reported? | 1 |  |
|  | Were the reasons for withdrawals and dropouts provided? | 1 |  |
| 5. Exposure/intervention | Was the type of food described (eg. Composition, matrix)? | 1 |  |
|  | Was the amount of food described (i.e. dose)? | 1 |  |
| 6. Health effect | Was the methodology used to measure the health effect reported? | 1 |  |
| 7. Statistical analysis | Was between group statistical analysis of the health effect reported? | 1 |  |
|  | Was an intention-to-treat analysis conducted? |  | 0 |
| 8. Potential confounders | Were potential confounders of the food health relationship considered? |  | 0 |
| TOTAL SCORE (maximum of 15): | | 8/15 |  |
| Higher quality (Score ≥ 8) | | X | |
| Lower quality (Score ≤ 7) | |  | |

Confounders: Energy intake significantly greater during peanut period and this was not adjusted for in the analysis

| **Quality Appraisal Tool for Experimental Studies** | | | |
| --- | --- | --- | --- |
| **Study reference:** O’Byrne et al. (1997) | | | |
| **Item** | **Question** | **Score** | |
|  |  | YES (1) | NO /NR(0) |
| 1. Inclusion/exclusion criteria | Were the inclusion and exclusion criteria for study participation reported? (eg. Age greater than 50 years, no history of heart disease)? | 1 |  |
| 2. Group allocation | Was the study described as randomized? |  | 0 |
|  | Was the randomization method reported? |  | 0 |
|  | Was the randomization appropriate? |  | 0 |
|  | Was the allocation concealed? |  | 0 |
| 3. Blinding | Were the study subjects blinded to the intervention received? |  | 0 (NR) |
|  | Were the researcher personnel blinded to the intervention received by the subjects? |  | 0 (NR) |
| 4. Attrition | Were attrition numerically reported? | 1 |  |
|  | Were the reasons for withdrawals and dropouts provided? | 1 |  |
| 5. Exposure/intervention | Was the type of food described (eg. Composition, matrix)? | 1 |  |
|  | Was the amount of food described (i.e. dose)? | 1 |  |
| 6. Health effect | Was the methodology used to measure the health effect reported? | 1 |  |
| 7. Statistical analysis | Was between group statistical analysis of the health effect reported? | 1 |  |
|  | Was an intention-to-treat analysis conducted? |  | 0 |
| 8. Potential confounders | Were potential confounders of the food health relationship considered? |  | 0 |
| TOTAL SCORE (maximum of 15): | | 7/15 |  |
| Higher quality (Score ≥ 8) | |  | |
| Lower quality (Score ≤ 7) | | X | |

Confounders deemed to not be considered as differences in dietary intake and baseline lipid levels were not controlled for in the analysis

| **Quality Appraisal Tool for Experimental Studies** | | | |
| --- | --- | --- | --- |
| **Study reference:** Olmedilla-Alonso et al. (2008) | | | |
| **Item** | **Question** | **Score** | |
|  |  | YES (1) | NO /NR(0) |
| 1. Inclusion/exclusion criteria | Were the inclusion and exclusion criteria for study participation reported? (eg. Age greater than 50 years, no history of heart disease)? | 1 |  |
| 2. Group allocation | Was the study described as randomized? | 1 |  |
|  | Was the randomization method reported? |  | 0 (NR) |
|  | Was the randomization appropriate? |  | 0 (NR) |
|  | Was the allocation concealed? |  | 0 (NR) |
| 3. Blinding | Were the study subjects blinded to the intervention received? |  | 0 |
|  | Were the researcher personnel blinded to the intervention received by the subjects? |  | 0 (NR) |
| 4. Attrition | Were attrition numerically reported? | 1 |  |
|  | Were the reasons for withdrawals and dropouts provided? | 1 |  |
| 5. Exposure/intervention | Was the type of food described (eg. Composition, matrix)? | 1 |  |
|  | Was the amount of food described (i.e. dose)? | 1 |  |
| 6. Health effect | Was the methodology used to measure the health effect reported? | 1 |  |
| 7. Statistical analysis | Was between group statistical analysis of the health effect reported? | 1 |  |
|  | Was an intention-to-treat analysis conducted? | 1 |  |
| 8. Potential confounders | Were potential confounders of the food health relationship considered? |  | 0 |
| TOTAL SCORE (maximum of 15): | | 9/15 |  |
| Higher quality (Score ≥ 8) | | X | |
| Lower quality (Score ≤ 7) | |  | |

Confounders: Assessed background diet (participants recommended to continue normal diet), but did not report intake or compliance

| **Quality Appraisal Tool for Experimental Studies** | | | |
| --- | --- | --- | --- |
| **Study reference: Orem et al. (2013)** | | | |
| **Item** | **Question** | **Score** | |
|  |  | YES (1) | NO /NR(0) |
| 1. Inclusion/exclusion criteria | Were the inclusion and exclusion criteria for study participation reported? (eg. Age greater than 50 years, no history of heart disease)? | 1 |  |
| 2. Group allocation | Was the study described as randomized? |  | 0 |
|  | Was the randomization method reported? |  | 0 NR |
|  | Was the randomization appropriate? |  | 0 NR |
|  | Was the allocation concealed? |  | 0 NR |
| 3. Blinding | Were the study subjects blinded to the intervention received? |  | 0 |
|  | Were the researcher personnel blinded to the intervention received by the subjects? |  | 0 |
| 4. Attrition | Were attrition numerically reported? |  | 0 |
|  | Were the reasons for withdrawals and dropouts provided? |  | 0 |
| 5. Exposure/intervention | Was the type of food described (eg. Composition, matrix)? | 1 |  |
|  | Was the amount of food described (i.e. dose)? | 1 |  |
| 6. Health effect | Was the methodology used to measure the health effect reported? | 1 |  |
| 7. Statistical analysis | Was between group statistical analysis of the health effect reported? | 1 |  |
|  | Was an intention-to-treat analysis conducted? |  | 0 |
| 8. Potential confounders | Were potential confounders of the food health relationship considered? | 1 |  |
| TOTAL SCORE (maximum of 15): | | 6/15 |  |
| Higher quality (Score ≥ 8) | |  | |
| Lower quality (Score ≤ 7) | | X | |

Confounders: Dietary intake data collected, no difference in kJ intake over time. Study design less rigorous than if had been randomised, however inclusion of two control periods does somewhat address this confounder

| **Quality Quality Appraisal Tool for Experimental Studies** | | | |
| --- | --- | --- | --- |
| **Reference (Author, year):**   Palacios et al. (2020) | |  | |
| **Item** | **Question** | **Score** | |
|  |  | YES (1) | NO /NR(0) |
| 1. Inclusion/ Exclusion Criteria | Were the inclusion and/or exclusion criteria for study participation reported (*e.g.*, age greater than 50 years, no history of heart disease)? | 1 |  |
| 2. Group Allocation | Was the study described as randomized? | 1 |  |
|  | Was the randomization method reported? |  | 0 (NR) |
|  | Was the randomization method appropriate? |  | 0 (NR) |
|  | Was allocation concealed? |  | 0 (NR) |
| 3. Blinding | Were the study subjects blinded to the intervention received? |  | 0 (NR) |
|  | Were the research personnel blinded to the intervention received by the subjects? |  | 0 (NR) |
| 4. Attrition | Was attrition numerically reported? | 1 |  |
|  | Were the reasons for withdrawals and dropouts provided? | 1 |  |
| 5. Exposure/  Intervention | Was the type of food described (e.g., composition, matrix)? | 1 |  |
|  | Was the amount of food described (i.e., dose)? | 1 |  |
| 6. Health Effect | Was the methodology used to measure the health effect reported? | 1 |  |
| 7. Statistical Analysis | Was a between-group statistical analysis of the health effect conducted (*i.e.*, control vs. intervention)? | 1 |  |
|  | Was an intention-to-treat analysis conducted? |  | 0 |
| 8. Potential Confounders | Were potential confounders of the food/health relationship considered? | 1 |  |
| TOTAL SCORE (maximum of 15): | | 9/15 | |
| Higher quality (Score ≥ 8) | | X | |
| Lower quality (Score ≤ 7) | |  | |

Confounders: not applicable as randomised cross-over design

| **Quality Appraisal Tool for Experimental Studies** | | | |
| --- | --- | --- | --- |
| **Study reference: Parham et al (2014)** | | | |
| **Item** | **Question** | **Score** | |
|  |  | YES (1) | NO /NR(0) |
| 1. Inclusion/exclusion criteria | Were the inclusion and exclusion criteria for study participation reported? (eg. Age greater than 50 years, no history of heart disease)? | 1 |  |
| 2. Group allocation | Was the study described as randomized? | 1 |  |
|  | Was the randomization method reported? | 1 |  |
|  | Was the randomization appropriate? | 1 |  |
|  | Was the allocation concealed? | 1 |  |
| 3. Blinding | Were the study subjects blinded to the intervention received? | 1 |  |
|  | Were the researcher personnel blinded to the intervention received by the subjects? |  | 0 |
| 4. Attrition | Were attrition numerically reported? | 1 |  |
|  | Were the reasons for withdrawals and dropouts provided? |  | 0 |
| 5. Exposure/intervention | Was the type of food described (e.g.. Composition, matrix)? | 1 |  |
|  | Was the amount of food described (i.e. dose)? | 1 |  |
| 6. Health effect | Was the methodology used to measure the health effect reported? | 1 |  |
| 7. Statistical analysis | Was between group statistical analysis of the health effect reported? | 1 |  |
|  | Was an intention-to-treat analysis conducted? |  | 0 |
| 8. Potential confounders | Were potential confounders of the food health relationship considered? |  | 0 |
| TOTAL SCORE (maximum of 15): | | 11/15 |  |
| Higher quality (Score ≥ 8) | | X | |
| Lower quality (Score ≤ 7) | |  | |

Confounders: Intake of medication of participants were not assessed. Dietary intakes of participants were also not reported.

| **Quality Appraisal Tool for Experimental Studies** | | | |
| --- | --- | --- | --- |
| **Study reference: Pelkman et al. (2004)** | | | |
| **Item** | **Question** | **Score** | |
|  |  | YES (1) | NO /NR(0) |
| 1. Inclusion/exclusion criteria | Were the inclusion and exclusion criteria for study participation reported? (eg. Age greater than 50 years, no history of heart disease)? | 1 |  |
| 2. Group allocation | Was the study described as randomized? | 1 |  |
|  | Was the randomization method reported? |  | 0 (NR) |
|  | Was the randomization appropriate? |  | 0 (NR) |
|  | Was the allocation concealed? |  | 0 (NR) |
| 3. Blinding | Were the study subjects blinded to the intervention received? |  | 0 (NR) |
|  | Were the researcher personnel blinded to the intervention received by the subjects? |  | 0 (NR) |
| 4. Attrition | Were attrition numerically reported? | 1 |  |
|  | Were the reasons for withdrawals and dropouts provided? | 1 |  |
| 5. Exposure/intervention | Was the type of food described (eg. Composition, matrix)? | 1 |  |
|  | Was the amount of food described (i.e. dose)? |  | 0 (NR) |
| 6. Health effect | Was the methodology used to measure the health effect reported? | 1 |  |
| 7. Statistical analysis | Was between group statistical analysis of the health effect reported? |  | 0 |
|  | Was an intention-to-treat analysis conducted? |  | 0^1^ |
| 8. Potential confounders | Were potential confounders of the food health relationship considered? | 1 |  |
| TOTAL SCORE (maximum of 15): | | 7/15 |  |
| Higher quality (Score ≥ 8) | |  | |
| Lower quality (Score ≤ 7) | | X | |

^1^Not conducted for cardiovascular outcomes (primary outcomes)

Confounders: Added week one values as a covariate in each model to verify that baseline differences between groups did not alter results, compared lipid levels at baseline

| **Quality Appraisal Tool for Experimental Studies** | | | |
| --- | --- | --- | --- |
| **Study reference: Rajaram et al. (2001)** | | | |
| **Item** | **Question** | **Score** | |
|  |  | YES (1) | NO /NR(0) |
| 1. Inclusion/exclusion criteria | Were the inclusion and exclusion criteria for study participation reported? (eg. Age greater than 50 years, no history of heart disease)? | 1 |  |
| 2. Group allocation | Was the study described as randomized? | 1 |  |
|  | Was the randomization method reported? |  | 0 (NR) |
|  | Was the randomization appropriate? |  | 0 (NR) |
|  | Was the allocation concealed? |  | 0 (NR) |
| 3. Blinding | Were the study subjects blinded to the intervention received? | 1 |  |
|  | Were the researcher personnel blinded to the intervention received by the subjects? |  | 0 (NR) |
| 4. Attrition | Were attrition numerically reported? | 1 |  |
|  | Were the reasons for withdrawals and dropouts provided? |  | 0 (NR) |
| 5. Exposure/intervention | Was the type of food described (eg. Composition, matrix)? | 1 |  |
|  | Was the amount of food described (i.e. dose)? | 1 |  |
| 6. Health effect | Was the methodology used to measure the health effect reported? | 1 |  |
| 7. Statistical analysis | Was between group statistical analysis of the health effect reported? | 1 |  |
|  | Was an intention-to-treat analysis conducted? |  | 0 |
| 8. Potential confounders | Were potential confounders of the food health relationship considered? | 1 |  |
| TOTAL SCORE (maximum of 15): | | 9/15 |  |
| Higher quality (Score ≥ 8) | | X | |
| Lower quality (Score ≤ 7) | |  | |

Confounders: Baseline values and gender were used as covariates in analyses. Period and carry-over effects were evaluated (no carry over effect between diet periods found)

| **Quality Appraisal Tool for Experimental Studies** | | | |
| --- | --- | --- | --- |
| **Study reference: Rajaram et al. (2009)** | | | |
| **Item** | **Question** | **Score** | |
|  |  | YES (1) | NO /NR(0) |
| 1. Inclusion/exclusion criteria | Were the inclusion and exclusion criteria for study participation reported? (eg. Age greater than 50 years, no history of heart disease)? | 1 |  |
| 2. Group allocation | Was the study described as randomized? | 1 |  |
|  | Was the randomization method reported? |  | 0 (NR) |
|  | Was the randomization appropriate? |  | 0 (NR) |
|  | Was the allocation concealed? |  | 0 (NR) |
| 3. Blinding | Were the study subjects blinded to the intervention received? |  | 0 (NR) |
|  | Were the researcher personnel blinded to the intervention received by the subjects? |  | 0 (NR) |
| 4. Attrition | Were attrition numerically reported? | 1 |  |
|  | Were the reasons for withdrawals and dropouts provided? | 1 |  |
| 5. Exposure/intervention | Was the type of food described (eg. Composition, matrix)? | 1 |  |
|  | Was the amount of food described (i.e. dose)? | 1 |  |
| 6. Health effect | Was the methodology used to measure the health effect reported? | 1 |  |
| 7. Statistical analysis | Was between group statistical analysis of the health effect reported? | 1 |  |
|  | Was an intention-to-treat analysis conducted? |  | 0 |
| 8. Potential confounders | Were potential confounders of the food health relationship considered? | 1 |  |
| TOTAL SCORE (maximum of 15): | | 9/15 |  |
| Higher quality (Score ≥ 8) | | X | |
| Lower quality (Score ≤ 7) | |  | |

Confounders: Each group was stratified according to age, gender and baseline serum cholesterol. Fixed terms included in analysis: diet, treatment period, clinic day, period-by-clinic-day interaction. Interaction between diet and baseline lipids also assessed

| **Quality Appraisal Tool for Experimental Studies** | | | |
| --- | --- | --- | --- |
| **Study reference: Rajaram et al 2010/ Sabate et al. (2003)** | | | |
| **Item** | **Question** | **Score** | |
|  |  | YES (1) | NO /NR(0) |
| 1. Inclusion/exclusion criteria | Were the inclusion and exclusion criteria for study participation reported? (eg. Age greater than 50 years, no history of heart disease)? | 1 |  |
| 2. Group allocation | Was the study described as randomized? | 1 |  |
|  | Was the randomization method reported? |  | 0 (NR) |
|  | Was the randomization appropriate? |  | 0 (NR) |
|  | Was the allocation concealed? |  | 0 (NR) |
| 3. Blinding | Were the study subjects blinded to the intervention received? |  | 0 |
|  | Were the researcher personnel blinded to the intervention received by the subjects? |  | 0 |
| 4. Attrition | Were attrition numerically reported? | 1 |  |
|  | Were the reasons for withdrawals and dropouts provided? | 1 |  |
| 5. Exposure/intervention | Was the type of food described (eg. Composition, matrix)? | 1 |  |
|  | Was the amount of food described (i.e. dose)? | 1 |  |
| 6. Health effect | Was the methodology used to measure the health effect reported? | 1 |  |
| 7. Statistical analysis | Was between group statistical analysis of the health effect reported? | 1 |  |
|  | Was an intention-to-treat analysis conducted? |  | 0 |
| 8. Potential confounders | Were potential confounders of the food health relationship considered? | 1 |  |
| TOTAL SCORE (maximum of 15): | | 9/15 |  |
| Higher quality (Score ≥ 8) | | X | |
| Lower quality (Score ≤ 7) | |  | |

Confounders: Diets were isoenergetic, and the dietary profile of diets was compared. Study authors also justified why wash-out was not required.

| **Quality Appraisal Tool for Experimental Studies** | | | |
| --- | --- | --- | --- |
| **Study reference:** Rock et al (2017) | | | |
| **Item** | **Question** | **Score** | |
|  |  | YES (1) | NO /NR(0) |
| 1. Inclusion/exclusion criteria | Were the inclusion and exclusion criteria for study participation reported? (eg. Age greater than 50 years, no history of heart disease)? | 1 |  |
| 2. Group allocation | Was the study described as randomized? | 1 |  |
|  | Was the randomization method reported? | 1 |  |
|  | Was the randomization appropriate? | 1 |  |
|  | Was the allocation concealed? | 1 |  |
| 3. Blinding | Were the study subjects blinded to the intervention received? |  | 0 |
|  | Were the researcher personnel blinded to the intervention received by the subjects? | 1 |  |
| 4. Attrition | Were attrition numerically reported? | 1 |  |
|  | Were the reasons for withdrawals and dropouts provided? |  | 0 |
| 5. Exposure/intervention | Was the type of food described (e.g.. Composition, matrix)? | 1 |  |
|  | Was the amount of food described (i.e. dose)? | 1 |  |
| 6. Health effect | Was the methodology used to measure the health effect reported? | 1 |  |
| 7. Statistical analysis | Was between group statistical analysis of the health effect reported? | 1 |  |
|  | Was an intention-to-treat analysis conducted? |  | 0 |
| 8. Potential confounders | Were potential confounders of the food health relationship considered? | 1 |  |
| TOTAL SCORE (maximum of 15): | | 12/15 |  |
| Higher quality (Score ≥ 8) | | X | |
| Lower quality (Score ≤ 7) | |  | |

Confounders: The adherence of dietary intervention was assessed. Physical activity during the study was assessed and reported.

| **Quality Appraisal Tool for Experimental Studies** | | | |
| --- | --- | --- | --- |
| **Study reference:** Rock et al (2020) | |  | |
| **Item** | **Question** | **Score** | |
|  |  | YES (1) | NO /NR(0) |
| 1. Inclusion/ Exclusion Criteria | Were the inclusion and/or exclusion criteria for study participation reported (*e.g.*, age greater than 50 years, no history of heart disease)? | 1 |  |
| 2. Group Allocation | Was the study described as randomized? | 1 |  |
|  | Was the randomization method reported? |  | 0 (NR) |
|  | Was the randomization method appropriate? |  | 0 (NR) |
|  | Was allocation concealed? |  | 0 (NR) |
| 3. Blinding | Were the study subjects blinded to the intervention received? |  | 0 (NR) |
|  | Were the research personnel blinded to the intervention received by the subjects? |  | 0 (NR) |
| 4. Attrition | Was attrition numerically reported? | 1 |  |
|  | Were the reasons for withdrawals and dropouts provided? | 1 |  |
| 5. Exposure/  Intervention | Was the type of food described (e.g., composition, matrix)? | 1 |  |
|  | Was the amount of food described (i.e., dose)? | 1 |  |
| 6. Health Effect | Was the methodology used to measure the health effect reported? | 1 |  |
| 7. Statistical Analysis | Was a between-group statistical analysis of the health effect conducted (*i.e.*, control vs. intervention)? | 1 |  |
|  | Was an intention-to-treat analysis conducted? |  | 0 |
| 8. Potential Confounders | Were potential confounders of the food/health relationship considered? | 1 |  |
| TOTAL SCORE (maximum of 15): | | 9/15 | |
| Higher quality (Score ≥ 8) | | X | |
| Lower quality (Score ≤ 7) | |  | |

 Confounders: Physical activity collected

| **Quality Appraisal Tool for Experimental Studies** | | | |
| --- | --- | --- | --- |
| **Study reference: Ros et al. (2004)** | | | |
| **Item** | **Question** | **Score** | |
|  |  | YES (1) | NO /NR(0) |
| 1. Inclusion/exclusion criteria | Were the inclusion and exclusion criteria for study participation reported? (eg. Age greater than 50 years, no history of heart disease)? | 1 |  |
| 2. Group allocation | Was the study described as randomized? | 1 |  |
|  | Was the randomization method reported? |  | 0 (NR) |
|  | Was the randomization appropriate? |  | 0 (NR) |
|  | Was the allocation concealed? |  | 0 (NR) |
| 3. Blinding | Were the study subjects blinded to the intervention received? |  | 0 (NR) |
|  | Were the researcher personnel blinded to the intervention received by the subjects? |  | 0 (NR) |
| 4. Attrition | Were attrition numerically reported? | 1 |  |
|  | Were the reasons for withdrawals and dropouts provided? | 1 |  |
| 5. Exposure/intervention | Was the type of food described (eg. Composition, matrix)? | 1 |  |
|  | Was the amount of food described (i.e. dose)? | 1 |  |
| 6. Health effect | Was the methodology used to measure the health effect reported? | 1 |  |
| 7. Statistical analysis | Was between group statistical analysis of the health effect reported? | 1 |  |
|  | Was an intention-to-treat analysis conducted? |  | 0 |
| 8. Potential confounders | Were potential confounders of the food health relationship considered? | 1 |  |
| TOTAL SCORE (maximum of 15): | | 9/15 |  |
| Higher quality (Score ≥ 8) | | X | |
| Lower quality (Score ≤ 7) | |  | |

Confounders: Data was assessed for a carry-over effect (none found). Baseline values and sex were used as covariates in analyses.

| **Quality Appraisal Tool for Experimental Studies** | | | |
| --- | --- | --- | --- |
| **Study reference:** **Ruisinger et al (2015)** | | | |
| **Item** | **Question** | **Score** | |
|  |  | YES (1) | NO /NR(0) |
| 1. Inclusion/exclusion criteria | Were the inclusion and exclusion criteria for study participation reported? (eg. Age greater than 50 years, no history of heart disease)? | 1 |  |
| 2. Group allocation | Was the study described as randomized? | 1 |  |
|  | Was the randomization method reported? |  | 0 (NR) |
|  | Was the randomization appropriate? |  | 0 (NR) |
|  | Was the allocation concealed? |  | 0 (NR) |
| 3. Blinding | Were the study subjects blinded to the intervention received? |  | 0 (NR) |
|  | Were the researcher personnel blinded to the intervention received by the subjects? |  | 0 (NR) |
| 4. Attrition | Were attrition numerically reported? | 1 |  |
|  | Were the reasons for withdrawals and dropouts provided? | 1 |  |
| 5. Exposure/intervention | Was the type of food described (e.g.. Composition, matrix)? | 1 |  |
|  | Was the amount of food described (i.e. dose)? | 1 |  |
| 6. Health effect | Was the methodology used to measure the health effect reported? | 1 |  |
| 7. Statistical analysis | Was between group statistical analysis of the health effect reported? | 1 |  |
|  | Was an intention-to-treat analysis conducted? |  | 0 |
| 8. Potential confounders | Were potential confounders of the food health relationship considered? |  | 0 |
| TOTAL SCORE (maximum of 15): | | 8/15 |  |
| Higher quality (Score ≥ 8) | | X | |
| Lower quality (Score ≤ 7) | |  | |

Confounders: There were significant differences in energy intake and vegetable protein intake between groups at post-treatment. The physical activity level of control groups was decreased by 139kcal/week during the study, but the intervention group increased physical activity by 140 kcal/week.

| **Quality Appraisal Tool for Experimental Studies** | | | |
| --- | --- | --- | --- |
| **Study reference:** Sabate et al. (1993) | | | |
| **Item** | **Question** | **Score** | |
|  |  | YES (1) | NO /NR(0) |
| 1. Inclusion/exclusion criteria | Were the inclusion and exclusion criteria for study participation reported? (eg. Age greater than 50 years, no history of heart disease)? | 1 |  |
| 2. Group allocation | Was the study described as randomized? | 1 |  |
|  | Was the randomization method reported? |  | 0 (NR) |
|  | Was the randomization appropriate? |  | 0 (NR) |
|  | Was the allocation concealed? |  | 0 (NR) |
| 3. Blinding | Were the study subjects blinded to the intervention received? |  | 0 (NR) |
|  | Were the researcher personnel blinded to the intervention received by the subjects? | 1 |  |
| 4. Attrition | Were attrition numerically reported? | 1 |  |
|  | Were the reasons for withdrawals and dropouts provided? | 1 |  |
| 5. Exposure/intervention | Was the type of food described (eg. Composition, matrix)? | 1 |  |
|  | Was the amount of food described (i.e. dose)? | 1 |  |
| 6. Health effect | Was the methodology used to measure the health effect reported? | 1 |  |
| 7. Statistical analysis | Was between group statistical analysis of the health effect reported? | 1 |  |
|  | Was an intention-to-treat analysis conducted? |  | 0 |
| 8. Potential confounders | Were potential confounders of the food health relationship considered? | 1 |  |
| TOTAL SCORE (maximum of 15): | | 10/15 |  |
| Higher quality (Score ≥ 8) | | X | |
| Lower quality (Score ≤ 7) | |  | |

Confounders: Results of all measurements made during alternate days at the end of each dietary period were averaged. Randomisation was stratified based on age, baseline serum cholesterol level and BMI. Carry-over effect was assessed (no evidence of an effect)

| **Quality Appraisal Tool for Experimental Studies** | | | |
| --- | --- | --- | --- |
| **Study reference: Sabate et al. (2005)/Torabian et al. (2010)** | | | |
| **Item** | **Question** | **Score** | |
|  |  | YES (1) | NO /NR(0) |
| 1. Inclusion/exclusion criteria | Were the inclusion and exclusion criteria for study participation reported? (eg. Age greater than 50 years, no history of heart disease)? | 1 |  |
| 2. Group allocation | Was the study described as randomized? | 1 |  |
|  | Was the randomization method reported? |  | 0 (NR) |
|  | Was the randomization appropriate? |  | 0 (NR) |
|  | Was the allocation concealed? |  | 0 (NR) |
| 3. Blinding | Were the study subjects blinded to the intervention received? |  | 0 |
|  | Were the researcher personnel blinded to the intervention received by the subjects? |  | 0 |
| 4. Attrition | Were attrition numerically reported? | 1 |  |
|  | Were the reasons for withdrawals and dropouts provided? | 1 |  |
| 5. Exposure/intervention | Was the type of food described (eg. Composition, matrix)? | 1 |  |
|  | Was the amount of food described (i.e. dose)? | 1 |  |
| 6. Health effect | Was the methodology used to measure the health effect reported? | 1 |  |
| 7. Statistical analysis | Was between group statistical analysis of the health effect reported? | 1 |  |
|  | Was an intention-to-treat analysis conducted? |  | 0 |
| 8. Potential confounders | Were potential confounders of the food health relationship considered? | 1 |  |
| TOTAL SCORE (maximum of 15): | | 9/15 |  |
| Higher quality (Score ≥ 8) | | X | |
| Lower quality (Score ≤ 7) | |  | |

Confounders: Physical activity assessed. No significant difference in baseline variables between group randomised to start on walnut diet, or control diet. Anthropometric analysis took into account differences in energy intake between groups. Lipid analysis adjusted for body weight

| **Quality Appraisal Tool for Experimental Studies** | | | |
| --- | --- | --- | --- |
| **Study reference:** Salas-Huetos et al (2018) | |  | |
| **Item** | **Question** | **Score** | |
|  |  | YES (1) | NO /NR(0) |
| 1. Inclusion/ Exclusion Criteria | Were the inclusion and/or exclusion criteria for study participation reported (*e.g.*, age greater than 50 years, no history of heart disease)? | 1 |  |
| 2. Group Allocation | Was the study described as randomized? | 1 |  |
|  | Was the randomization method reported? | 1 |  |
|  | Was the randomization method appropriate? | 1 |  |
|  | Was allocation concealed? |  | 0 (NR) |
| 3. Blinding | Were the study subjects blinded to the intervention received? | 1 |  |
|  | Were the research personnel blinded to the intervention received by the subjects? | 1 |  |
| 4. Attrition | Was attrition numerically reported? | 1 |  |
|  | Were the reasons for withdrawals and dropouts provided? | 1 |  |
| 5. Exposure/  Intervention | Was the type of food described (e.g., composition, matrix)? | 1 |  |
|  | Was the amount of food described (i.e., dose)? | 1 |  |
| 6. Health Effect | Was the methodology used to measure the health effect reported? | 1 |  |
| 7. Statistical Analysis | Was a between-group statistical analysis of the health effect conducted (*i.e.*, control vs. intervention)? | 1 |  |
|  | Was an intention-to-treat analysis conducted? | 1 |  |
| 8. Potential Confounders | Were potential confounders of the food/health relationship considered? |  | 0 |
| TOTAL SCORE (maximum of 15): | | 13/15 | |
| Higher quality (Score ≥ 8) | | X | |
| Lower quality (Score ≤ 7) | |  | |

Confounders: changes in physical activity not reported

| **Quality Appraisal Tool for Experimental Studies** | | | |
| --- | --- | --- | --- |
| **Study reference: Sales et al. (2008)/** **Akuamoah-Boateng et al. (2007)** | | | |
| **Item** | **Question** | **Score** | |
|  |  | YES (1) | NO /NR(0) |
| 1. Inclusion/exclusion criteria | Were the inclusion and exclusion criteria for study participation reported? (eg. Age greater than 50 years, no history of heart disease)? | 1 |  |
| 2. Group allocation | Was the study described as randomized? | 1 |  |
|  | Was the randomization method reported? |  | 0 (NR) |
|  | Was the randomization appropriate? |  | 0 (NR) |
|  | Was the allocation concealed? |  | 0 (NR) |
| 3. Blinding | Were the study subjects blinded to the intervention received? | 1 |  |
|  | Were the researcher personnel blinded to the intervention received by the subjects? |  | 0 (NR) |
| 4. Attrition | Were attrition numerically reported? |  | 0 (NR) |
|  | Were the reasons for withdrawals and dropouts provided? |  | 0 (NR) |
| 5. Exposure/intervention | Was the type of food described (eg. Composition, matrix)? | 1 |  |
|  | Was the amount of food described (i.e. dose)? |  | 0 (NR) |
| 6. Health effect | Was the methodology used to measure the health effect reported? | 1 |  |
| 7. Statistical analysis | Was between group statistical analysis of the health effect reported? |  | 0^1^ |
|  | Was an intention-to-treat analysis conducted? |  | 0 (NR) |
| 8. Potential confounders | Were potential confounders of the food health relationship considered? |  | 0 |
| TOTAL SCORE (maximum of 15): | | 5/15 |  |
| Higher quality (Score ≥ 8) | |  | |
| Lower quality (Score ≤ 7) | | X | |

^1^Not clear if conducted for cardiovascular outcomes (primary outcomes)

Confounders: Did not adjust for control group consuming substantially lower energy intakes over study

| **Quality Appraisal Tool for Experimental Studies** | | | |
| --- | --- | --- | --- |
| **Study reference:** Sapp et al. (2021) | |  | |
| **Item** | **Question** | **Score** | |
|  |  | YES (1) | NO /NR(0) |
| 1. Inclusion/ Exclusion Criteria | Were the inclusion and/or exclusion criteria for study participation reported (*e.g.*, age greater than 50 years, no history of heart disease)? | 1 |  |
| 2. Group Allocation | Was the study described as randomized? | 1 |  |
|  | Was the randomization method reported? | 1 |  |
|  | Was the randomization method appropriate? | 1 |  |
|  | Was allocation concealed? | 1 |  |
| 3. Blinding | Were the study subjects blinded to the intervention received? |  | 0 |
|  | Were the research personnel blinded to the intervention received by the subjects? | 1 |  |
| 4. Attrition | Was attrition numerically reported? | 1 |  |
|  | Were the reasons for withdrawals and dropouts provided? | 1 |  |
| 5. Exposure/  Intervention | Was the type of food described (e.g., composition, matrix)? | 1 |  |
|  | Was the amount of food described (i.e., dose)? | 1 |  |
| 6. Health Effect | Was the methodology used to measure the health effect reported? | 1 |  |
| 7. Statistical Analysis | Was a between-group statistical analysis of the health effect conducted (*i.e.*, control vs. intervention)? | 1 |  |
|  | Was an intention-to-treat analysis conducted? | 1 |  |
| 8. Potential Confounders | Were potential confounders of the food/health relationship considered? | 1 |  |
| TOTAL SCORE (maximum of 15): | | 14/15 | |
| Higher quality (Score ≥ 8) | | X | |
| Lower quality (Score ≤ 7) | |  | |

Confounders: adherence and changes to lifestyle reported

| **Quality Appraisal Tool for Experimental Studies** | | | |
| --- | --- | --- | --- |
| **Study reference: Sauder et al (2014)/(2015)** | | | |
| **Item** | **Question** | **Score** | |
|  |  | YES (1) | NO /NR(0) |
| 1. Inclusion/exclusion criteria | Were the inclusion and exclusion criteria for study participation reported? (eg. Age greater than 50 years, no history of heart disease)? | 1 |  |
| 2. Group allocation | Was the study described as randomized? | 1 |  |
|  | Was the randomization method reported? | 1 |  |
|  | Was the randomization appropriate? | 1 |  |
|  | Was the allocation concealed? | 1 |  |
| 3. Blinding | Were the study subjects blinded to the intervention received? |  | 0 |
|  | Were the researcher personnel blinded to the intervention received by the subjects? | 1 |  |
| 4. Attrition | Were attrition numerically reported? | 1 |  |
|  | Were the reasons for withdrawals and dropouts provided? | 1 |  |
| 5. Exposure/intervention | Was the type of food described (e.g.. Composition, matrix)? | 1 |  |
|  | Was the amount of food described (i.e. dose)? | 1 |  |
| 6. Health effect | Was the methodology used to measure the health effect reported? | 1 |  |
| 7. Statistical analysis | Was between group statistical analysis of the health effect reported? | 1 |  |
|  | Was an intention-to-treat analysis conducted? |  | 0 |
| 8. Potential confounders | Were potential confounders of the food health relationship considered? | 1 |  |
| TOTAL SCORE (maximum of 15): | | 13/15 |  |
| Higher quality (Score ≥ 8) | | X | |
| Lower quality (Score ≤ 7) | |  | |

Confounders: The consumed foods during the study were provided by the study.

| **Quality Appraisal Tool for Experimental Studies** | | | |
| --- | --- | --- | --- |
| **Study reference: Sheridan et al. (2007)** | | | |
| **Item** | **Question** | **Score** | |
|  |  | YES (1) | NO /NR(0) |
| 1. Inclusion/exclusion criteria | Were the inclusion and exclusion criteria for study participation reported? (eg. Age greater than 50 years, no history of heart disease)? | 1 |  |
| 2. Group allocation | Was the study described as randomized? | 1 |  |
|  | Was the randomization method reported? |  | 0 (NR) |
|  | Was the randomization appropriate? |  | 0 (NR) |
|  | Was the allocation concealed? |  | 0 (NR) |
| 3. Blinding | Were the study subjects blinded to the intervention received? |  | 0 (NR) |
|  | Were the researcher personnel blinded to the intervention received by the subjects? | 1 |  |
| 4. Attrition | Were attrition numerically reported? | 1 |  |
|  | Were the reasons for withdrawals and dropouts provided? | 1 |  |
| 5. Exposure/intervention | Was the type of food described (eg. Composition, matrix)? | 1 |  |
|  | Was the amount of food described (i.e. dose)? | 1 |  |
| 6. Health effect | Was the methodology used to measure the health effect reported? | 1 |  |
| 7. Statistical analysis | Was between group statistical analysis of the health effect reported? | 1 |  |
|  | Was an intention-to-treat analysis conducted? |  | 0 |
| 8. Potential confounders | Were potential confounders of the food health relationship considered? | 1 |  |
| TOTAL SCORE (maximum of 15): | | 10/15 |  |
| Higher quality (Score ≥ 8) | | X | |
| Lower quality (Score ≤ 7) | |  | |

Confounders: Assessed for difference in effect based on sequence (none found). Measured dietary intake, no difference in total energy or macronutrients found between periods

| **Quality Appraisal Tool for Experimental Studies** | | | |
| --- | --- | --- | --- |
| **Study reference: Solá et al. (2012)** | | | |
| **Item** | **Question** | **Score** | |
|  |  | YES (1) | NO /NR(0) |
| 1. Inclusion/exclusion criteria | Were the inclusion and exclusion criteria for study participation reported? (eg. Age greater than 50 years, no history of heart disease)? | 1 |  |
| 2. Group allocation | Was the study described as randomized? | 1 |  |
|  | Was the randomization method reported? | 1 |  |
|  | Was the randomization appropriate? | 1 |  |
|  | Was the allocation concealed? | 1 |  |
| 3. Blinding | Were the study subjects blinded to the intervention received? | 1 |  |
|  | Were the researcher personnel blinded to the intervention received by the subjects? | 1 |  |
| 4. Attrition | Were attrition numerically reported? | 1 |  |
|  | Were the reasons for withdrawals and dropouts provided? | 1 |  |
| 5. Exposure/intervention | Was the type of food described (eg. Composition, matrix)? | 1 |  |
|  | Was the amount of food described (i.e. dose)? | 1 |  |
| 6. Health effect | Was the methodology used to measure the health effect reported? | 1 |  |
| 7. Statistical analysis | Was between group statistical analysis of the health effect reported? | 1 |  |
|  | Was an intention-to-treat analysis conducted? | 1 |  |
| 8. Potential confounders | Were potential confounders of the food health relationship considered? | 1 |  |
| TOTAL SCORE (maximum of 15): | | 15/15 |  |
| Higher quality (Score ≥ 8) | | X | |
| Lower quality (Score ≤ 7) | |  | |

Confounders considered included baseline values in statistical analysis and measurement of diet (no differences in energy between groups). Some differences between groups in age and family history of CVD at baseline, but overall groups similar. Randomisation was stratified by gender.

| **Quality Appraisal Tool for Experimental Studies** | | | |
| --- | --- | --- | --- |
| **Study reference: Somerset et al. (2013)** | | | |
| **Item** | **Question** | **Score** | |
|  |  | YES (1) | NO /NR(0) |
| 1. Inclusion/exclusion criteria | Were the inclusion and exclusion criteria for study participation reported? (eg. Age greater than 50 years, no history of heart disease)? | 1 |  |
| 2. Group allocation | Was the study described as randomized? | 1 |  |
|  | Was the randomization method reported? |  | 0 (NR) |
|  | Was the randomization appropriate? |  | 0 (NR) |
|  | Was the allocation concealed? |  | 0 (NR) |
| 3. Blinding | Were the study subjects blinded to the intervention received? |  | 0 (NR) |
|  | Were the researcher personnel blinded to the intervention received by the subjects? |  | 0 (NR) |
| 4. Attrition | Were attrition numerically reported? |  | 0 |
|  | Were the reasons for withdrawals and dropouts provided? |  | 0 |
| 5. Exposure/intervention | Was the type of food described (eg. Composition, matrix)? | 1 |  |
|  | Was the amount of food described (i.e. dose)? |  | 0 (NR) |
| 6. Health effect | Was the methodology used to measure the health effect reported? | 1 |  |
| 7. Statistical analysis | Was between group statistical analysis of the health effect reported? |  | 0 (NR) |
|  | Was an intention-to-treat analysis conducted? |  | 0 |
| 8. Potential confounders | Were potential confounders of the food health relationship considered? | 1 |  |
| TOTAL SCORE (maximum of 15): | | 5/15 |  |
| Higher quality (Score ≥ 8) | |  | |
| Lower quality (Score ≤ 7) | | X | |

Confounders: Physical activity monitored as potential confounder. No difference in dietary intake over time

| **Quality Appraisal Tool for Experimental Studies** | | | |
| --- | --- | --- | --- |
| **Study reference: Spaccarotella et al. (2008)** | | | |
| **Item** | **Question** | **Score** | |
|  |  | YES (1) | NO /NR(0) |
| 1. Inclusion/exclusion criteria | Were the inclusion and exclusion criteria for study participation reported? (eg. Age greater than 50 years, no history of heart disease)? | 1 |  |
| 2. Group allocation | Was the study described as randomized? | 1 |  |
|  | Was the randomization method reported? |  | 0 |
|  | Was the randomization appropriate? |  | 0 (NR) |
|  | Was the allocation concealed? |  | 0 (NR) |
| 3. Blinding | Were the study subjects blinded to the intervention received? |  | 0 (NR) |
|  | Were the researcher personnel blinded to the intervention received by the subjects? |  | 0 (NR) |
| 4. Attrition | Were attrition numerically reported? | 1 |  |
|  | Were the reasons for withdrawals and dropouts provided? | 1 |  |
| 5. Exposure/intervention | Was the type of food described (eg. Composition, matrix)? | 1 |  |
|  | Was the amount of food described (i.e. dose)? | 1 |  |
| 6. Health effect | Was the methodology used to measure the health effect reported? | 1 |  |
| 7. Statistical analysis | Was between group statistical analysis of the health effect reported? | 1 |  |
|  | Was an intention-to-treat analysis conducted? |  | 0 |
| 8. Potential confounders | Were potential confounders of the food health relationship considered? |  | 0 |
| TOTAL SCORE (maximum of 15): | | 8/15 |  |
| Higher quality (Score ≥ 8) | | X | |
| Lower quality (Score ≤ 7) | |  | |

Confounders: Analyses did consider sequence and period effects and the potential effect of weight change on outcomes; however participants consumed significantly greater energy intakes during walnut period, which was not adjusted for

| **Quality Appraisal Tool for Experimental Studies** | | | |
| --- | --- | --- | --- |
| **Study reference: Spiller et al. (1998)** | | | |
| **Item** | **Question** | **Score** | |
|  |  | YES (1) | NO /NR(0) |
| 1. Inclusion/exclusion criteria | Were the inclusion and exclusion criteria for study participation reported? (eg. Age greater than 50 years, no history of heart disease)? | 1 |  |
| 2. Group allocation | Was the study described as randomized? | 1 |  |
|  | Was the randomization method reported? |  | 0 (NR) |
|  | Was the randomization appropriate? |  | 0 (NR) |
|  | Was the allocation concealed? |  | 0 (NR) |
| 3. Blinding | Were the study subjects blinded to the intervention received? |  | 0 (NR) |
|  | Were the researcher personnel blinded to the intervention received by the subjects? |  | 0 |
| 4. Attrition | Were attrition numerically reported? | 1 |  |
|  | Were the reasons for withdrawals and dropouts provided? | 1 |  |
| 5. Exposure/intervention | Was the type of food described (eg. Composition, matrix)? | 1 |  |
|  | Was the amount of food described (i.e. dose)? | 1 |  |
| 6. Health effect | Was the methodology used to measure the health effect reported? | 1 |  |
| 7. Statistical analysis | Was between group statistical analysis of the health effect reported? | 1 |  |
|  | Was an intention-to-treat analysis conducted? |  | 0 |
| 8. Potential confounders | Were potential confounders of the food health relationship considered? |  | 0 |
| TOTAL SCORE (maximum of 15): | | 8/15 |  |
| Higher quality (Score ≥ 8) | | X | |
| Lower quality (Score ≤ 7) | |  | |

Confounders: Energy intake significantly higher in olive oil group (no adjustment used). Subjects were randomized based on fasting total cholesterol, however due to the confounding nature of energy intake, the study was deemed to have not adequately considered confounders.

| **Quality Appraisal Tool for Experimental Studies** | | | |
| --- | --- | --- | --- |
| **Study reference: Sweazea et al. (2014)** | | | |
| **Item** | **Question** | **Score** | |
|  |  | YES (1) | NO /NR(0) |
| 1. Inclusion/exclusion criteria | Were the inclusion and exclusion criteria for study participation reported? (eg. Age greater than 50 years, no history of heart disease)? | 1 |  |
| 2. Group allocation | Was the study described as randomized? | 1 |  |
|  | Was the randomization method reported? |  | 0 |
|  | Was the randomization appropriate? |  | 0 |
|  | Was the allocation concealed? |  | 0 |
| 3. Blinding | Were the study subjects blinded to the intervention received? |  | 0 |
|  | Were the researcher personnel blinded to the intervention received by the subjects? |  | 0 |
| 4. Attrition | Were attrition numerically reported? | 1 |  |
|  | Were the reasons for withdrawals and dropouts provided? | 1 |  |
| 5. Exposure/intervention | Was the type of food described (eg. Composition, matrix)? | 1 |  |
|  | Was the amount of food described (i.e. dose)? | 1 |  |
| 6. Health effect | Was the methodology used to measure the health effect reported? | 1 |  |
| 7. Statistical analysis | Was between group statistical analysis of the health effect reported? | 1 |  |
|  | Was an intention-to-treat analysis conducted? |  | 0 |
| 8. Potential confounders | Were potential confounders of the food health relationship considered? | 1 |  |
| TOTAL SCORE (maximum of 15): | | 9/15 |  |
| Higher quality (Score ≥ 8) | | X | |
| Lower quality (Score ≤ 7) | |  | |

Confounders: Participants stratified by HbA1c, age, duration of diabetes, gender, and body mass index prior to randomisation. Both groups reported a reduction in energy consumption at the end of the trial that did not differ between groups

| **Quality Appraisal Tool for Experimental Studies** | | | |
| --- | --- | --- | --- |
| **Study reference: Tamizifar et al. (2005)** | | | |
| **Item** | **Question** | **Score** | |
|  |  | YES (1) | NO /NR(0) |
| 1. Inclusion/exclusion criteria | Were the inclusion and exclusion criteria for study participation reported? (eg. Age greater than 50 years, no history of heart disease)? | 1 |  |
| 2. Group allocation | Was the study described as randomized? | 1 |  |
|  | Was the randomization method reported? |  | 0 |
|  | Was the randomization appropriate? |  | 0 (NR) |
|  | Was the allocation concealed? |  | 0 (NR) |
| 3. Blinding | Were the study subjects blinded to the intervention received? |  | 0 |
|  | Were the researcher personnel blinded to the intervention received by the subjects? | 1 |  |
| 4. Attrition | Were attrition numerically reported? | 1 |  |
|  | Were the reasons for withdrawals and dropouts provided? | 1 |  |
| 5. Exposure/intervention | Was the type of food described (eg. Composition, matrix)? | 1 |  |
|  | Was the amount of food described (i.e. dose)? | 1 |  |
| 6. Health effect | Was the methodology used to measure the health effect reported? | 1 |  |
| 7. Statistical analysis | Was between group statistical analysis of the health effect reported? | 1 |  |
|  | Was an intention-to-treat analysis conducted? |  | 0 |
| 8. Potential confounders | Were potential confounders of the food health relationship considered? | 1 |  |
| TOTAL SCORE (maximum of 15): | | 10/15 |  |
| Higher quality (Score ≥ 8) | | X | |
| Lower quality (Score ≤ 7) | |  | |

Confounders: Participants randomised based on baseline cholesterol levels. No difference in prescribed energy between periods. No difference in baseline levels between participants randomised to start almond or control diet first

| **Quality Appraisal Tool for Experimental Studies** | | | |
| --- | --- | --- | --- |
| **Study reference: Tan et al. (2013)** | | | |
| **Item** | **Question** | **Score** | |
|  |  | YES (1) | NO /NR(0) |
| 1. Inclusion/exclusion criteria | Were the inclusion and exclusion criteria for study participation reported? (eg. Age greater than 50 years, no history of heart disease)? | 1 |  |
| 2. Group allocation | Was the study described as randomized? | 1 |  |
|  | Was the randomization method reported? | 1 |  |
|  | Was the randomization appropriate? | 1 |  |
|  | Was the allocation concealed? |  | 0 (NR) |
| 3. Blinding | Were the study subjects blinded to the intervention received? |  | 0 (NR) |
|  | Were the researcher personnel blinded to the intervention received by the subjects? |  | 0 (NR) |
| 4. Attrition | Were attrition numerically reported? | 1 |  |
|  | Were the reasons for withdrawals and dropouts provided? | 1 |  |
| 5. Exposure/intervention | Was the type of food described (eg. Composition, matrix)? | 1 |  |
|  | Was the amount of food described (i.e. dose)? | 1 |  |
| 6. Health effect | Was the methodology used to measure the health effect reported? | 1 |  |
| 7. Statistical analysis | Was between group statistical analysis of the health effect reported? | 1 |  |
|  | Was an intention-to-treat analysis conducted? |  | 0 |
| 8. Potential confounders | Were potential confounders of the food health relationship considered? | 1 |  |
| TOTAL SCORE (maximum of 15): | | 11/15 |  |
| Higher quality (Score ≥ 8) | | X | |
| Lower quality (Score ≤ 7) | |  | |

Confounders: Adjustment in statistical analysis not stated, however confounders viewed as being considered due to there being no difference in baseline anthropometry, blood biochemistry and dietary intake

| **Quality Appraisal Tool for Experimental Studies** | | | |
| --- | --- | --- | --- |
| **Study reference: Tapsell et al. (2004)** | | | |
| **Item** | **Question** | **Score** | |
|  |  | YES (1) | NO /NR(0) |
| 1. Inclusion/exclusion criteria | Were the inclusion and exclusion criteria for study participation reported? (eg. Age greater than 50 years, no history of heart disease)? | 1 |  |
| 2. Group allocation | Was the study described as randomized? | 1 |  |
|  | Was the randomization method reported? |  | 0 (NR) |
|  | Was the randomization appropriate? |  | 0 (NR) |
|  | Was the allocation concealed? |  | 0 (NR) |
| 3. Blinding | Were the study subjects blinded to the intervention received? |  | 0 |
|  | Were the researcher personnel blinded to the intervention received by the subjects? |  | 0 |
| 4. Attrition | Were attrition numerically reported? | 1 |  |
|  | Were the reasons for withdrawals and dropouts provided? |  | 0 |
| 5. Exposure/intervention | Was the type of food described (eg. Composition, matrix)? | 1 |  |
|  | Was the amount of food described (i.e. dose)? | 1 |  |
| 6. Health effect | Was the methodology used to measure the health effect reported? | 1 |  |
| 7. Statistical analysis | Was between group statistical analysis of the health effect reported? | 1 |  |
|  | Was an intention-to-treat analysis conducted? | 1 |  |
| 8. Potential confounders | Were potential confounders of the food health relationship considered? |  | 0 |
| TOTAL SCORE (maximum of 15): | | 8/15 |  |
| Higher quality (Score ≥ 8) | | X | |
| Lower quality (Score ≤ 7) | |  | |

Confounders: Baseline and repeated measures analysis of weight and percent body fat adjusted for sex. Cholesterol levels of control group significantly higher than walnut at baseline – as this was a primary outcome this confounder was deemed not to be considered

| **Quality Appraisal Tool for Experimental Studies** | | | |
| --- | --- | --- | --- |
| **Study reference: Tapsell et al. (2009)** | | | |
| **Item** | **Question** | **Score** | |
|  |  | YES (1) | NO /NR(0) |
| 1. Inclusion/exclusion criteria | Were the inclusion and exclusion criteria for study participation reported? (eg. Age greater than 50 years, no history of heart disease)? | 1 |  |
| 2. Group allocation | Was the study described as randomized? | 1 |  |
|  | Was the randomization method reported? | 1 |  |
|  | Was the randomization appropriate? | 1 |  |
|  | Was the allocation concealed? | 1 |  |
| 3. Blinding | Were the study subjects blinded to the intervention received? | 1 |  |
|  | Were the researcher personnel blinded to the intervention received by the subjects? |  | 0 |
| 4. Attrition | Were attrition numerically reported? | 1 |  |
|  | Were the reasons for withdrawals and dropouts provided? | 1 |  |
| 5. Exposure/intervention | Was the type of food described (eg. Composition, matrix)? | 1 |  |
|  | Was the amount of food described (i.e. dose)? | 1 |  |
| 6. Health effect | Was the methodology used to measure the health effect reported? | 1 |  |
| 7. Statistical analysis | Was between group statistical analysis of the health effect reported? | 1 |  |
|  | Was an intention-to-treat analysis conducted? |  | 0 |
| 8. Potential confounders | Were potential confounders of the food health relationship considered? | 1 |  |
| TOTAL SCORE (maximum of 15): | | 13/15 |  |
| Higher quality (Score ≥ 8) | | X | |
| Lower quality (Score ≤ 7) | |  | |

Confounders: Sex and body composition included as covariates in statistical models where appropriate

| **Quality Appraisal Tool for Experimental Studies** | | | |
| --- | --- | --- | --- |
| **Study reference:** **Tapsell et al (2017)** | | | |
| **Item** | **Question** | **Score** | |
|  |  | YES (1) | NO /NR(0) |
| 1. Inclusion/exclusion criteria | Were the inclusion and exclusion criteria for study participation reported? (eg. Age greater than 50 years, no history of heart disease)? | 1 |  |
| 2. Group allocation | Was the study described as randomized? | 1 |  |
|  | Was the randomization method reported? | 1 |  |
|  | Was the randomization appropriate? | 1 |  |
|  | Was the allocation concealed? | 1 |  |
| 3. Blinding | Were the study subjects blinded to the intervention received? |  | 0 |
|  | Were the researcher personnel blinded to the intervention received by the subjects? | 1 |  |
| 4. Attrition | Were attrition numerically reported? | 1 |  |
|  | Were the reasons for withdrawals and dropouts provided? | 1 |  |
| 5. Exposure/intervention | Was the type of food described (e.g.. Composition, matrix)? | 1 |  |
|  | Was the amount of food described (i.e. dose)? | 1 |  |
| 6. Health effect | Was the methodology used to measure the health effect reported? | 1 |  |
| 7. Statistical analysis | Was between group statistical analysis of the health effect reported? | 1 |  |
|  | Was an intention-to-treat analysis conducted? | 1 |  |
| 8. Potential confounders | Were potential confounders of the food health relationship considered? |  | 0 |
| TOTAL SCORE (maximum of 15): | | 13/15 |  |
| Higher quality (Score ≥ 8) | | X | |
| Lower quality (Score ≤ 7) | |  | |

Confounders: The adherence of dietary intervention was not reported.

| **Quality Appraisal Tool for Experimental Studies** | | | |
| --- | --- | --- | --- |
| **Study reference: Tey et al. (2011)** | | | |
| **Item** | **Question** | **Score** | |
|  |  | YES (1) | NO /NR(0) |
| 1. Inclusion/exclusion criteria | Were the inclusion and exclusion criteria for study participation reported? (eg. Age greater than 50 years, no history of heart disease)? | 1 |  |
| 2. Group allocation | Was the study described as randomized? | 1 |  |
|  | Was the randomization method reported? |  | 0 |
|  | Was the randomization appropriate? |  | 0 (NR) |
|  | Was the allocation concealed? | 1 |  |
| 3. Blinding | Were the study subjects blinded to the intervention received? |  | 0 (NR) |
|  | Were the researcher personnel blinded to the intervention received by the subjects? |  | 0 (NR) |
| 4. Attrition | Were attrition numerically reported? | 1 |  |
|  | Were the reasons for withdrawals and dropouts provided? | 1 |  |
| 5. Exposure/intervention | Was the type of food described (eg. Composition, matrix)? | 1 |  |
|  | Was the amount of food described (i.e. dose)? | 1 |  |
| 6. Health effect | Was the methodology used to measure the health effect reported? | 1 |  |
| 7. Statistical analysis | Was between group statistical analysis of the health effect reported? | 1 |  |
|  | Was an intention-to-treat analysis conducted? | 1 |  |
| 8. Potential confounders | Were potential confounders of the food health relationship considered? | 1 |  |
| TOTAL SCORE (maximum of 15): | | 11/15 |  |
| Higher quality (Score ≥ 8) | | X | |
| Lower quality (Score ≤ 7) | |  | |

Confounders: Stratified according to age, sex and BMI prior to randomisation. Physical activity assessed via accelerometer. P-values adjusted for: baseline value, sex, baseline age, and baseline BMI. Baseline variables provided, total energy intake did not differ between groups over study

| **Quality Appraisal Tool for Experimental Studies** | | | |
| --- | --- | --- | --- |
| **Study reference: Tey et al. (2013)** | | | |
| **Item** | **Question** | **Score** | |
|  |  | YES (1) | NO /NR(0) |
| 1. Inclusion/exclusion criteria | Were the inclusion and exclusion criteria for study participation reported? (eg. Age greater than 50 years, no history of heart disease)? | 1 |  |
| 2. Group allocation | Was the study described as randomized? | 1 |  |
|  | Was the randomization method reported? | 1 |  |
|  | Was the randomization appropriate? | 1 |  |
|  | Was the allocation concealed? | 1 |  |
| 3. Blinding | Were the study subjects blinded to the intervention received? |  | 0 (NR) |
|  | Were the researcher personnel blinded to the intervention received by the subjects? | 1 |  |
| 4. Attrition | Were attrition numerically reported? | 1 |  |
|  | Were the reasons for withdrawals and dropouts provided? | 1 |  |
| 5. Exposure/intervention | Was the type of food described (eg. Composition, matrix)? | 1 |  |
|  | Was the amount of food described (i.e. dose)? | 1 |  |
| 6. Health effect | Was the methodology used to measure the health effect reported? | 1 |  |
| 7. Statistical analysis | Was between group statistical analysis of the health effect reported? | 1 |  |
|  | Was an intention-to-treat analysis conducted? | 1 |  |
| 8. Potential confounders | Were potential confounders of the food health relationship considered? | 1 |  |
| TOTAL SCORE (maximum of 15): | | 14/15 |  |
| Higher quality (Score ≥ 8) | | X | |
| Lower quality (Score ≤ 7) | |  | |

Confounders: Analysis adjusted for baseline values, age, gender and (where appropriate) BMI. Both hazelnut diets consumed greater energy compared to control at 7 weeks (last data available), p=0.013, though no significant differences at baseline

| **Quality Appraisal Tool for Experimental Studies** | | | |
| --- | --- | --- | --- |
| **Study reference:** Tindall et al (2019) | |  | |
| **Item** | **Question** | **Score** | |
|  |  | Yes | No/NR |
| 1. Inclusion/ Exclusion Criteria | Were the inclusion and/or exclusion criteria for study participation reported (*e.g.*, age greater than 50 years, no history of heart disease)? | 1 |  |
| 2. Group Allocation | Was the study described as randomized? | 1 |  |
|  | Was the randomization method reported? | 1 |  |
|  | Was the randomization method appropriate? | 1 |  |
|  | Was allocation concealed? |  | 0 (NR) |
| 3. Blinding | Were the study subjects blinded to the intervention received? |  | 0 |
|  | Were the research personnel blinded to the intervention received by the subjects? | 1 |  |
| 4. Attrition | Was attrition numerically reported? | 1 |  |
|  | Were the reasons for withdrawals and dropouts provided? | 1 |  |
| 5. Exposure/  Intervention | Was the type of food described (e.g., composition, matrix)? | 1 |  |
|  | Was the amount of food described (i.e., dose)? | 1 |  |
| 6. Health Effect | Was the methodology used to measure the health effect reported? | 1 |  |
| 7. Statistical Analysis | Was a between-group statistical analysis of the health effect conducted (*i.e.*, control vs. intervention)? | 1 |  |
|  | Was an intention-to-treat analysis conducted? | 1 |  |
| 8. Potential Confounders | Were potential confounders of the food/health relationship considered? | 1 |  |
| TOTAL SCORE (maximum of 15): | | 13/15 | |
| Higher quality (Score ≥ 8) | | X | |
| Lower quality (Score ≤ 7) | |  | |

Confounders: not applicable as randomised cross-over design

| **Quality Appraisal Tool for Experimental Studies** | | | |
| --- | --- | --- | --- |
| **Study reference: PREDIMED** | | | |
| **Item** | **Question** | **Score** | |
|  |  | YES (1) | NO /NR(0) |
| 1. Inclusion/exclusion criteria | Were the inclusion and exclusion criteria for study participation reported? (eg. Age greater than 50 years, no history of heart disease)? | 1 |  |
| 2. Group allocation | Was the study described as randomized? | 1 |  |
|  | Was the randomization method reported? | 1 |  |
|  | Was the randomization appropriate? | 1 |  |
|  | Was the allocation concealed? | 1 |  |
| 3. Blinding | Were the study subjects blinded to the intervention received? |  | 0 (NR) |
|  | Were the researcher personnel blinded to the intervention received by the subjects? | 1 |  |
| 4. Attrition | Were attrition numerically reported? | 1 |  |
|  | Were the reasons for withdrawals and dropouts provided? | 1 |  |
| 5. Exposure/intervention | Was the type of food described (eg. Composition, matrix)? | 1 |  |
|  | Was the amount of food described (i.e. dose)? | 1 |  |
| 6. Health effect | Was the methodology used to measure the health effect reported? | 1 |  |
| 7. Statistical analysis | Was between group statistical analysis of the health effect reported? | 1 |  |
|  | Was an intention-to-treat analysis conducted? | 1 |  |
| 8. Potential confounders | Were potential confounders of the food health relationship considered? | 1 |  |
| TOTAL SCORE (maximum of 15): | | 14/15 |  |
| Higher quality (Score ≥ 8) | | X | |
| Lower quality (Score ≤ 7) | |  | |

Confounders: Adjusted for sex, baseline clinical characteristics compared, change in dietary intake assessed

| **Quality Appraisal Tool for Experimental Studies** | | | |
| --- | --- | --- | --- |
| **Study reference: Wang et al. (2012)** | | | |
| **Item** | **Question** | **Score** | |
|  |  | YES (1) | NO /NR(0) |
| 1. Inclusion/exclusion criteria | Were the inclusion and exclusion criteria for study participation reported? (eg. Age greater than 50 years, no history of heart disease)? | 1 |  |
| 2. Group allocation | Was the study described as randomized? | 1 |  |
|  | Was the randomization method reported? |  | 0 (NR) |
|  | Was the randomization appropriate? |  | 0 (NR) |
|  | Was the allocation concealed? |  | 0 (NR) |
| 3. Blinding | Were the study subjects blinded to the intervention received? |  | 0 (NR) |
|  | Were the researcher personnel blinded to the intervention received by the subjects? |  | 0 (NR) |
| 4. Attrition | Were attrition numerically reported? | 1 |  |
|  | Were the reasons for withdrawals and dropouts provided? | 1 |  |
| 5. Exposure/intervention | Was the type of food described (eg. Composition, matrix)? | 1 |  |
|  | Was the amount of food described (i.e. dose)? | 1 |  |
| 6. Health effect | Was the methodology used to measure the health effect reported? | 1 |  |
| 7. Statistical analysis | Was between group statistical analysis of the health effect reported? | 1 |  |
|  | Was an intention-to-treat analysis conducted? |  | 0 |
| 8. Potential confounders | Were potential confounders of the food health relationship considered? |  | 0 |
| TOTAL SCORE (maximum of 15): | | 8/15 |  |
| Higher quality (Score ≥ 8) | | X | |
| Lower quality (Score ≤ 7) | |  | |

Confounders: Values included in paper were not adjusted for confounders. Dietary intake was not compared between groups

| **Quality Appraisal Tool for Experimental Studies** | | | |
| --- | --- | --- | --- |
| **Study reference:** Wang et al. (2021a) | |  | |
| **Item** | **Question** | **Score** | |
|  |  | Yes | No/NR |
| 1. Inclusion/ Exclusion Criteria | Were the inclusion and/or exclusion criteria for study participation reported (*e.g.*, age greater than 50 years, no history of heart disease)? | 1 |  |
| 2. Group Allocation | Was the study described as randomized? | 1 |  |
|  | Was the randomization method reported? | 1 |  |
|  | Was the randomization method appropriate? | 1 |  |
|  | Was allocation concealed? | 1 |  |
| 3. Blinding | Were the study subjects blinded to the intervention received? |  | 0 (NR) |
|  | Were the research personnel blinded to the intervention received by the subjects? | 1 |  |
| 4. Attrition | Was attrition numerically reported? | 1 |  |
|  | Were the reasons for withdrawals and dropouts provided? | 1 |  |
| 5. Exposure/  Intervention | Was the type of food described (e.g., composition, matrix)? | 1 |  |
|  | Was the amount of food described (i.e., dose)? | 1 |  |
| 6. Health Effect | Was the methodology used to measure the health effect reported? | 1 |  |
| 7. Statistical Analysis | Was a between-group statistical analysis of the health effect conducted (*i.e.*, control vs. intervention)? | 1 |  |
|  | Was an intention-to-treat analysis conducted? | 1 |  |
| 8. Potential Confounders | Were potential confounders of the food/health relationship considered? | 1 |  |
| TOTAL SCORE (maximum of 15): | | 14/15 | |
| Higher quality (Score ≥ 8) | | X | |
| Lower quality (Score ≤ 7) | |  | |

Confounders: All participants were advised to maintain their habitual level of physical activity and background diet

| **Quality Appraisal Tool for Experimental Studies** | | | |
| --- | --- | --- | --- |
| **Study reference:** Wang et al. (2021b) | |  | |
| **Item** | **Question** | **Score** | |
|  |  | Yes | No/NR |
|  |  |  |  |
| 1. Inclusion/ Exclusion Criteria | Were the inclusion and/or exclusion criteria for study participation reported (*e.g.*, age greater than 50 years, no history of heart disease)? | 1 |  |
| 2. Group Allocation | Was the study described as randomized? | 1 |  |
|  | Was the randomization method reported? | 1 |  |
|  | Was the randomization method appropriate? | 1 |  |
|  | Was allocation concealed? |  | 0 (NR) |
| 3. Blinding | Were the study subjects blinded to the intervention received? |  | 0 (NR) |
|  | Were the research personnel blinded to the intervention received by the subjects? |  | 0 (NR) |
| 4. Attrition | Was attrition numerically reported? | 1 |  |
|  | Were the reasons for withdrawals and dropouts provided? |  | 0 (NR) |
| 5. Exposure/  Intervention | Was the type of food described (e.g., composition, matrix)? | 1 |  |
|  | Was the amount of food described (i.e., dose)? | 1 |  |
| 6. Health Effect | Was the methodology used to measure the health effect reported? | 1 |  |
| 7. Statistical Analysis | Was a between-group statistical analysis of the health effect conducted (*i.e.*, control vs. intervention)? | 1 |  |
|  | Was an intention-to-treat analysis conducted? |  | 0 |
| 8. Potential Confounders | Were potential confounders of the food/health relationship considered? |  | 0 |
| TOTAL SCORE (maximum of 15): | | 9/15 | |
| Higher quality (Score ≥ 8) | | X | |
| Lower quality (Score ≤ 7) | |  | |

| **Quality Appraisal Tool for Experimental Studies** | | | |
| --- | --- | --- | --- |
| **Study reference: Wien et al. (2003)** | | | |
| **Item** | **Question** | **Score** | |
|  |  | YES (1) | NO /NR(0) |
| 1. Inclusion/exclusion criteria | Were the inclusion and exclusion criteria for study participation reported? (eg. Age greater than 50 years, no history of heart disease)? | 1 |  |
| 2. Group allocation | Was the study described as randomized? | 1 |  |
|  | Was the randomization method reported? | 1 |  |
|  | Was the randomization appropriate? | 1 |  |
|  | Was the allocation concealed? |  | 0 (NR) |
| 3. Blinding | Were the study subjects blinded to the intervention received? |  | 0 (NR) |
|  | Were the researcher personnel blinded to the intervention received by the subjects? |  | 0 (NR) |
| 4. Attrition | Were attrition numerically reported? | 1 |  |
|  | Were the reasons for withdrawals and dropouts provided? | 1 |  |
| 5. Exposure/intervention | Was the type of food described (eg. Composition, matrix)? | 1 |  |
|  | Was the amount of food described (i.e. dose)? | 1 |  |
| 6. Health effect | Was the methodology used to measure the health effect reported? | 1 |  |
| 7. Statistical analysis | Was between group statistical analysis of the health effect reported? | 1 |  |
|  | Was an intention-to-treat analysis conducted? | 1 |  |
| 8. Potential confounders | Were potential confounders of the food health relationship considered? | 1 |  |
| TOTAL SCORE (maximum of 15): | | 12/15 |  |
| Higher quality (Score ≥ 8) | | X | |
| Lower quality (Score ≤ 7) | |  | |

Confounders: Models were adjusted for baseline measurements, both intention-to-treat and as-treated analysis conducted (intention-to-treat presented in paper as is less biased). Randomisation was stratified according to gender and documented presence/absence of Type II diabetes

| **Quality Appraisal Tool for Experimental Studies** | | | |
| --- | --- | --- | --- |
| **Study reference: Wien et al. (2010)** | | | |
| **Item** | **Question** | **Score** | |
|  |  | YES (1) | NO /NR(0) |
| 1. Inclusion/exclusion criteria | Were the inclusion and exclusion criteria for study participation reported? (eg. Age greater than 50 years, no history of heart disease)? | 1 |  |
| 2. Group allocation | Was the study described as randomized? | 1 |  |
|  | Was the randomization method reported? | 1 |  |
|  | Was the randomization appropriate? | 1 |  |
|  | Was the allocation concealed? | 1 |  |
| 3. Blinding | Were the study subjects blinded to the intervention received? |  | 0 (NR) |
|  | Were the researcher personnel blinded to the intervention received by the subjects? |  | 0 (NR) |
| 4. Attrition | Were attrition numerically reported? | 1 |  |
|  | Were the reasons for withdrawals and dropouts provided? | 1 |  |
| 5. Exposure/intervention | Was the type of food described (eg. Composition, matrix)? | 1 |  |
|  | Was the amount of food described (i.e. dose)? | 1 |  |
| 6. Health effect | Was the methodology used to measure the health effect reported? | 1 |  |
| 7. Statistical analysis | Was between group statistical analysis of the health effect reported? | 1 |  |
|  | Was an intention-to-treat analysis conducted? | 1 |  |
| 8. Potential confounders | Were potential confounders of the food health relationship considered? | 1 |  |
| TOTAL SCORE (maximum of 15): | | 13/15 |  |
| Higher quality (Score ≥ 8) | | X | |
| Lower quality (Score ≤ 7) | |  | |

Confounders: Repeated measures analysis of covariance adjusted for baseline measurements of outcomes. No difference between groups in change in total energy (although significant differences between groups in macronutrients)

| **Quality Appraisal Tool for Experimental Studies** | | | |
| --- | --- | --- | --- |
| **Study reference: Wien et al. (2014)** | | | |
| **Item** | **Question** | **Score** | |
|  |  | YES (1) | NO /NR(0) |
| 1. Inclusion/exclusion criteria | Were the inclusion and exclusion criteria for study participation reported? (eg. Age greater than 50 years, no history of heart disease)? | 1 |  |
| 2. Group allocation | Was the study described as randomized? | 1 |  |
|  | Was the randomization method reported? | 1 |  |
|  | Was the randomization appropriate? | 1 |  |
|  | Was the allocation concealed? |  | 0 NR |
| 3. Blinding | Were the study subjects blinded to the intervention received? |  | 0 |
|  | Were the researcher personnel blinded to the intervention received by the subjects? |  | 0 |
| 4. Attrition | Were attrition numerically reported? | 1 |  |
|  | Were the reasons for withdrawals and dropouts provided? | 1 |  |
| 5. Exposure/intervention | Was the type of food described (eg. Composition, matrix)? | 1 |  |
|  | Was the amount of food described (i.e. dose)? | 1 |  |
| 6. Health effect | Was the methodology used to measure the health effect reported? | 1 |  |
| 7. Statistical analysis | Was between group statistical analysis of the health effect reported? | 1 |  |
|  | Was an intention-to-treat analysis conducted? | 1 |  |
| 8. Potential confounders | Were potential confounders of the food health relationship considered? | 1 |  |
| TOTAL SCORE (maximum of 15): | | 12/15 |  |
| Higher quality (Score ≥ 8) | | X | |
| Lower quality (Score ≤ 7) | |  | |

Confounders: Analyses adjusted for baseline values, weight change included in model for waist circumference and biological measures. Analyses were also stratified by age, gender, baseline BMI and statin us

| **Quality Appraisal Tool for Experimental Studies** | | | |
| --- | --- | --- | --- |
| **Study reference:** Williams et al (2019) | |  | |
| **Item** | **Question** | **Score** | |
|  |  | Yes | No/NR |
| 1. Inclusion/ Exclusion Criteria | Were the inclusion and/or exclusion criteria for study participation reported (*e.g.*, age greater than 50 years, no history of heart disease)? | 1 |  |
| 2. Group Allocation | Was the study described as randomized? | 1 |  |
|  | Was the randomization method reported? | 1 |  |
|  | Was the randomization method appropriate? | 1 |  |
|  | Was allocation concealed? | 1 |  |
| 3. Blinding | Were the study subjects blinded to the intervention received? |  | 0 |
|  | Were the research personnel blinded to the intervention received by the subjects? | 1 |  |
| 4. Attrition | Was attrition numerically reported? | 1 |  |
|  | Were the reasons for withdrawals and dropouts provided? | 1 |  |
| 5. Exposure/  Intervention | Was the type of food described (e.g., composition, matrix)? | 1 |  |
|  | Was the amount of food described (i.e., dose)? | 1 |  |
| 6. Health Effect | Was the methodology used to measure the health effect reported? | 1 |  |
| 7. Statistical Analysis | Was a between-group statistical analysis of the health effect conducted (*i.e.*, control vs. intervention)? | 1 |  |
|  | Was an intention-to-treat analysis conducted? |  | 0 |
| 8. Potential Confounders | Were potential confounders of the food/health relationship considered? | 1 |  |
| TOTAL SCORE (maximum of 15): | | 13/15 | |
| Higher quality (Score ≥ 8) | | X | |
| Lower quality (Score ≤ 7) | |  | |

Confounders: not applicable as randomised cross-over design

| **Quality Appraisal Tool for Experimental Studies** | | | |
| --- | --- | --- | --- |
| **Study reference: Wu et al. (2010)** | | | |
| **Item** | **Question** | **Score** |  |
|  |  | YES (1) | NO /NR(0) |
| 1. Inclusion/exclusion criteria | Were the inclusion and exclusion criteria for study participation reported? (eg. Age greater than 50 years, no history of heart disease)? | 1 |  |
| 2. Group allocation | Was the study described as randomized? | 1 |  |
|  | Was the randomization method reported? |  | 0 |
|  | Was the randomization appropriate? |  | 0 (NR) |
|  | Was the allocation concealed? |  | 0 (NR) |
| 3. Blinding | Were the study subjects blinded to the intervention received? |  | 0 |
|  | Were the researcher personnel blinded to the intervention received by the subjects? | 1 |  |
| 4. Attrition | Were attrition numerically reported? | 1 |  |
|  | Were the reasons for withdrawals and dropouts provided? | 1 |  |
| 5. Exposure/intervention | Was the type of food described (eg. Composition, matrix)? | 1 |  |
|  | Was the amount of food described (i.e. dose)? | 1 |  |
| 6. Health effect | Was the methodology used to measure the health effect reported? | 1 |  |
| 7. Statistical analysis | Was between group statistical analysis of the health effect reported? | 1 |  |
|  | Was an intention-to-treat analysis conducted? | 1 |  |
| 8. Potential confounders | Were potential confounders of the food health relationship considered? | 1 |  |
| TOTAL SCORE (maximum of 15): | | 11/15 |  |
| Higher quality (Score ≥ 8) | | X | |
| Lower quality (Score ≤ 7) | |  | |

Confounders: Subgroup analyses stratified by sex, baseline central obesity status, baseline BMI, and weight change was performed. No difference in variables between groups at baseline. All groups experienced significant reductions in energy from baseline, no differences between groups at 12 weeks

| **Quality Appraisal Tool for Experimental Studies** | | | |
| --- | --- | --- | --- |
| **Study reference: Wu et al. (2014)** | | | |
| **Item** | **Question** | **Score** |  |
|  |  | YES (1) | NO /NR(0) |
| 1. Inclusion/exclusion criteria | Were the inclusion and exclusion criteria for study participation reported? (eg. Age greater than 50 years, no history of heart disease)? | 1 |  |
| 2. Group allocation | Was the study described as randomized? | 1 |  |
|  | Was the randomization method reported? | 1 |  |
|  | Was the randomization appropriate? | 1 |  |
|  | Was the allocation concealed? |  | 0(NR) |
| 3. Blinding | Were the study subjects blinded to the intervention received? |  | 0 (NR) |
|  | Were the researcher personnel blinded to the intervention received by the subjects? |  | 0 (NR) |
| 4. Attrition | Were attrition numerically reported? | 1 |  |
|  | Were the reasons for withdrawals and dropouts provided? | 1 |  |
| 5. Exposure/intervention | Was the type of food described (eg. Composition, matrix)? | 1 |  |
|  | Was the amount of food described (i.e. dose)? | 1 |  |
| 6. Health effect | Was the methodology used to measure the health effect reported? | 1 |  |
| 7. Statistical analysis | Was between group statistical analysis of the health effect reported? | 1 |  |
|  | Was an intention-to-treat analysis conducted? |  | 0 |
| 8. Potential confounders | Were potential confounders of the food health relationship considered? | 1 |  |
| TOTAL SCORE (maximum of 15): | | 11/15 |  |
| Higher quality (Score ≥ 8) | | X | |
| Lower quality (Score ≤ 7) | |  | |

Confounders: gender, age, diet sequence

| **Quality Appraisal Tool for Experimental Studies** | | | |
| --- | --- | --- | --- |
| **Study reference: Zambon et al. (2000)** | | | |
| **Item** | **Question** | **Score** | |
|  |  | YES (1) | NO /NR(0) |
| 1. Inclusion/exclusion criteria | Were the inclusion and exclusion criteria for study participation reported? (eg. Age greater than 50 years, no history of heart disease)? | 1 |  |
| 2. Group allocation | Was the study described as randomized? | 1 |  |
|  | Was the randomization method reported? | 1 |  |
|  | Was the randomization appropriate? | 1 |  |
|  | Was the allocation concealed? |  | 0 (NR) |
| 3. Blinding | Were the study subjects blinded to the intervention received? |  | 0 |
|  | Were the researcher personnel blinded to the intervention received by the subjects? |  | 0 |
| 4. Attrition | Were attrition numerically reported? | 1 |  |
|  | Were the reasons for withdrawals and dropouts provided? | 1 |  |
| 5. Exposure/intervention | Was the type of food described (eg. Composition, matrix)? | 1 |  |
|  | Was the amount of food described (i.e. dose)? | 1 |  |
| 6. Health effect | Was the methodology used to measure the health effect reported? | 1 |  |
| 7. Statistical analysis | Was between group statistical analysis of the health effect reported? | 1 |  |
|  | Was an intention-to-treat analysis conducted? |  | 0 (NR) |
| 8. Potential confounders | Were potential confounders of the food health relationship considered? | 1 |  |
| TOTAL SCORE (maximum of 15): | | 11/15 |  |
| Higher quality (Score ≥ 8) | | X | |
| Lower quality (Score ≤ 7) | |  | |

Confounders: Randomisation stratified by sex. Baseline values or sex were used as covariates in the analysis. Dietary intake was compared, no significant difference in total energy between arms. Justified lack of wash out period, assessed for carryover effect (no evidence of effect found)

| **Quality Appraisal Tool for Experimental Studies** | | | |
| --- | --- | --- | --- |
| **Study reference: Zaveri et al. (2009)** | | | |
| **Item** | **Question** | **Score** | |
|  |  | YES (1) | NO /NR(0) |
| 1. Inclusion/exclusion criteria | Were the inclusion and exclusion criteria for study participation reported? (eg. Age greater than 50 years, no history of heart disease)? | 1 |  |
| 2. Group allocation | Was the study described as randomized? |  | 0 |
|  | Was the randomization method reported? |  | 0 |
|  | Was the randomization appropriate? |  | 0 (NR) |
|  | Was the allocation concealed? |  | 0 (NR) |
| 3. Blinding | Were the study subjects blinded to the intervention received? |  | 0 (NR) |
|  | Were the researcher personnel blinded to the intervention received by the subjects? |  | 0 (NR) |
| 4. Attrition | Were attrition numerically reported? | 1 |  |
|  | Were the reasons for withdrawals and dropouts provided? |  | 0 |
| 5. Exposure/intervention | Was the type of food described (eg. Composition, matrix)? | 1 |  |
|  | Was the amount of food described (i.e. dose)? | 1 |  |
| 6. Health effect | Was the methodology used to measure the health effect reported? | 1 |  |
| 7. Statistical analysis | Was between group statistical analysis of the health effect reported? | 1 |  |
|  | Was an intention-to-treat analysis conducted? |  | 0 |
| 8. Potential confounders | Were potential confounders of the food health relationship considered? | 1 |  |
| TOTAL SCORE (maximum of 15): | | 7/15 |  |
| Higher quality (Score ≥ 8) | |  | |
| Lower quality (Score ≤ 7) | | X | |

Confounders: No difference in anthropometric characteristics at baseline. No difference in total energy consumed between groups over the duration of the study

| **Quality Appraisal Tool for Experimental Studies** | | | |
| --- | --- | --- | --- |
| **Study reference:** Zhao et al. (2004)/ West et al. (2010) | |  | |
| **Item** | **Question** | **Score** | |
|  |  | Yes | No/NR |
| 1. Inclusion/ Exclusion Criteria | Were the inclusion and/or exclusion criteria for study participation reported (*e.g.*, age greater than 50 years, no history of heart disease)? | 1 |  |
| 2. Group Allocation | Was the study described as randomized? | 1 |  |
|  | Was the randomization method reported? | 1 |  |
|  | Was the randomization method appropriate? | 1 |  |
|  | Was allocation concealed? | 1 |  |
| 3. Blinding | Were the study subjects blinded to the intervention received? |  | 0 |
|  | Were the research personnel blinded to the intervention received by the subjects? | 1 |  |
| 4. Attrition | Was attrition numerically reported? | 1 |  |
|  | Were the reasons for withdrawals and dropouts provided? |  | 0 |
| 5. Exposure/  Intervention | Was the type of food described (e.g., composition, matrix)? | 1 |  |
|  | Was the amount of food described (i.e., dose)? | 1 |  |
| 6. Health Effect | Was the methodology used to measure the health effect reported? | 1 |  |
| 7. Statistical Analysis | Was a between-group statistical analysis of the health effect conducted (*i.e.*, control vs. intervention)? | 1 |  |
|  | Was an intention-to-treat analysis conducted? | 1 |  |
| 8. Potential Confounders | Were potential confounders of the food/health relationship considered? | 1 |  |
| TOTAL SCORE (maximum of 15): | | 13/15 | |
| Higher quality (Score ≥ 8) | | X | |
| Lower quality (Score ≤ 7) | |  | |

Confounders: not applicable as randomised cross-over design

| **Quality Appraisal Tool for Experimental Studies** | | | |
| --- | --- | --- | --- |
| **Study reference: Zibaeenezhad et al. (2003)** | | | |
| **Item** | **Question** | **Score** | |
|  |  | YES (1) | NO /NR(0) |
| 1. Inclusion/exclusion criteria | Were the inclusion and exclusion criteria for study participation reported? (eg. Age greater than 50 years, no history of heart disease)? |  | 0 |
| 2. Group allocation | Was the study described as randomized? | 1 |  |
|  | Was the randomization method reported? |  | 0 (NR) |
|  | Was the randomization appropriate? |  | 0 (NR) |
|  | Was the allocation concealed? |  | 0 (NR) |
| 3. Blinding | Were the study subjects blinded to the intervention received? | 1 |  |
|  | Were the researcher personnel blinded to the intervention received by the subjects? | 1 |  |
| 4. Attrition | Were attrition numerically reported? |  | 0 |
|  | Were the reasons for withdrawals and dropouts provided? |  | 0 |
| 5. Exposure/intervention | Was the type of food described (eg. Composition, matrix)? | 1 |  |
|  | Was the amount of food described (i.e. dose)? | 1 |  |
| 6. Health effect | Was the methodology used to measure the health effect reported? | 1 |  |
| 7. Statistical analysis | Was between group statistical analysis of the health effect reported? |  | 0 |
|  | Was an intention-to-treat analysis conducted? |  | 0 |
| 8. Potential confounders | Were potential confounders of the food health relationship considered? |  | 0 |
| TOTAL SCORE (maximum of 15): | | 6/15 |  |
| Higher quality (Score ≥ 8) | |  | |
| Lower quality (Score ≤ 7) | | X | |

Confounders: No assessment of background diet at any time in either group. Triglyceride levels of walnut group substantially higher at baseline than control group (not compared statistically). No confounders considered in analysis

| **Quality Appraisal Tool for Experimental Studies** | | | |
| --- | --- | --- | --- |
| **Study reference: Zibaeenezhad et al. (2005)** | | | |
| **Item** | **Question** | **Score** | |
|  |  | YES (1) | NO /NR(0) |
| 1. Inclusion/exclusion criteria | Were the inclusion and exclusion criteria for study participation reported? (eg. Age greater than 50 years, no history of heart disease)? | 1 |  |
| 2. Group allocation | Was the study described as randomized? | 1 |  |
|  | Was the randomization method reported? |  | 0 (NR) |
|  | Was the randomization appropriate? |  | 0 (NR) |
|  | Was the allocation concealed? |  | 0 (NR) |
| 3. Blinding | Were the study subjects blinded to the intervention received? |  | 0 (NR) |
|  | Were the researcher personnel blinded to the intervention received by the subjects? |  | 0 (NR) |
| 4. Attrition | Were attrition numerically reported? | 1 |  |
|  | Were the reasons for withdrawals and dropouts provided? |  | 0 |
| 5. Exposure/intervention | Was the type of food described (eg. Composition, matrix)? | 1 |  |
|  | Was the amount of food described (i.e. dose)? | 1 |  |
| 6. Health effect | Was the methodology used to measure the health effect reported? | 1 |  |
| 7. Statistical analysis | Was between group statistical analysis of the health effect reported? | 1 |  |
|  | Was an intention-to-treat analysis conducted? |  | 0 |
| 8. Potential confounders | Were potential confounders of the food health relationship considered? |  | 0 |
| TOTAL SCORE (maximum of 15): | | 7/15 |  |
| Higher quality (Score ≥ 8) | |  | |
| Lower quality (Score ≤ 7) | | X | |

Confounders: No assessment of background diet at any time in either group. Triglyceride levels of walnut group substantially higher at baseline than control group (not compared statistically). No confounders considered in analysis

| **Quality Appraisal Tool for Experimental Studies** | | | |
| --- | --- | --- | --- |
| **Study reference:** Zibaeenezhad et al (2017) | | | |
| **Item** | **Question** | **Score** | |
|  |  | YES (1) | NO /NR(0) |
| 1. Inclusion/exclusion criteria | Were the inclusion and exclusion criteria for study participation reported? (eg. Age greater than 50 years, no history of heart disease)? | 1 |  |
| 2. Group allocation | Was the study described as randomized? | 1 |  |
|  | Was the randomization method reported? | 1 |  |
|  | Was the randomization appropriate? | 1 |  |
|  | Was the allocation concealed? | 1 |  |
| 3. Blinding | Were the study subjects blinded to the intervention received? | 1 |  |
|  | Were the researcher personnel blinded to the intervention received by the subjects? | 1 |  |
| 4. Attrition | Were attrition numerically reported? | 1 |  |
|  | Were the reasons for withdrawals and dropouts provided? | 1 |  |
| 5. Exposure/intervention | Was the type of food described (e.g.. Composition, matrix)? | 1 |  |
|  | Was the amount of food described (i.e. dose)? | 1 |  |
| 6. Health effect | Was the methodology used to measure the health effect reported? | 1 |  |
| 7. Statistical analysis | Was between group statistical analysis of the health effect reported? | 1 |  |
|  | Was an intention-to-treat analysis conducted? |  | 0 |
| 8. Potential confounders | Were potential confounders of the food health relationship considered? |  | 0 |
| TOTAL SCORE (maximum of 15): | | 13/15 |  |
| Higher quality (Score ≥ 8) | | X | |
| Lower quality (Score ≤ 7) | |  | |

Confounders: The adherence to the dietary intervention was not reported.

| **Quality Appraisal Tool for Experimental Studies** | | | |
| --- | --- | --- | --- |
| **Study reference:** Zibaeenezhad et al (2019) | |  | |
| **Item** | **Question** | **Score** | |
|  |  | YES (1) | NO /NR(0) |
| 1. Inclusion/ Exclusion Criteria | Were the inclusion and/or exclusion criteria for study participation reported (*e.g.*, age greater than 50 years, no history of heart disease)? | 1 |  |
| 2. Group Allocation | Was the study described as randomized? | 1 |  |
|  | Was the randomization method reported? | 1 |  |
|  | Was the randomization method appropriate? | 1 |  |
|  | Was allocation concealed? | 1 |  |
| 3. Blinding | Were the study subjects blinded to the intervention received? |  | 0 (NR) |
|  | Were the research personnel blinded to the intervention received by the subjects? | 1 |  |
| 4. Attrition | Was attrition numerically reported? | 1 |  |
|  | Were the reasons for withdrawals and dropouts provided? | 1 |  |
| 5. Exposure/  Intervention | Was the type of food described (e.g., composition, matrix)? | 1 |  |
|  | Was the amount of food described (i.e., dose)? | 1 |  |
| 6. Health Effect | Was the methodology used to measure the health effect reported? | 1 |  |
| 7. Statistical Analysis | Was a between-group statistical analysis of the health effect conducted (*i.e.*, control vs. intervention)? | 1 |  |
|  | Was an intention-to-treat analysis conducted? |  | 0 |
| 8. Potential Confounders | Were potential confounders of the food/health relationship considered? |  | 0 |
| TOTAL SCORE (maximum of 15): | | 12/15 | |
| Higher quality (Score ≥ 8) | | X | |
| Lower quality (Score ≤ 7) | |  | |

 Confounders: did not collect information on physical activity

# **Supplementary Table 16:** GRADE evidence profile

| **Certainty assessment** | | | | | | | **№ of patients** | | **Effect** | | **Certainty** | **Importance** |
| --- | --- | --- | --- | --- | --- | --- | --- | --- | --- | --- | --- | --- |
| **№ of studies** | **Study design** | **Risk of bias** | **Inconsistency** | **Indirectness** | **Imprecision** | **Other considerations** | **Nut consumption** | **control** | **Relative (95% CI)** | **Absolute (95% CI)** |  |  |
| LDL cholesterol | | | | | | | | | | | | |
| 126 | randomised trials | not serious | very serious^a^ | not serious | not serious | publication bias strongly suspected^b^ | 4911 | 4576 | - | MD **0.11 mmol/L lower** (0.14 lower to 0.07 lower) | ⨁◯◯◯ Very low | IMPORTANT |
| HDL cholesterol | | | | | | | | | | | | |
| 126 | randomised trials | not serious | very serious^c^ | not serious | not serious | publication bias strongly suspected^d^ | 5212 | 4887 | - | MD **0 mmol/L**  (0.01 lower to 0.02 higher) | ⨁◯◯◯ Very low | IMPORTANT |
| TC | | | | | | | | | | | | |
| 126 | randomised trials | not serious | very serious^e^ | not serious | not serious | publication bias strongly suspected^f^ | 4861 | 4577 | - | MD **0.13 mmol/L lower** (0.18 lower to 0.09 lower) | ⨁◯◯◯ Very low | IMPORTANT |
| TG | | | | | | | | | | | | |
| 122 | randomised trials | not serious | serious^g^ | not serious | not serious | publication bias strongly suspected^h^ | 5010 | 4731 | - | MD **0.06 mmol/L lower** (0.08 lower to 0.03 lower) | ⨁⨁◯◯ Low | IMPORTANT |
| TG: HDL cholesterol | | | | | | | | | | | | |
| 5 | randomised trials | not serious | not serious | not serious | serious^i^ | publication bias strongly suspected^j^ | 187 | 144 | - | MD **0.12 lower** (0.40 lower to 0.15 higher) | ⨁⨁◯◯ Low | IMPORTANT |
| TC:HDL cholesterol | | | | | | | | | | | | |
| 51 | randomised trials | not serious | serious^k^ | not serious | not serious | none | 2013 | 1812 | - | MD **0.15 lower** (0.24 lower to 0.07 lower) | ⨁⨁⨁◯ Moderate | IMPORTANT |
| HDL cholesterol:LDL cholesterol | | | | | | | | | | | | |
| 3 | randomised trials | not serious | serious^l^ | not serious | serious^m^ | publication bias strongly suspected^n^ | 131 | 78 | - | MD **0.06 lower** (0.23 lower to 0.11 higher) | ⨁◯◯◯ Very low | IMPORTANT |
| LDL cholesterol: HDL cholesterol | | | | | | | | | | | | |
| 39 | randomised trials | not serious | serious^o^ | not serious | not serious | none | 1335 | 1192 | - | MD **0.14 lower** (0.20 lower to 0.08 lower) | ⨁⨁⨁◯ Moderate | IMPORTANT |
| ApoB | | | | | | | | | | | | |
| 40 | randomised trials | not serious | serious^p^ | not serious | not serious | none | 1532 | 1537 | - | MD **3.01 mg/dL lower** (4.44 lower to 1.58 lower) | ⨁⨁⨁◯ Moderate | IMPORTANT |
| ApoA-I | | | | | | | | | | | | |
| 33 | randomised trials | not serious | not serious | not serious | not serious | none | 1119 | 1140 | - | MD **1.02 mg/dL lower** (2.53 lower to 0.49 higher) | ⨁⨁⨁⨁ High | IMPORTANT |
| SBP | | | | | | | | | | | | |
| 71 | randomised trials | not serious | serious^q^ | not serious | not serious | publication bias strongly suspected^r^ | 3373 | 3143 | - | MD **0.05 mmHg lower** (0.82 lower to 0.73 higher) | ⨁⨁◯◯ Low | IMPORTANT |
| DBP | | | | | | | | | | | | |
| 67 | randomised trials | not serious | not serious | not serious | not serious | publication bias strongly suspected^s^ | 3175 | 3023 | - | MD **0.10 mmHg lower** (0.53 lower to 0.33 higher) | ⨁⨁⨁◯ Moderate | IMPORTANT |

**CI:** confidence interval; **MD:** mean difference

a. I squared value of 80% indicating high heterogeneity

b. Egger's test indicates funnel plot asymmetry, suggesting the presence of small study effects which may have been attributable to publication bias.

c. I squared of 81%, indicating high heterogeneity

d. Egger's test indicates funnel plot asymmetry, suggesting the presence of small study effects which may have been attributable to publication bias.

e. I squared of 87%, indicating high heterogeneity

f. Egger's test indicates funnel plot asymmetry, suggesting the presence of small study effects which may have been attributable to publication bias.

g. I squared of 59%, indicating moderate heterogeneity

h. Egger's test indicates funnel plot asymmetry, suggesting the presence of small study effects which may be due to publication bias

i. Total sample size of 331, which is unlikely to meet the optimal information size

j. Funnel plot unable to be completed based on number of studies (<10), however 3/5 studies had funding sources which may have resulted in publication bias

k. I squared value of 67% indicating moderate heterogeneity

l. I squared value of 53% indicates moderate heterogeneity

m. Total sample size of 209, which is unlikely to meet the optimal information size

n. Funnel plot unable to be completed based on number of studies (<10), however 2/3 studies had funding sources which may have resulted in publication bias

o. I squared value of 74%, indicating moderate heterogeneity

p. I squared value of 44%, indicating moderate heterogeneity

q. I squared of 44%, which indicates moderate heterogeneity

r. Egger's test indicates funnel plot asymmetry, suggesting the presence of small study effects which may have been attributable to publication bias.

s. Egger's test indicates funnel plot asymmetry, suggesting the presence of small study effects which may have been attributable to publication bias.

# **Supplementary Figure 1:** Difference in HDL cholesterol (mmol/L) between nut consumption and control. Diamond indicates weighted mean difference with 95% CIs.

Abbreviation: IV, inverse variance.

# **Supplementary Figure 2:** Difference in triglycerides to HDL cholesterol ratio between nut consumption and control. Diamond indicates weighted mean difference with 95% CIs.

Abbreviation: IV, inverse variance.

**Supplementary Figure 3:** Difference in HDL cholesterol to LDL cholesterol ratio between nut consumption and control. Diamond indicates weighted mean difference with 95% CIs.

Abbreviation: IV, inverse variance.

# **Supplementary Figure 4:** Difference in apoA-I (mg/dL) between nut consumption and control. Diamond indicates weighted mean difference with 95% CIs.

Abbreviation: IV, inverse variance.

# **Supplementary Figure 5:** Difference in SBP (mmHg) between nut consumption and control. Diamond indicates weighted mean difference with 95% CIs.

Abbreviation: IV, inverse variance.

**Supplementary Figure 6:** Difference in DBP (mmHg) between nut consumption and control. Diamond indicates weighted mean difference with 95% CIs.

Abbreviation: IV, inverse variance.

# **Supplementary Figure 7:** LDL cholesterol funnel plot

# **Supplementary Figure 8:** HDL cholesterol funnel plot

# **Supplementary Figure 9:** TC funnel plot

# **Supplementary Figure 10:** TG funnel plot

# **Supplementary Figure 11:** TC:HDL cholesterol funnel plot

# **Supplementary Figure 12:** LDL cholesterol:HDL cholesterol funnel plot

# **Supplementary Figure 13:** ApoB funnel plot

# **Supplementary Figure 14:** ApoA-I funnel plot

# **Supplementary Figure 15:** SBP funnel plot

# **Supplementary Figure 16:** DBP funnel plot
